# Viscosity and Thermal Conductivity Models of 151 Common Fluids Based on Residual Entropy Scaling and Cubic Equations of State

**DOI:** 10.1021/acsomega.4c10815

**Published:** 2025-02-08

**Authors:** Xiaoxian Yang

**Affiliations:** Applied Thermodynamics, Chemnitz University of Technology, Chemnitz 09107, Germany

## Abstract

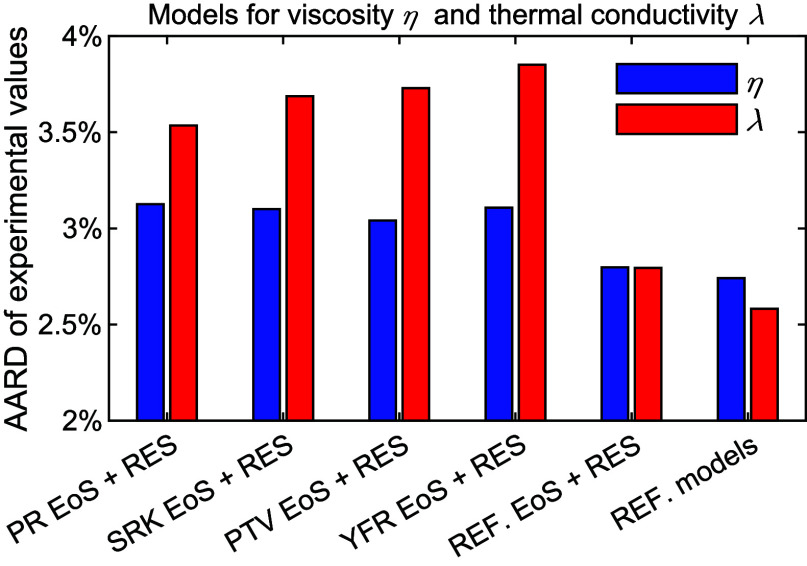

A residual entropy scaling (RES) approach combined with the cubic equation of state (EoS) was developed to calculate the viscosity and thermal conductivity of 151 common fluids. These pure fluids are all the pure fluids available in the NIST’s REFPROP 10.0 database. Seven cubic EoS were studied, while only four yielded good and similar results; these are Peng–Robinson (PR), Soave–Redlich–Kwong (SRK), Patel–Teja–Valderrama (PTV), and Yang–Frotscher–Richter (YFR) EoS. The parameters of a pure fluid in this cubic EoS + RES approach were fitted using experimental data if they are available in the NIST ThermoData Engine database 10.1, otherwise, using the calculations of REFPROP 10.0. This approach is applicable in the entire temperature and pressure ranges for thermal conductivity and at pressures lower than 60 MPa for viscosity. Using this approach, the average absolute value of the relative deviation (AARD) of all of the analyzable experimental values from model calculations was approximately 3.1% and 3.6% for viscosity and thermal conductivity, respectively. This result is not too bad compared to 2.7% and 2.5% obtained by the state-of-the-art viscosity and thermal conductivity models in REFPROP 10.0. The key advantage of this approach is that it has a much simpler equation form and can be easily extended to more fluids. The developed approach has been implemented in the OilMixProp 1.0 software package, and this work will be a basis for the future development of more than 600 pure fluids.

## 1 Introduction

The residual entropy scaling (RES) approach expresses residual transport properties (for example, viscosity, thermal conductivity, and self-diffusion coefficient) in terms of residual entropy, a thermodynamic property that can be calculated directly from the equations of state (EoS). A residual transport property is typically defined as the actual property minus the property at the dilute gas limit at the same temperature (for thermal conductivity, the critical enhancement term needs to be subtracted as well), while a residual thermodynamic property (such as entropy and enthalpy) is the actual property minus the ideal gas property at the same temperature and density. In fluid thermodynamics, for a pure fluid, an EoS is a mathematical relation between 3 intensive state variables. The most well-known EoS are the cubic ones which express pressure as a function of temperature and molar volume, i.e., *p* = *f*(*v*, *T*). An EoS alone can calculate residual thermodynamic properties and together with a proper RES approach can calculate residual transport properties.

The RES approach was first investigated by Rosenfeld^1^ in 1977 who introduced a macroscopically scaled viscosity and mapped the 3-dimensional (3-D) plot of viscosity vs temperature vs pressure (or density) into a 2-D plot of macroscopically scaled residual viscosity vs residual entropy. On the basis of this approach, the experimental and/or simulated data of a pure fluid collapse into a single curve in the dense region but diverge near the dilute gas limit in the 2-D plot; this makes it hard to model. Later, various approaches were developed to scale the viscosity and entropy to improve the performance near the dilute gas limit. A review was given by Liu et al.^2^ In these approaches, the one proposed by Bell et al.^3^ in 2019 is among the most successful ones. Inspired by the theoretical work of Rosenfeld^4^ in 1999, Bell et al.^3^ introduced a plus-scaled residual viscosity and yielded that the simulated data of a Lennard–Jones (L–J) fluid collapse into a single curve in the 2-D plot of plus-scaled residual viscosity vs residual entropy. The single curve can be easily correlated using a low-power equation (e.g., a polynomial equation), thus making modeling of viscosity much easier.

Based on the work of Bell et al.^3^ and the various multiparameter EoS available in the NIST REFPROP database 10.0,^5^ Yang and his colleagues^6−10^ extended and applied the RES approach of 39 refrigerants^6,7^ to all fluids^8−10^ available in REFPROP 10.0^5^ (148 fluids originally implemented in the software, and additional 3 fluids obtained from Bruno et al.).^11^ Especially, Martinek et al.^10^ in 2025 proposed a refined RES approach for viscosity that has an accuracy very close to those various state-of-the-art models implemented in REFPROP 10.0. The RES approach has great potential to become reference models for transport properties of many pure fluids because (1) it has good accuracy with simpler equation forms and fewer parameters than most of other models, (2) its accuracy can be increased with the improvement of the underlying EoS, and (3) it is much faster to be developed and evaluated than the multiparameter models in REFPROP 10.0.

However, the RES approach developed by Yang and his colleagues^6−10^ is limited to only 151 fluids whose multiparameter EoS are available in REFPROP 10.0. Furthermore, a brief literature review as listed in Table 1 reveals that, so far, only approximately 300 pure fluids can be modeled with the RES approaches, which are mainly limited by the adopted EoS (multiparameter EoS in REFPROP 10.0,^5^ PC-SAFT,^12^ or CPA^13^ etc.). For a pure fluid, these relatively accurate EoS generally have more adjustable parameters, for which more experimental data are required to fit. Therefore, they are not suitable or even capable to study fluids with very few experimental data. In this context, cubic EoS, having a reasonable balance of simplicity, accuracy, and generality, are a suitable solution for such fluids, because only critical point information (mainly critical pressure *p*_c_ and critical temperature *T*_c_) and, for most popular cubic EoS, the acentric factor ω are needed. For many pure or quasi-pure fluids (such as lubricant oils), the critical point information can be easily fitted with the Rackett equation^14^ or the modified Rackett equation^15^ using two density data at different temperatures, and the acentric factor ω can be fitted with the cubic Patel–Teja–Valderrama (PTV)^16,17^ or Yang–Frotscher–Richter (YFR)^18^ EoS using the same two density measurements.^15^ However, as shown in the literature review in Table 1, cubic EoS are marginally investigated for viscosity and thermal conductivity modeling based on RES.

**Table 1:** List of Selected Publications Using Residual Entropy Scaling

Property^a^	Fluid^b^	Equation of state^c^	Authors and year
η	refrigerants, other working fluids	multiparameter in REFPROP^5^	Bell and Laesecke 2016^20^
η	8 pure fluids and 4 model potentials	multiparameter in REFPROP^5^	Bell 2019^21^
η, λ, *D*	L–J fluids	multiparameter^22^	Bell et al. 2019^3^
η	propane	multiparameter^23^	Bell et al. 2020^24^
η	normal alkanes	multiparameter, PR,^25^ PC-SAFT^12^	Bell et al. 2020^26^
η	krypton	multiparameter^27^	Polychroniadou et al. 2021^28^
η	model potentials of CO_2_	multiparameter in REFPROP^5^	Bell et al. 2022^29^
η	hydrogen, deuterium, and neon	multiparameter in REFPROP^5^	Bell et al. 2023^30^
η, λ, *D*	simple monatomic fluids	multiparameter^22^	Saric et al. 2024^31^
η	a few refrigerants and blends	multiparameter in REFPROP^5^	Yang et al. 2021^32^
η	39 refrigerants	multiparameter in REFPROP^5^	Yang et al. 2021^7^
λ	39 refrigerants	multiparameter in REFPROP^5^	Yang et al. 2021^6^
λ	a few refrigerants and blends	multiparameter in REFPROP^5^	Kim et al. 2021^33^
η	HC mixtures	multiparameter in REFPROP^5^	Al Ghafri et al.^34^
η	124 pure fluids	multiparameter in REFPROP^5^	Yang et al. 2022^8,35^
η, λ	lubricant oils and mixtures	PR,^25^ SRK,^36,37^ PTV^16,17^	Yang et al. 2023^15^
η	124 pure fluids	multiparameter in REFPROP^5^	Martinek et al. 2025^10^
λ	125 pure fluids	multiparameter in REFPROP^5^	Li et al. 2024^9^
η, λ, Pr	HFC and HFO refrigerants	PC-SAFT^12^	Fouad and Vega 2018^38^
η, σ	a few refrigerants and blends	PC-SAFT^12^	Fouad and Vega 2018^39^
η, *D*	a few refrigerants	PC-SAFT^12^	Fouad and Alasiri 2020^40^
λ	12 refrigerant and their blends	PC-SAFT^12^	Fouad 2020^41^
η	HFC and HFO refrigerants	CPA^13^	Liu et al. 2020^42^
λ	HFC and HFO refrigerants	CPA^13^	Liu et al. 2021^43^
η, λ	CO_2_	multiparameter,^44^ volume-translated SRK^45,46^	Liu et al. 2022^2^
η	110 pure fluids	PCP-SAFT^47,48^	Lötgering-Lin and Gross 2015^49^
λ	water and 147 organic substances	PC-SAFT^12^	Hopp and Gross 2017^50^
η	140 pure fluids and 566 mixtures	PCP-SAFT^47,48^	Lötgering-Lin et al. 2018^51^
*D*	pure substances	PCP-SAFT^47,48^	Hopp et al. 2018^52^
λ	231 pure fluids	PCP-SAFT^47,48^	Hopp and Gross 2019^53^
λ	approximately 20 pure fluids	PCP-SAFT^47,48^	Hopp et al. 2019^54^
η, λ, *D*	fluid in TAMie force field	PC-SAFT^12^	Fischer et al. 2020^55^
η	refrigerants	PC-SAFT^12^	Kang et al. 2022^56^
λ	pure refrigerants and mixtures	PC-SAFT^12^	Kang et al. 2022^57^
η	11 pure refrigerants	PC-SAFT^12^	Li et al. 2022^58^
η	HFC, HFO refrigerants	PC-SAFT^12^	Kang et al. 2024^59^
η	alkanes	multiparameter in REFPROP^5^	Jäger et al. 2023^60^
η	branched alkanes	LKP^61,62^ and PC-SAFT^12^	Mickoleit et al. 2023^63^
η	HC and HC + CO_2_	multiparameter in REFPROP^5^	Binti Mohd Taib and Trusler 2020^64^
λ	CO_2_, CO_2_ + *n*-decane CO_2_ + water,	RG^65^ + CPA^13^	Zhu et al. 2020^66^
η	natural gas, its 21 pure components	GERG-2008^67^	Mairhofer 2021^68^
η	142 pure compounds	*I*-PC-SAFT,^69^*translated consistent*-PR^70^	Dehlouz et al. 2021^71,72^
λ	119 pure compounds	*I*-PC-SAFT,^69^*translated consistent*-PR^70^	Dehlouz et al. 2022^73,74^
*D*	72 pure compounds	*I*-PC-SAFT,^69^*translated consistent*-PR^70^	Dehlouz et al. 2022^75^
λ	HC mixtures and diesel fuels	PC-SAFT^12^	Rokni et al. 2019^76^
η	HC mixtures and diesel fuels	PC-SAFT^12^	Rokni et al. 2019^77^
η	CF3I	multiparameter	Tuhin et al. 2024^78^

a Property: viscosity η, thermal conductivity λ, self-diffusion coefficient *D*, surface tension σ, Prandtl’s number Pr.

b Abbreviation: hydrofluorocarbon (HFC), hydrofluoroolefin (HFO), and hydrocarbon (HC).

c Abbreviation: Peng–Robinson (PR), Soave–Redlich–Kwong (SRK), Lee–Kesler–Plöcker (LKP), cubic-plus-association (CPA), perturbed-chain polar statistical associating fluid theory (PCP-SAFT), perturbed-chain statistical associating fluid theory (PC-SAFT), renormalization group (RG).

Therefore, the aim of this work is to develop and evaluate viscosity and thermal conductivity models of 151 common fluids based on the combination of RES and some selected cubic EoS. The RES approaches developed and the experimental data of 125 pure fluids collected in our previous work^8−10^ are the basis of this work. The accuracy, reliability, and limitations of this new cubic EoS + RES approach will be investigated, and this work will be the foundation for the future development of the viscosity and thermal conductivity models of more than 600 pure fluids. This overall effort is part of the KETEC (Research Platform for Refrigeration and Energy Technology) project.^19^

## 2 Models

In this section, viscosity and thermal conductivity models of pure fluids are presented based on the combination of the RES approach of Yang et al.^6,7^ and a generalized three-parameter cubic EoS. Compared to models based on complicated EoS, such as multiparameter and PC-SAFT, the presented models here are relatively easy to reproduce and implement into existing software tools. Moreover, the dilute gas properties are calculated using simple polynomial equations. They are also easy to reproduce and in general yield slightly higher accuracy than those typically used in the literature, i.e., the Chapman–Enskog^79^ solution for viscosity and the Chichester and Huber^80^ solution for thermal conductivity. Mixtures can be predicted with proper mixing rules: van der Waals mixing rules for cubic EoS, those developed by Yang et al.^8^ for the viscosity RES model and by Li et al.^9^ for the thermal conductivity RES model.

### 2.1 Viscosity Model

Pure fluid viscosity η is calculated as the sum of dilute gas viscosity η_ρ*→*0_ and the residual part η^r^:
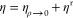
1

Here η_ρ→0_ is calculated with a polynomial equation:^10^
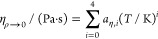
2where *T* is the temperature and parameters *a*_*η,i*_ (*i* = 0,1,2,3,4) were fitted to the calculations of REFPROP 10.0^5^ for each pure fluid from triple point temperature to generally 1000 K or the highest calculable temperature. These values have been published in Supporting Information of Martinek et al.,^10^ provided again in Supporting Information of this paper, and are implemented in the OilMixProp 1.0 software package^81^ (a tool for computing essential thermophysical properties of user-defined oils, common fluids, and their mixtures).

The residual viscosity η^r^ can be calculated with,^3,8,10^
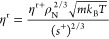
3
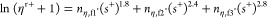
4
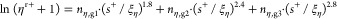
5
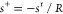
6
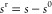
7

Here, ρ_N_, in unit of m^–3^, is the number density, *m*, in unit of kg, is the mass of one molecule, *k*_B_ = 1.380649 × 10^–23^ J·K^–1^ is the Boltzmann constant, *s*^r^, in unit of J·mol^–1^·K^–1^, is the residual entropy being the actual entropy *s* minus the ideal gas entropy *s*^0^ at the same temperature and density, and *R* = 8.31446261815324 J·mol^–1^·K^–1^ is the molar gas constant. The number density ρ_N_ and the residual entropy *s*^r^ can be calculated with cubic EoS, which is discussed in Section 2.3. Parameters *n*_*μ*,f*i*_ and *n*_*μ*,g*i*_ (*i* = 1,2,3) are fluid-specific parameters for each fluid and group-specific parameters for each group of similar fluids, respectively. Each pure fluid belongs to a fluid group classified by Yang et al.^8^ and has a corresponding fluid-specific scaling factor *ξ*_*μ*_. Only fluids with adequately good quality data can have the fluid-specific parameters *n*_*μ*,f*i*_ fitted. To use this model, fluid-specific parameters *n*_*μ*,f*i*_ are suggested and only if they are not available, group-specific parameters *n*_*μ*,g*i*_ with fluid-specific scaling factor *ξ*_*μ*_ are used.

### 2.2 Thermal Conductivity Model

Fluid thermal conductivity is calculated as the sum of the dilute gas term *λ*_*ρ*→0_, the residual part λ^r^ and the critical enhancement term Δλ_c_:
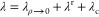
8

Here *λ*_*ρ*→0_ is calculated with a polynomial equation:^9^
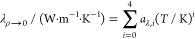
9where parameters *a*_*λ,i*_ (*i* = 0,1,2,3,4) were determined in the same way as *a*_*η,i*_ in eq 2, published in the Supporting Information of Li et al.,^9^ provided again in the Supporting Information of this paper, and implemented in the OilMixProp 1.0.^81^

The residual thermal conductivity λ^r^ can be calculated with^3,6^
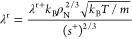
10
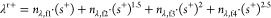
11
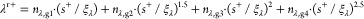
12

Parameters *n*_*λ*,f*i*_, *n*_*λ*,g_*_i_,* and *ξ*_*λ*_ (*i* = 1,2,3,4) for thermal conductivity, similar to *n*_*μ*,f*i*_, *n*_*μ*,g_*_i_,* and *ξ*_*μ*_ for viscosity, are fluid-specific parameters of each fluid, group-specific parameters of each group, and fluid-specific scaling factor, respectively. The classification of each fluid group is the same as our previous work^9^ and the way these parameters are used is the same as those for viscosity.

The critical enhancement of thermal conductivity Δλ_c_, which needs to be taken into consideration especially at the vicinity of the critical point, is calculated according to a crossover model proposed by Olchowy and Sengers,^82^ and has been well summarized in literature.^6^ In this crossover model, calculations of isobaric and isochoric heat capacity *c*_*p*_ and *c*_*v*_ require an EoS and an equation for the ideal gas isobaric heat capacity  as a function of temperature. In this work, a linear equation of (*T*) is used
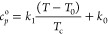
13where *k*_0_ is the value of  at *T*_0_ = 298.15 K, and *k*_1_/*T*_c_ is the derivative of  with respect to temperature at *T*_0_ = 298.15 K. These values were determined in the same way as *a*_*η,i*_ in eq 2 and *a*_*λ,i*_ in eq 9, given in Supporting Information of this work, and has been implemented in OilMixProp 1.0.^81^ The calculation details for *c*_*p*_ and *c*_*v*_ are more or less textbook knowledge and have been given in the Supporting Information of Yang et al.^83^

### 2.3 Generalized Three-Parameter Cubic EoS

The generalized three-parameter cubic equation of state (EoS) is^16,17^
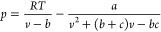
14where *p* is the pressure, *v* is the molar volume, and parameters *a*, *b,* and *c* are calculated as
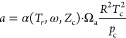
15
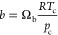
16
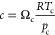
17

Here *T*_r_ = *T*/*T*_c_ is the reduced temperature, ω is the acentric factor, and *Z*_c_ is the compressibility factor at the critical point. The critical point information were obtained from REFPORP 10.0 and is given in the SI of this paper. The function α(*T*_r_, ω, *Z*_c_) and parameters Ω_a_, Ω_b_ and Ω_c_ are different for each studied EoS (see Section 2.4). Defining terms:
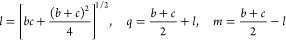
18then molar residual entropy *s*^r^ can be calculated with
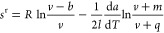
19

Here, derivatives d*a*/d*T* are calculated according to eq 15.

### 2.4 The Studied Cubic EoS

The studied EoS includes: Peng–Robinson (PR),^25^ Peng–Robinson-Stryjek-Vera (PRSV),^84^ Redlich–Kwong (RK),^37^ Soave–Redlich–Kwong (SRK),^36^ Wilson-Redlich–Kwong (WRK),^85^ PTV,^16,17^ and YFR EoS.^18^ The function of α(*T*_r_, ω, *Z*_c_) and the values of Ω_a_, Ω_b_ and Ω_c_ for each cubic EoS are given in Tables 2 and 3. Here, the YFR is a new cubic EoS developed in our research group aided by symbolic regression tools^86−89^ for improved liquid phase density calculation. It is exceptionally good at fitting fluid constants (mainly *T*_c_, *p*_c_ and ω) using very few liquid density data,^15^ and will be an essential tool for future development of viscosity and thermal conductivity models of pure or quasi-pure fluids with very few experimental data. Note that although seven cubic EoS were studied, three will not be further discussed in this work, as they all yield a relatively worse result due to RK and WRK are too simple and PRSV has no parameter *m*_1_ (see Table 2) for all fluids studied.

**Table 2:** Summary of the Studied Cubic Equations of State

EoS^a^	Ω_a_	Ω_b_	Ω_c_	α
PR	0.45724	0.07779	0.07779	α = (1 + *m*·(1–*T*_r_^1/2^))^2^
				*m* = 0.37464 + 1.54226ω – 0.26992ω^2^ (ω ≤ 0.49)
				*m* = 0.37964 + 1.48503ω – 0.164423ω^2^ + 0.016666ω^3^ (ω > 0.49);
RK	0.42748	0.08664	0	α = *T*_r,*i*_^–1/2^;
PTV	0.66121 – 0.761057·*Z*_C_	0.02207 + 0.20868·*Z*_C_	0.57765 – 1.87080·*Z*_C_	α = (1 + *m*·(1 – *T*_r_^1/2^))^2^; *Z*_C_ is compression factor at critical point
				*m* = 0.46283 + 3.58230ω*Z*_C_ + 8.19417(ω*Z*_C_)^2^;
PRSV	0.45724	0.07779	0.07779	α = (1 + *m*·(1 – *T*_r_^1/2^))^2^
				*m* = *m*_0_ + *m*_1_·(1 + *T*_r_^1/2^)·(0.7 – *T*_r_)
				*m*_0_ = 0.378893 + 1.4897153ω – 0.17131848ω^2^ + 0.0196554ω^3^
				*m*_1_ = 0 (*T*_r_ > 0.7); otherwise value in Table 1 of Ref. (84)
SRK	0.42748	0.08664	0	α = (1 + *m*·(1 – *T*_r_^1/2^))^2^
				*m* = 0.48508 + 1.55171ω – 0.15613ω^2^;
WRK	0.42748	0.08664	0	α = *T*_r_·(1 + *m*·(*T*_r_^–1^ – 1))
				*m* = 1.57 + 1.62ω
YFR^b^	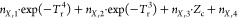			α = (1 + *m*·(1 – *T*_r_^1/2^))^2^;
	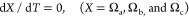			*m* = 2.779200*Z*_C_ + 5.208803ω*Z*_C_ – 0.314477;

a Peng–Robinson (PR),^25^ Redlich–Kwong (RK),^37^ Patel-Teja-Valderrama (PTV),^16,17^ Peng–Robinson-Stryjek-Vera (PRSV),^84^ Soave–Redlich–Kwong (SRK),^36^ Wilson-Redlich–Kwong (WRK),^85^ and Yang–Frotscher–Richter(YFR) EoS.^18^

b *X* refers to Ω_a_, Ω_b_, and Ω_c_, respectively with parameter given in Table 3.

**Table 3:** Parameters of the Yang–Frotscher–Richter (YFR) EoS^18^

	*i* = 1	*i* = 2	*i* = 3	*i* = 4
*n*_Ω*a*,*i*_	–0.174696	0.156625	–1.158565	0.784751
*n*_Ω*b*,*i*_	0.048371	–0.043334	0.319103	–0.012341
*n*_Ω*c*,*i*_	–0.434682	0.389505	–2.872362	0.889348

## 3 Results

To better present and explain the results, two statistical terms are defined here. The average relative deviation (ARD) and the average absolute value of the relative deviation (AARD) of the experimental values *X*_exp_ (*X* refers to η or λ) from model calculations *X*_model_ are
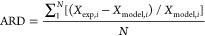
20
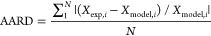
21where *N* is the total number of experimental data points for a given fluid. ARD and AARD represent the systematic offset and scatter, respectively, from the experimental data to the model.

### 3.1 Data Processing and Parameter Fitting

The methods for the experimental data processing and the RES model parameter fitting (fluid-specific parameter, group-specific parameter, and fluid-specific scaling factor) are almost the same as in our previous work for viscosity^8^ and thermal conductivity.^9^ The key differences here are (1) for density and residual entropy calculation, cubic EoS was used instead of the multiparameter EoS in REFPROP 10.0, and (2) a fourth filter (see Section 3.2) was applied to viscosity data. In this context, this section will only be briefly introduced, and most of the resulting figures are given in Supporting Information.

**Figure 1:**
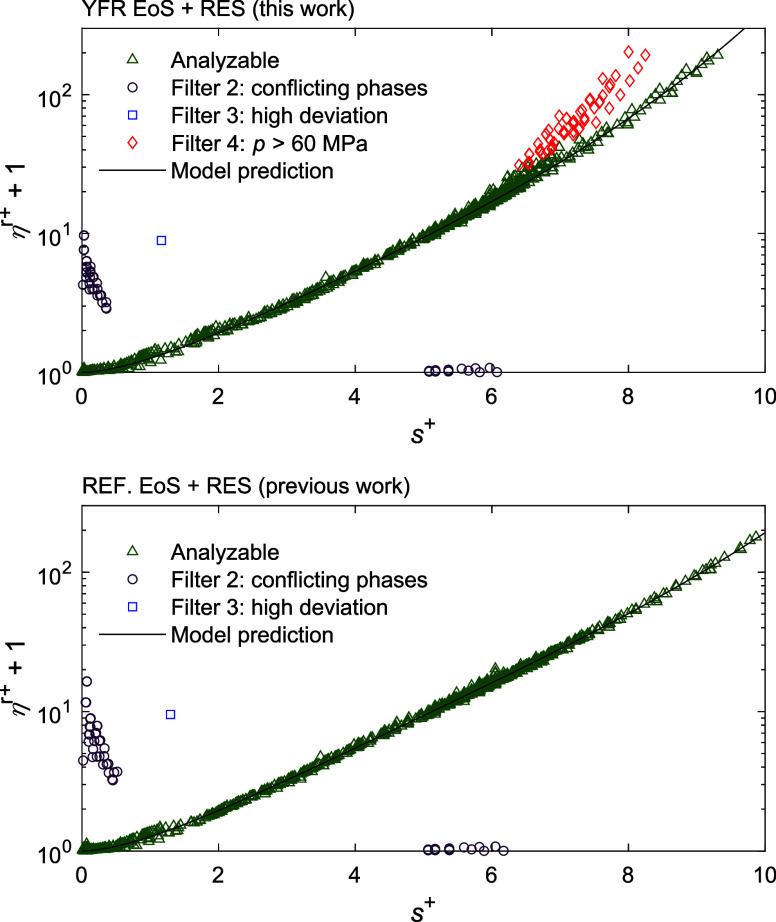
Experimental data of hexane plotted in plus-scaled dimensionless residual viscosity η^*r*+^ vs plus-scaled residual entropy *s*^+^ to illustrate the function of each filter. Data filtered out by filter 1 cannot be plotted because either density or residual entropy cannot be calculated. The results of the RES approach combined with multiparameter EoS in REFPROP 10.0 (REF. EoS) were obtained from our previous work.^8^

Approximately 52,000 experimental viscosity values of 124 pure fluids and 72,000 experimental thermal conductivity values of 125 pure fluids collected in our previous work^8,9^ mainly from the NIST ThermoData Engine (TDE) database 10.1^90^ were adopted for this work. Detailed citation information is provided in Supporting Information of our previous work.^8,9^ The same three filters are first applied to the raw experimental data: filter 1 removes data which exceed the limitation of fluid’s EoS (e.g., data with temperature below triple point) in calculating density and residual entropy; filter 2 removes data in conflicting phases (e.g., the fluid is in the gas phase according to the given temperature and pressure, but the given high viscosity values suggest it to be in the liquid phase); and filter 3 removes outliers whose relative deviation from the model prediction is larger than 30%. Data removed by filter 3 were determined in iteration between filtering and model parameter fitting. A fourth filter, data with pressure values higher than 60 MPa were removed, was applied to viscosity data; the reason for this is discussed in Section 3.2. Figure 1 illustrates the performance of these filters, with the viscosity of hexane taken as an example. This figure was generated using the YFR EoS, and in the following discussions, all the calculations presented were based on this cubic EoS as well. Unless otherwise stated, other three cubic EoS yielded similar results. Data filtered by filter 1 are not shown in this figure because either the density or the residual entropy cannot be calculated.

For each of the four cubic EoS and each pure fluid, the RES parameters (fluid-specific parameter, group-specific parameter, and fluid-specific scaling factor) for both viscosity and thermal conductivity were fitted using the analyzable experimental data; i.e., data pass all filters. In this step, parameters were obtained for the viscosity of 124 pure fluids and for the thermal conductivity of 125 pure fluids. For approximately 26 additional pure fluids (=151–125), which have multiparameter EoS in REFPROP 10.0 but no experimental data were collected, their group number and fluid-specific scaling factor were determined by the following steps. (1) Calculating reference data for a pure fluid in both liquid and gas phases with REFPROP 10.0; (2) using these reference data to fit the fluid-specific scaling factors ξ of this fluid in each group; (3) assigning the group number of this fluid in which the fluid’s ξ is closest to 1. In this way, the proposed RES model for approximately 26 fluids was anchored to the reference viscosity or thermal conductivity models in REFPROP 10.0. This can be easily improved when reliable experimental data on these fluids are available. The values of these fitted parameters of the 151 pure fluids using each of the four cubic EoS are given in Supporting Information and have been implemented in the OilMixProp 1.0 software package.

Similar to our previous work,^8,9^ the ln(η^*r*^^+^+1) vs *s*^+^/ξ_η_ plot for viscosity and λ^*r*^+ vs *s*^+^/ξ_λ_ plot for thermal conductivity of each pure fluid and each group of fluids using each cubic EoS are generated; they are also provided in Supporting Information. As expected, each curve generally collapses into a single curve except for a few outliers (see Section 3.2) for viscosity. The figures showing the relative deviation of each analyzable experimental data from the calculation of the RES model are given in Supporting Information as well. In the following sections, only statistical results are given.

### 3.2 Viscosity

**Figure 2:**
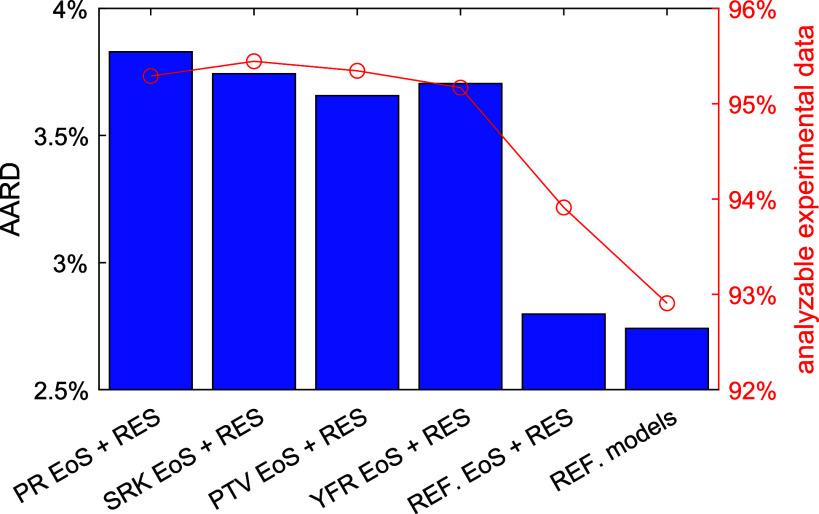
AARD of the analyzable experimental viscosity data (passing the three filters) from model predictions. The first five models are RES approaches combined with the PR, SRK, PTV, YFR, and multiparameter EoS in REFPROP 10.0, respectively. REF. models are the default viscosity models in REFPROP 10.0. Red circle: ratio of the analyzable experimental data to all of the collected data.

In the first step, only three filters as used in our previous work^8^ were applied to the experimental viscosity data for the cubic EoS + RES approach. The AARD of all analyzable experimental data (passing all three filters) of six viscosity models is shown in Figure 2. The first four models are the cubic EoS + RES approaches, based on the PR, SRK, PTV, and YFR EoS, respectively. The fifth one (“REF. EoS + RES” in Figure 2) is also an RES approach but combined with the various multiparameter EoS in REFPROP 10.0. The last one (“REF. models” in Figure 2) is the default state-of-the-art viscosity model in REFPROP 10.0. The results of the last two models were obtained from our previous work.^10^ Despite simplicity, the results, AARD = 3.7% to 3.8%, obtained by the four cubic EoS + RES approaches are not too bad compared to the state-of-the-art method (AARD ≈ 2.7%). Besides, as cubic EoS have larger applicable temperature and pressure ranges than the multiparameter EoS, more than 95% of all the experimental data pass the 3 filters, while only 94% and 93%, respectively, pass if REF. EoS + RES and REF. models are used. Among the four cubic EoS, the PTV EoS yields the lowest AARD followed by the YFR EoS, whereas the SKR EoS has the largest amount of analyzable data followed by the PTV EoS. Nonetheless, performances among these four cubic EoS are quite similar. For each pure fluid and each group of fluids, the performance comparison (AARD and analyzable experimental data) between these six models is illustrated in each individual figure similar to Figure 2 and provided in Supporting Information. Although slight differences in performance were observed, no cubic EoS obviously outperforms the others in any particular fluid or group.

**Figure 3:**
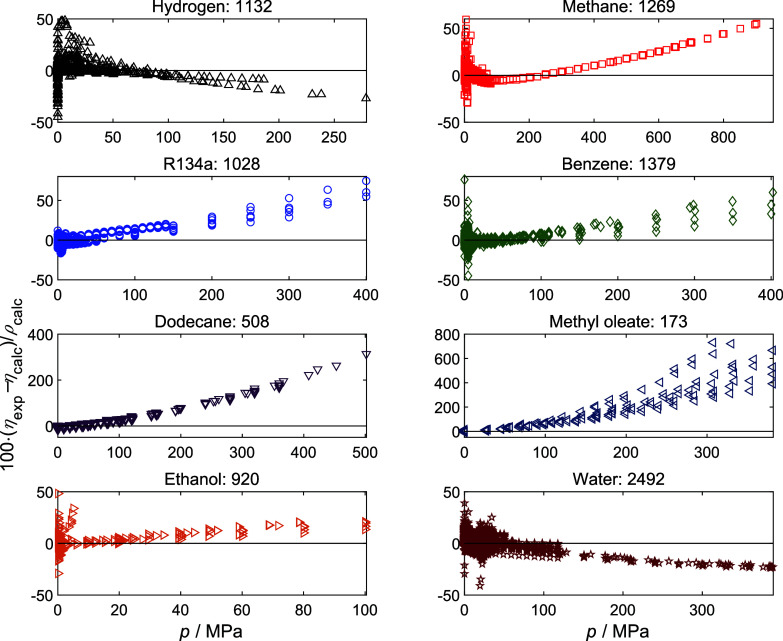
Relative deviation of the experimental viscosity from the YFR EoS + RES approach as a function of pressure. The number following the fluid name is the number of experimental data that pass filters 1 and 2. Note that hydrogen was considered as a pure component here, although hydrogen itself is a mixture of its spin isomers.

The reason for the higher AARD (as shown in Figure 2) of the cubic EoS and RES approaches was further investigated. One can first observe from Figure 1 that there are a lot more outliers in the high residual entropy range (top figure) than that yielded by the RES + multiparameter EoS approach (bottom figure). Taking example pure fluids from each group, Figure 3 illustrates the relative deviation of the experimental viscosity data that pass filters 1 and 2 from model predictions as a function of pressure. A clear trend of increasing the absolute value of relative deviation with increasing pressure was observed. The relative deviation at high pressures is particularly high for dense fluids, e.g., up to 300% for dodecane of group 5 and methyl oleate of group 6. Similar plots were generated but as functions of temperature, density, and residual entropy; however, no as clear a trend as of pressure was observed; see Supporting Information. This inspired the cubic EoS + RES approach for viscosity to be not applicable in the high-pressure range. This is mainly due to the fact that cubic EoS cannot calculate residual entropy accurately at high pressures.^18^

**Figure 4:**
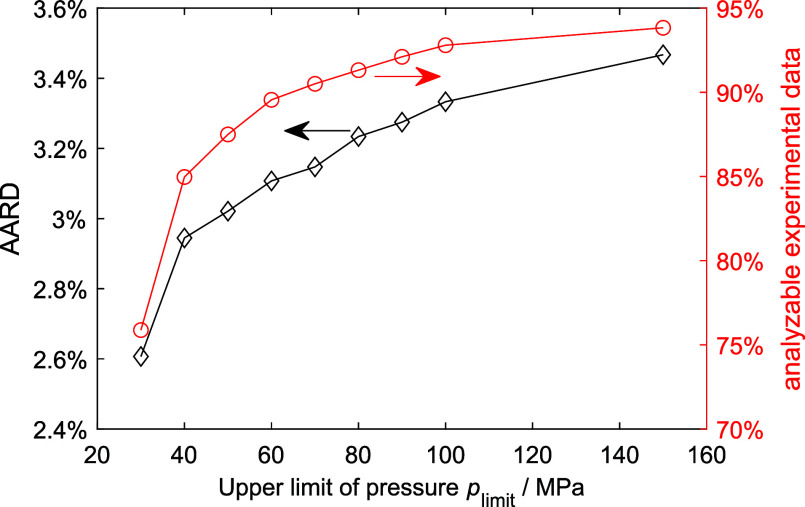
AARD and analyzable experimental data (passing all four filters) at different upper limits of pressures. The calculations are based on the YFR EoS + RES approach.

**Figure 5:**
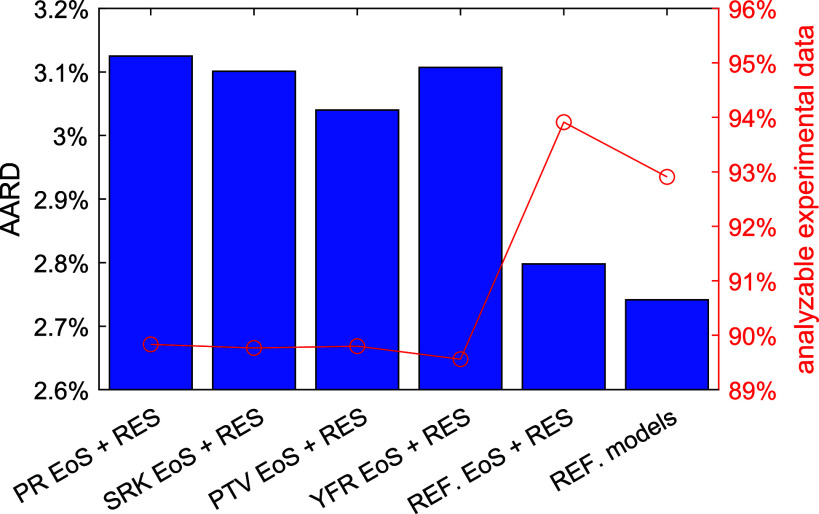
AARD of the analyzable experimental viscosity data (passing all filters) from the model predictions. Three filters were applied to the five RES approaches and the default viscosity models in REFPROP 10.0 (REF. models). A fourth filter (data with pressure values higher than 60 MPa were removed) were applied to the four cubic EoS + RES approaches. Red circle: ratio of the analyzable experimental data to all the collected data.

The next step is to determine the application range of the cubic EoS + RES approach for the viscosity. To achieve this, a fourth filter was introduced: experimental data with a pressure value higher than a pressure threshold, *p*_limit_, were filtered out. The AARD and the analyzable experimental data (passing all filters) at different *p*_limit_ values are shown in Figure 4. With decreasing *p*_limit_, more data are filtered out and the AARD decreases. Interestingly, if *p*_limit_ = 30 MPa, the cubic EoS + RES approach yields AARD as low as the state-of-the-art viscosity models, but unfortunately, only about 75% of the data pass all four filters. Compromising between the two conflicting targets: low AARD and high analyzable data, a *p*_limit_ of 60 MPa was eventually chosen as the upper limit of the application range. With *p*_limit_ = 60 MPa, there are still approximately 90% data pass all filters and the AARD was reduced to 3.1%, see Figure 4. Figure 5 shows the AARD of the analyzable experimental data of the six viscosity models. This figure is similar to that in Figure 2, except that the fourth filter was applied to the cubic EoS + RES approach. The four cubic EoS still yield similar performance: AARD is approximately 3.1%, and 90% of data pass all filters.

**Figure 6:**
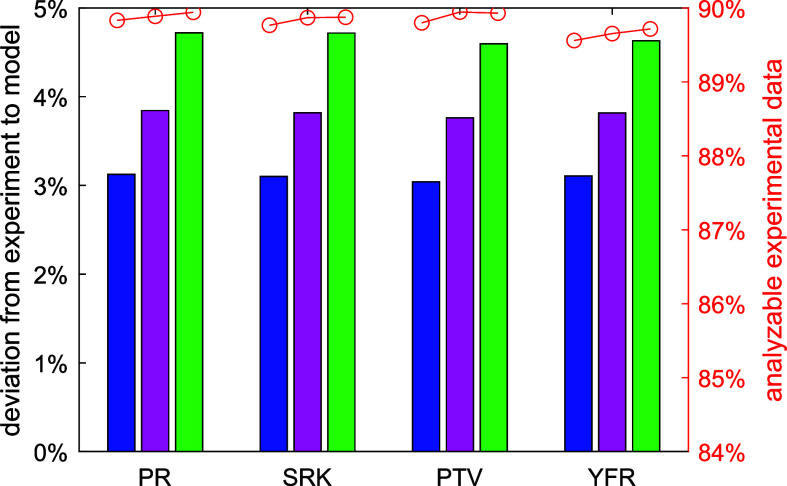
AARD of the analyzable experimental viscosity data (passing all four filters) from the cubic EoS + RES approach using different EoS and different dilute gas viscosity calculation methods. Blue bar: polynomial eq 2; purple: Chapman–Enskog^79^ solution with fitted L-J parameters; green bar: Chapman–Enskog^79^ solution with L-J parameters estimated by critical point information; red circle: ratio of the analyzable experimental data to all the collected data.

In the last step, in addition to the one described in Section 2, two more dilute gas viscosity η_ρ→0_ calculation methods were studied. One is based on the Chapman–Enskog^79^ solution of the Boltzmann transport equation, which requires two L-J parameters (the pair-potential energy ε/*k*_B_ and the collision diameter σ) of each pure fluid to calculate η_ρ→0_. Another is to estimate the two L-J parameters using critical point information (*T*_c_ and ρ_c_) based on the predictive correlations of Chung et al.^91^ Details of these two methods are given in our previous work.^10^ With the last method, η_ρ→0_ can be estimated using *T*_c_ and ρ_c_, instead of the polynomial equation eq 2 whose parameters are only available for the 151 fluids. These two methods are bridges to future work in which the current cubic EoS + RES approach will be extended to more fluids. The AARD of the analyzable experimental data (passing all four filters) from the model predictions is plotted in Figure 6 for the three η_ρ→0_ calculation methods and four cubic EoS. AARD is about 3.1%, 3.8%, and 4.7%, if η_ρ→0_ is calculated with the polynomial equation eq 2, the method based on L-J parameters, and the method based on critical point information, respectively. Although AARD = 4.7% seems to be high, it is still acceptable for fluids with very few experimental data, considering that typical viscosity experiment in industry has such a level of uncertainty or even higher.

### 3.3 Thermal Conductivity

**Figure 7:**
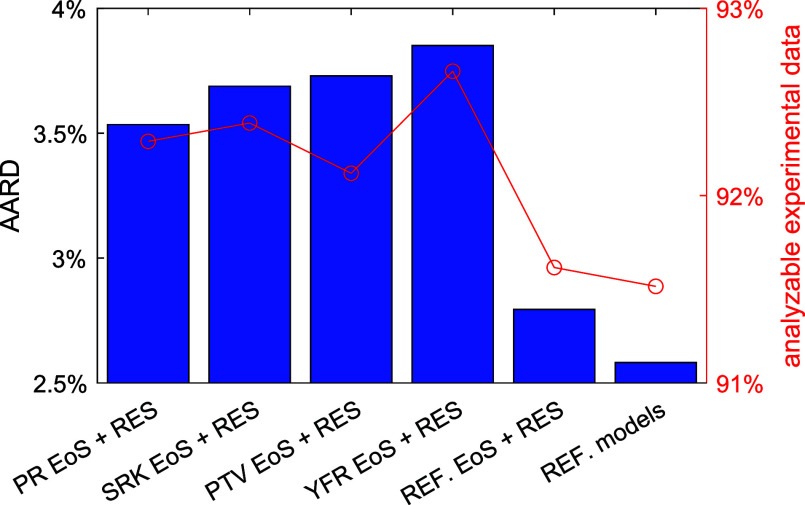
AARD of the analyzable experimental thermal conductivity data (passing all three filters) from model predictions. The first five models are based on the RES approach using PR, SRK, PTV, YFR, and multiparameter EoS in REFPROP 10.0, respectively. REF. models uses to the default thermal conductivity models in REFPROP 10.0. Red circle: ratio of the analyzable experimental data to all the collected data.

Similar to the study of viscosity, the AARD of all the analyzable experimental data (passing the three filters) from six thermal conductivity models are given in Figure 7. (The AARD and ARD for each pure fluid and each group are given in Supporting Information.) Four models are the cubic EoS + RES approaches based on PR, SRK, PTV, and YFR EoS, respectively. The remaining two are REF. EoS + RES (see Figure 7), an RES approach combined with multiparameter EoS in REFPROP 10.0 developed in our previous work,^9^ and REF. models were the default state-of-the-art thermal conductivity models in REFPROP 10.0. The cubic EoS + RES approaches for thermal conductivity yield higher AARD (3.5% to 3.8%) than the state-of-the-art model (AARD = 2.5% with REF. models), while more data are analyzable using the cubic EoS + RES approach. Among the four cubic EoS, the PR EoS yields the lowest AARD followed by SRK EoS, whereas YFR has the largest analyzable data, followed by SRK EoS. Nonetheless, performances among these four cubic EoS are relatively similar as well.

Similar to the study of viscosity, investigation about the worse performance (i.e., higher AARD as shown in Figure 7) of the cubic EoS + RES approaches for thermal conductivity was carried out. However, no obvious relations between the relative deviation (from the analyzable experimental data to model prediction) and other properties (pressure, temperature, density, and residual entropy) were observed. A similar pressure threshold (*p*_limit_ = 60 MPa) was applied but yielded a merely 0.1% reduction of AARD at a cost of 5% more data being filtered out. Therefore, for thermal conductivity, it is not necessary to apply filter 4 and there is no upper pressure limit in the application range of the cubic EoS + RES approach.

**Figure 8:**
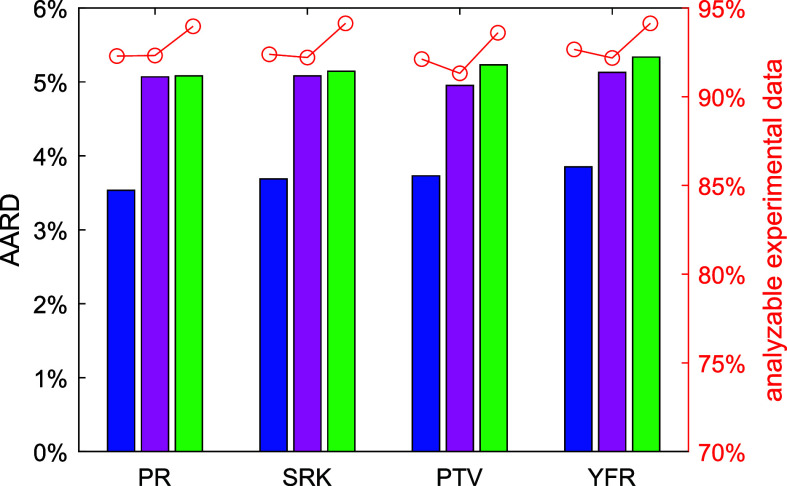
AARD of the analyzable experimental thermal conductivity data (passing all three filters) from the cubic EoS + RES approach using different EoS and different dilute gas thermal conductivity calculation methods. Blue bar: polynomial (eq 9); purple: Chichester and Huber^80^ with fitted L-J parameters; green bar, Chichester and Huber^80^ with L-J parameters estimated by critical point information; red circle: ratio of the analyzable experimental data to all the collected data.

Similar to the study of viscosity, two other dilute gas thermal conductivity λ_ρ→0_ calculation methods were studied. One is the method developed by Chichester and Huber,^80^ which requires values of the two L-J parameters (ε/*k*_B_ and σ) and ideal gas isobaric heat capacity . Another one is to estimate the two L-J parameters using critical point information (*T*_c_ and ρ_c_) based on the predictive correlations of Chung et al.^91^ This is a challenge but is outside the scope of the current work. The AARD of the analyzable experimental data (passing all three filters) from model predictions is plotted in Figure 8 for the three λ_ρ→0_ calculation methods and the four cubic EoS. The AARD is about 5.0% if λ_ρ→0_ is calculated with the last two methods, as compared to 3.5% if λ_ρ→0_ is calculated with the polynomial equation eq 9. Similar to viscosity, AARD = 5.0% is still acceptable for fluids with very few experimental data. Different from viscosity, to extend the current cubic EoS + RES approach to more fluids’ thermal conductivity, proper estimation of  is needed.

## 4 Conclusion, Discussion, and Future Work

In this work, a residual entropy scaling (RES) approach combined with four cubic equation of state (EoS) was developed for viscosity and thermal conductivity modeling of all pure fluids available in the NIST REFPROP database 10.0 (151 fluids). The four cubic EoS are Peng–Robinson (PR), Soave–Redlich–Kwong (SRK), Patel–Teja–Valderrama (PTV), and Yang–Frotscher–Richter (YFR) EoS. Three more cubic EoS were studied, but significantly worse results were obtained.

The model development was based on approximately 52,000 experimental viscosity values of 124 pure fluids and 72,000 experimental thermal conductivity values of 125 pure fluids collected mainly from the NIST ThermoData Engine (TDE) database 10.1. For the remaining fluids, which are implemented in REFPROP 10.0 but no experimental data were collected, the cubic EoS + RES approach was anchored to the reference viscosity and thermal conductivity models in REFPROP 10.0. In the model development, it was noticed that relative deviations from experimental viscosity data to predictions of the cubic EoS + RES approach increase with increasing pressure. Compromising between the two conflicting targets: high accuracy and high applicable range, an upper limit of pressure *p*_limit_ = 60 MPa was chosen as the application range of the cubic EoS + RES approach for viscosity.

The performance of the developed cubic EoS + RES approaches was evaluated by comparing with two other models: one is the RES approach combined with multiparameter EoS in REFPROP 10.0, and another is the default state-of-the-art viscosity and thermal conductivity models in REFPROP 10.0. The average of the absolute value of the relative deviation (AARD) of the analyzable experimental values (passing all filters) from model calculations was calculated. The AARD for viscosity and thermal conductivity are approximately 3.1% and 3.6%, respectively, using the cubic EoS + RES approach. These are not too bad compared to the 2.7% and 2.5% obtained by the state-of-the-art models. The advantages are that the cubic EoS + RES approach has much simpler functional forms and is more general: it can analyze more experimental data and can be easily extended to more fluids. The cubic EoS + RES approaches and the values of all of the fitted parameters obtained in this work have been implemented in the OilMixProp 1.0 software package. This work is a basis for future work in which the similar cubic EoS + RES approach for viscosity and thermal conductivity will be extended to more than 600 pure fluids.

## Supporting Information Available

Tables for parameters for dilute gas viscosity calculation, parameters for dilute gas thermal conductivity calculation, fluid constants, RES parameters for viscosity, RES parameters for thermal conductivity; figures for viscosity including: residual viscosity ln(η^*r*+^ + 1) vs residual entropy *s*^+^/ξ_η_, the relative deviation of each viscosity experimental point to the RES model, detailed comparison among different viscosity models, relative deviation of viscosity data vs other properties; figures for thermal conductivity including: residual thermal conductivity λ^*r*+^ vs residual entropy *s*^+^/ξ_λ_, relative deviation of each thermal conductivity experimental point to the RES model, detailed comparison among different thermal conductivity models (PDF)
Figures and tables supporting the Supporting Information PDF (ZIP)
