# Supplementary material for: Viscosity and Thermal Conductivity Models of 151 Common Fluids Based on Residual Entropy Scaling and Cubic Equations of State
Source: ACS Omega. 2025 Feb 8;10(6):6124–34. doi: 10.1021/acsomega.4c10815 (PMC11840589; doi:10.1021/acsomega.4c10815)
Supplement: Supplementary file 1 — ao4c10815_si_001.pdf [file ao4c10815_si_001.pdf]

Supporting Information

Viscosity and thermal conductivity models of 151 common fluids based on residual entropy scaling and cubic equations of state

Xiaoxian Yang*

Chemnitz University of Technology, Applied Thermodynamics, 09107 Chemnitz, Germany

This document explains the large amount of tables and figures in the 'supporting information.zip' published along with the manuscript.

1. Tables

1.1. Parameters for dilute gas viscosity calculation

Location: Supporting Information/Parameters/Dilute gas for viscosity.txt

Contains: parameters $a_{\eta,i}$ (i = 0,1,2,3,4) to be used in the Eq. (2) in the main manuscript, and the temperature ranges these parameters were fitted to the calculations of REFPROP 10.0.[1]

1.2. Parameters for dilute gas thermal conductivity calculation

Location: Supporting Information/Parameters/Dilute gas for thermal conductivity.txt

Contains: parameters $a_{\lambda,i}$ (i = 0,1,2,3,4) to be used in the Eq. (8) in the main manuscript, and the temperature ranges these parameters were fitted to the calculations of REFPROP 10.0.[1]

1.3. Fluid constants

Location: Supporting Information/Parameters/Fluid constants.txt

Contains:

(I) Molar mass M, critical point information (T_c, p_c, ρ_c), acentric factor ω needed in the cubic EoS calculation.

(II) k_0 and k_1 for the for ideal gas isobaric heat capacity calculation to be used in the Eq. (12) in the main manuscript.

(III) L-J parameters (the pair-potential energy ε/k_B and the collision diameter σ) used for an alternative method of dilute gas property calculation.

* Corresponding author. Xiaoxian Yang. Email address: xiaoxian.yang@mb.tu-chemnitz.de. ORCID ID: https://orcid.org/0000-0003-4655-3156.

(IV) Other properties that are not used in this work.

1.4. RES parameters for viscosity

Location: Supporting Information/Parameters/RES parameters for viscosity/xxx

Contains: RES parameters ($n_{\lambda,\mathrm{f}i}$, $n_{\lambda,\mathrm{g}i}$ and ξ_λ) of viscosity developed for each of the four cubic EoS (PR, SRK, PTV and YFR).

In each file: the number of experimental data used for parameter fitting (DataN), the group number of this fluid (GroupN), the group-specific parameters (n1_glb and so on), the fluid-specific parameters (n1_ind and so on) and fluid-specific scaling factor (xita) are given for each pure fluid.

1.5. RES parameters for thermal conductivity

Location: Supporting Information/Parameters/RES parameters for thermal conductivity/xxx

Contains: RES parameters ($n_{\lambda,\mathrm{f}i}$, $n_{\lambda,\mathrm{g}i}$ and ξ_λ) of thermal conductivity developed for each of the four cubic EoS (PR, SRK, PTV and YFR).

In each file: the number of experimental data used for parameter fitting (DataN), the group number of this fluid (GroupN), the group-specific parameters (n1_glb, and so on), the fluid-specific parameters (n1_ind and so on) and fluid-specific scaling factor (xita) are given for each pure fluid.

2. Figures for viscosity

2.1. Residual viscosity ln($\eta^{\mathrm{r}+}$+1) vs. residual entropy s^+/ξ_η

Location: Supporting Information/Fig. V1 - s_plus vs eta_plus - all data - YFR EoS/xxx

Contains: ln($\eta^{\mathrm{r}+}$+1) vs. s^+/ξ_η for each of the 124 pure fluids with all experimental data, except for those filtered out by Filter 1, plotted. The black solid curves and red dash curves are calculated with group-specific parameters and fluid-specific parameters respectively. To reduce the size of the supporting information, only plots with the YFR EoS were provided. Detailed citation information is provided in the SI of our previous work.[2]

2.2. The relative deviation of each viscosity experimental point to the RES model

Location: Supporting Information/Fig. V2 - relative deviation - analyzable data - YFR EoS/xx

Contains: Relative deviations of the analyzable experimental data from the RES model and the models in REFPROP 10.0 for each of the 124 pure fluids. To reduce the size of the supporting information, only plots with the YFR EoS were provided. Detailed citation information is provided in the SI of our previous work.[2]

2.3 Detailed comparison among different viscosity models

Location: Supporting Information/ Fig. V3 - Model comparison - 124 fluids in 8 groups/xxx

Contains: The AARD and ARD from the analyzable experimental data to the six viscosity models for each pure fluid and each group.

2.4 Relative deviation of viscosity data vs other properties

Location: Supporting Information/Fig. V4 - relative deviation vs other properties/xxx

Contains: Relative deviation from experimental data, those filtered out by Filters 1 and 2, to model prediction (YFR EoS + RES as an example) as a function of density, pressure, temperature and residual entropy.

3. Figures for thermal conductivity

3.1. Residual thermal conductivity λ^{r+} vs. residual entropy s^{+}/ξ_{η}

Location: Supporting Information/ Fig. TC1 - s_plus vs lambda_plus - all data - YFR EoS /xxx

Contains: λ^{r+} vs. s^{+}/ξ_{λ} for each of the 125 pure fluids with all experimental data, except for those filtered out by Filter 1, plotted. The black solid curves and red dashed curves are calculated with group-specific parameters, and fluid-specific parameters respectively. To reduce the size of the supporting information, only plots with YFR EoS were provided. Detailed citation information is provided in the SI of our previous work.[3]

3.2. Relative deviation of each thermal conductivity experimental point to the RES model

Location: Supporting Information/Fig. TC2 - relative deviation - analyzable data - YFR EoS/x

Contains: Relative deviations of the analyzable experimental data from the RES model and the models in REFPROP 10.0 for each of the 124 pure fluids. To reduce the size of the supporting information, only plots with YFR EoS were provided. Detailed citation information is provided in the SI of our previous work.[3]

3.3 Detailed comparison among different thermal conductivity models

Location: Supporting Information/Fig. TC3 - Model comparison - 125 fluids in 8 groups/xxx

Contains: The AARD and ARD from the analyzable experimental data to the six thermal conductivity models for each pure fluid and each group.

Reference

(1) Lemmon, E. W.; Bell, I. H.; Huber, M. L.; McLinden, M. O. *NIST Standard Reference Database 23: Reference Fluid Thermodynamic and Transport Properties-REFPROP, Version 10.0, National Institute of Standards and Technology*; 2018. https://doi.org/10.18434/T4/1502528.

(2) Yang, X.; Xiao, X.; Thol, M.; Richter, M.; Bell, I. H. Linking Viscosity to Equations of State Using Residual Entropy Scaling Theory. *Int. J. Thermophys.* **2022**, *43* (12), 183. https://doi.org/10.1007/s10765-022-03096-9.

(3) Li, Z.; Duan, Y.; Yang, X. Linking Thermal Conductivity to Equations of State Using the Residual Entropy Scaling Theory. *Ind. Eng. Chem. Res.* **2024**, *63* (42), 18160–18175. https://doi.org/10.1021/acs.iecr.4c02946.
