# Supplementary figures and images for: Viscosity and Thermal Conductivity Models of 151 Common Fluids Based on Residual Entropy Scaling and Cubic Equations of State (part 2 of 2)
Source: ACS Omega. 2025 Feb 8;10(6):6124–34. doi: 10.1021/acsomega.4c10815 (PMC11840589; doi:10.1021/acsomega.4c10815)

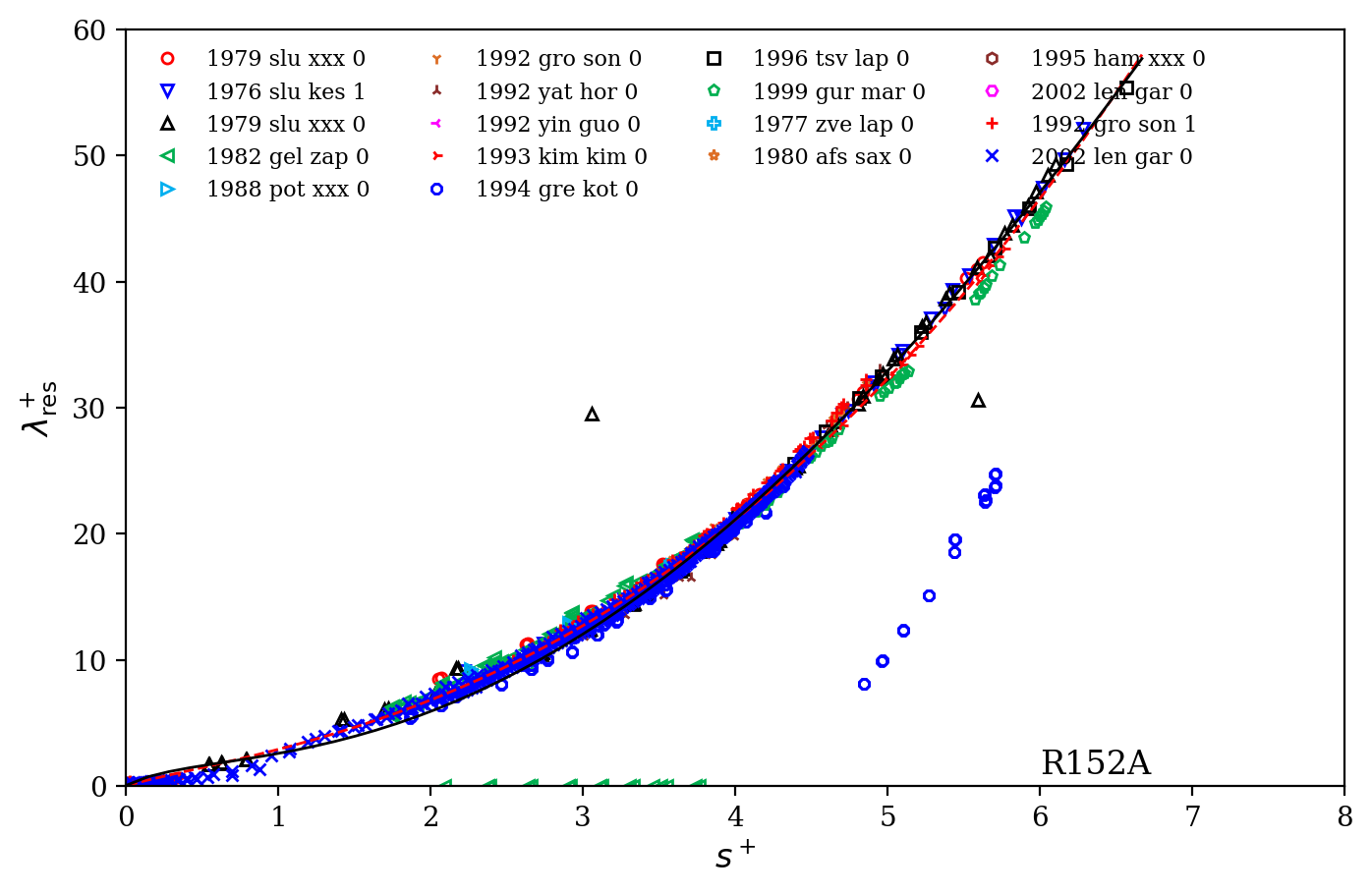

Supplement: Supplementary file 2 — ao4c10815_si_002.zip [file ao4c10815_si_002.zip › Supporting Information/Fig. TC1 - s_plus vs lambda_plus - all data - YFR EoS/R152A.png]

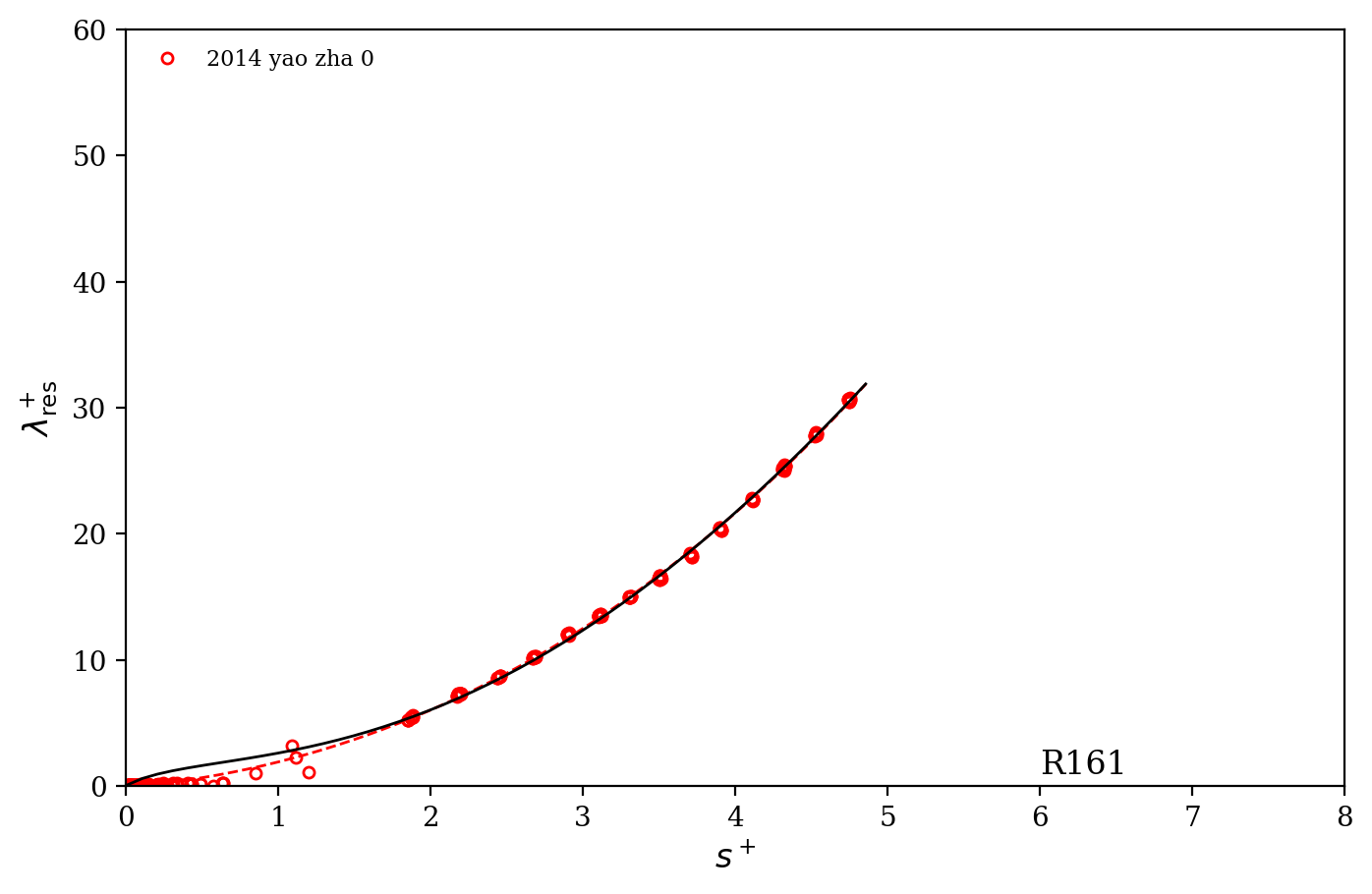

Supplement: Supplementary file 2 — ao4c10815_si_002.zip [file ao4c10815_si_002.zip › Supporting Information/Fig. TC1 - s_plus vs lambda_plus - all data - YFR EoS/R161.png]

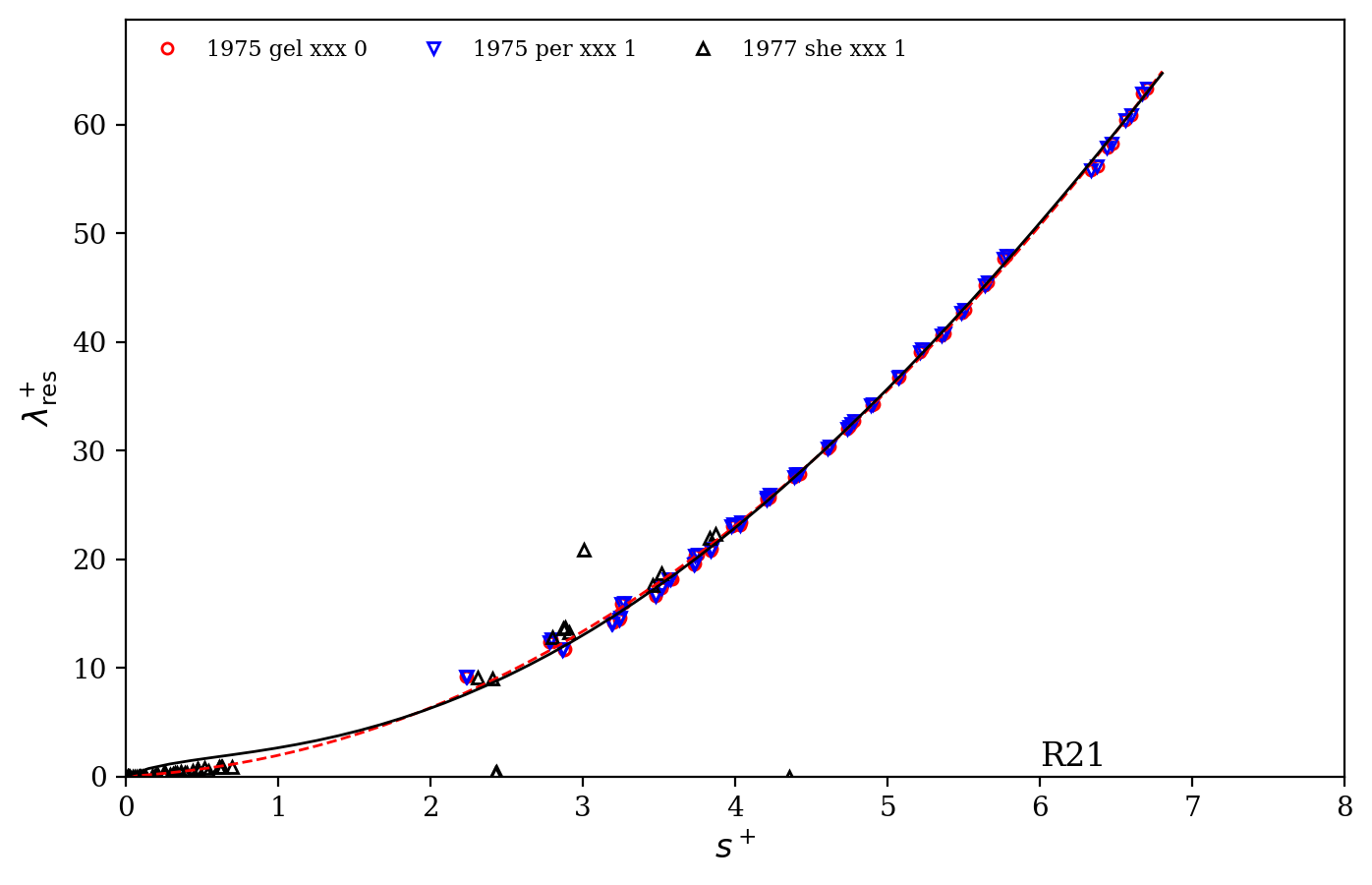

Supplement: Supplementary file 2 — ao4c10815_si_002.zip [file ao4c10815_si_002.zip › Supporting Information/Fig. TC1 - s_plus vs lambda_plus - all data - YFR EoS/R21.png]

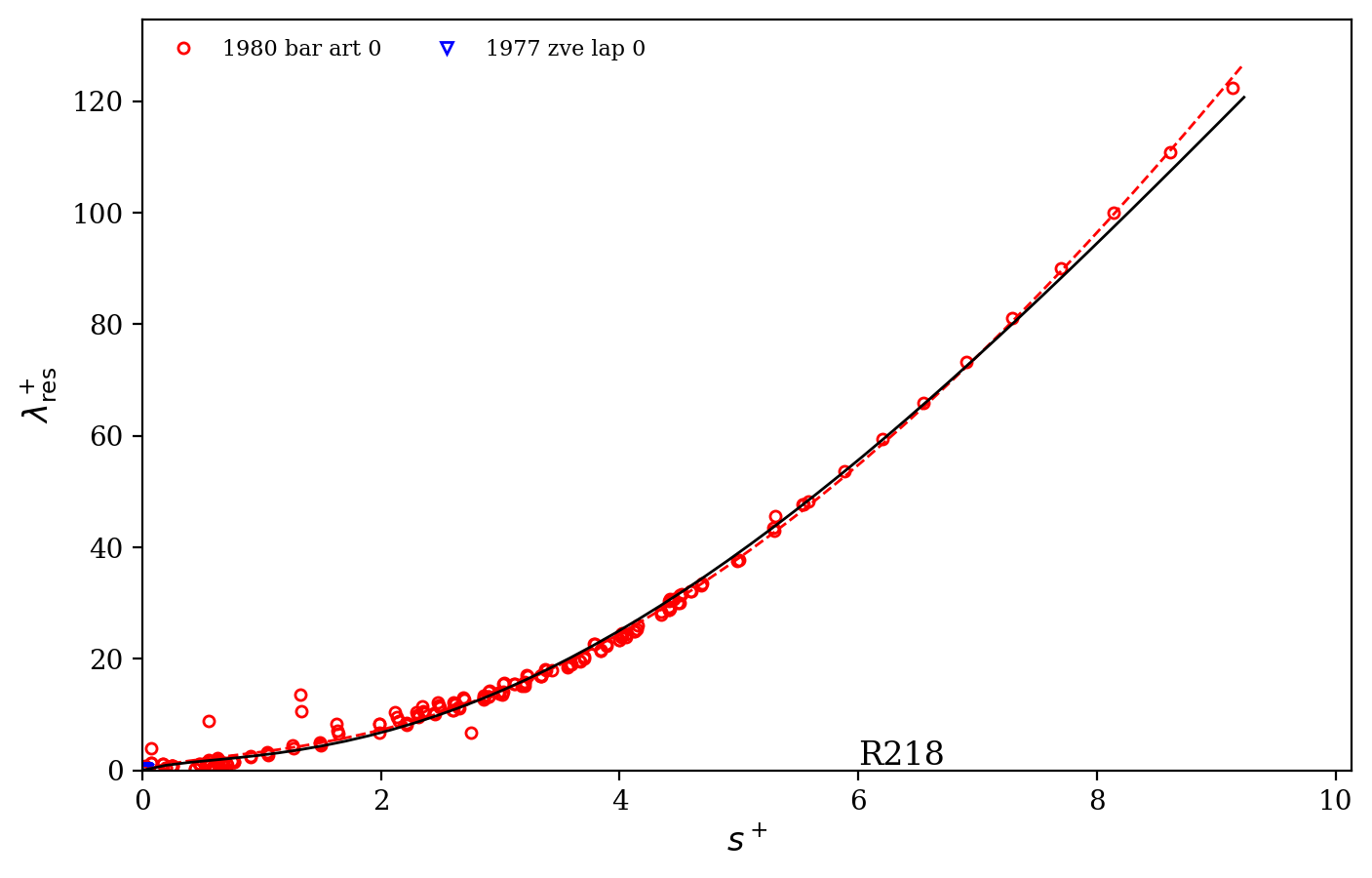

Supplement: Supplementary file 2 — ao4c10815_si_002.zip [file ao4c10815_si_002.zip › Supporting Information/Fig. TC1 - s_plus vs lambda_plus - all data - YFR EoS/R218.png]

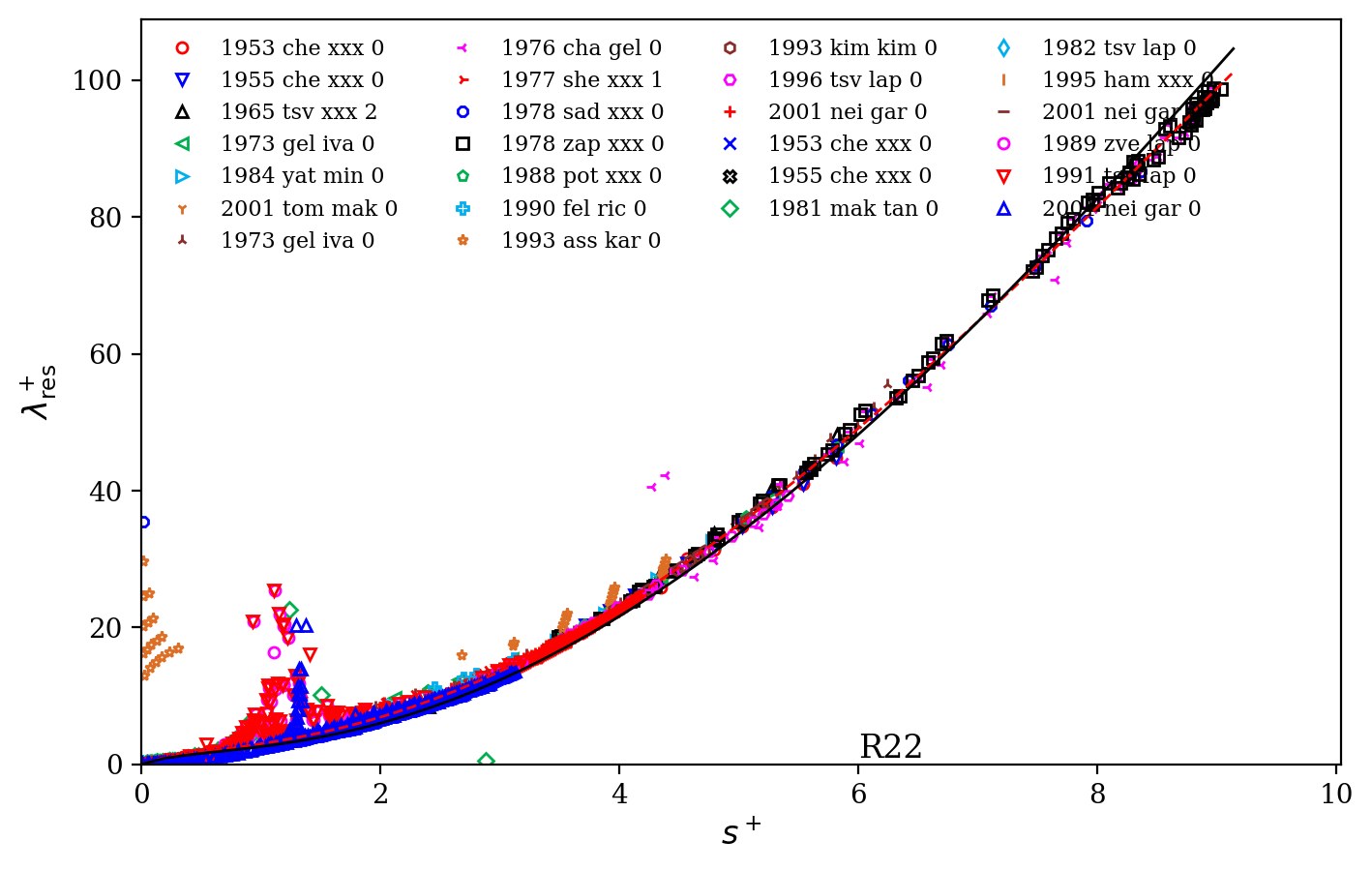

Supplement: Supplementary file 2 — ao4c10815_si_002.zip [file ao4c10815_si_002.zip › Supporting Information/Fig. TC1 - s_plus vs lambda_plus - all data - YFR EoS/R22.png]

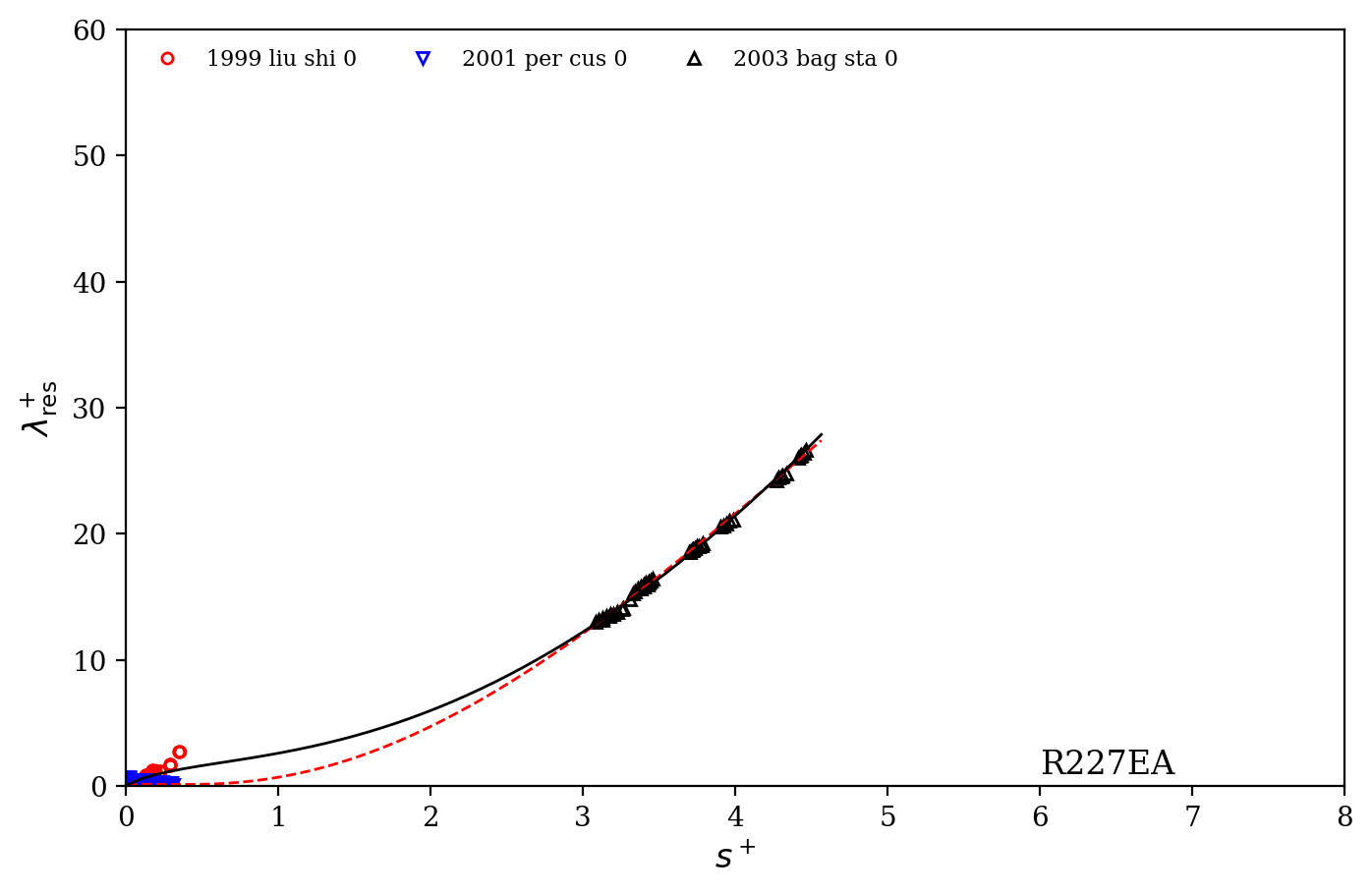

Supplement: Supplementary file 2 — ao4c10815_si_002.zip [file ao4c10815_si_002.zip › Supporting Information/Fig. TC1 - s_plus vs lambda_plus - all data - YFR EoS/R227EA.png]

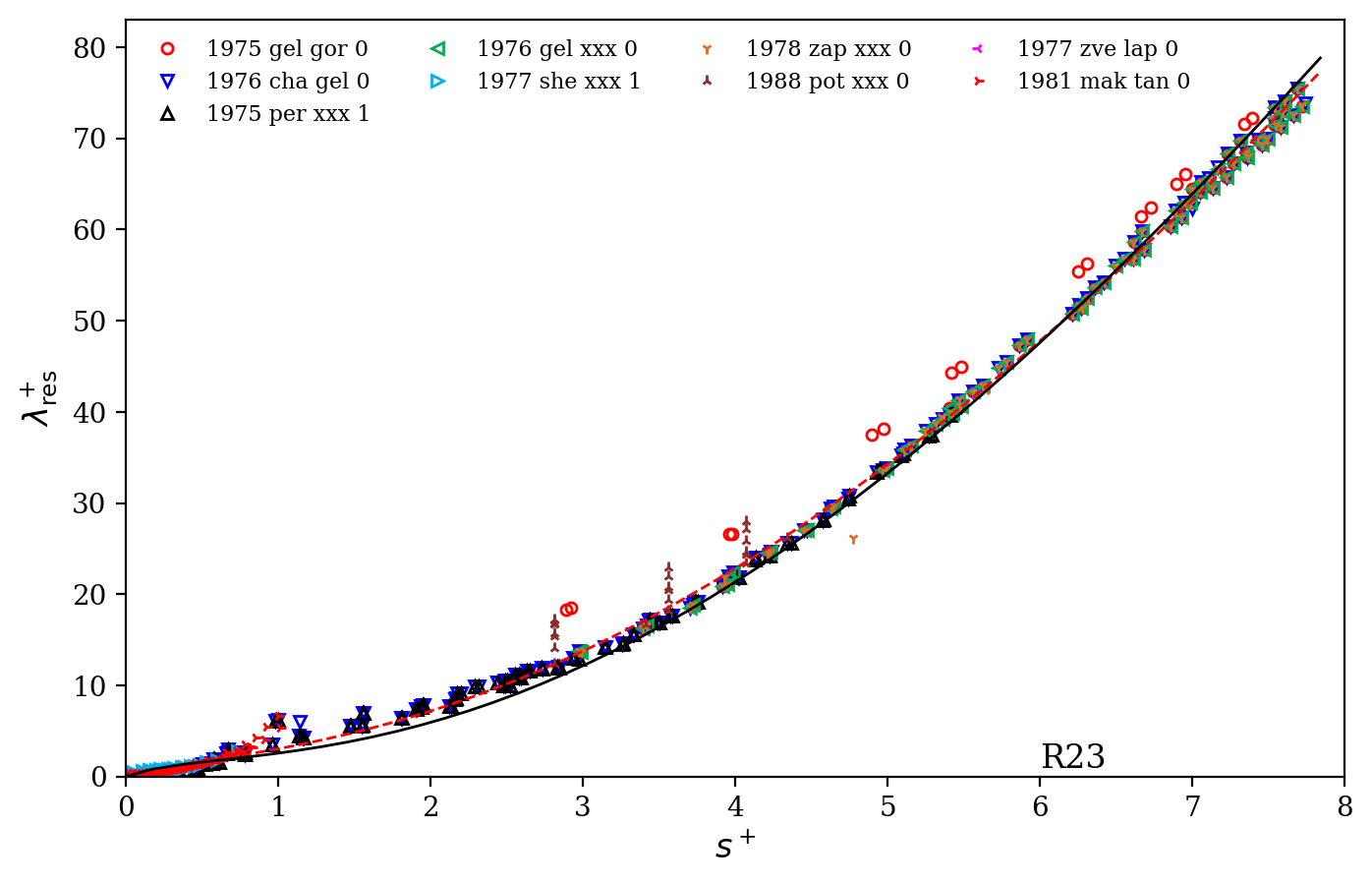

Supplement: Supplementary file 2 — ao4c10815_si_002.zip [file ao4c10815_si_002.zip › Supporting Information/Fig. TC1 - s_plus vs lambda_plus - all data - YFR EoS/R23.png]

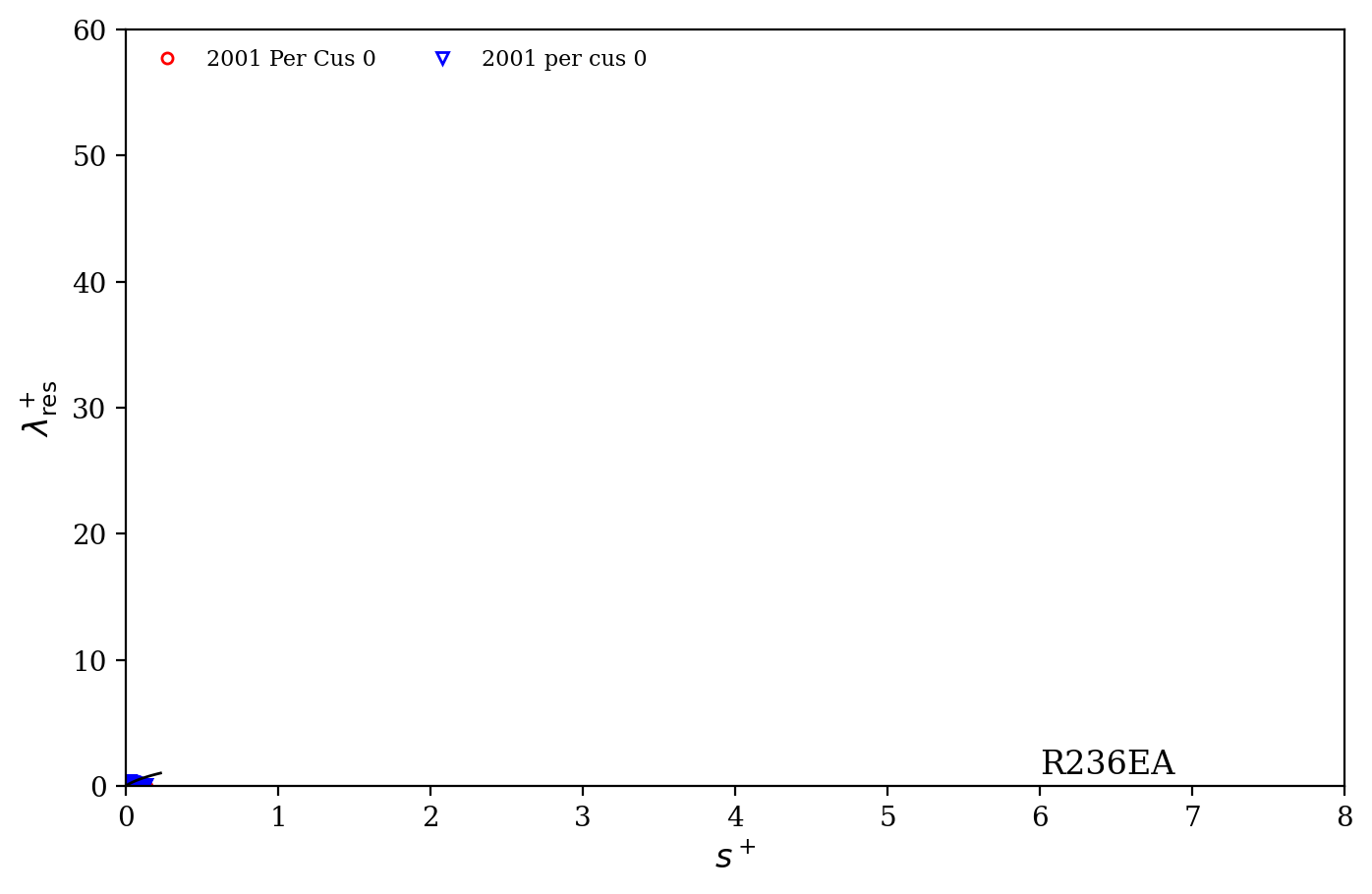

Supplement: Supplementary file 2 — ao4c10815_si_002.zip [file ao4c10815_si_002.zip › Supporting Information/Fig. TC1 - s_plus vs lambda_plus - all data - YFR EoS/R236EA.png]

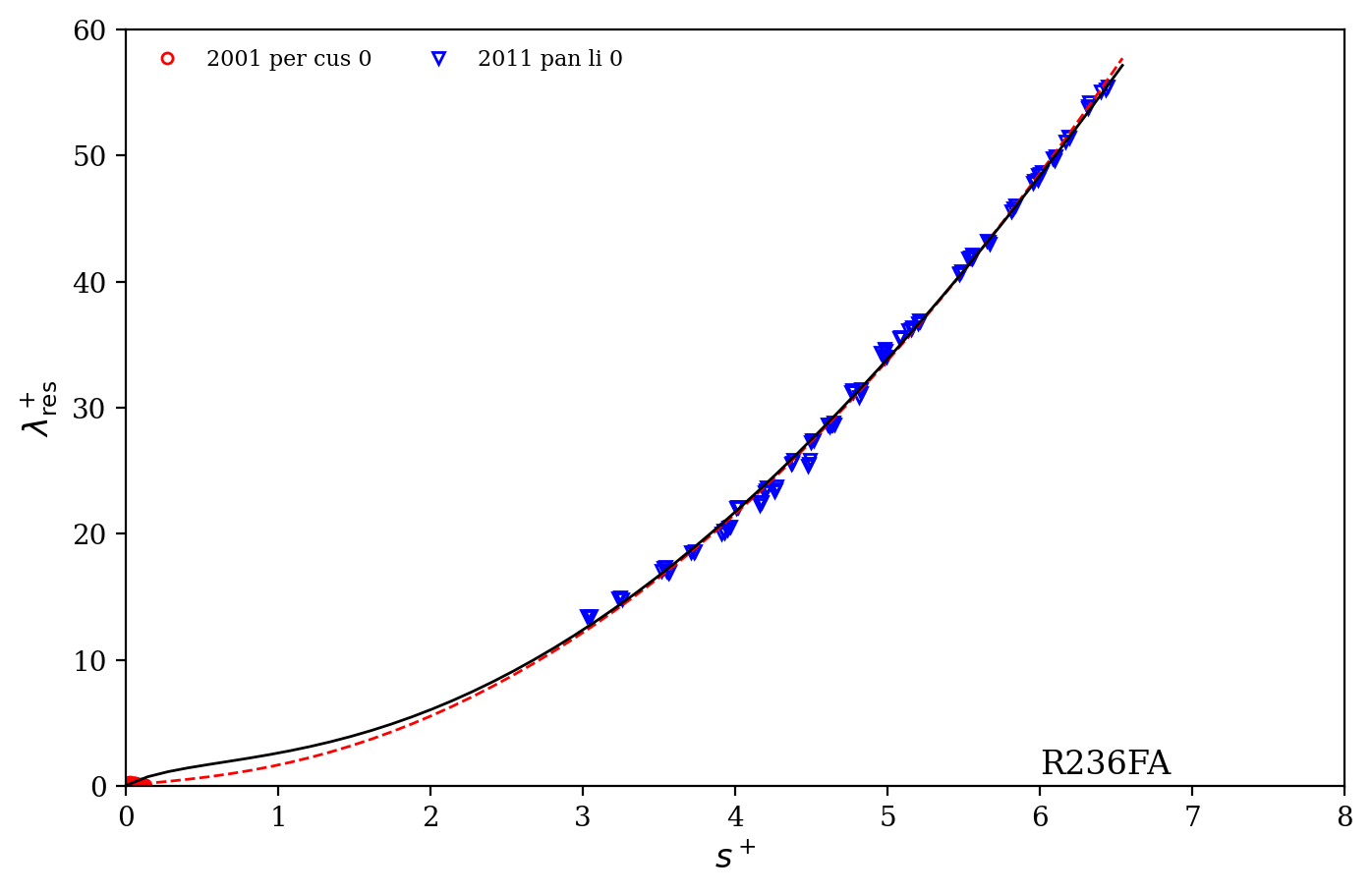

Supplement: Supplementary file 2 — ao4c10815_si_002.zip [file ao4c10815_si_002.zip › Supporting Information/Fig. TC1 - s_plus vs lambda_plus - all data - YFR EoS/R236FA.png]

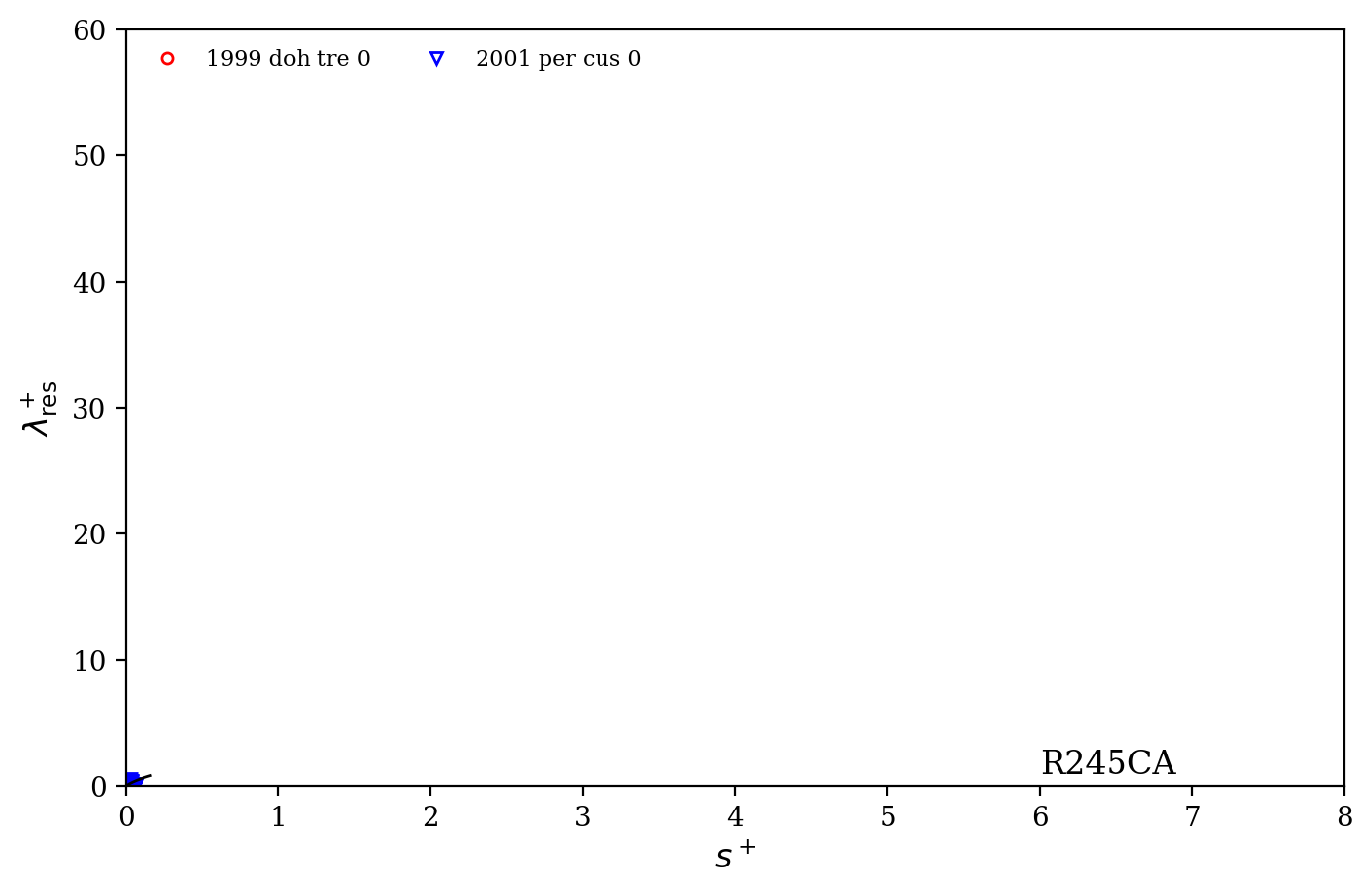

Supplement: Supplementary file 2 — ao4c10815_si_002.zip [file ao4c10815_si_002.zip › Supporting Information/Fig. TC1 - s_plus vs lambda_plus - all data - YFR EoS/R245CA.png]

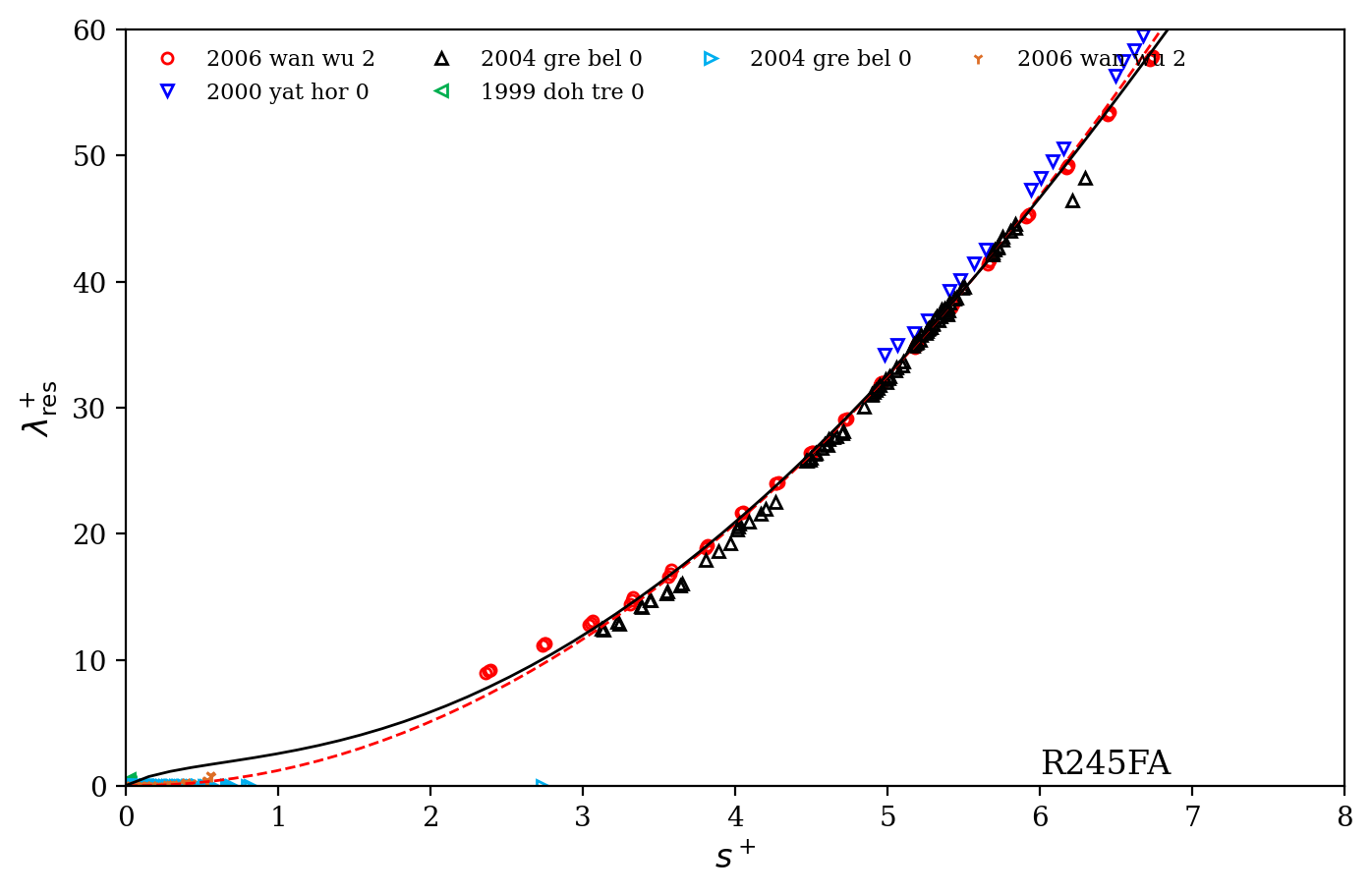

Supplement: Supplementary file 2 — ao4c10815_si_002.zip [file ao4c10815_si_002.zip › Supporting Information/Fig. TC1 - s_plus vs lambda_plus - all data - YFR EoS/R245FA.png]

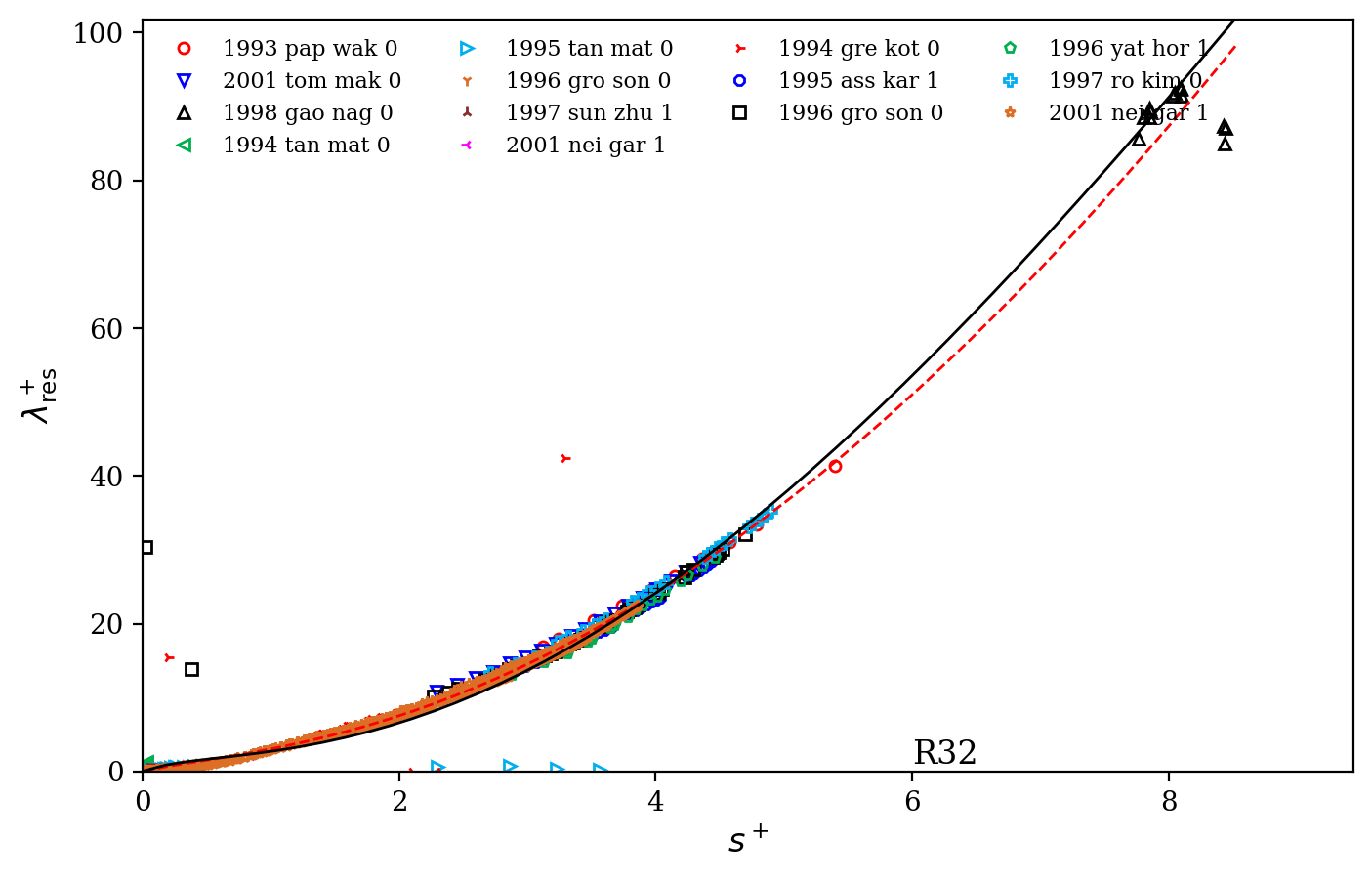

Supplement: Supplementary file 2 — ao4c10815_si_002.zip [file ao4c10815_si_002.zip › Supporting Information/Fig. TC1 - s_plus vs lambda_plus - all data - YFR EoS/R32.png]

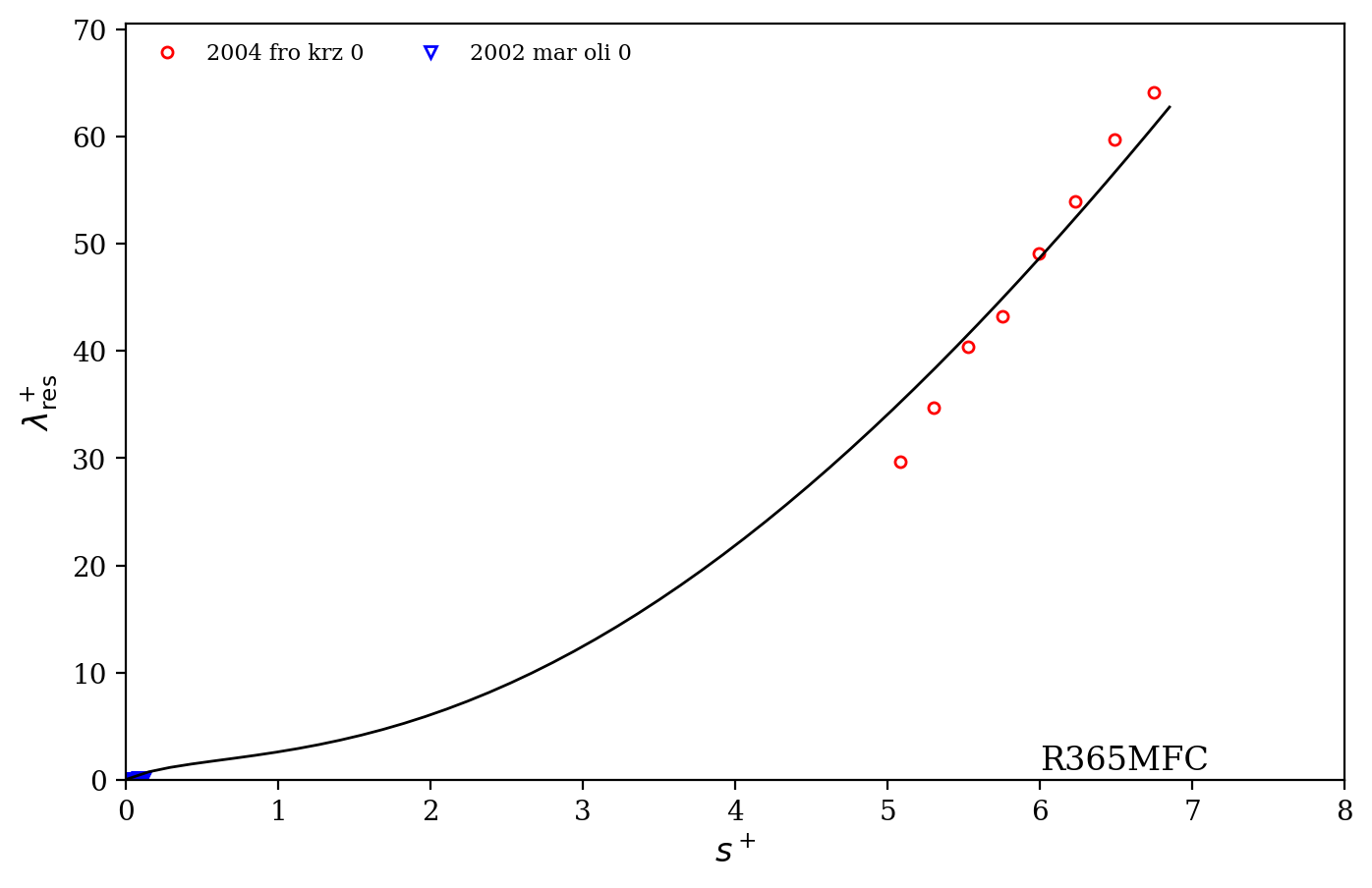

Supplement: Supplementary file 2 — ao4c10815_si_002.zip [file ao4c10815_si_002.zip › Supporting Information/Fig. TC1 - s_plus vs lambda_plus - all data - YFR EoS/R365MFC.png]

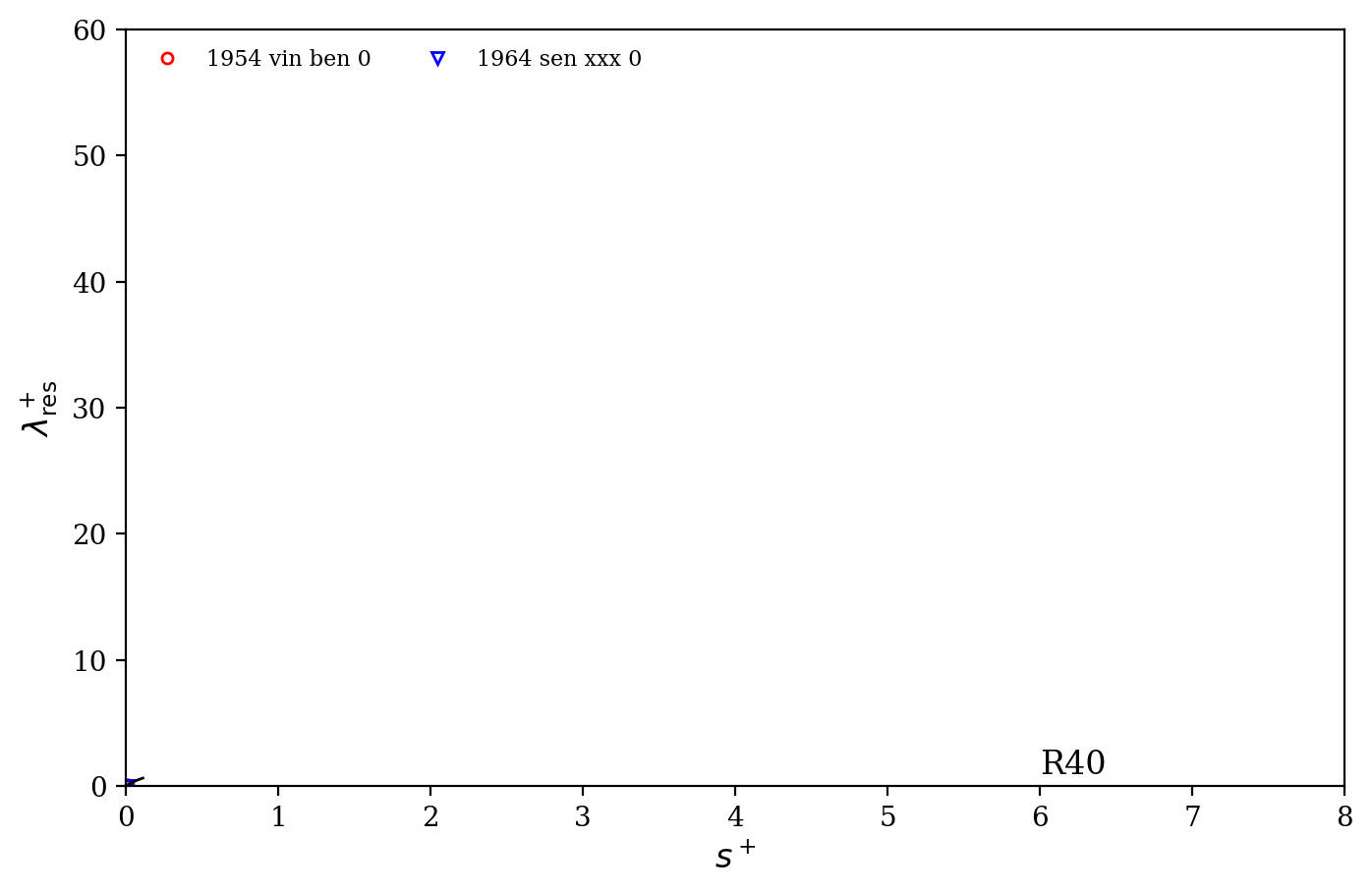

Supplement: Supplementary file 2 — ao4c10815_si_002.zip [file ao4c10815_si_002.zip › Supporting Information/Fig. TC1 - s_plus vs lambda_plus - all data - YFR EoS/R40.png]

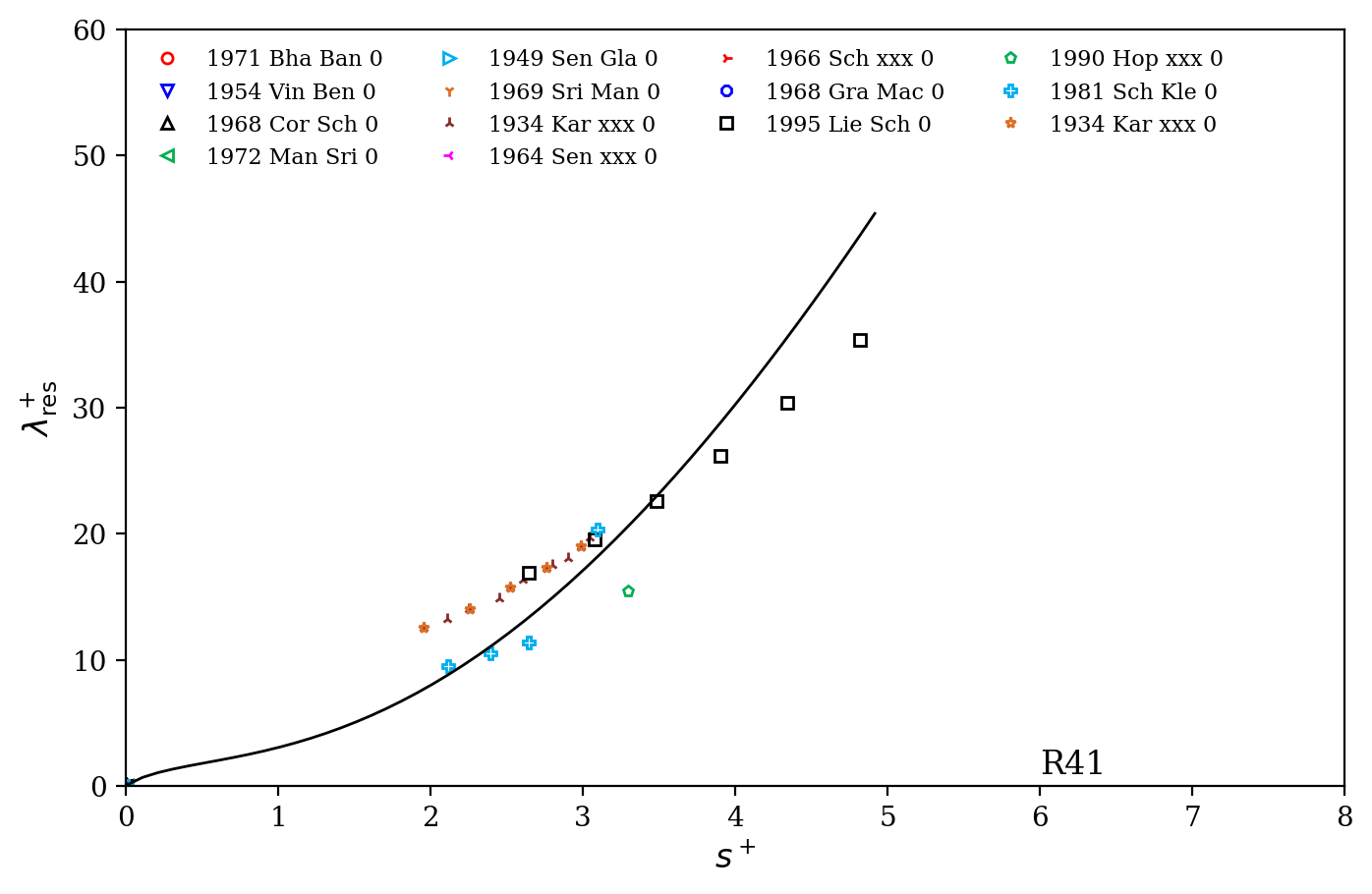

Supplement: Supplementary file 2 — ao4c10815_si_002.zip [file ao4c10815_si_002.zip › Supporting Information/Fig. TC1 - s_plus vs lambda_plus - all data - YFR EoS/R41.png]

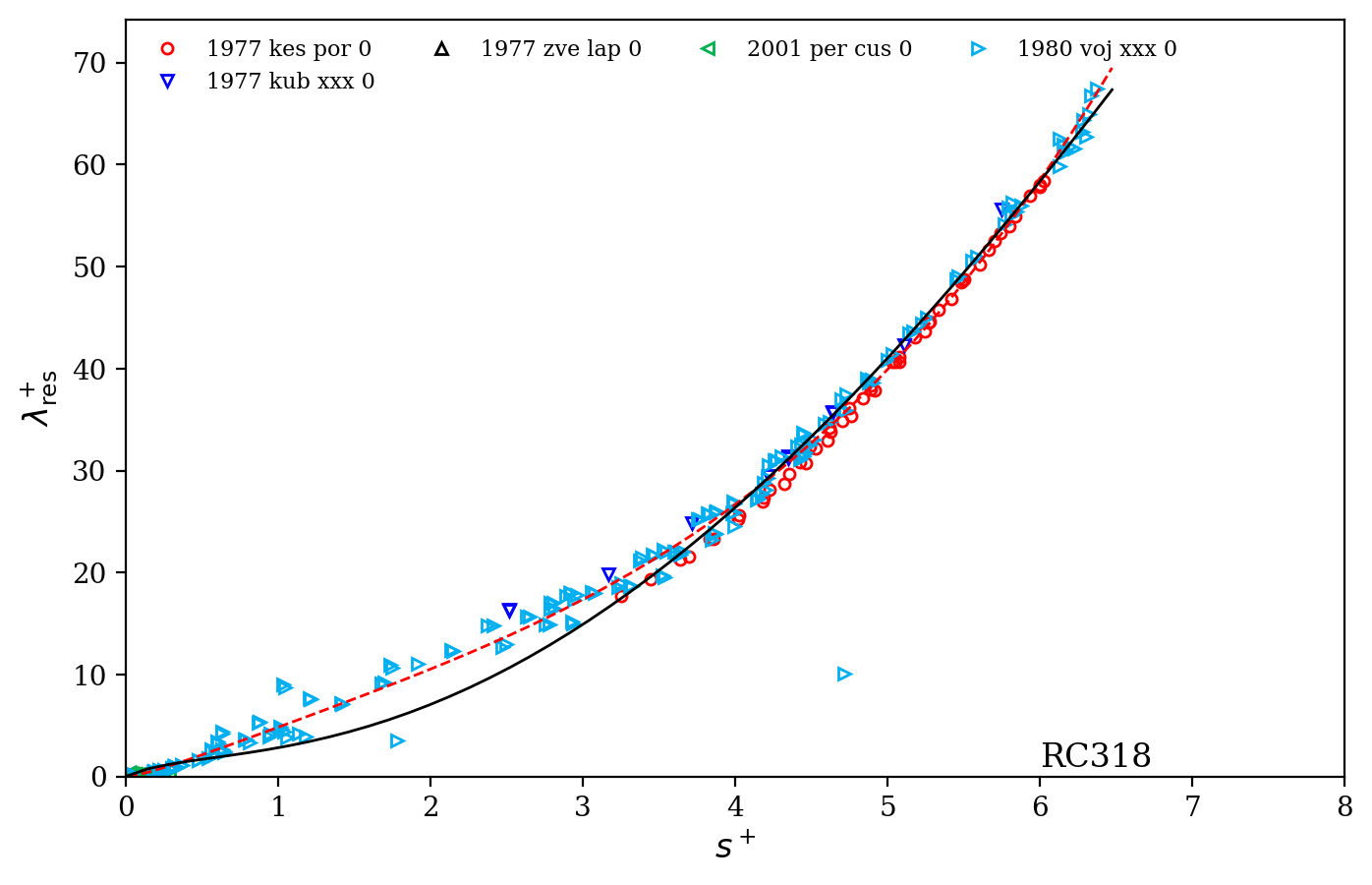

Supplement: Supplementary file 2 — ao4c10815_si_002.zip [file ao4c10815_si_002.zip › Supporting Information/Fig. TC1 - s_plus vs lambda_plus - all data - YFR EoS/RC318.png]

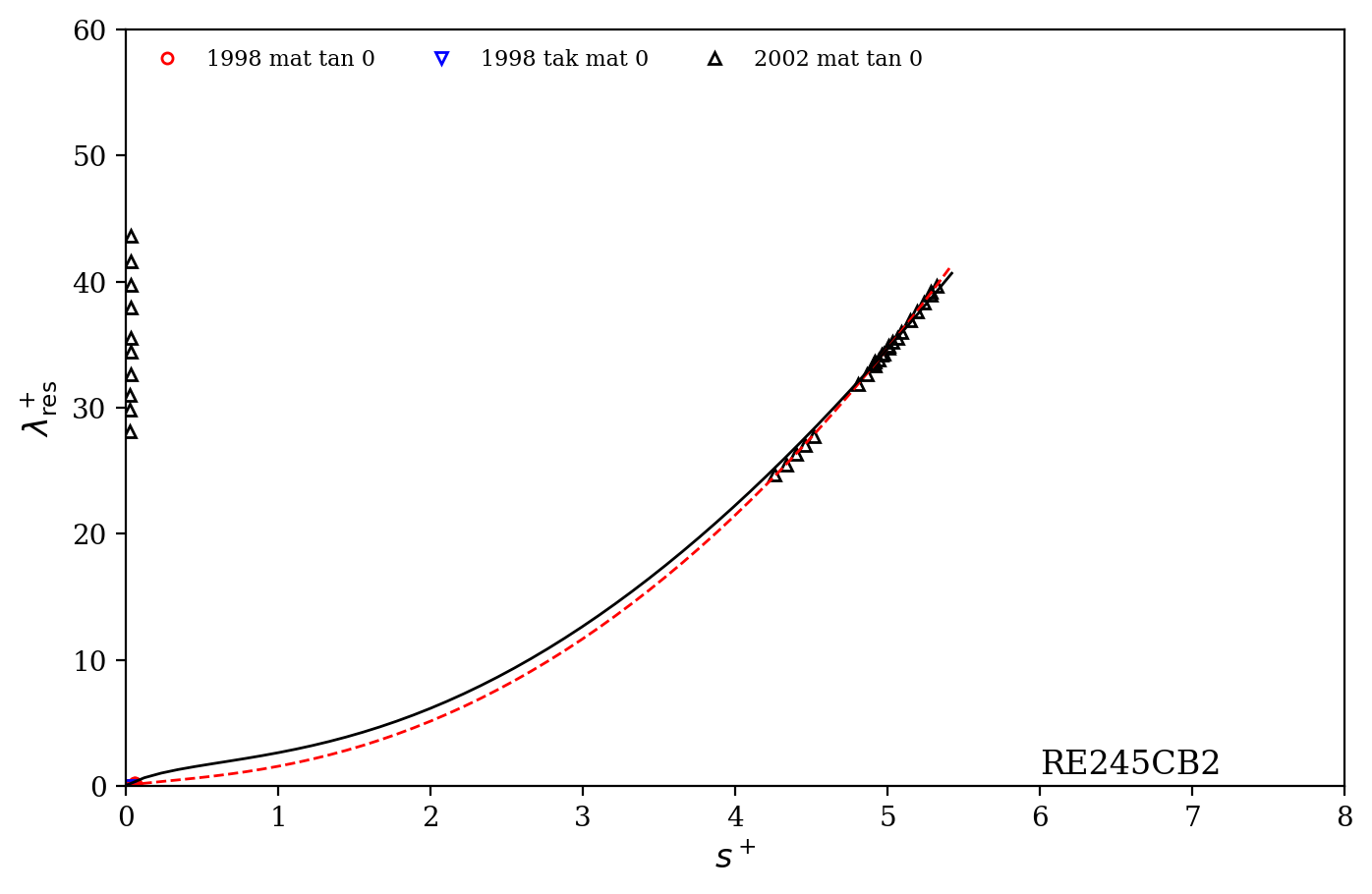

Supplement: Supplementary file 2 — ao4c10815_si_002.zip [file ao4c10815_si_002.zip › Supporting Information/Fig. TC1 - s_plus vs lambda_plus - all data - YFR EoS/RE245CB2.png]

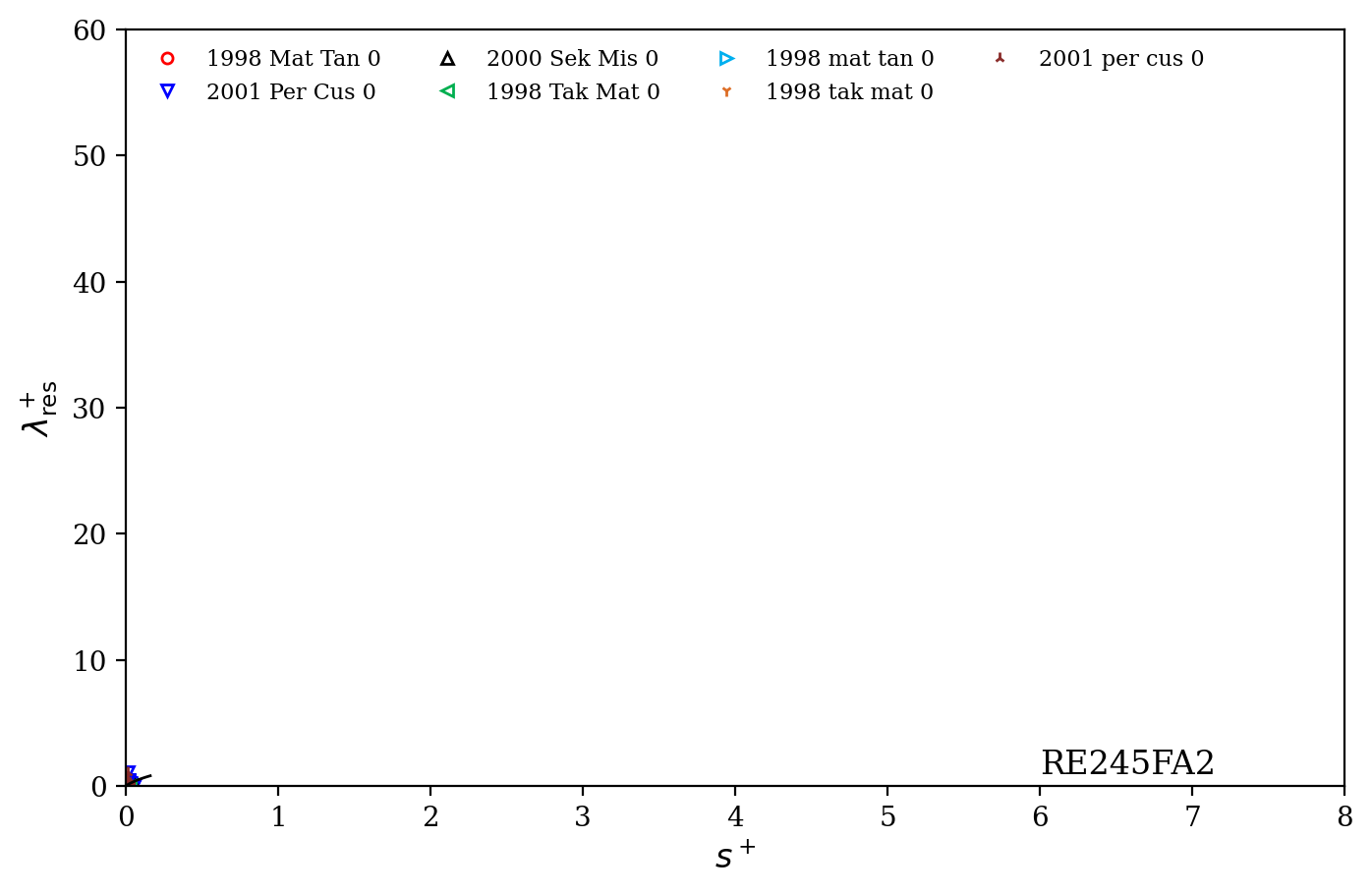

Supplement: Supplementary file 2 — ao4c10815_si_002.zip [file ao4c10815_si_002.zip › Supporting Information/Fig. TC1 - s_plus vs lambda_plus - all data - YFR EoS/RE245FA2.png]

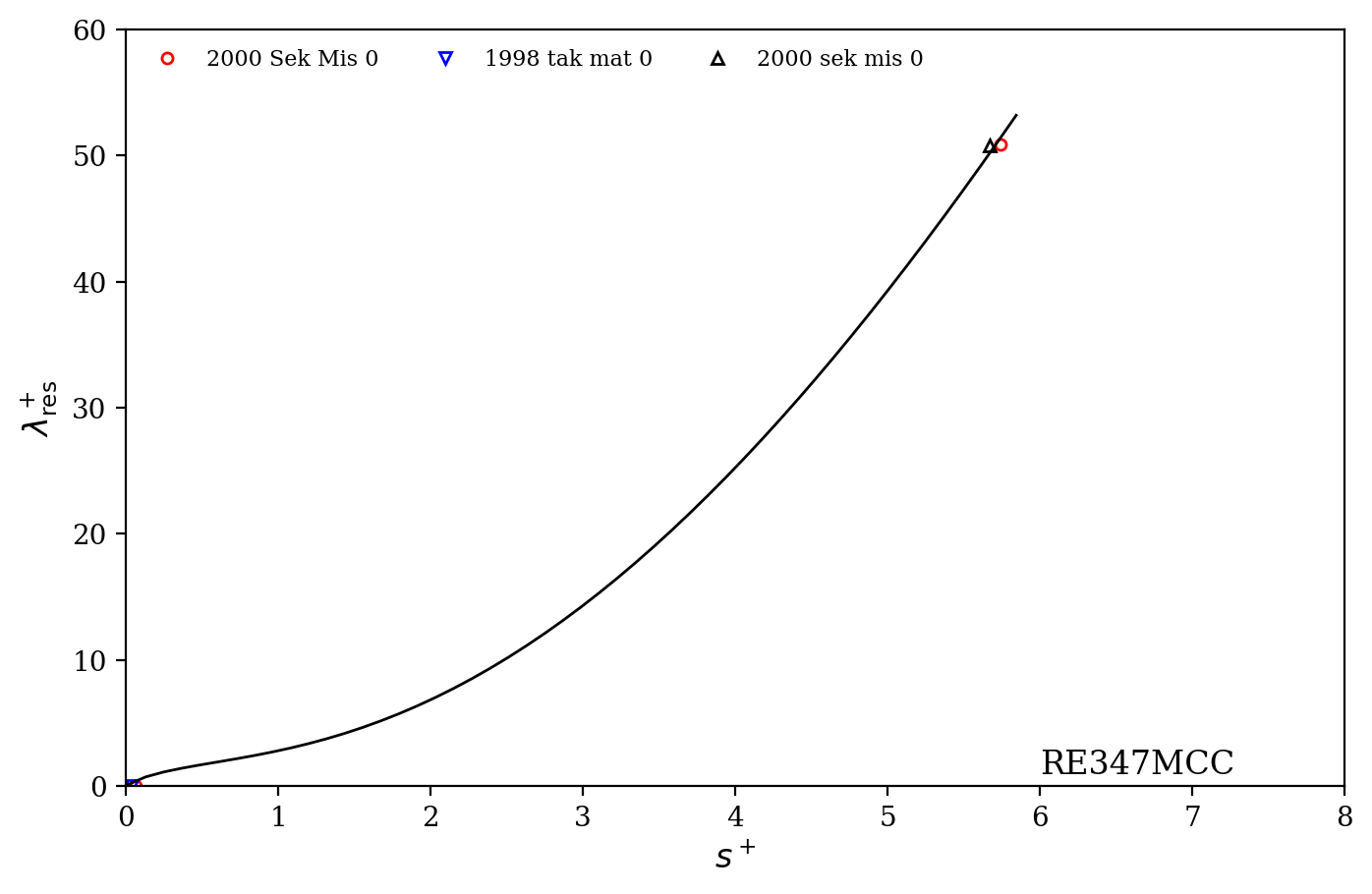

Supplement: Supplementary file 2 — ao4c10815_si_002.zip [file ao4c10815_si_002.zip › Supporting Information/Fig. TC1 - s_plus vs lambda_plus - all data - YFR EoS/RE347MCC.png]

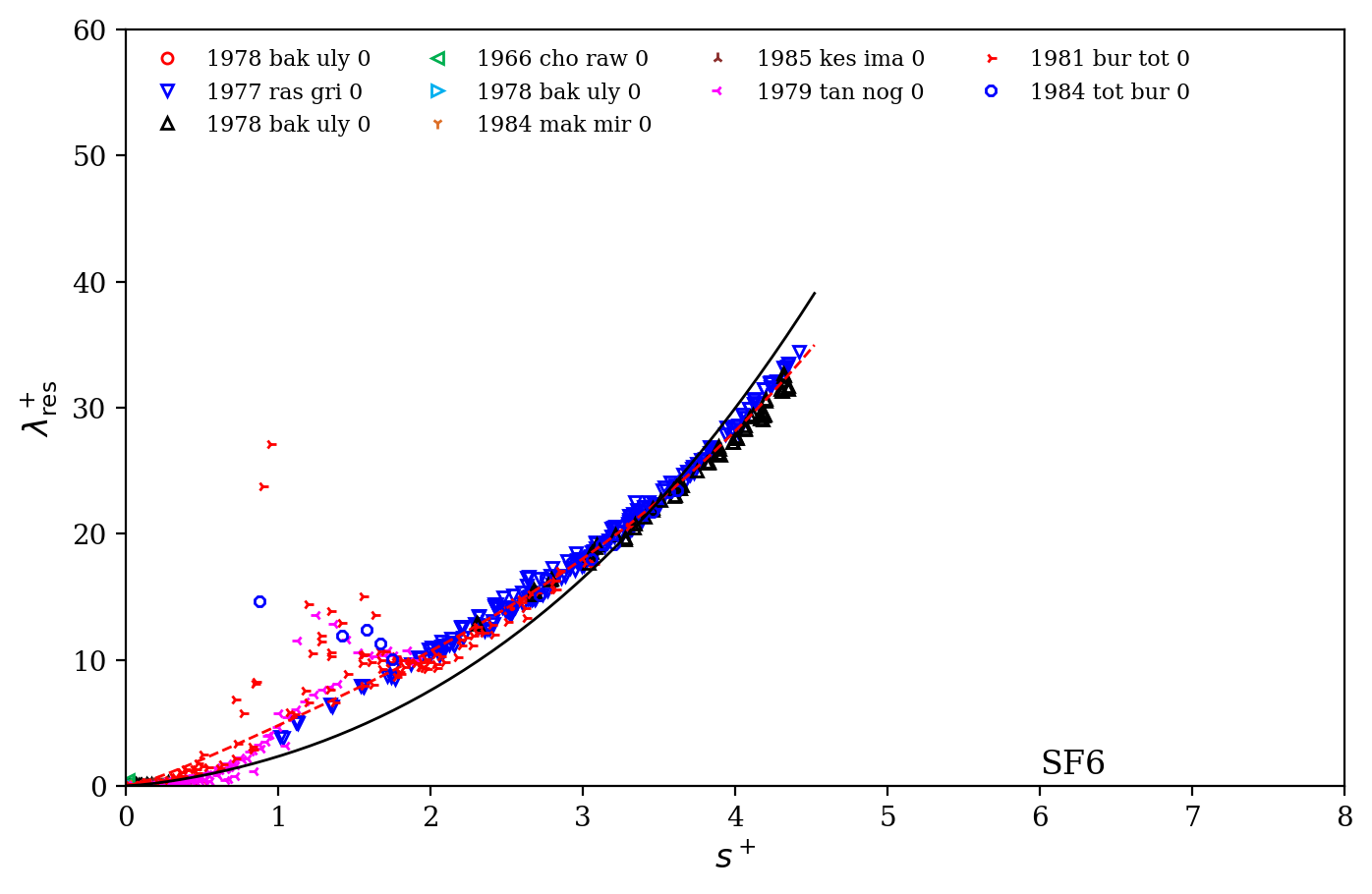

Supplement: Supplementary file 2 — ao4c10815_si_002.zip [file ao4c10815_si_002.zip › Supporting Information/Fig. TC1 - s_plus vs lambda_plus - all data - YFR EoS/SF6.png]

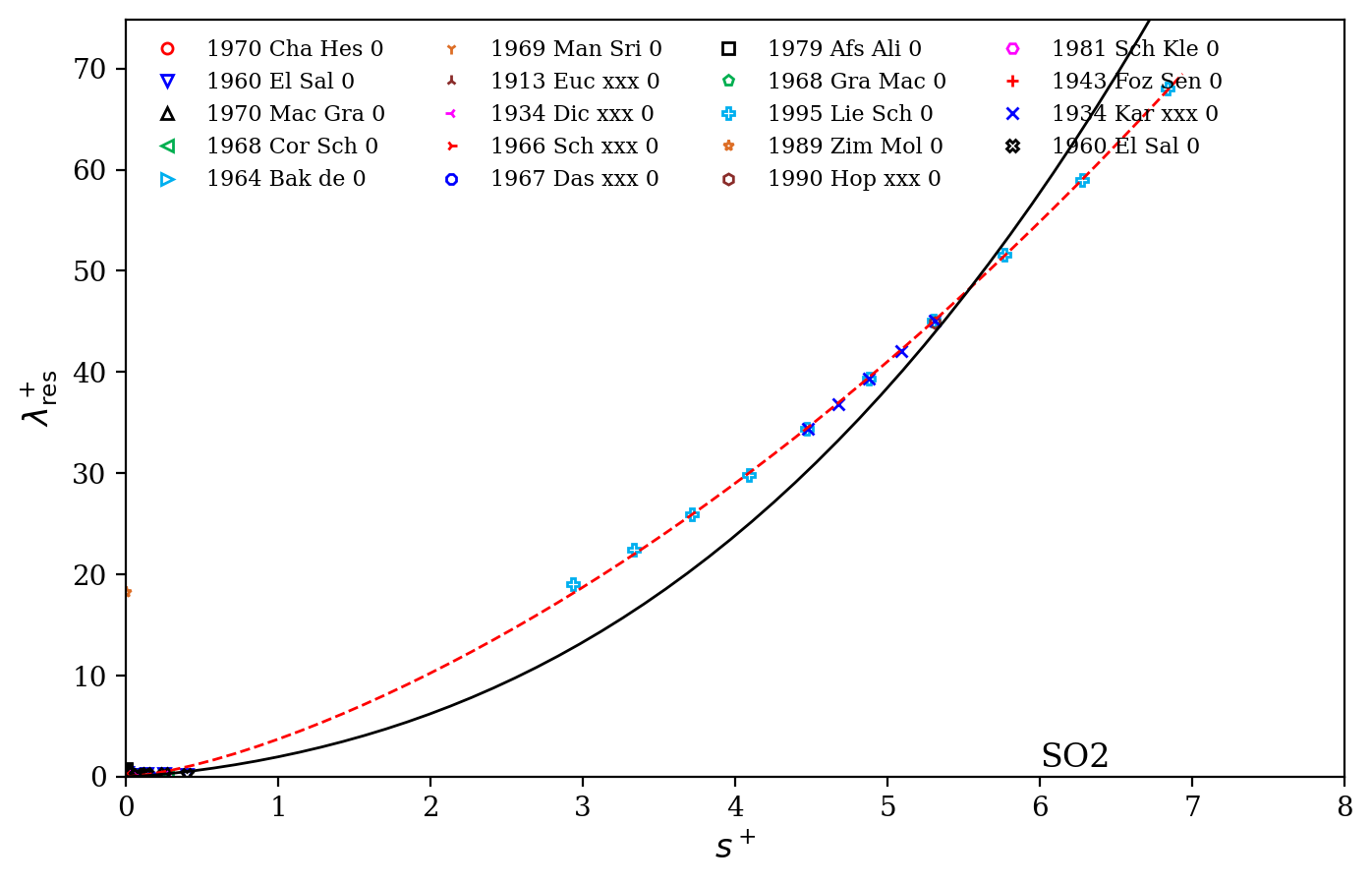

Supplement: Supplementary file 2 — ao4c10815_si_002.zip [file ao4c10815_si_002.zip › Supporting Information/Fig. TC1 - s_plus vs lambda_plus - all data - YFR EoS/SO2.png]

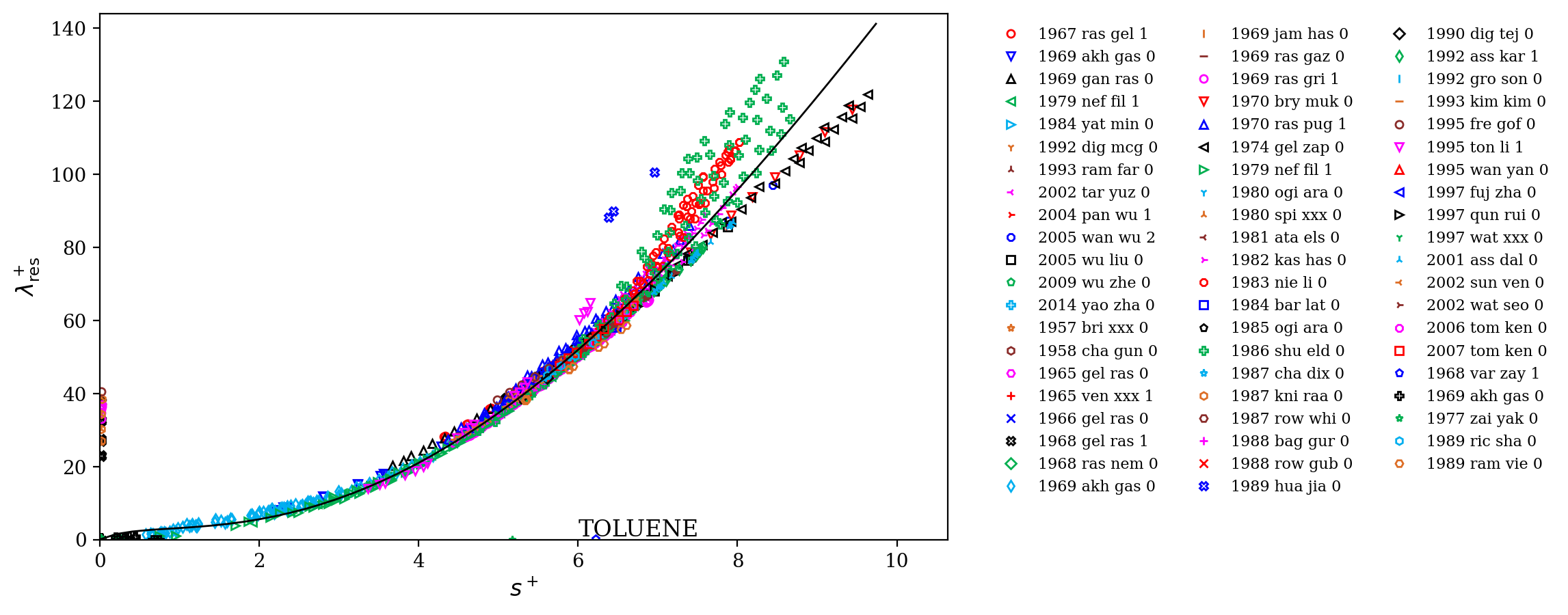

Supplement: Supplementary file 2 — ao4c10815_si_002.zip [file ao4c10815_si_002.zip › Supporting Information/Fig. TC1 - s_plus vs lambda_plus - all data - YFR EoS/TOLUENE.png]

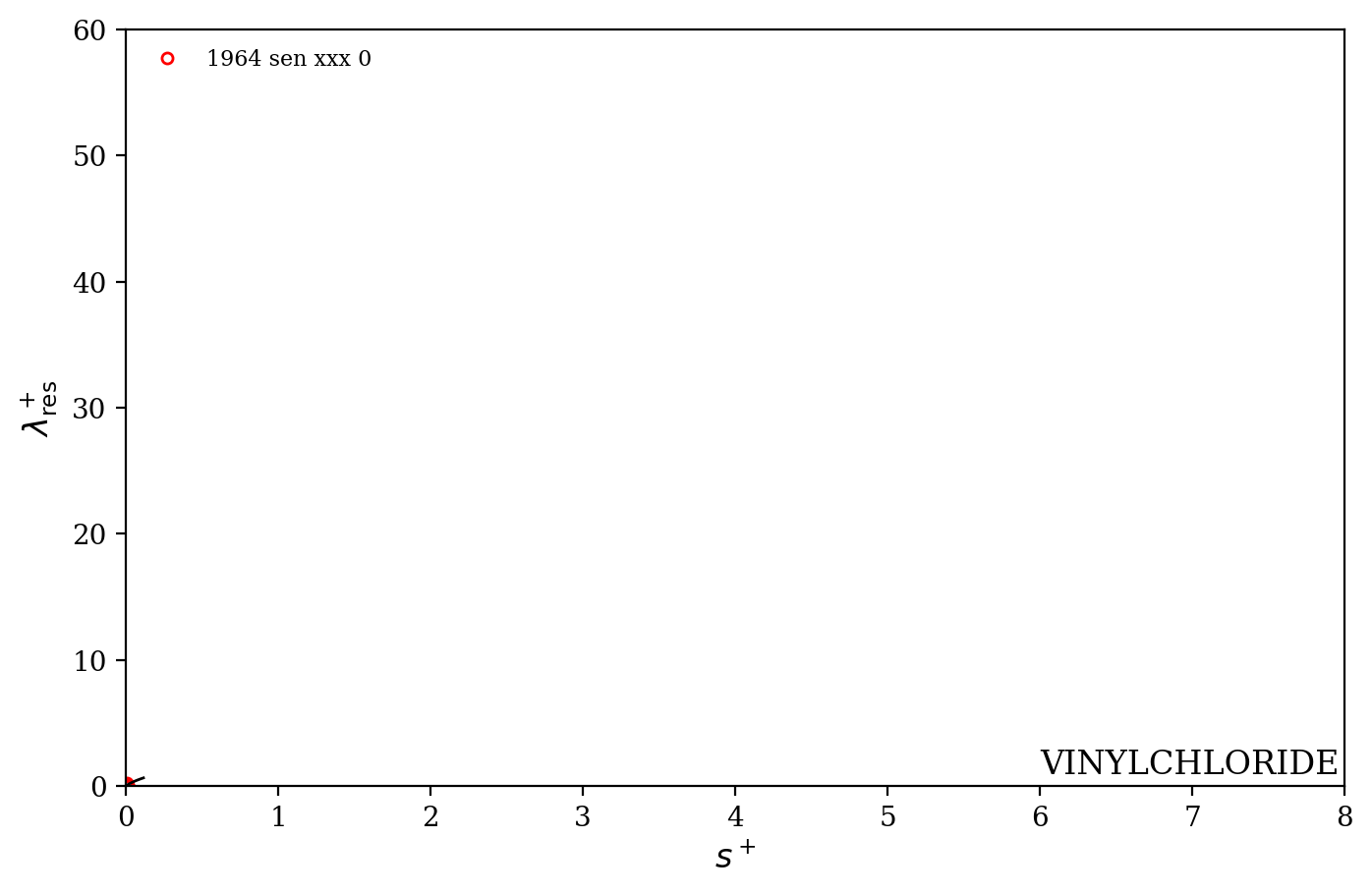

Supplement: Supplementary file 2 — ao4c10815_si_002.zip [file ao4c10815_si_002.zip › Supporting Information/Fig. TC1 - s_plus vs lambda_plus - all data - YFR EoS/VINYLCHLORIDE.png]

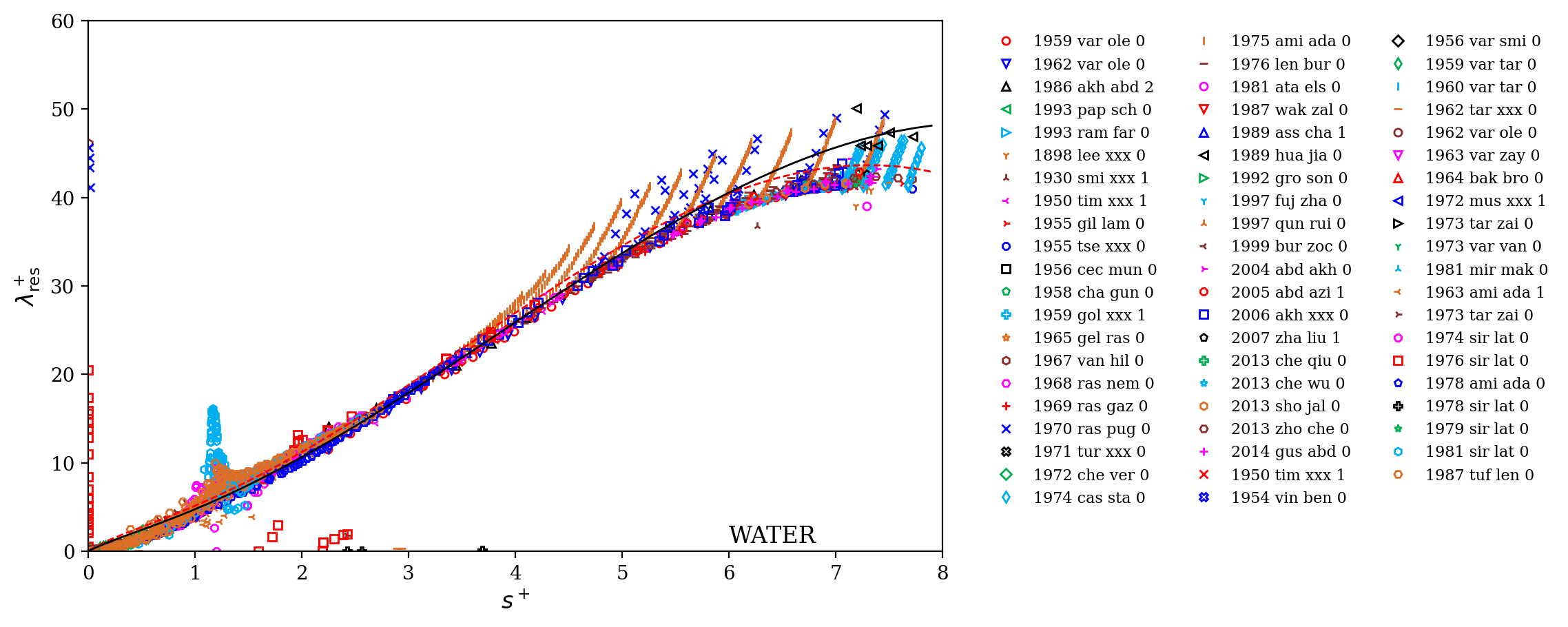

Supplement: Supplementary file 2 — ao4c10815_si_002.zip [file ao4c10815_si_002.zip › Supporting Information/Fig. TC1 - s_plus vs lambda_plus - all data - YFR EoS/WATER.png]

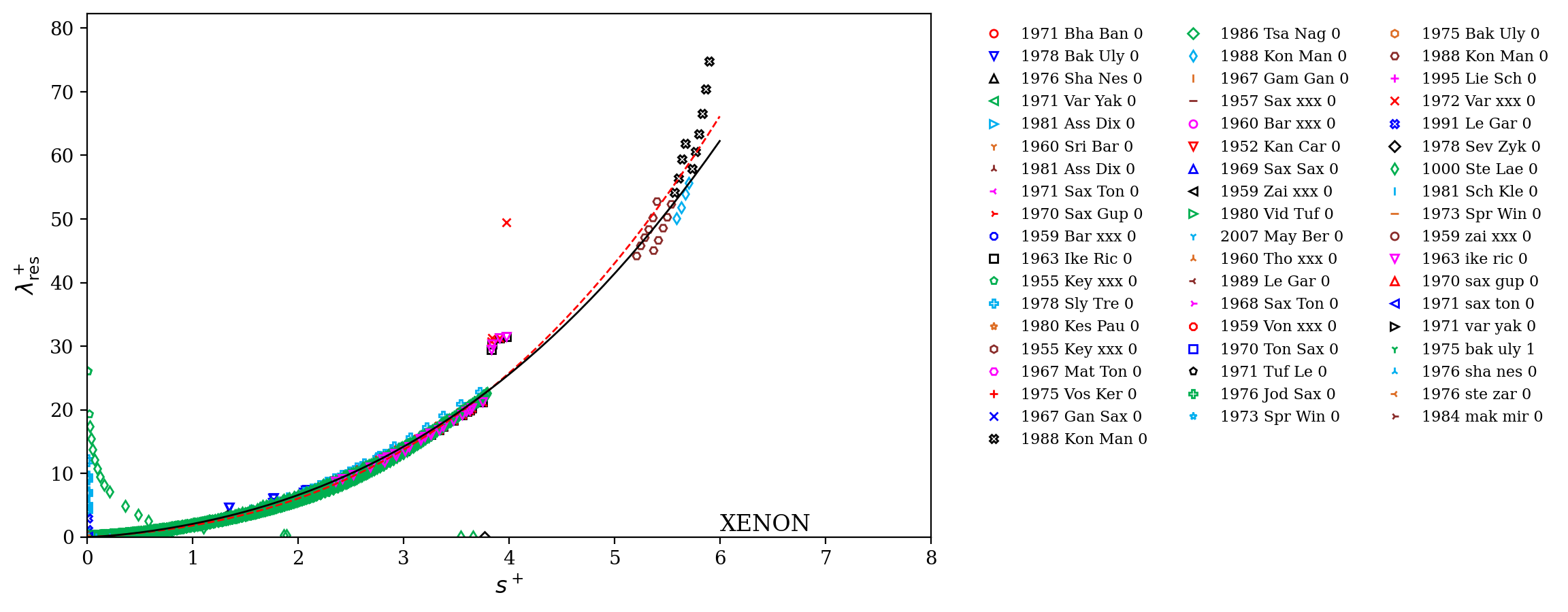

Supplement: Supplementary file 2 — ao4c10815_si_002.zip [file ao4c10815_si_002.zip › Supporting Information/Fig. TC1 - s_plus vs lambda_plus - all data - YFR EoS/XENON.png]

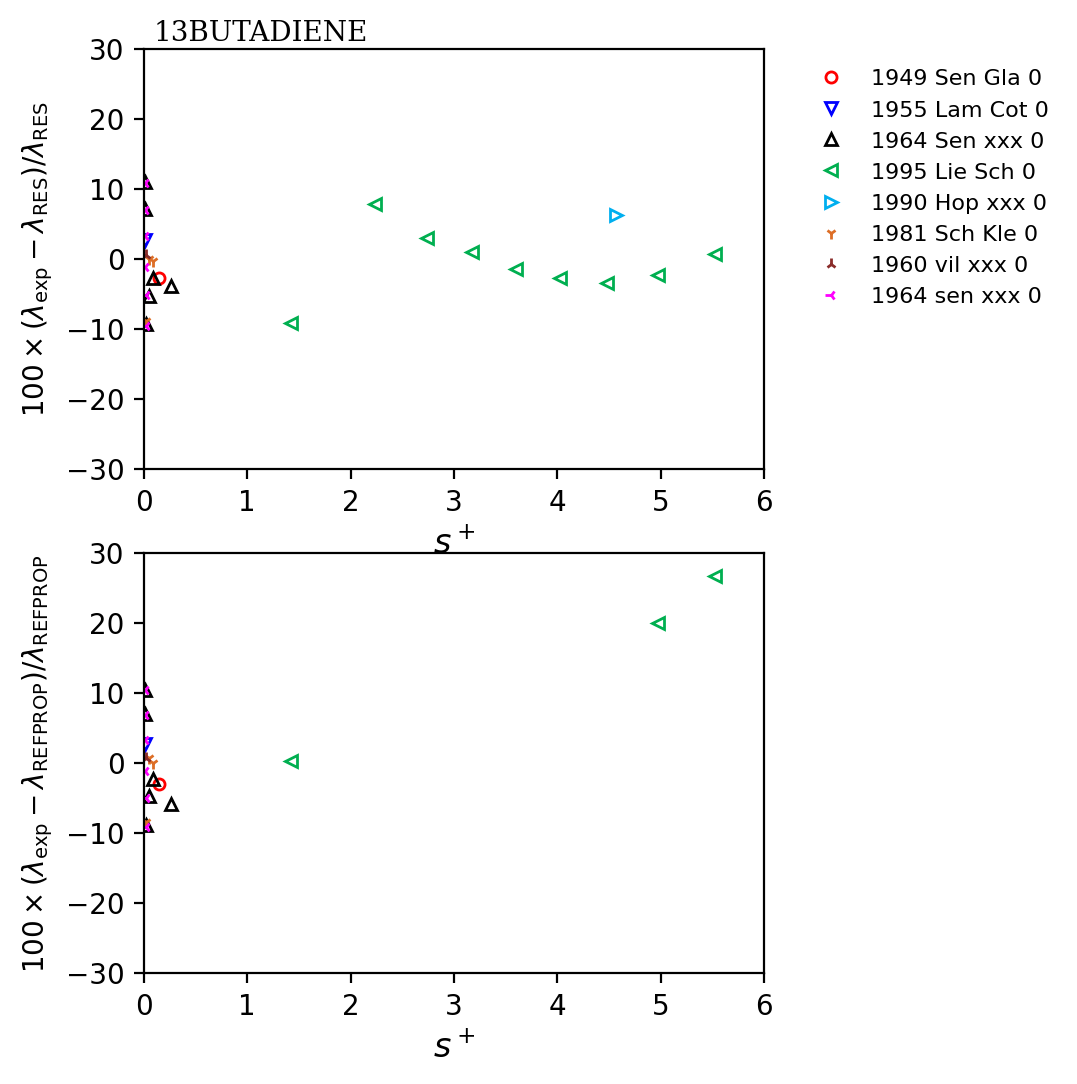

Supplement: Supplementary file 2 — ao4c10815_si_002.zip [file ao4c10815_si_002.zip › Supporting Information/Fig. TC2 - relative deviation - analyzable data - YFR EoS/13BUTADIENE.png]

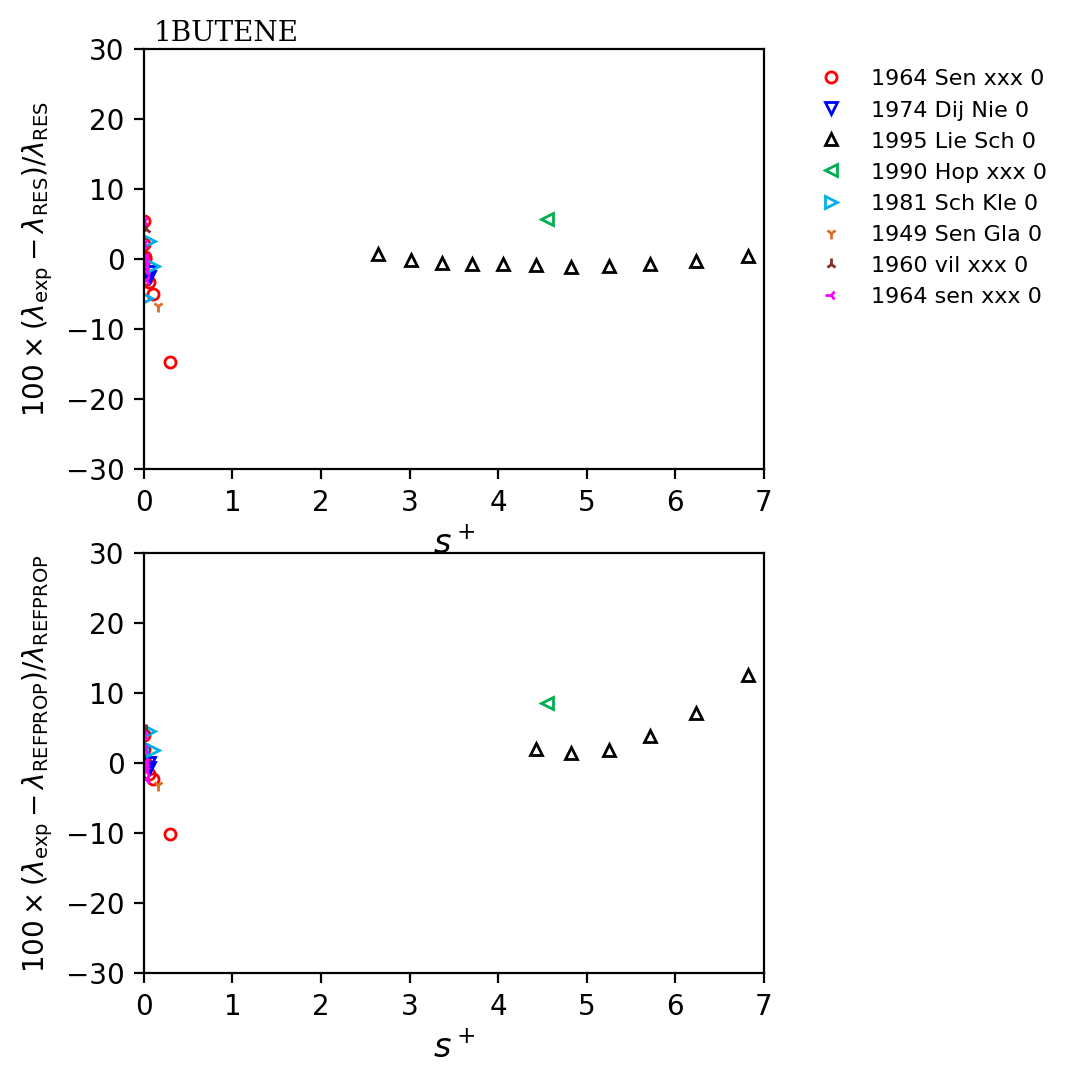

Supplement: Supplementary file 2 — ao4c10815_si_002.zip [file ao4c10815_si_002.zip › Supporting Information/Fig. TC2 - relative deviation - analyzable data - YFR EoS/1BUTENE.png]

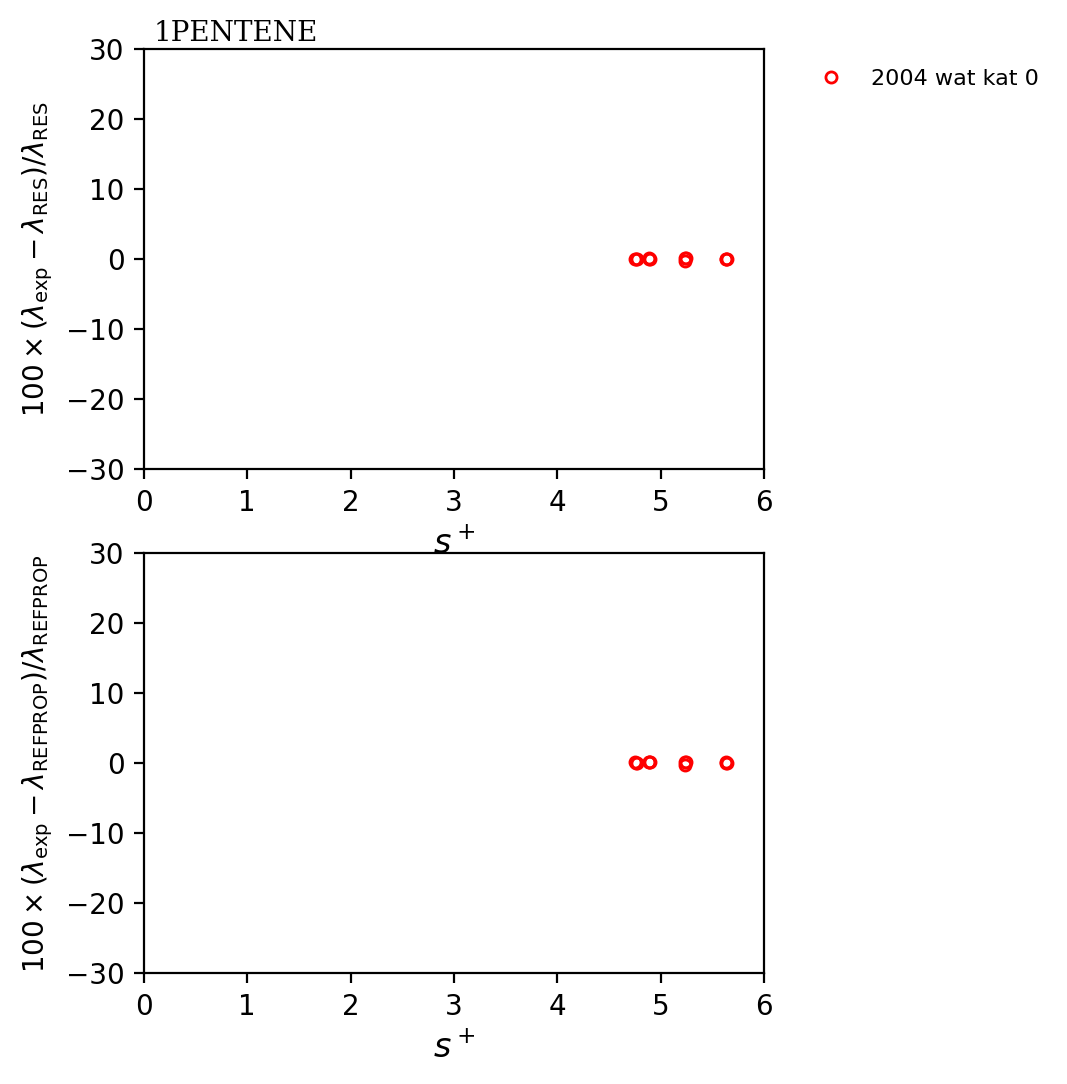

Supplement: Supplementary file 2 — ao4c10815_si_002.zip [file ao4c10815_si_002.zip › Supporting Information/Fig. TC2 - relative deviation - analyzable data - YFR EoS/1PENTENE.png]

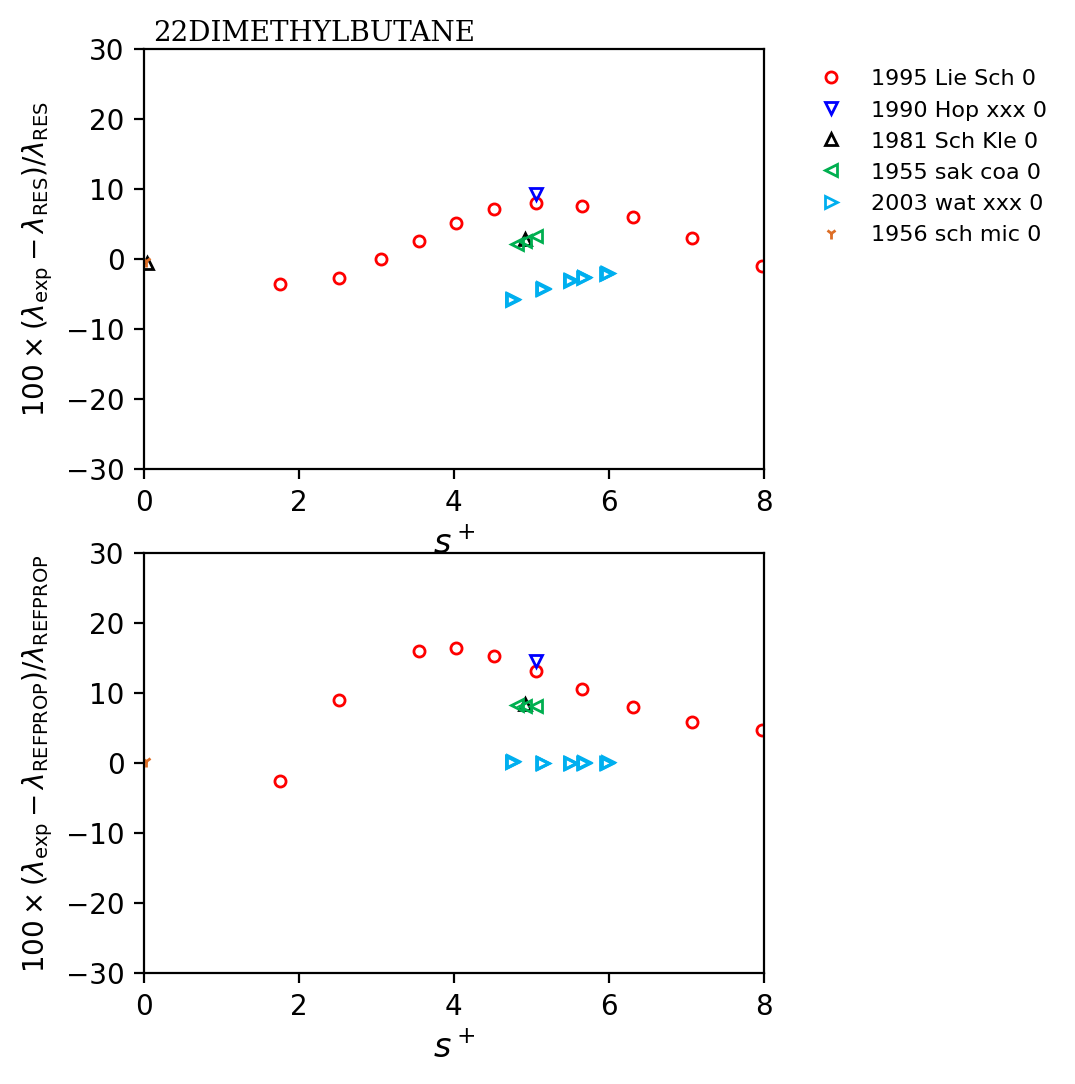

Supplement: Supplementary file 2 — ao4c10815_si_002.zip [file ao4c10815_si_002.zip › Supporting Information/Fig. TC2 - relative deviation - analyzable data - YFR EoS/22DIMETHYLBUTANE.png]

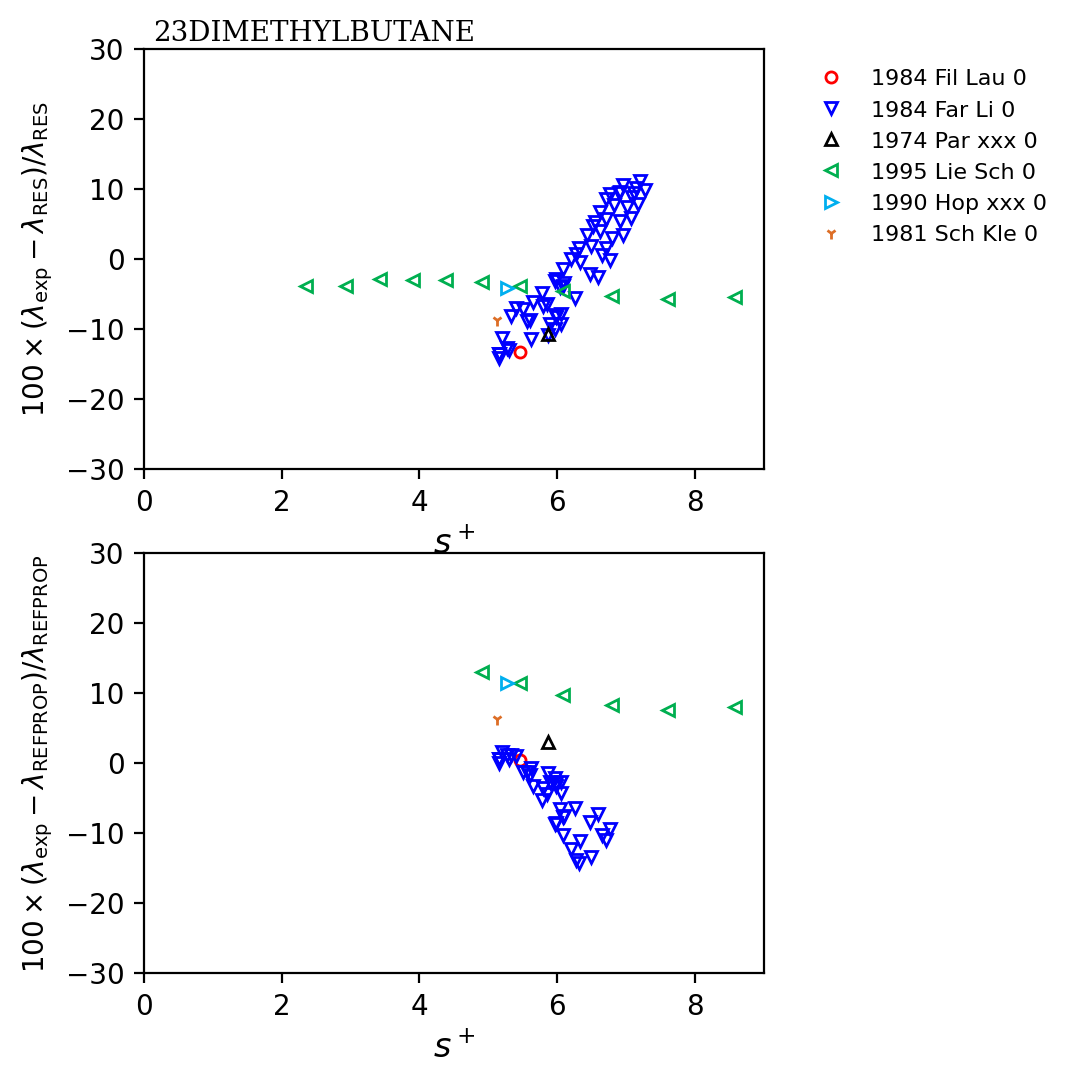

Supplement: Supplementary file 2 — ao4c10815_si_002.zip [file ao4c10815_si_002.zip › Supporting Information/Fig. TC2 - relative deviation - analyzable data - YFR EoS/23DIMETHYLBUTANE.png]

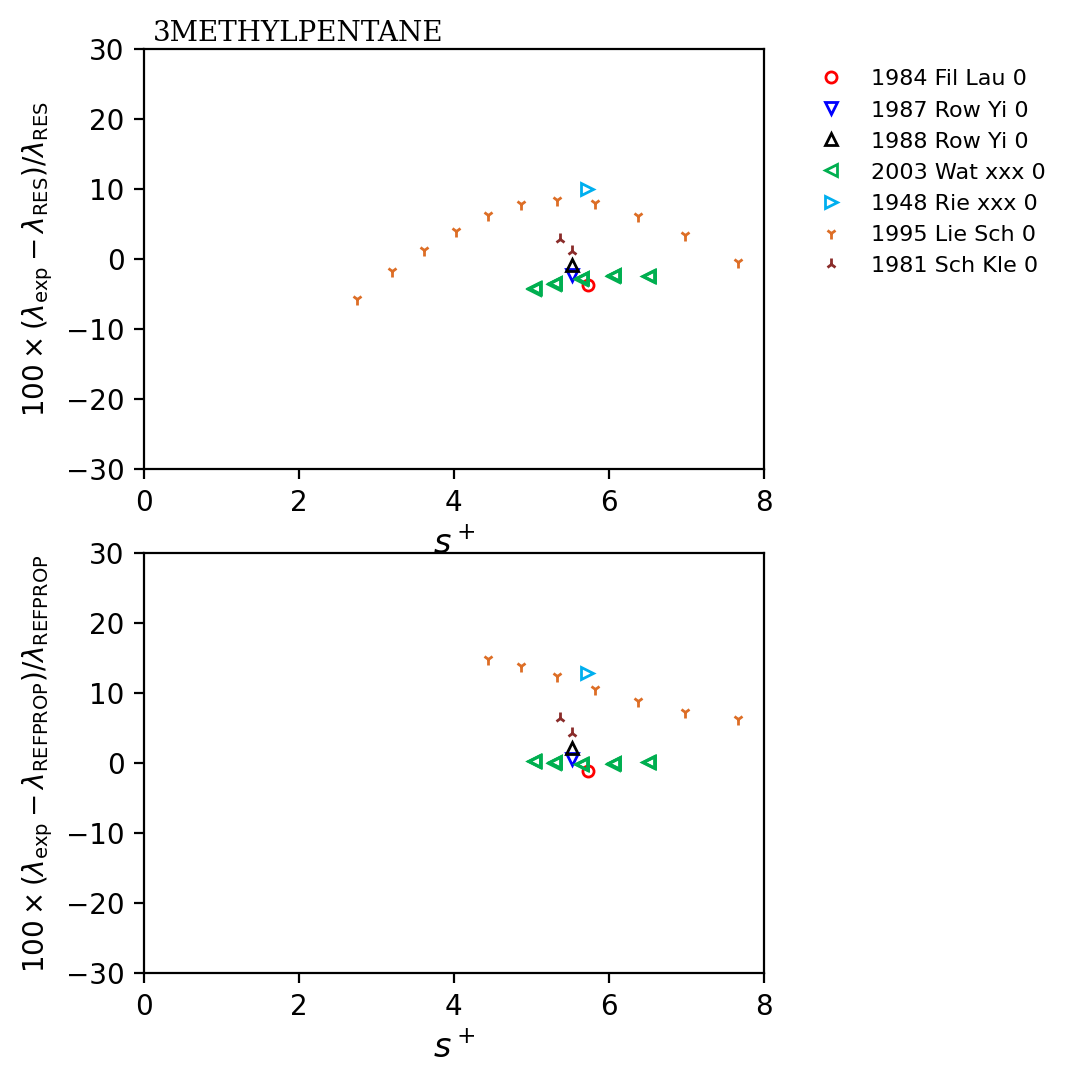

Supplement: Supplementary file 2 — ao4c10815_si_002.zip [file ao4c10815_si_002.zip › Supporting Information/Fig. TC2 - relative deviation - analyzable data - YFR EoS/3METHYLPENTANE.png]

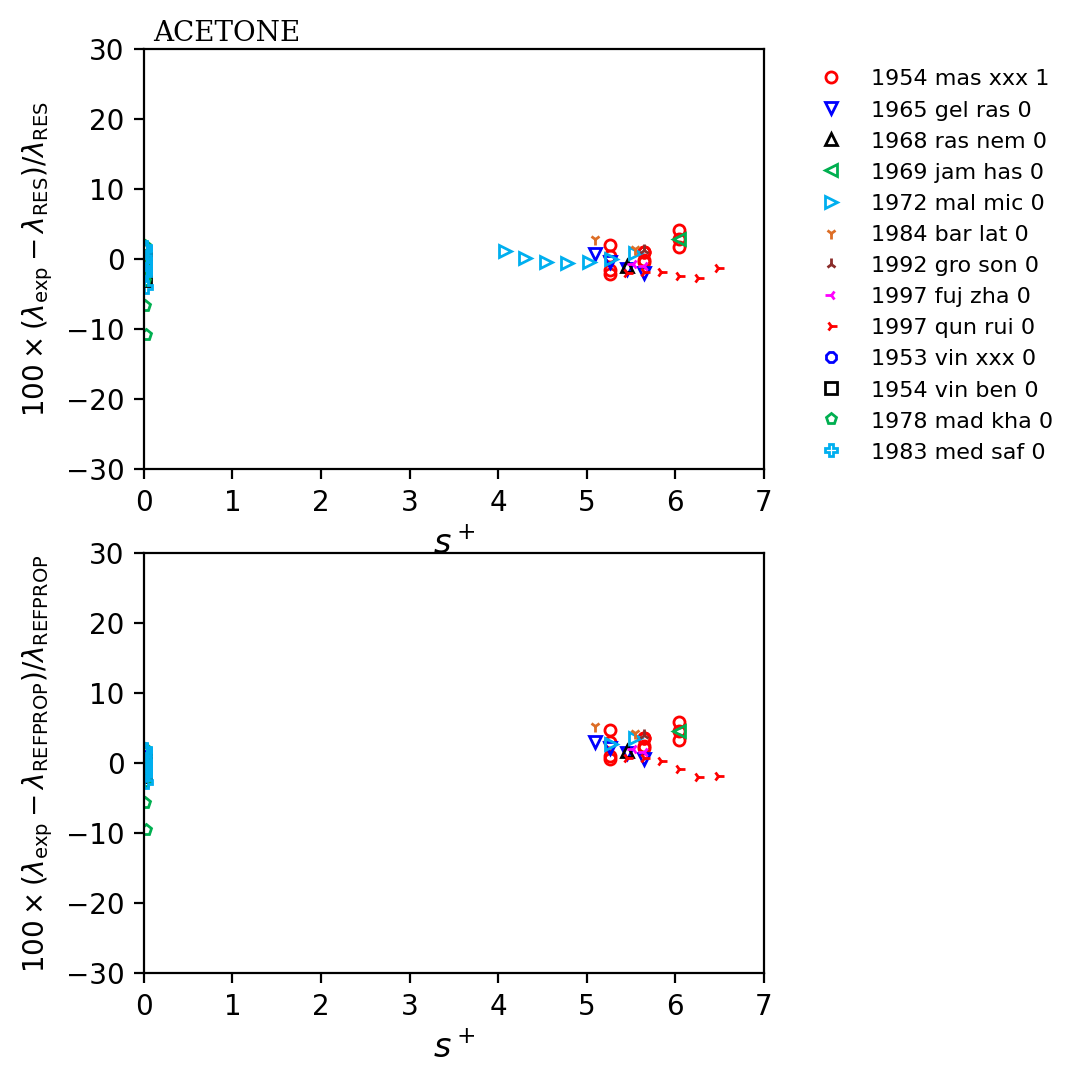

Supplement: Supplementary file 2 — ao4c10815_si_002.zip [file ao4c10815_si_002.zip › Supporting Information/Fig. TC2 - relative deviation - analyzable data - YFR EoS/ACETONE.png]

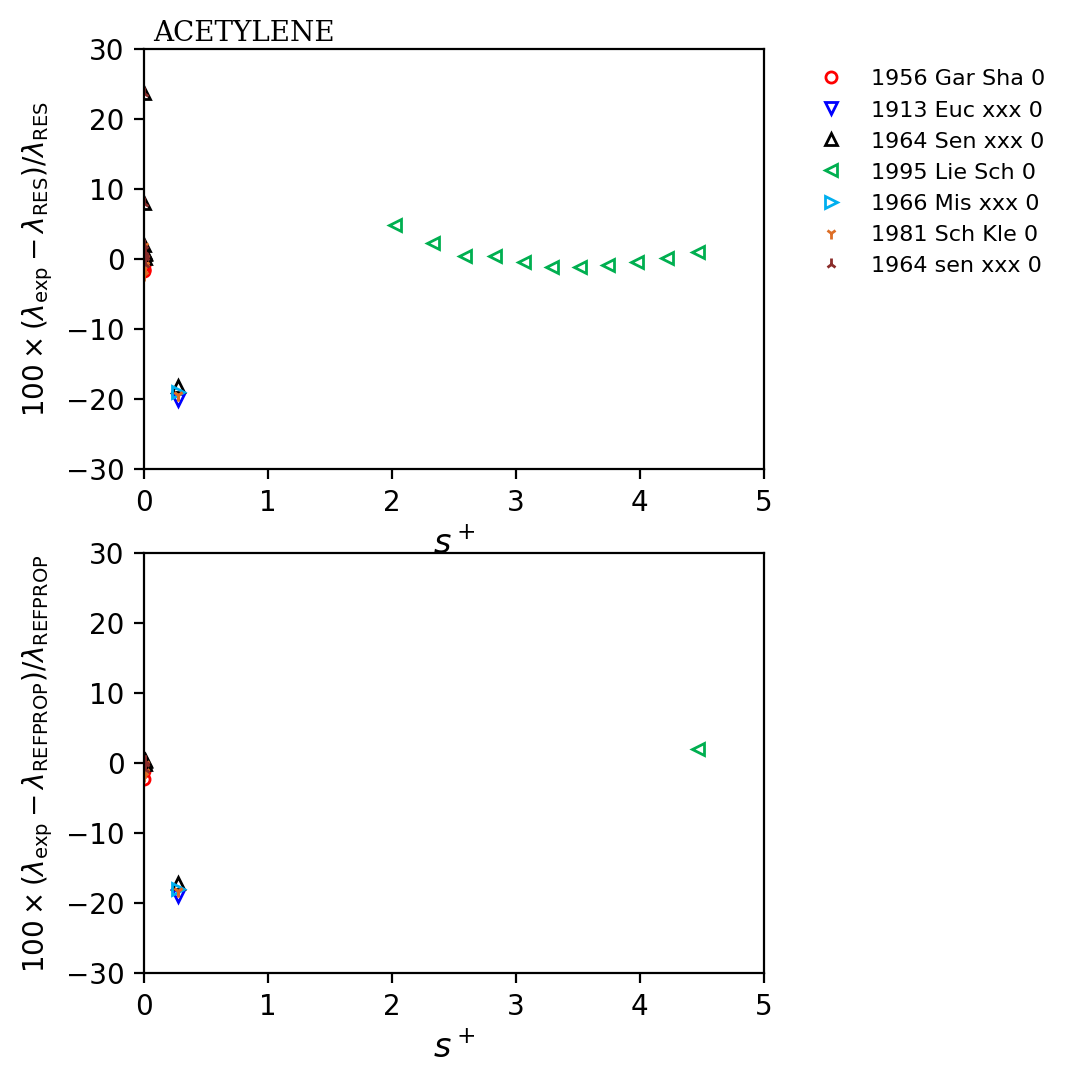

Supplement: Supplementary file 2 — ao4c10815_si_002.zip [file ao4c10815_si_002.zip › Supporting Information/Fig. TC2 - relative deviation - analyzable data - YFR EoS/ACETYLENE.png]

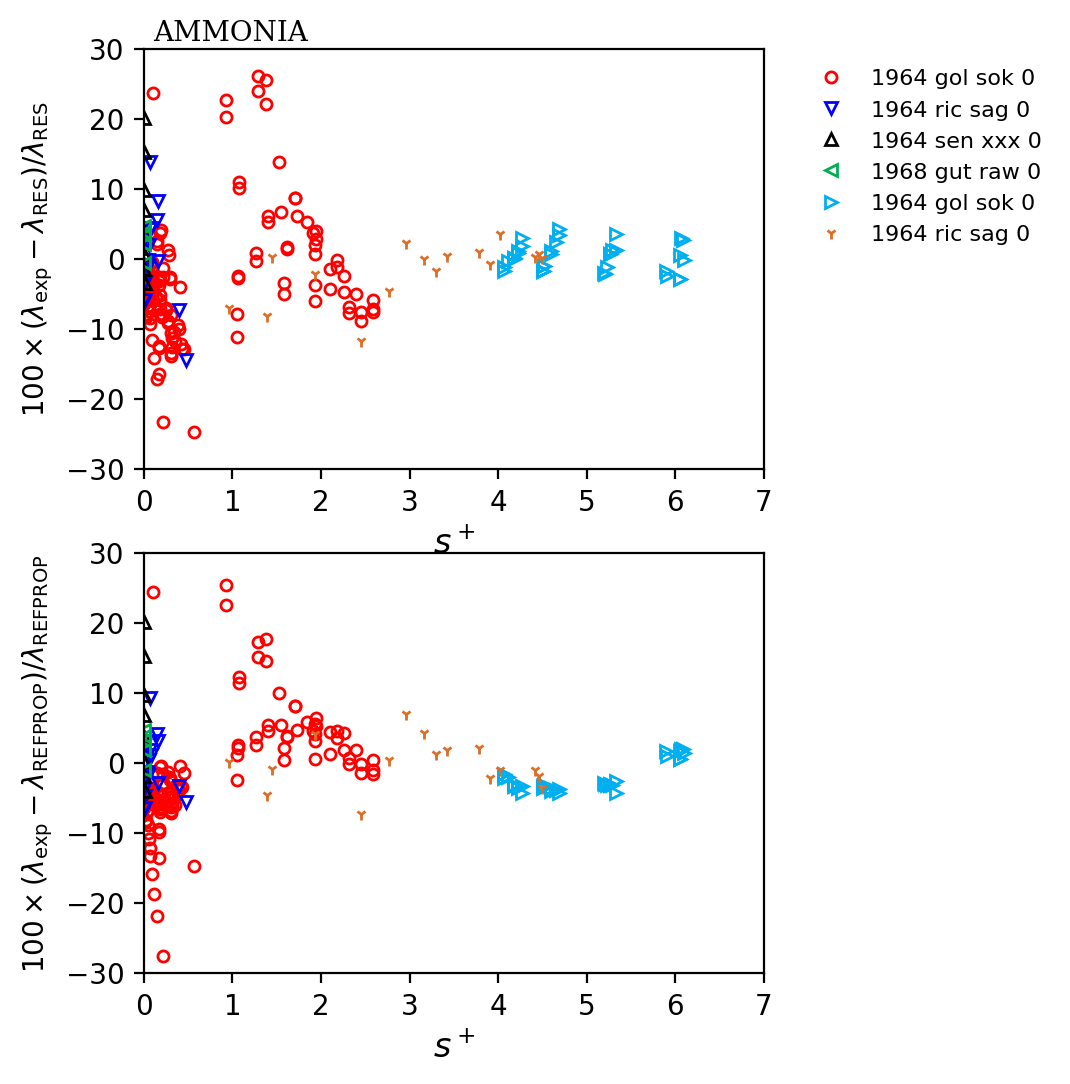

Supplement: Supplementary file 2 — ao4c10815_si_002.zip [file ao4c10815_si_002.zip › Supporting Information/Fig. TC2 - relative deviation - analyzable data - YFR EoS/AMMONIA.png]

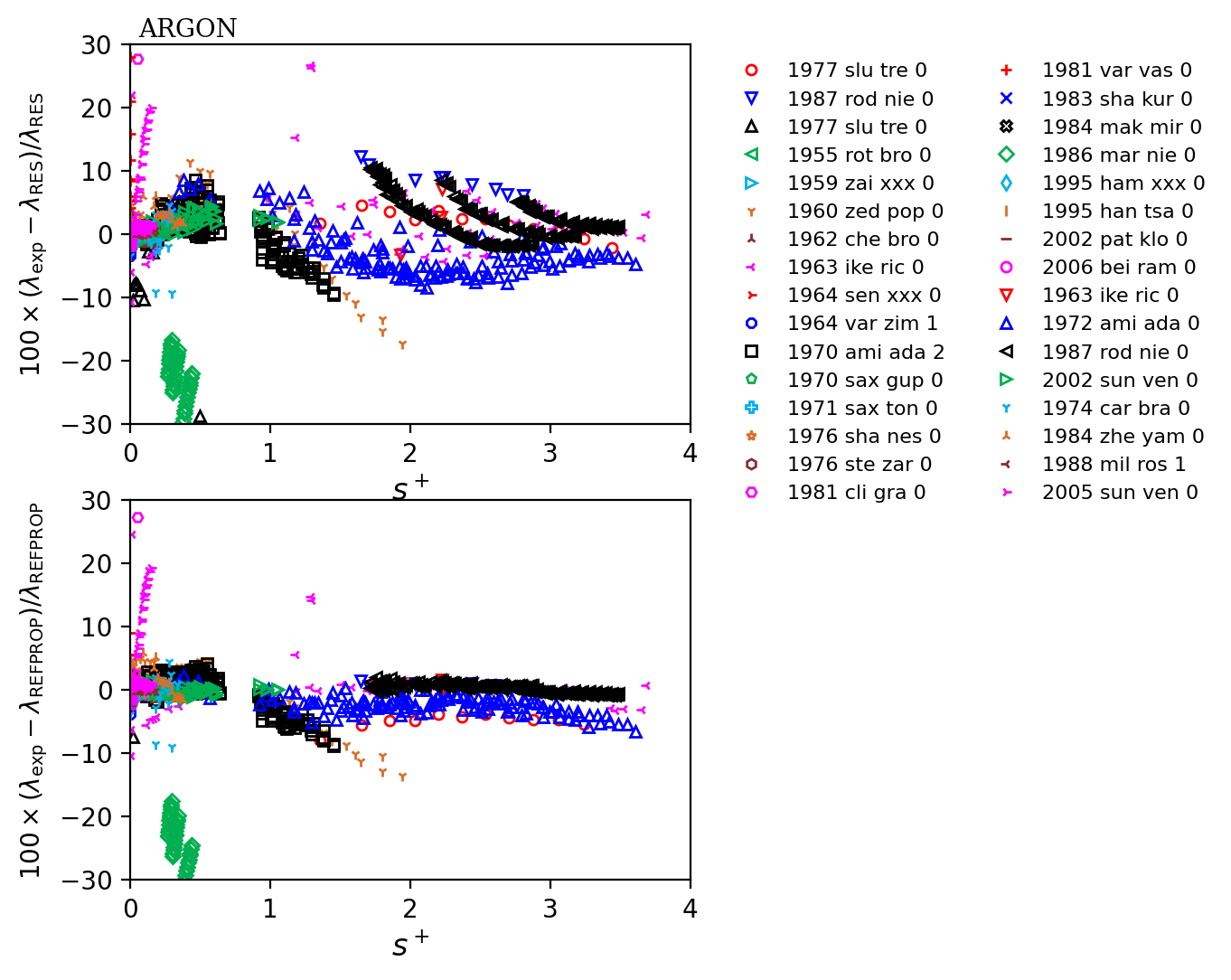

Supplement: Supplementary file 2 — ao4c10815_si_002.zip [file ao4c10815_si_002.zip › Supporting Information/Fig. TC2 - relative deviation - analyzable data - YFR EoS/ARGON.png]

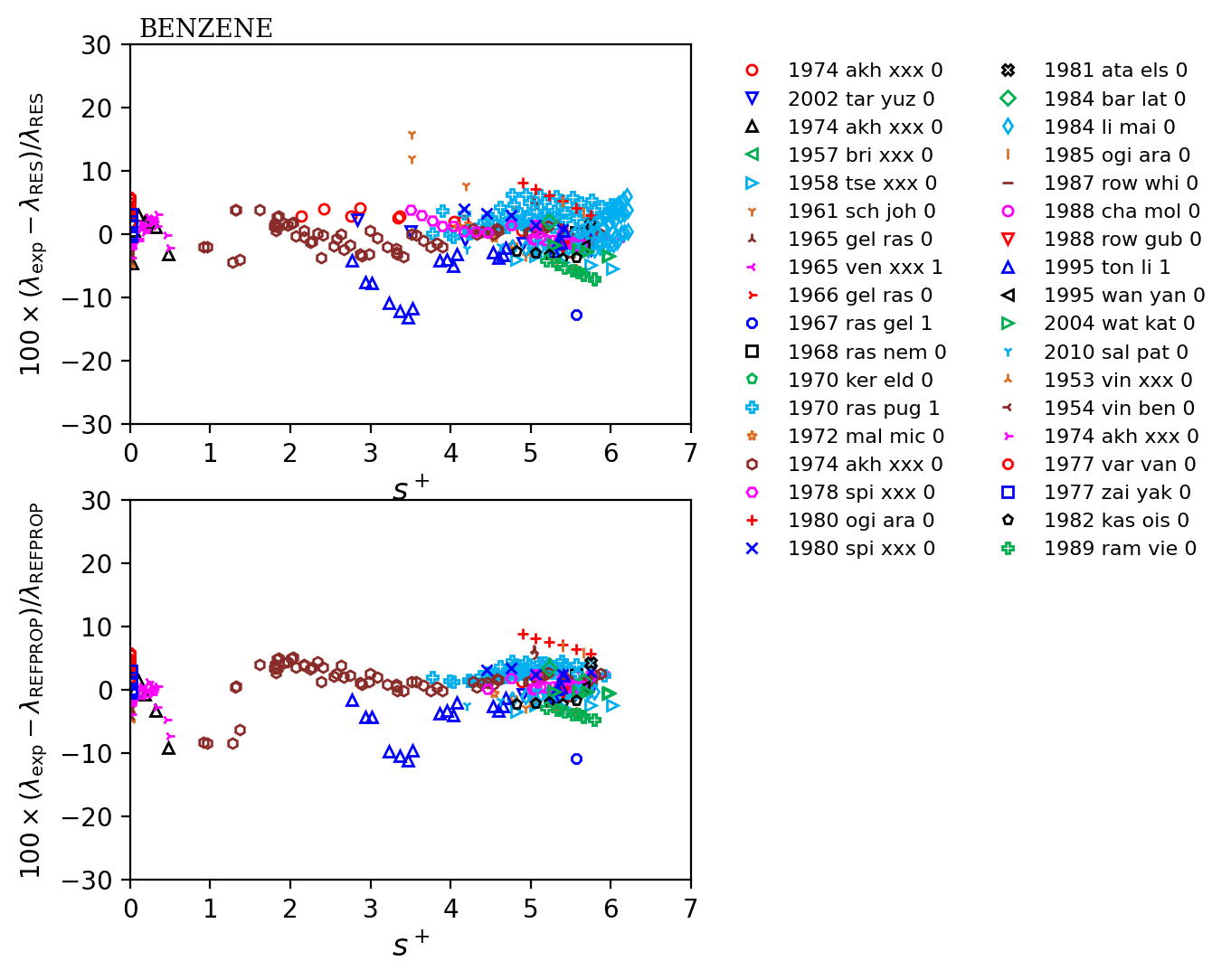

Supplement: Supplementary file 2 — ao4c10815_si_002.zip [file ao4c10815_si_002.zip › Supporting Information/Fig. TC2 - relative deviation - analyzable data - YFR EoS/BENZENE.png]

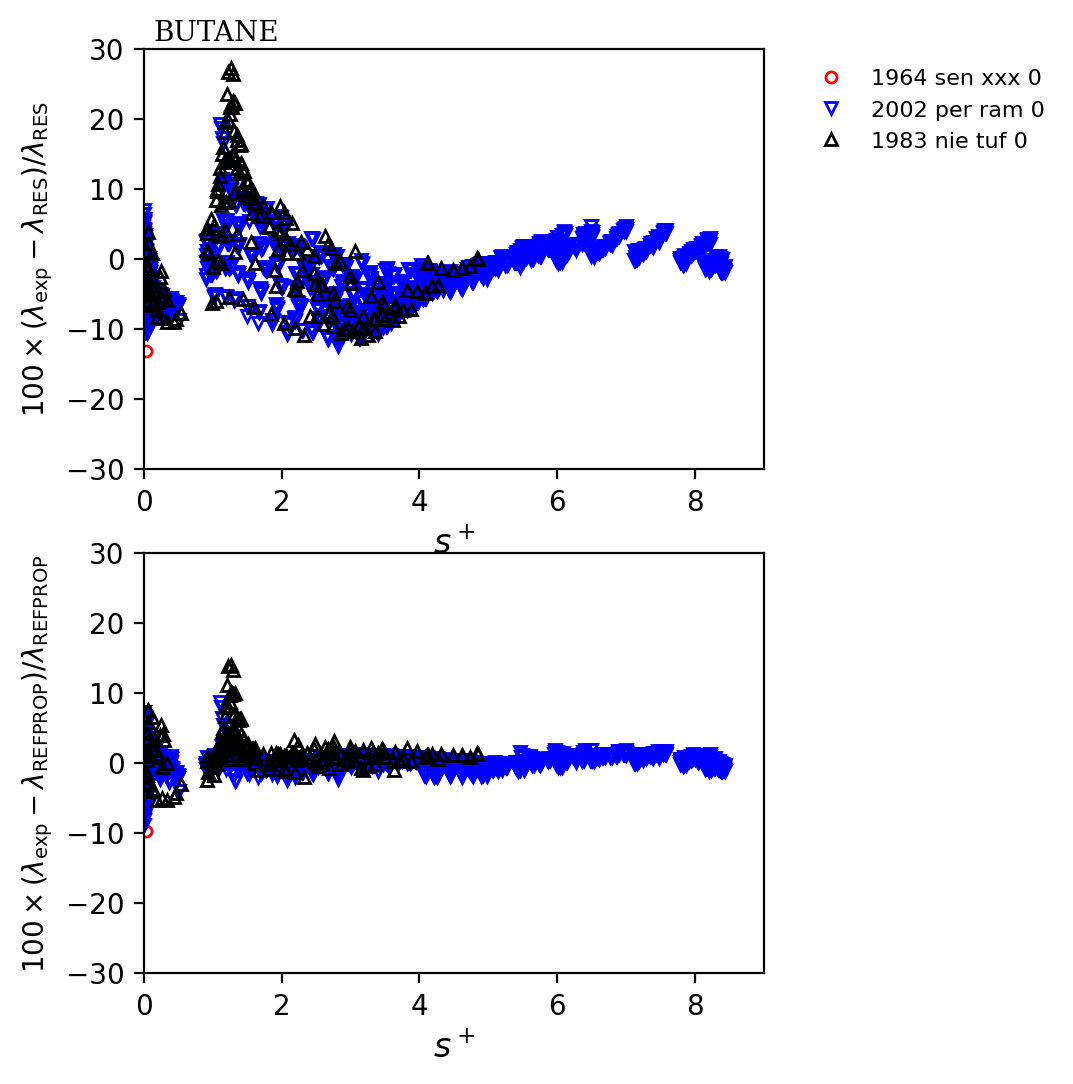

Supplement: Supplementary file 2 — ao4c10815_si_002.zip [file ao4c10815_si_002.zip › Supporting Information/Fig. TC2 - relative deviation - analyzable data - YFR EoS/BUTANE.png]

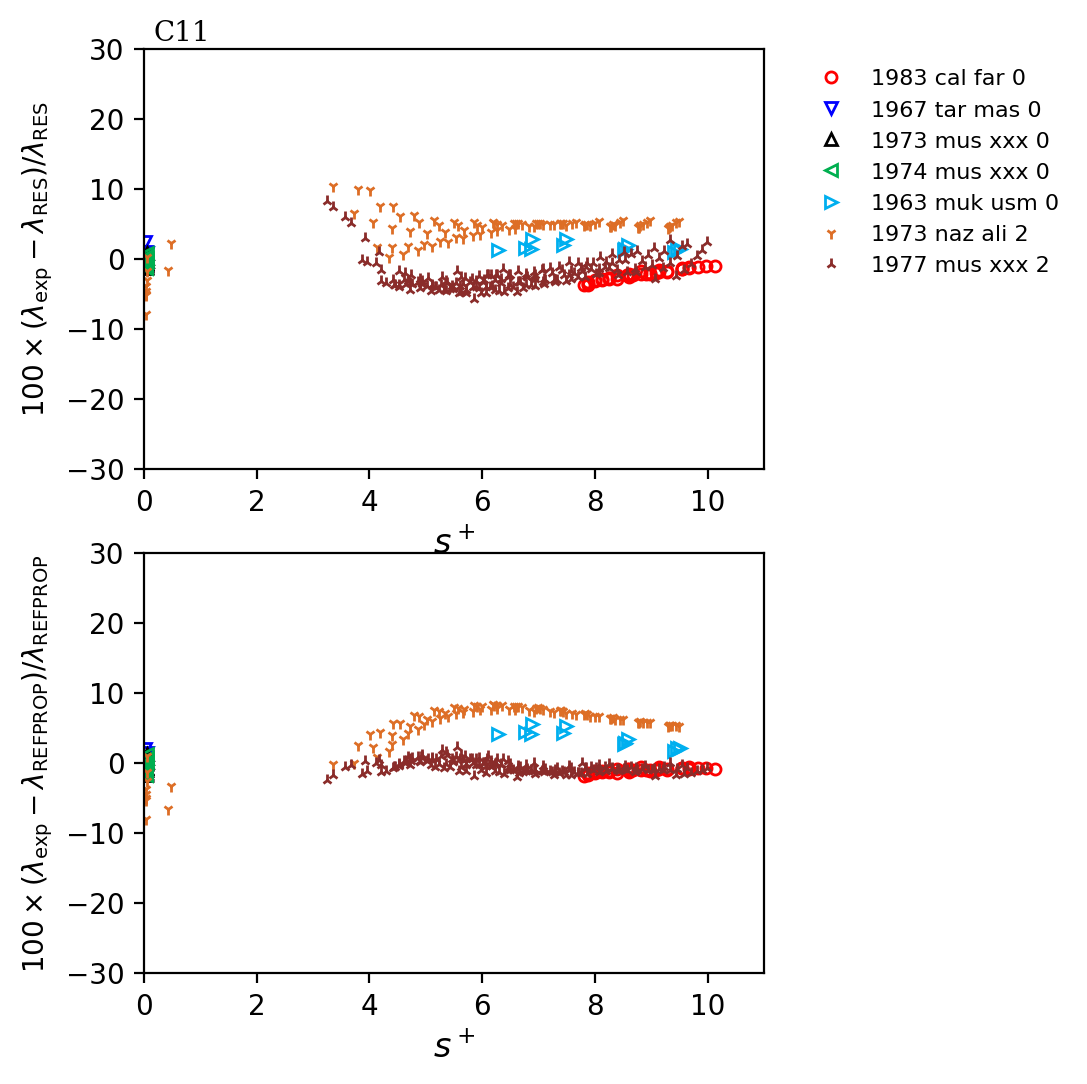

Supplement: Supplementary file 2 — ao4c10815_si_002.zip [file ao4c10815_si_002.zip › Supporting Information/Fig. TC2 - relative deviation - analyzable data - YFR EoS/C11.png]

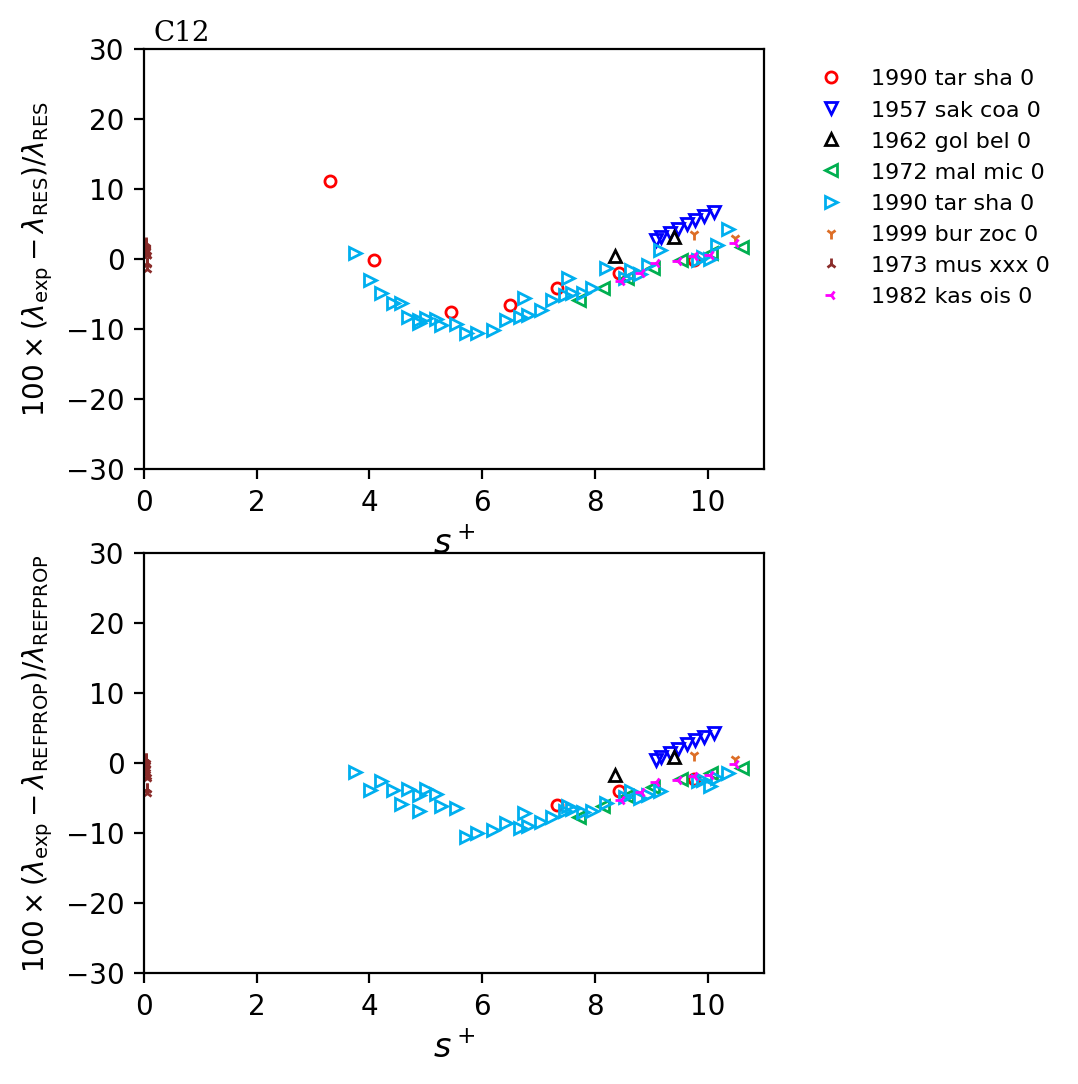

Supplement: Supplementary file 2 — ao4c10815_si_002.zip [file ao4c10815_si_002.zip › Supporting Information/Fig. TC2 - relative deviation - analyzable data - YFR EoS/C12.png]

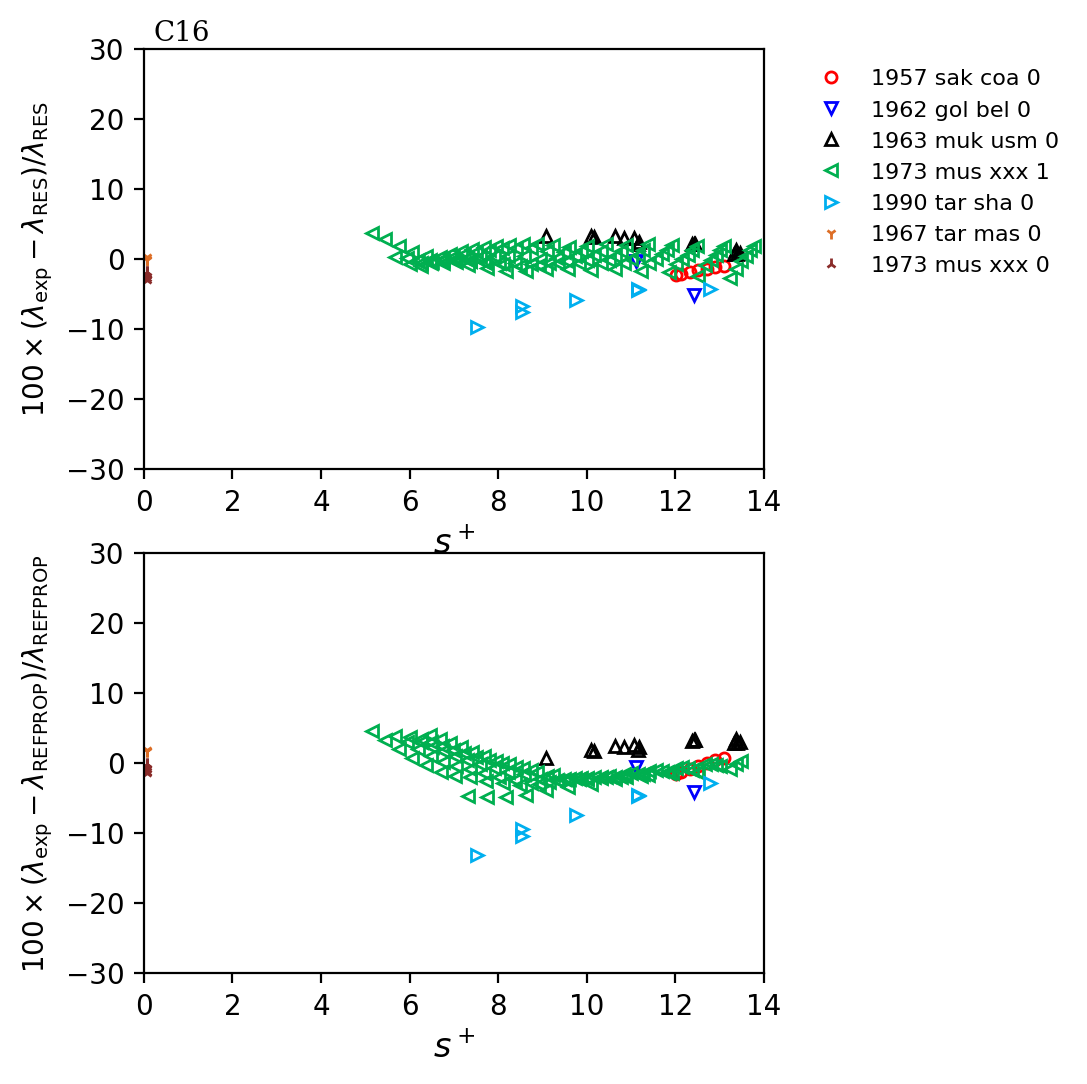

Supplement: Supplementary file 2 — ao4c10815_si_002.zip [file ao4c10815_si_002.zip › Supporting Information/Fig. TC2 - relative deviation - analyzable data - YFR EoS/C16.png]

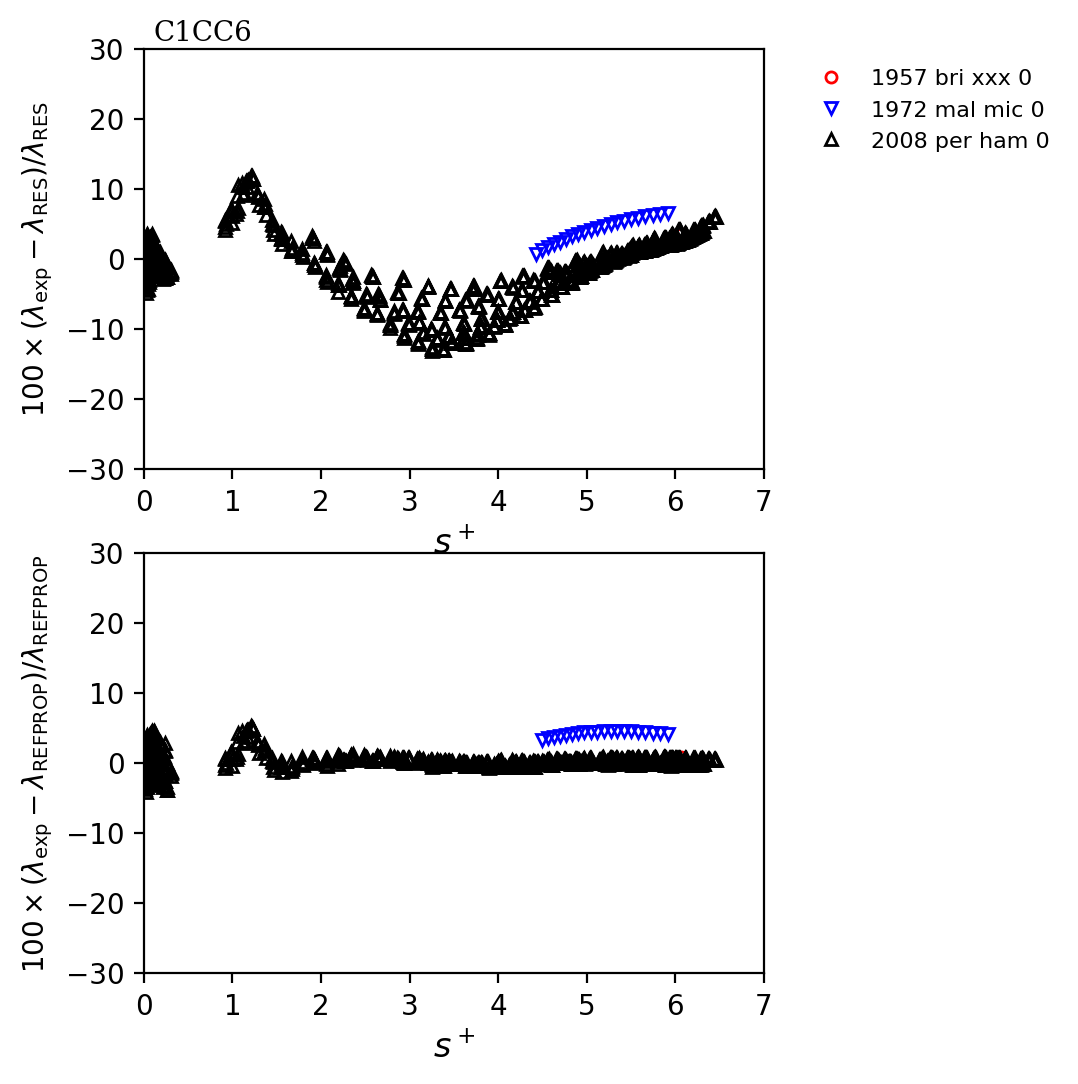

Supplement: Supplementary file 2 — ao4c10815_si_002.zip [file ao4c10815_si_002.zip › Supporting Information/Fig. TC2 - relative deviation - analyzable data - YFR EoS/C1CC6.png]

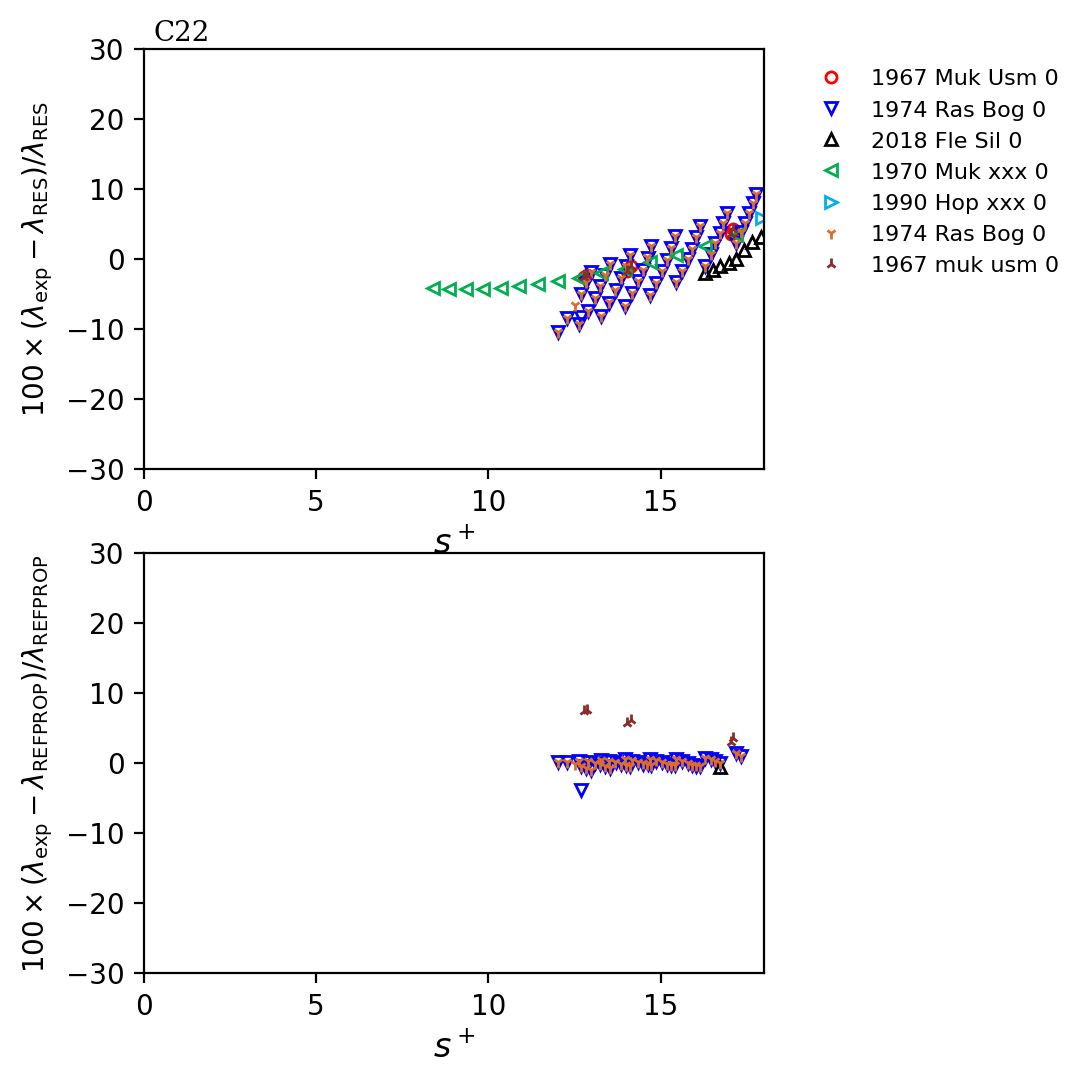

Supplement: Supplementary file 2 — ao4c10815_si_002.zip [file ao4c10815_si_002.zip › Supporting Information/Fig. TC2 - relative deviation - analyzable data - YFR EoS/C22.png]

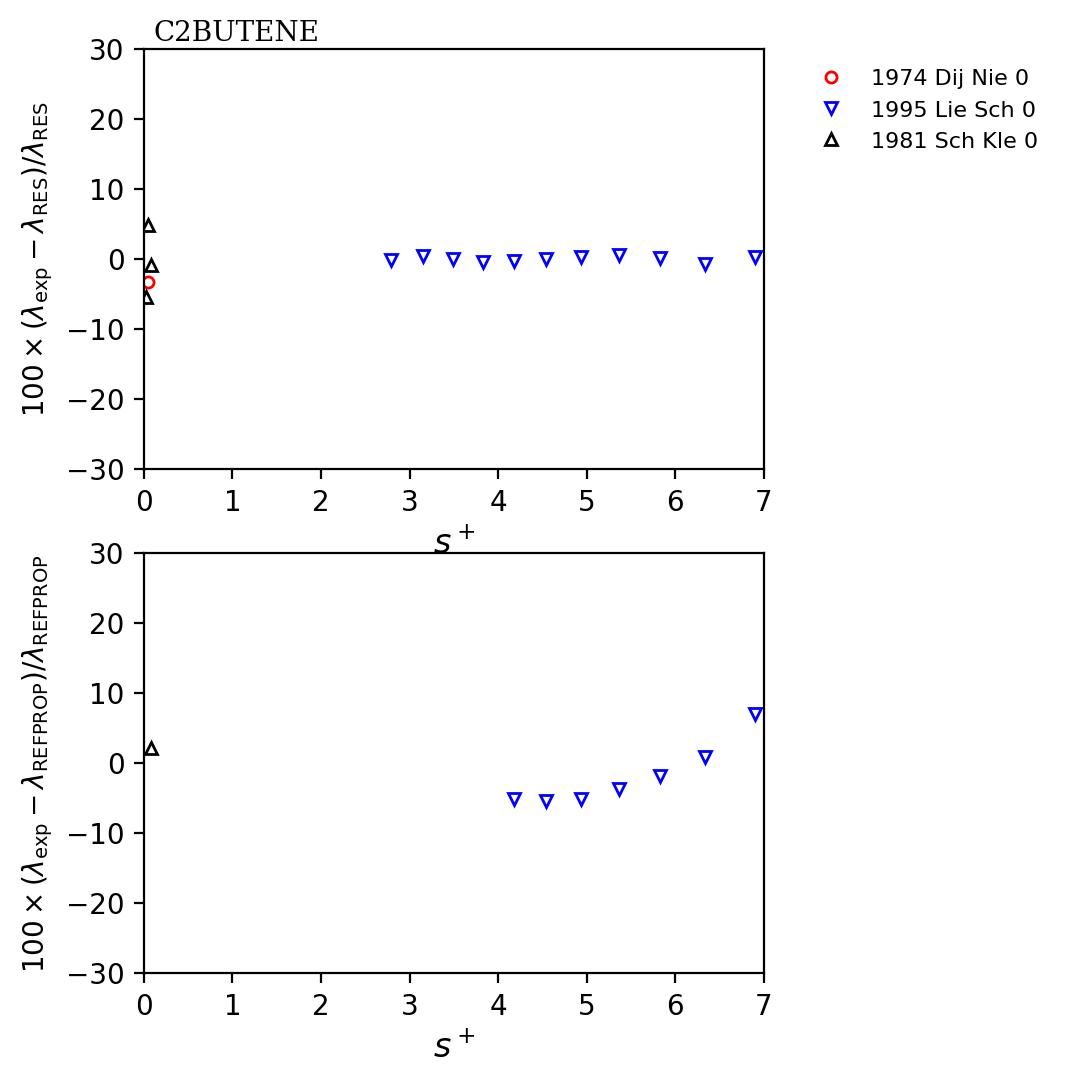

Supplement: Supplementary file 2 — ao4c10815_si_002.zip [file ao4c10815_si_002.zip › Supporting Information/Fig. TC2 - relative deviation - analyzable data - YFR EoS/C2BUTENE.png]

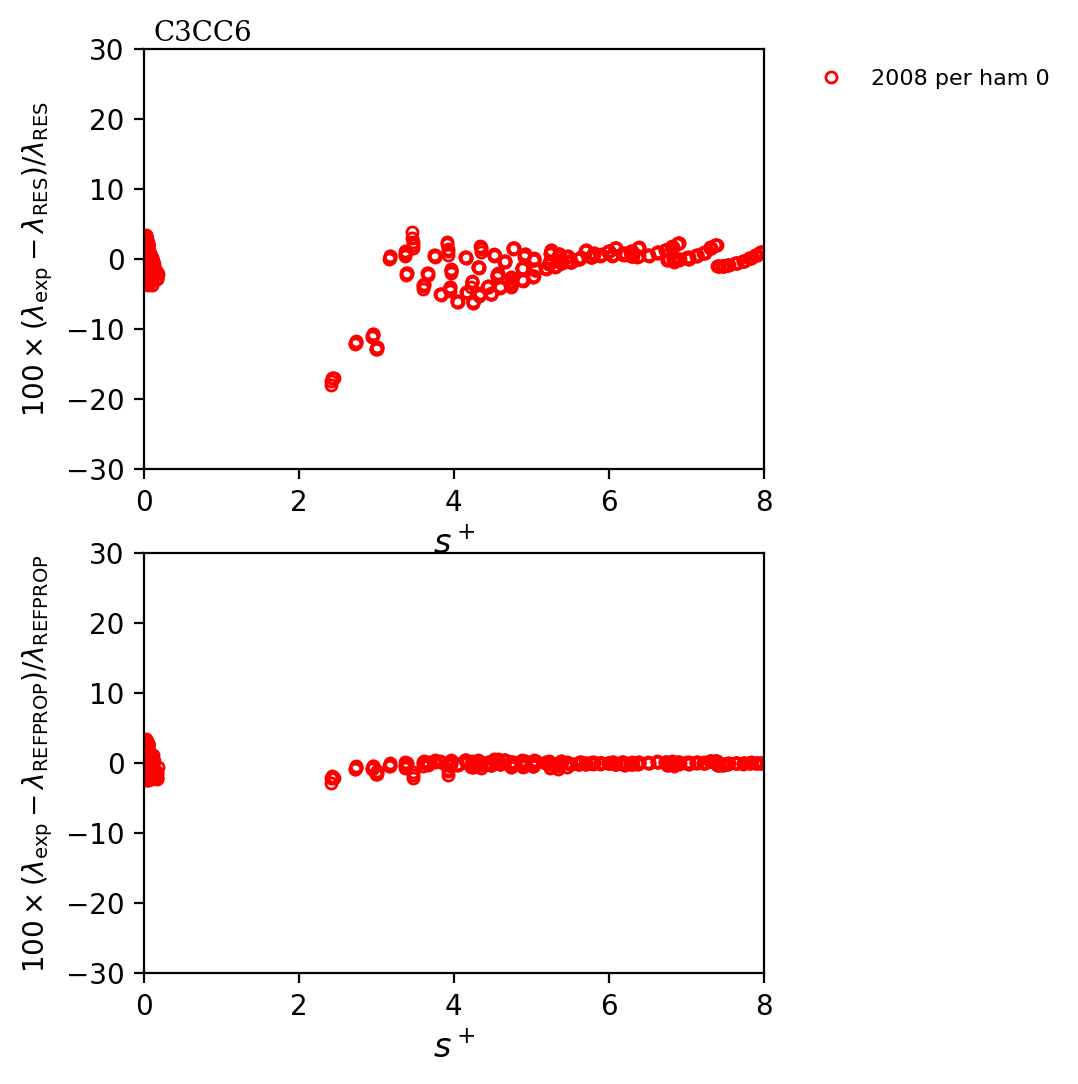

Supplement: Supplementary file 2 — ao4c10815_si_002.zip [file ao4c10815_si_002.zip › Supporting Information/Fig. TC2 - relative deviation - analyzable data - YFR EoS/C3CC6.png]

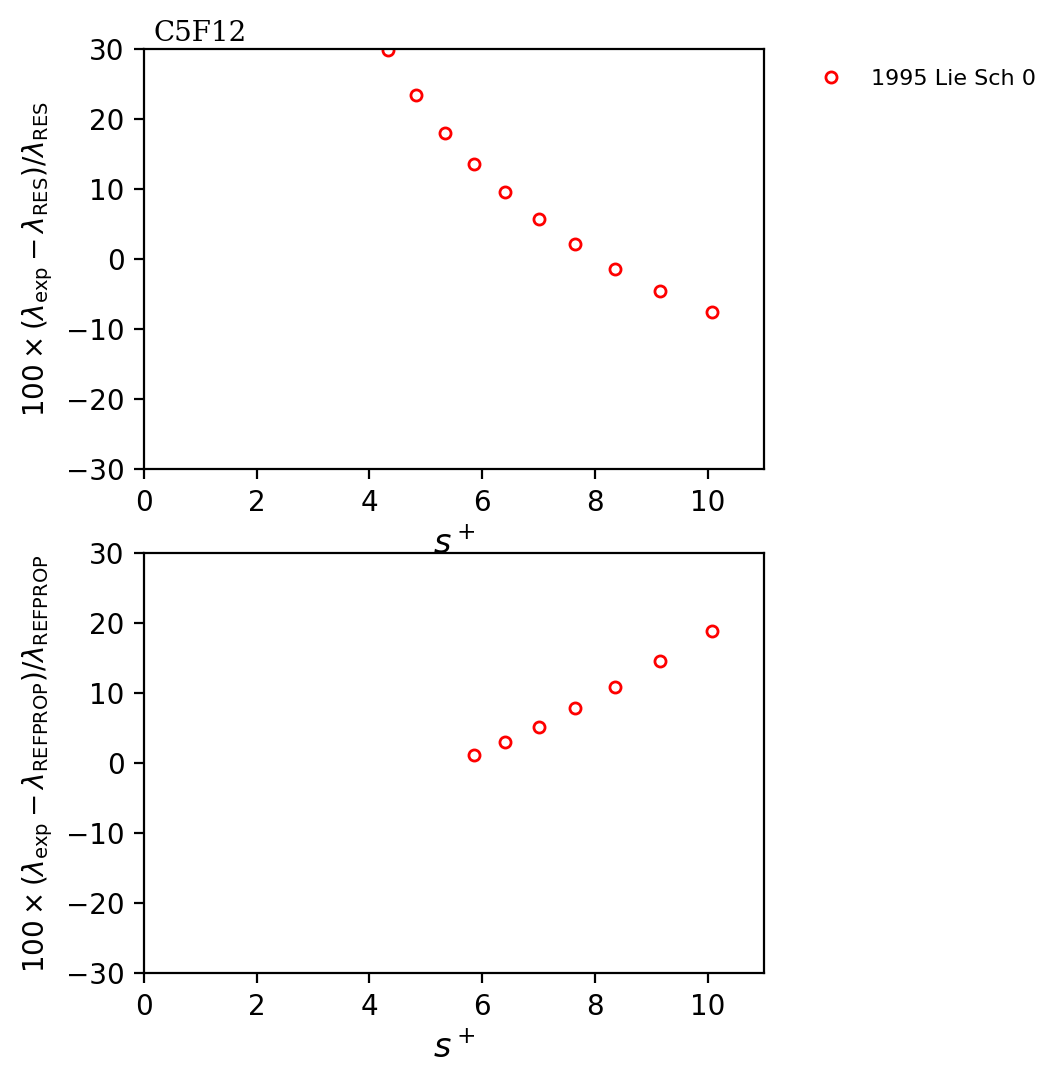

Supplement: Supplementary file 2 — ao4c10815_si_002.zip [file ao4c10815_si_002.zip › Supporting Information/Fig. TC2 - relative deviation - analyzable data - YFR EoS/C5F12.png]

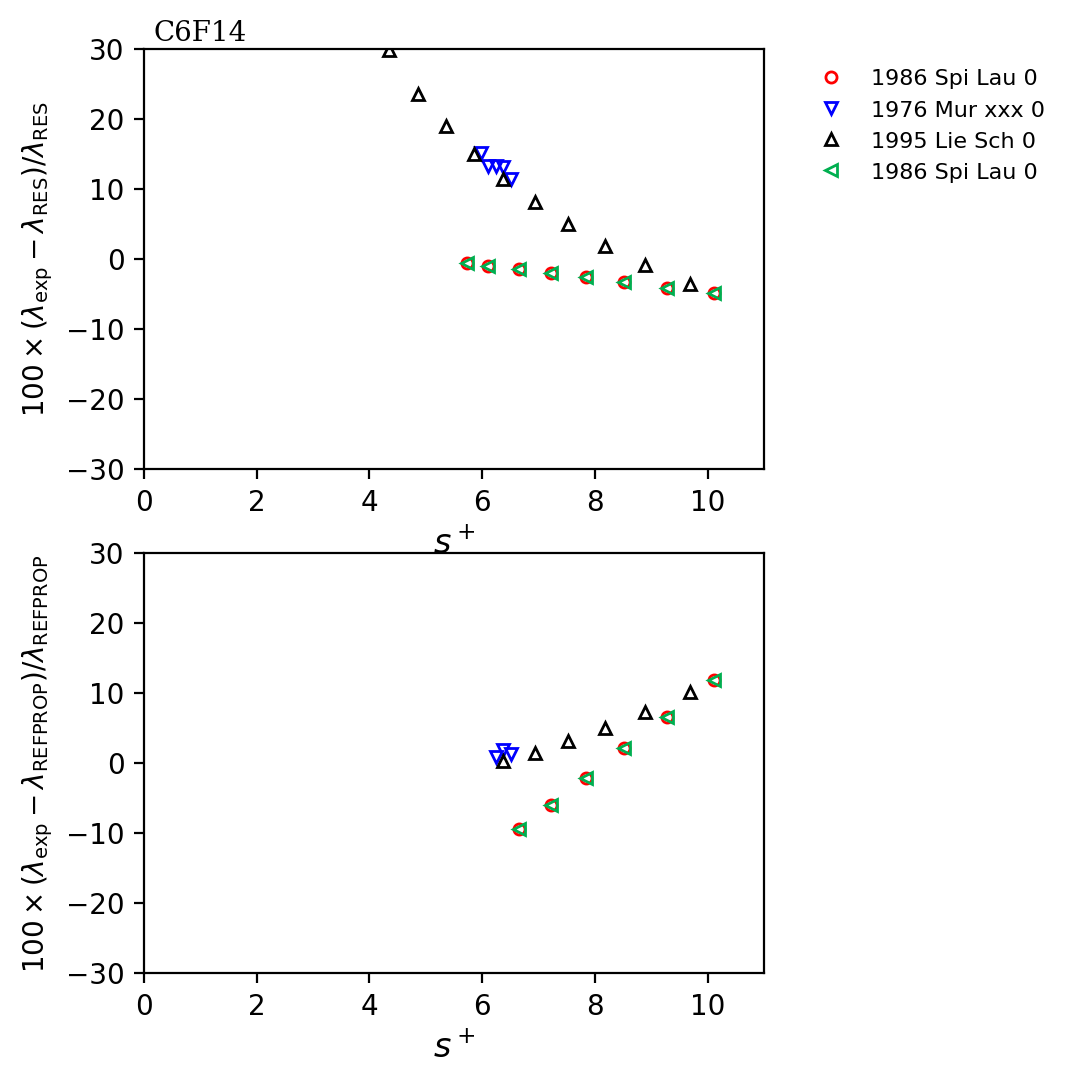

Supplement: Supplementary file 2 — ao4c10815_si_002.zip [file ao4c10815_si_002.zip › Supporting Information/Fig. TC2 - relative deviation - analyzable data - YFR EoS/C6F14.png]

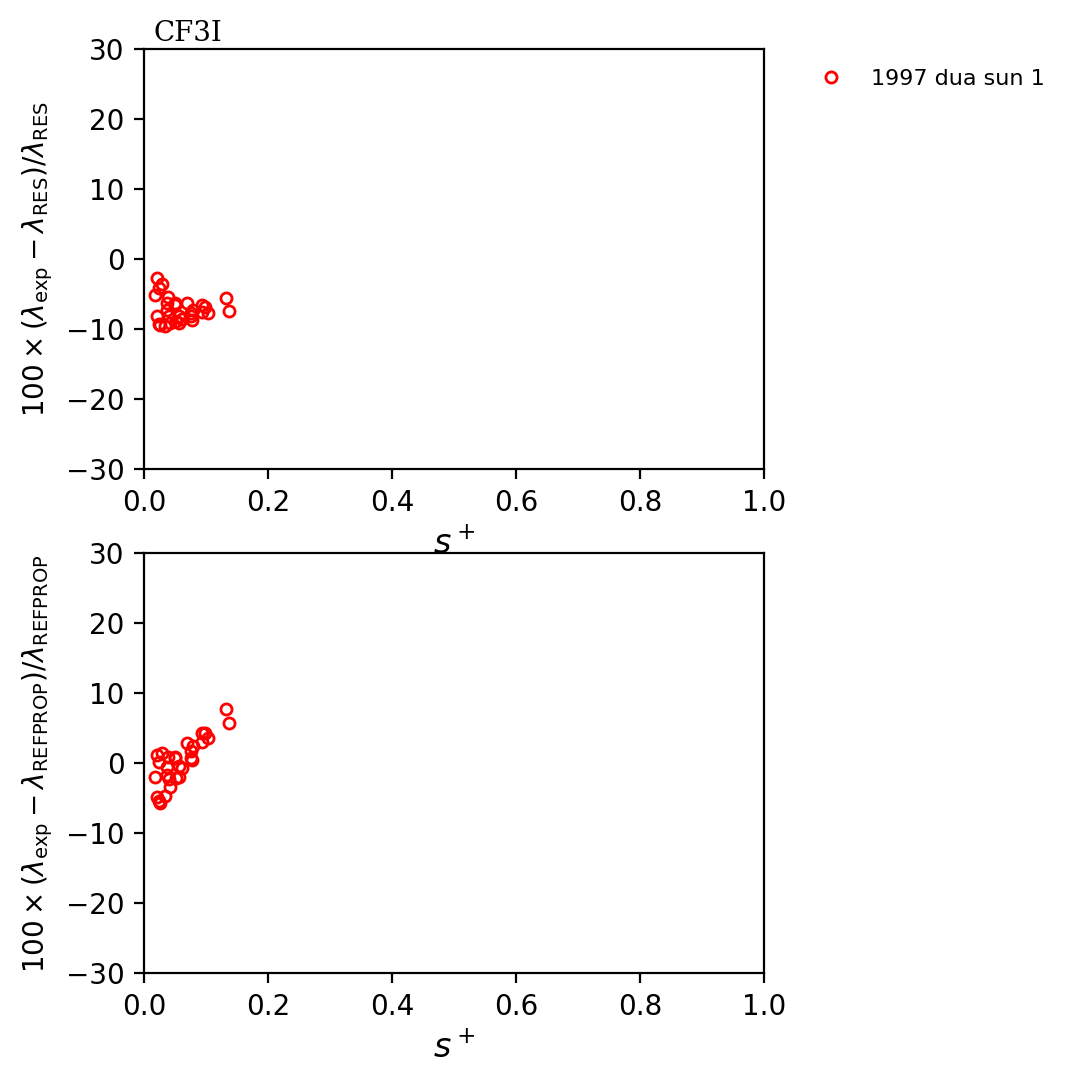

Supplement: Supplementary file 2 — ao4c10815_si_002.zip [file ao4c10815_si_002.zip › Supporting Information/Fig. TC2 - relative deviation - analyzable data - YFR EoS/CF3I.png]

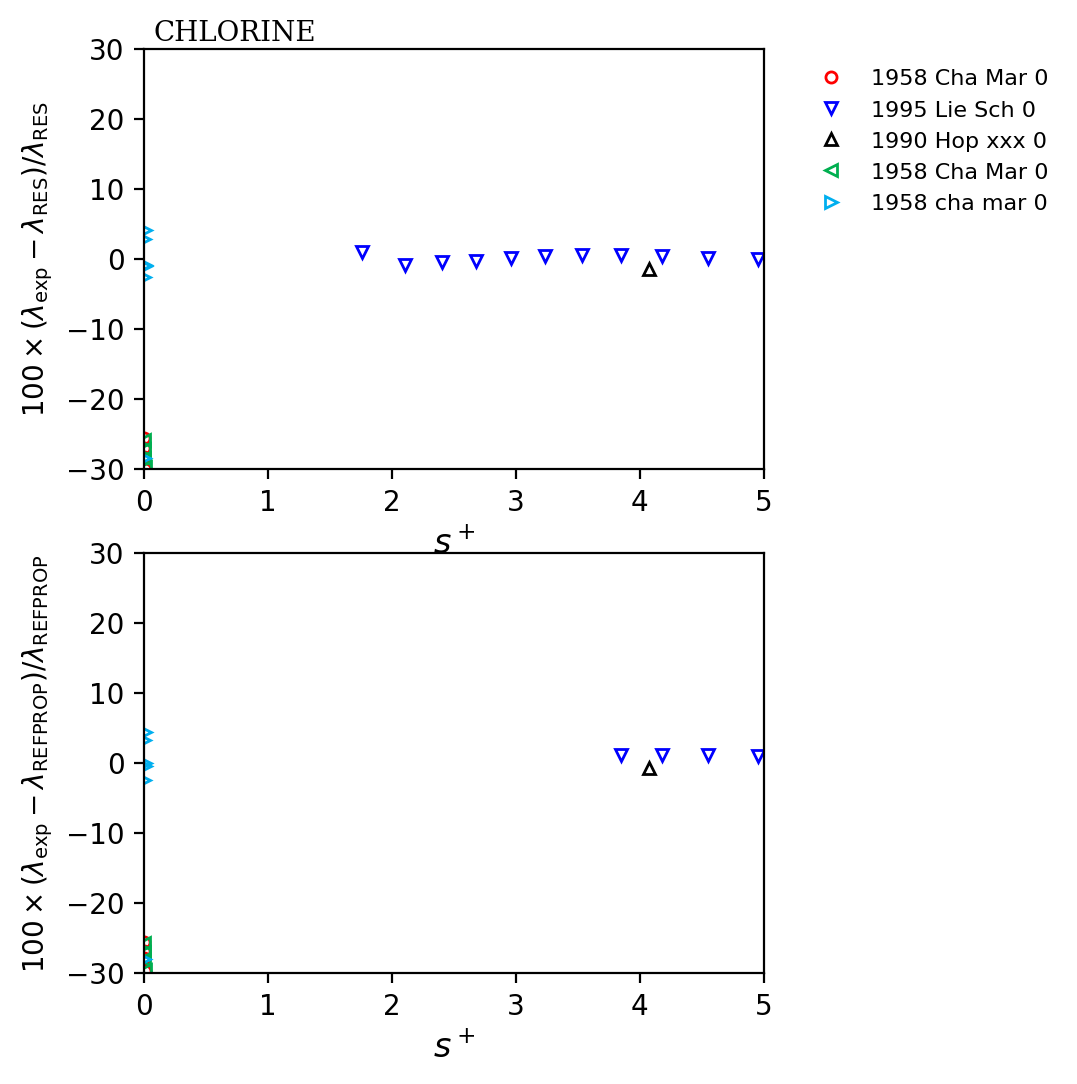

Supplement: Supplementary file 2 — ao4c10815_si_002.zip [file ao4c10815_si_002.zip › Supporting Information/Fig. TC2 - relative deviation - analyzable data - YFR EoS/CHLORINE.png]

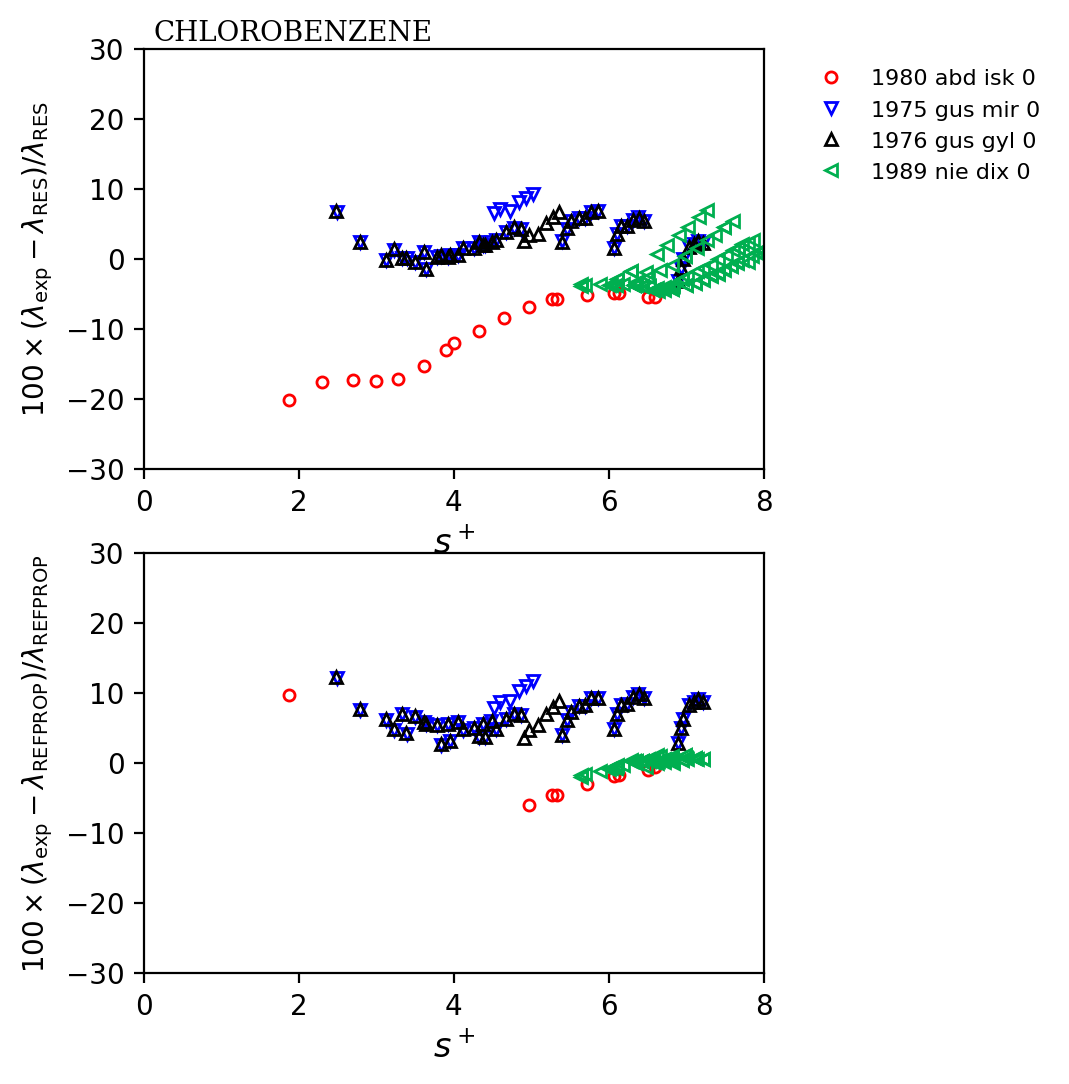

Supplement: Supplementary file 2 — ao4c10815_si_002.zip [file ao4c10815_si_002.zip › Supporting Information/Fig. TC2 - relative deviation - analyzable data - YFR EoS/CHLOROBENZENE.png]

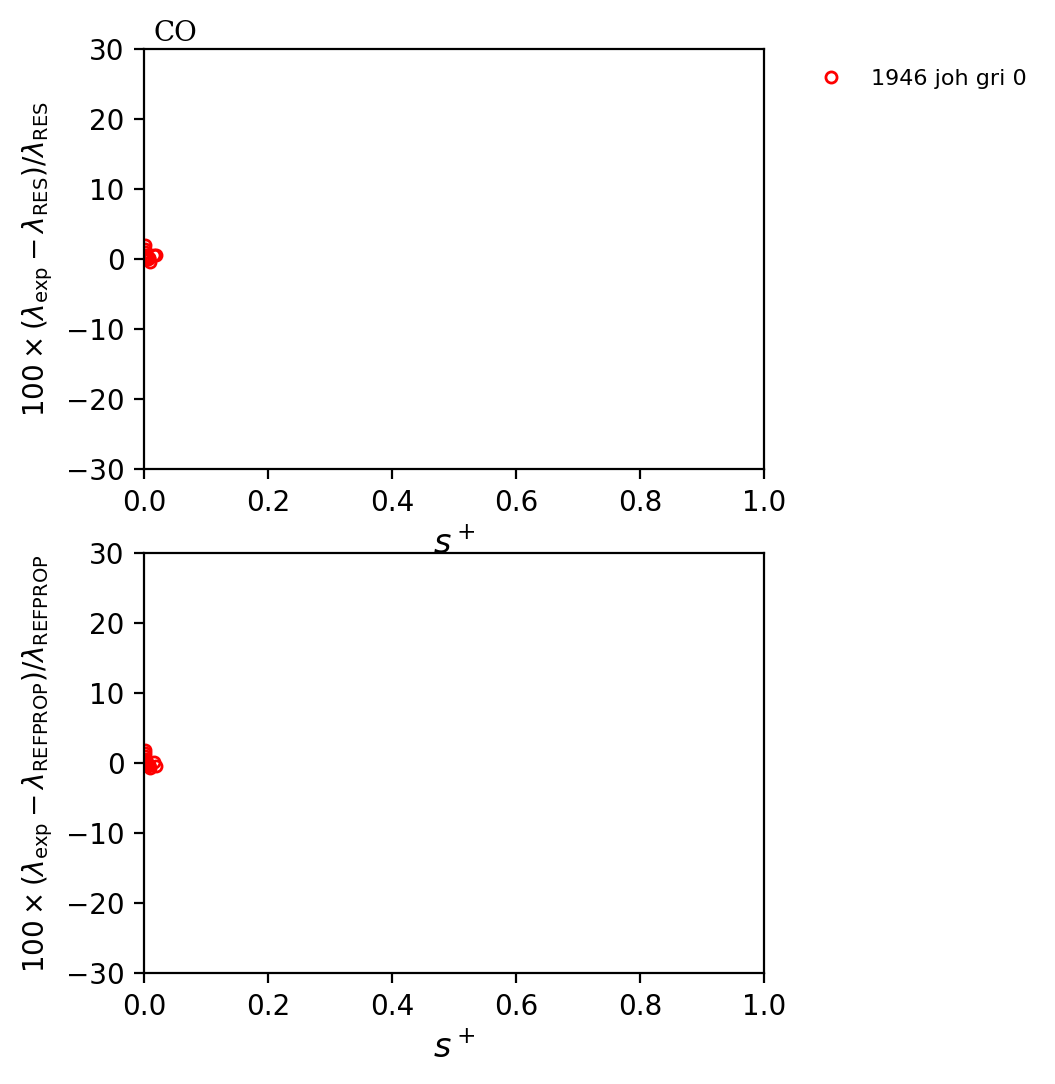

Supplement: Supplementary file 2 — ao4c10815_si_002.zip [file ao4c10815_si_002.zip › Supporting Information/Fig. TC2 - relative deviation - analyzable data - YFR EoS/CO.png]

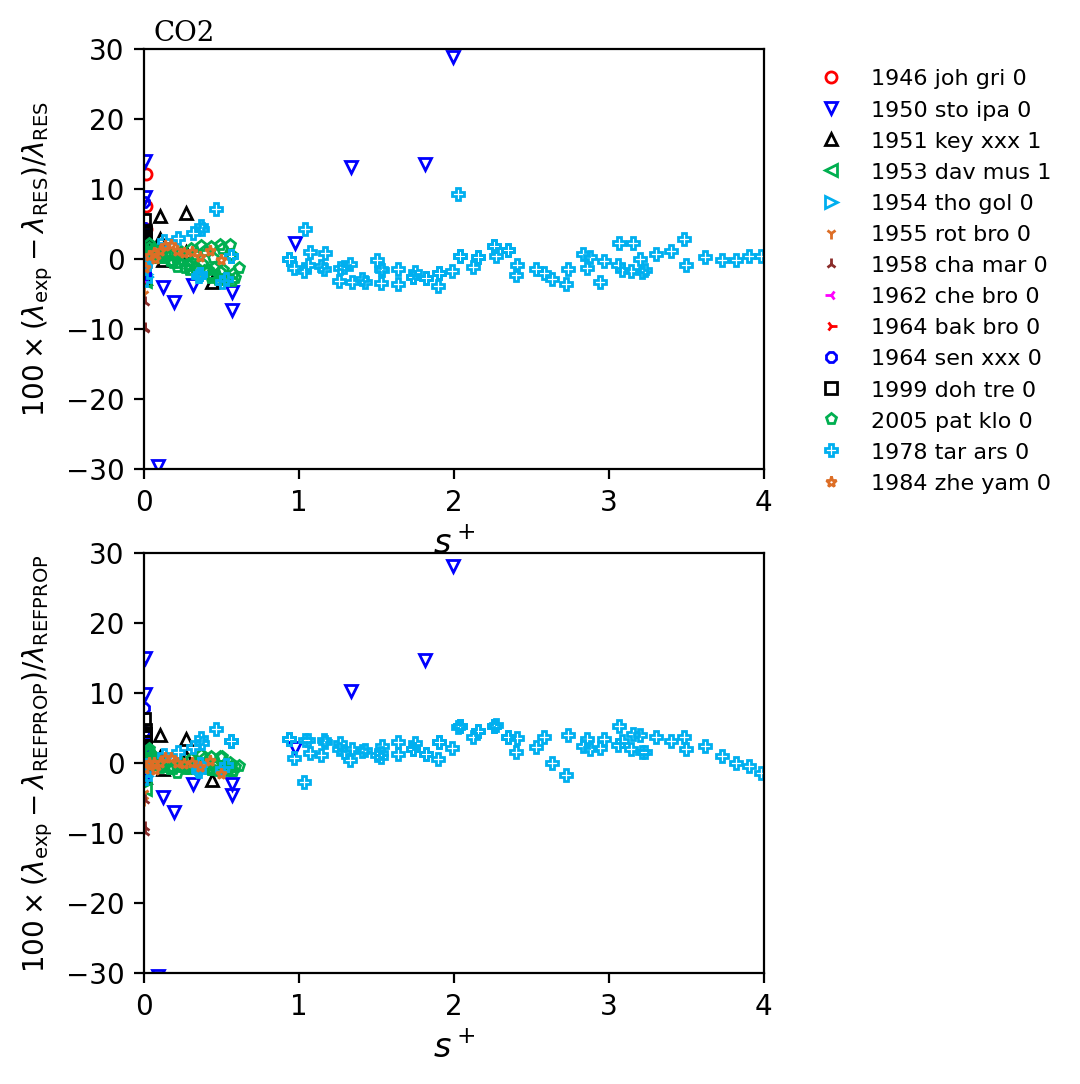

Supplement: Supplementary file 2 — ao4c10815_si_002.zip [file ao4c10815_si_002.zip › Supporting Information/Fig. TC2 - relative deviation - analyzable data - YFR EoS/CO2.png]

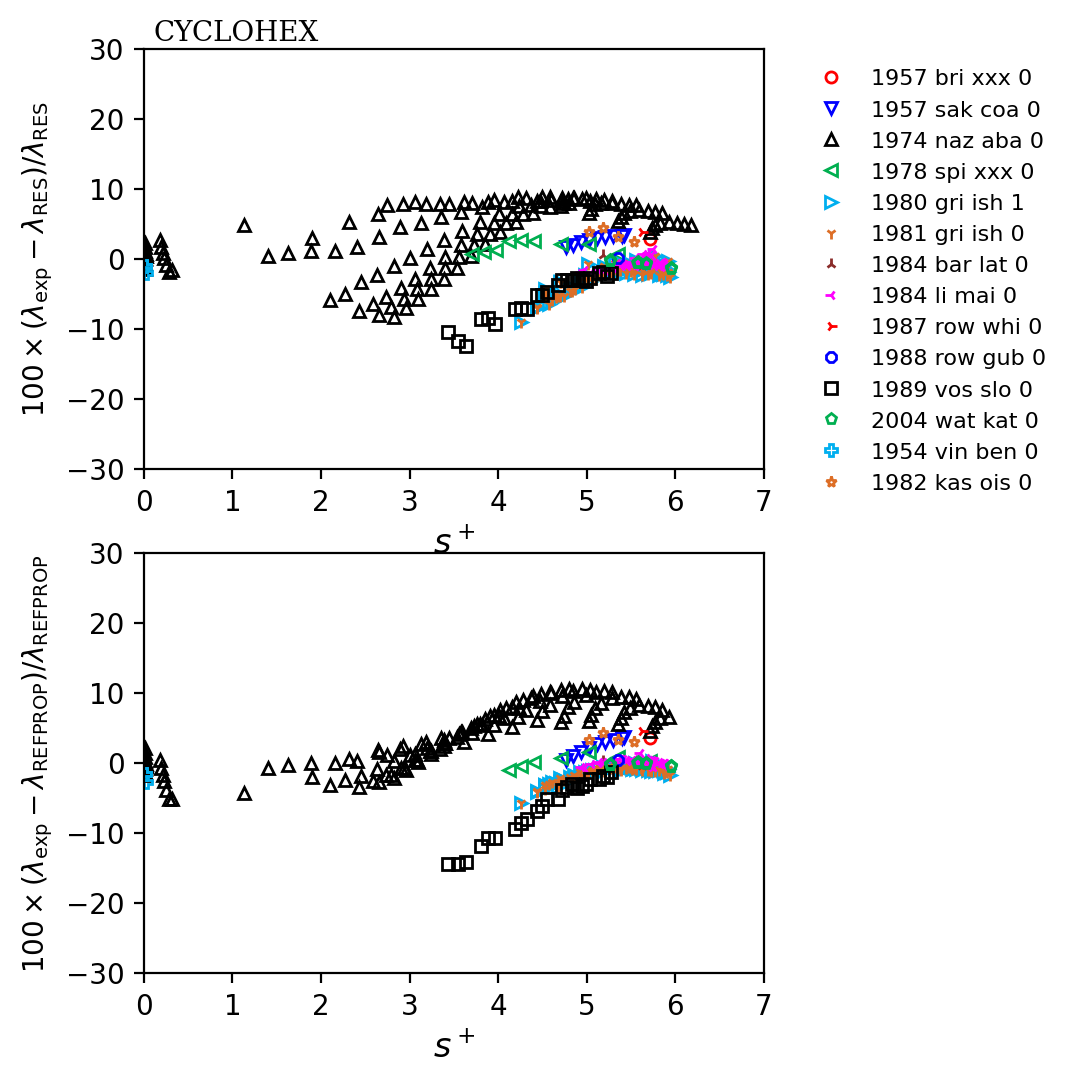

Supplement: Supplementary file 2 — ao4c10815_si_002.zip [file ao4c10815_si_002.zip › Supporting Information/Fig. TC2 - relative deviation - analyzable data - YFR EoS/CYCLOHEX.png]

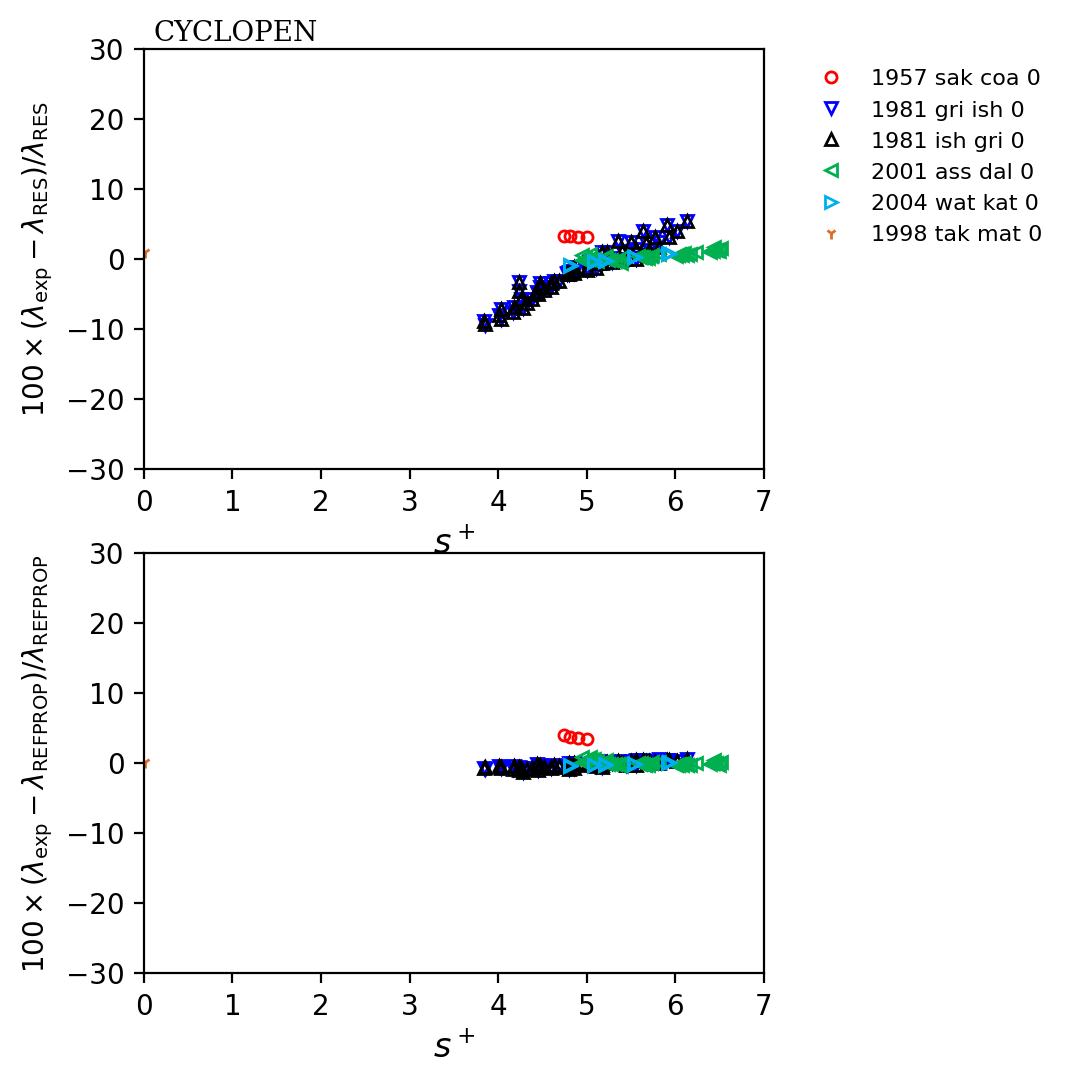

Supplement: Supplementary file 2 — ao4c10815_si_002.zip [file ao4c10815_si_002.zip › Supporting Information/Fig. TC2 - relative deviation - analyzable data - YFR EoS/CYCLOPEN.png]

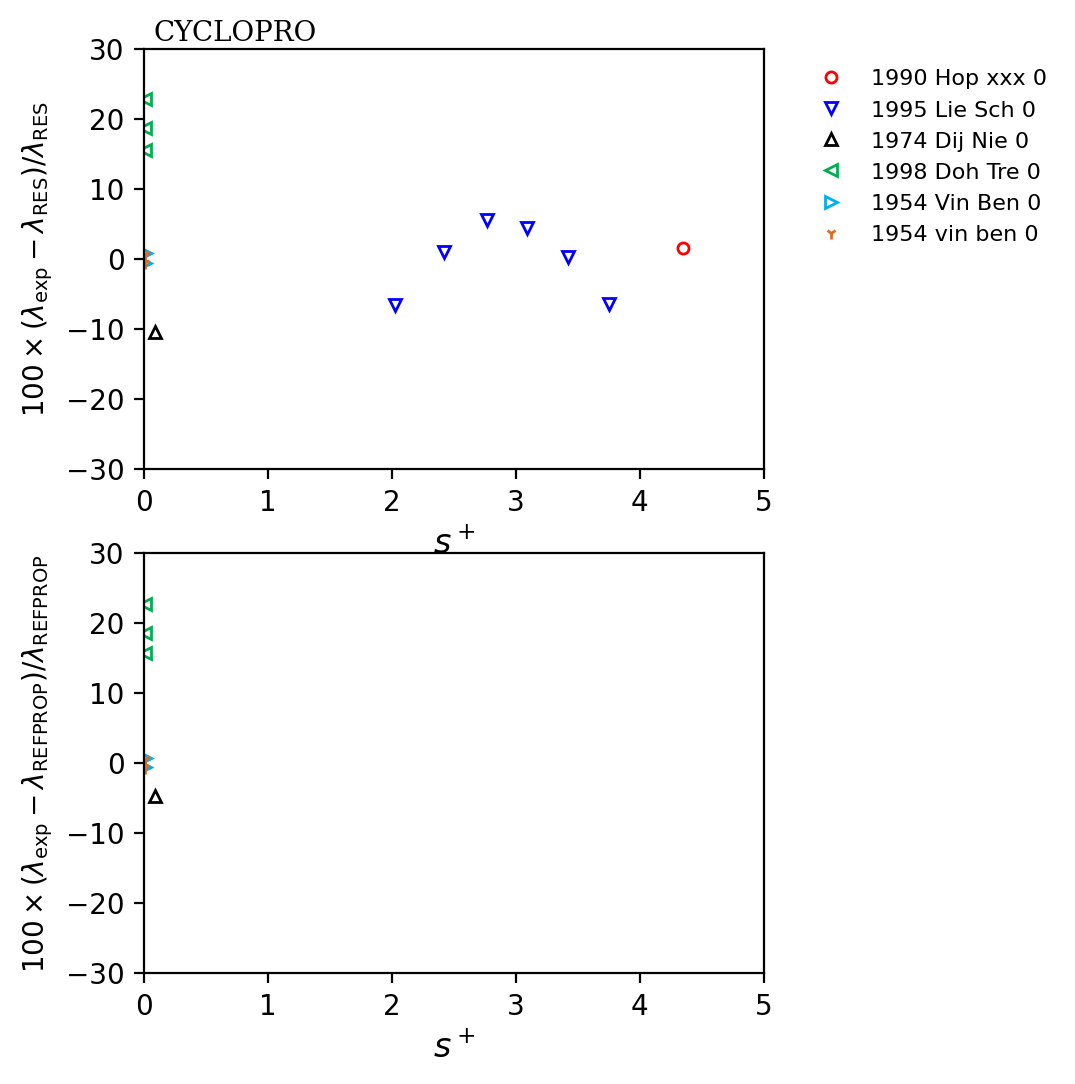

Supplement: Supplementary file 2 — ao4c10815_si_002.zip [file ao4c10815_si_002.zip › Supporting Information/Fig. TC2 - relative deviation - analyzable data - YFR EoS/CYCLOPRO.png]

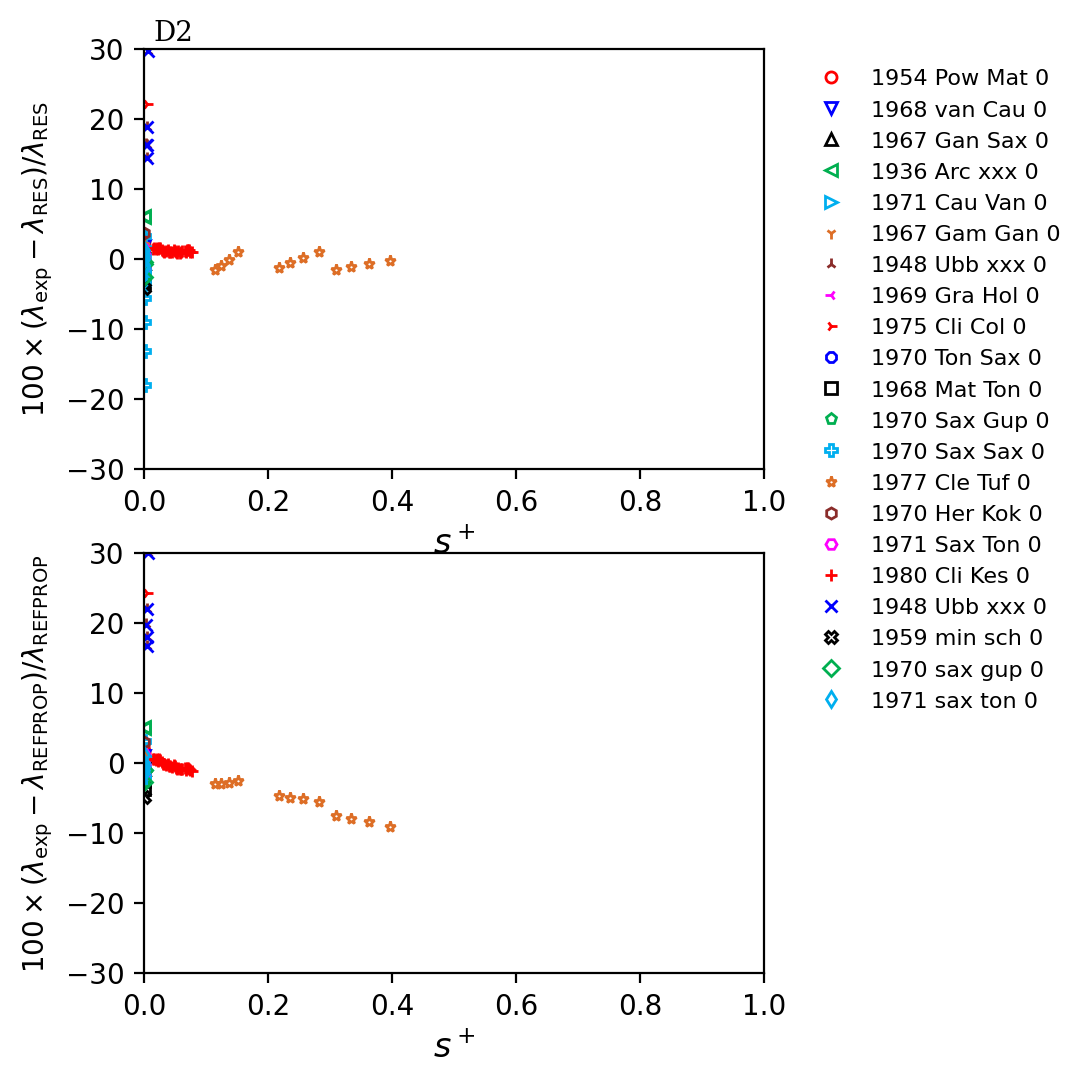

Supplement: Supplementary file 2 — ao4c10815_si_002.zip [file ao4c10815_si_002.zip › Supporting Information/Fig. TC2 - relative deviation - analyzable data - YFR EoS/D2.png]

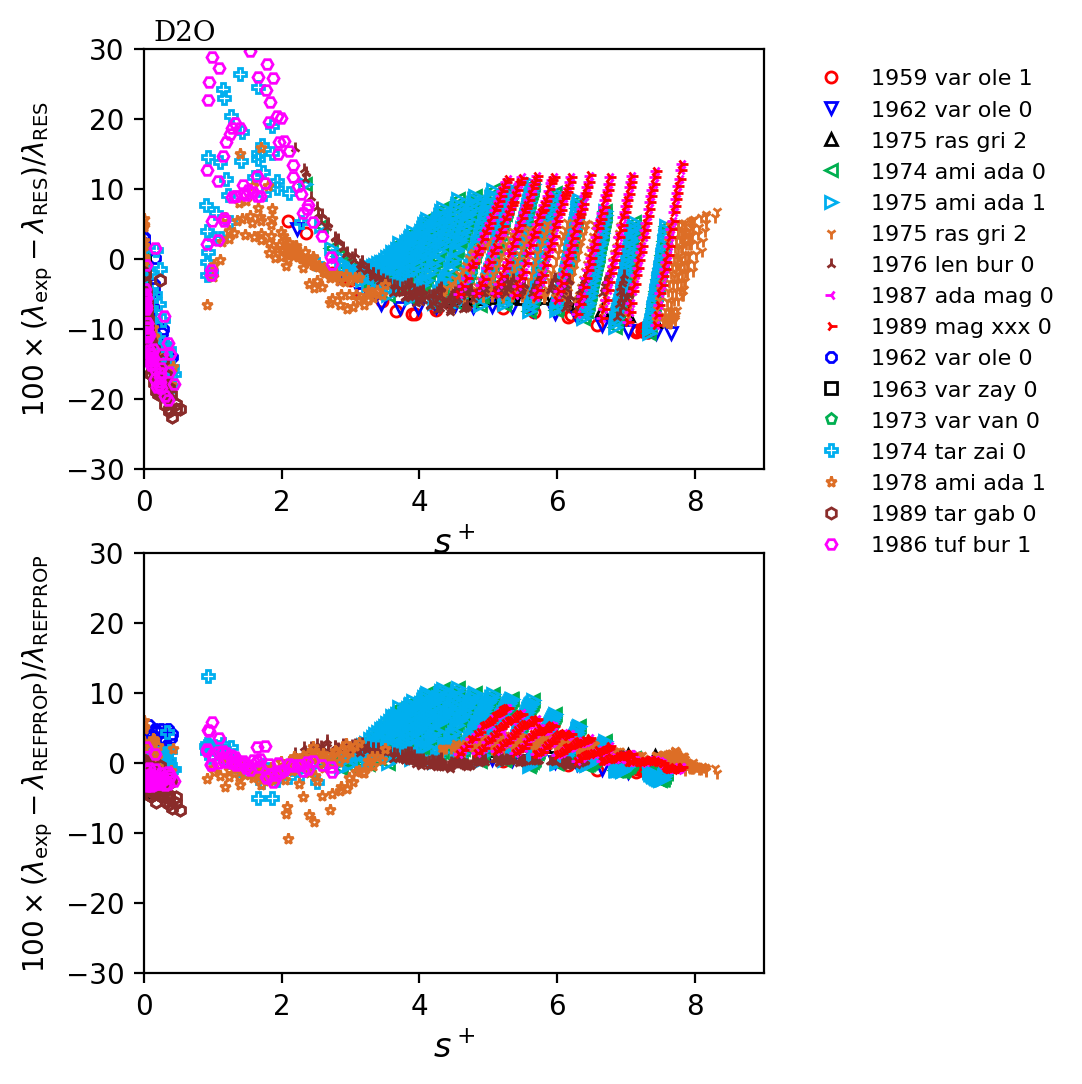

Supplement: Supplementary file 2 — ao4c10815_si_002.zip [file ao4c10815_si_002.zip › Supporting Information/Fig. TC2 - relative deviation - analyzable data - YFR EoS/D2O.png]

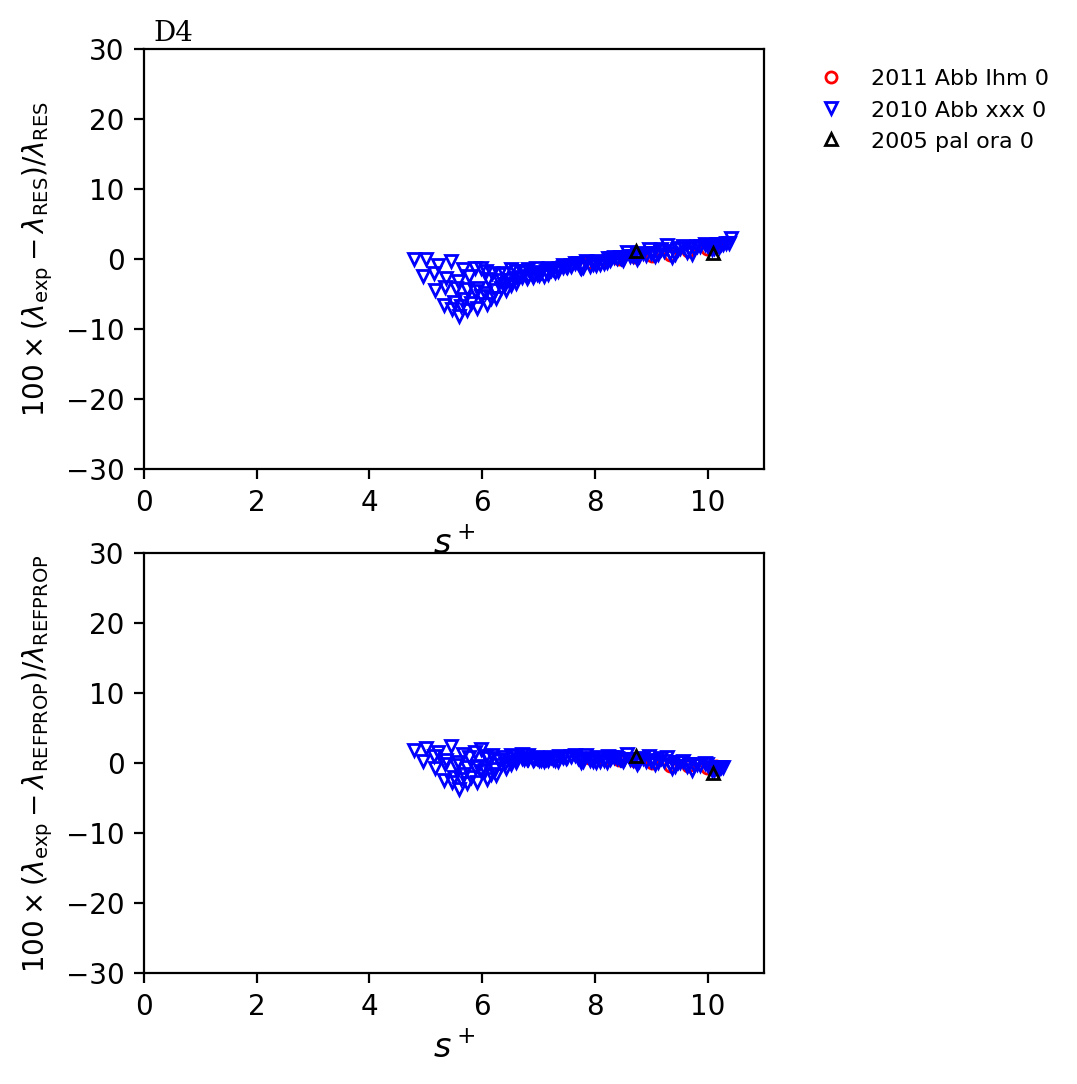

Supplement: Supplementary file 2 — ao4c10815_si_002.zip [file ao4c10815_si_002.zip › Supporting Information/Fig. TC2 - relative deviation - analyzable data - YFR EoS/D4.png]

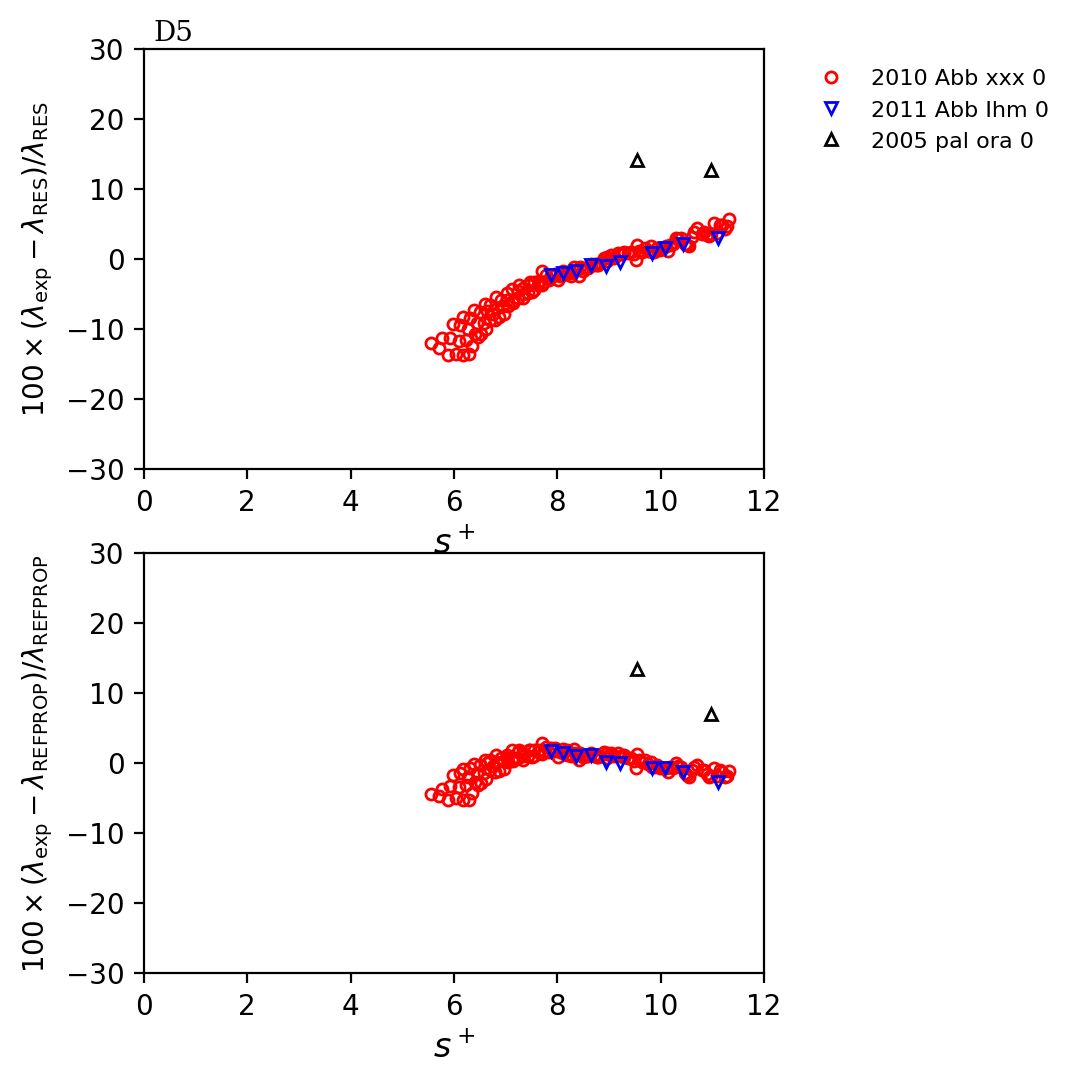

Supplement: Supplementary file 2 — ao4c10815_si_002.zip [file ao4c10815_si_002.zip › Supporting Information/Fig. TC2 - relative deviation - analyzable data - YFR EoS/D5.png]

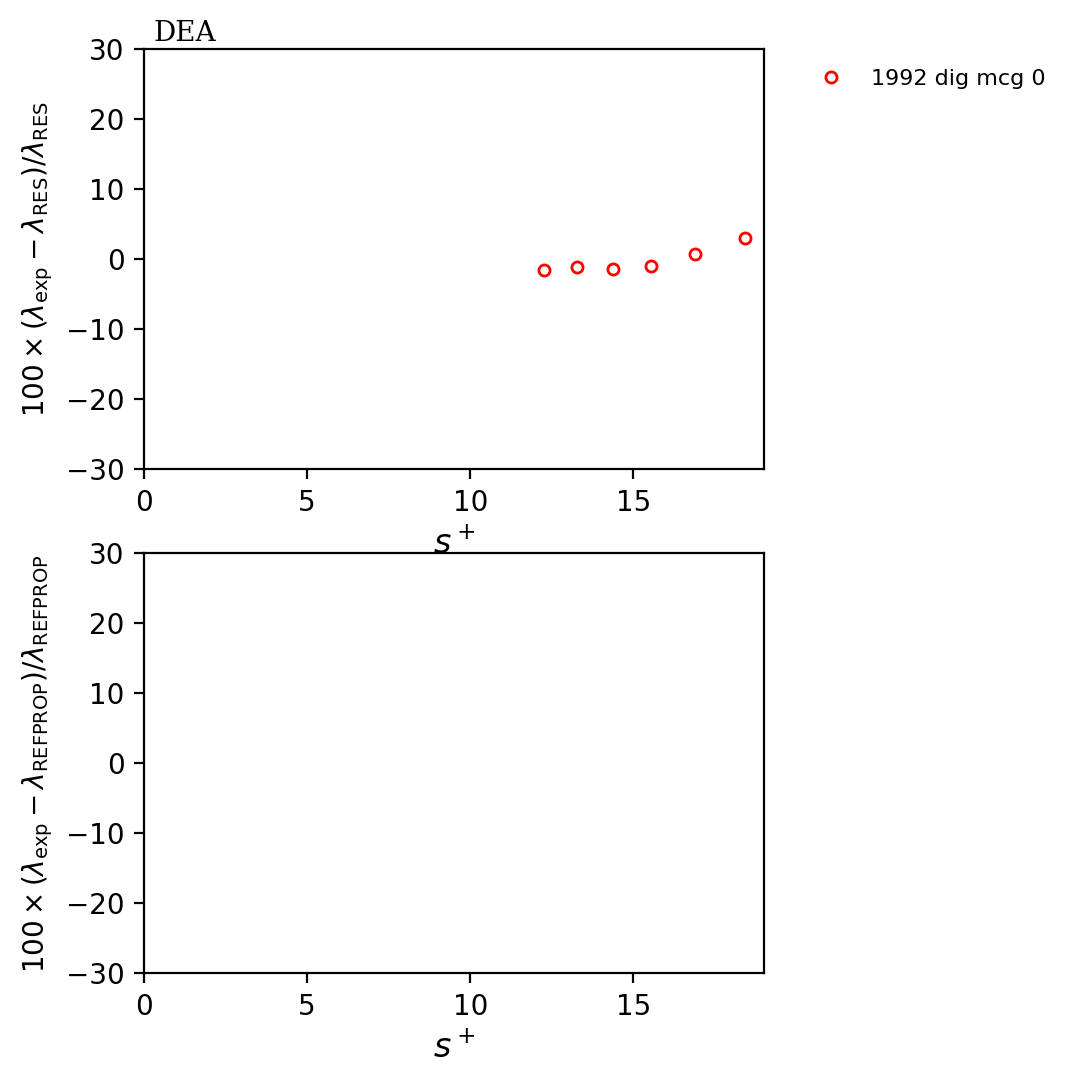

Supplement: Supplementary file 2 — ao4c10815_si_002.zip [file ao4c10815_si_002.zip › Supporting Information/Fig. TC2 - relative deviation - analyzable data - YFR EoS/DEA.png]

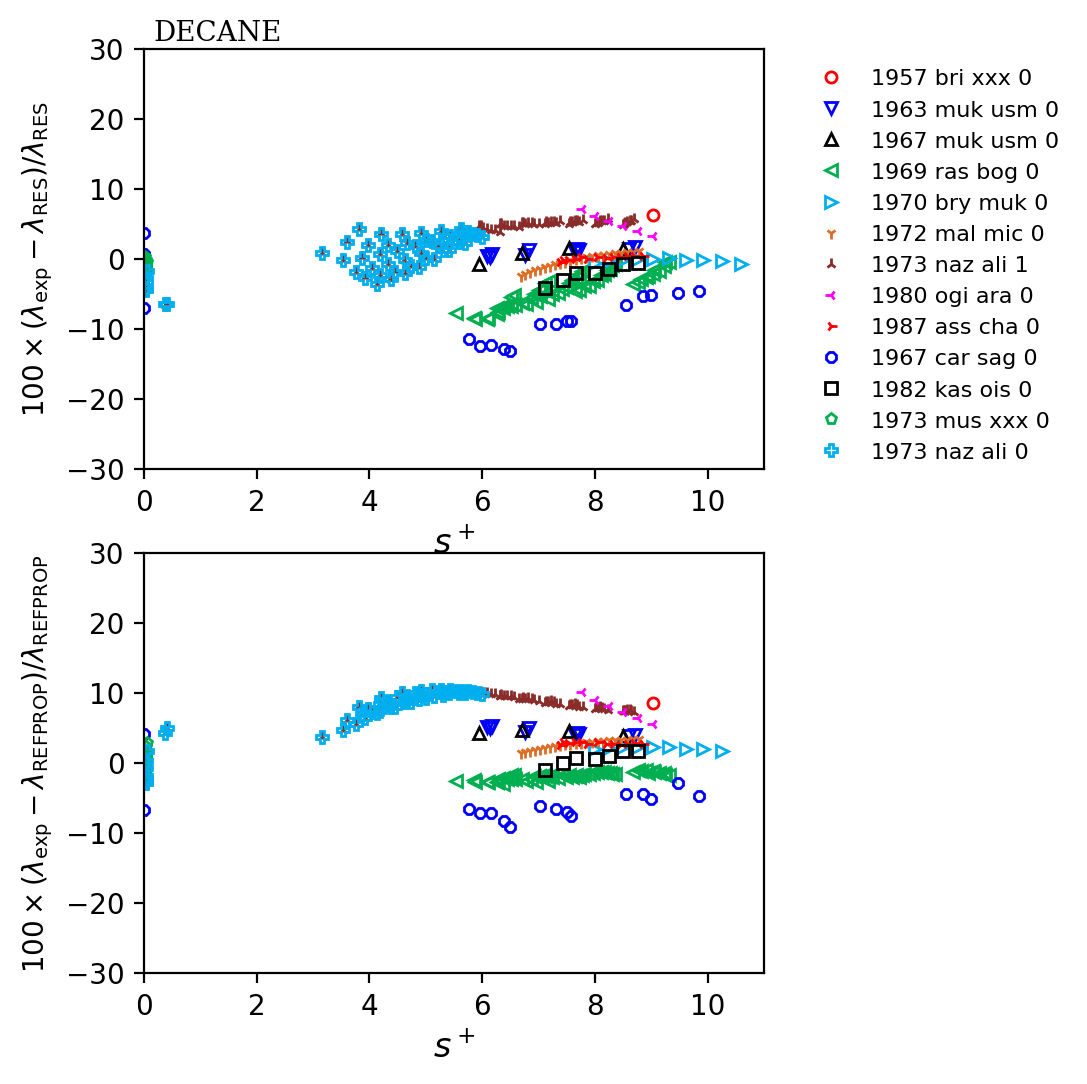

Supplement: Supplementary file 2 — ao4c10815_si_002.zip [file ao4c10815_si_002.zip › Supporting Information/Fig. TC2 - relative deviation - analyzable data - YFR EoS/DECANE.png]

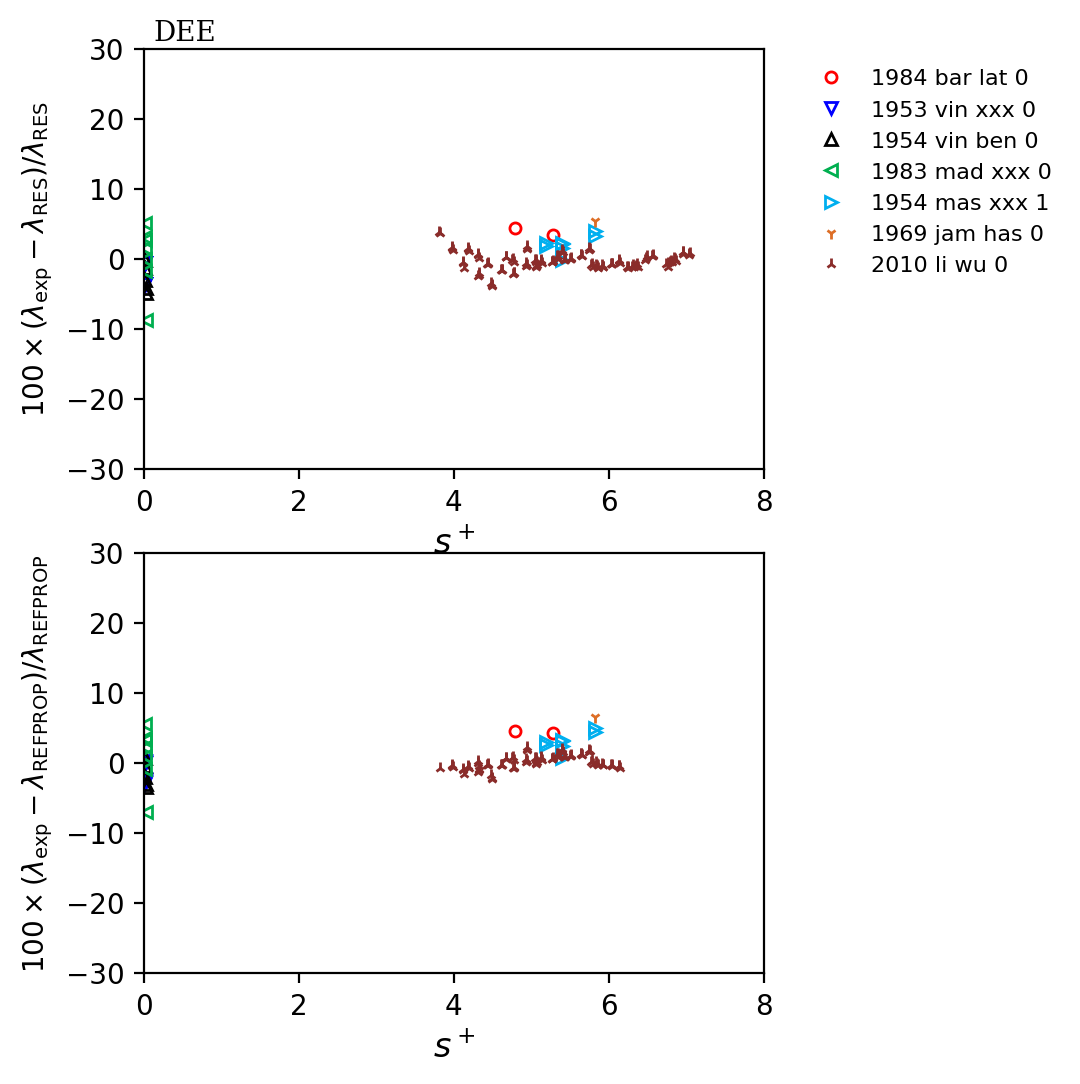

Supplement: Supplementary file 2 — ao4c10815_si_002.zip [file ao4c10815_si_002.zip › Supporting Information/Fig. TC2 - relative deviation - analyzable data - YFR EoS/DEE.png]

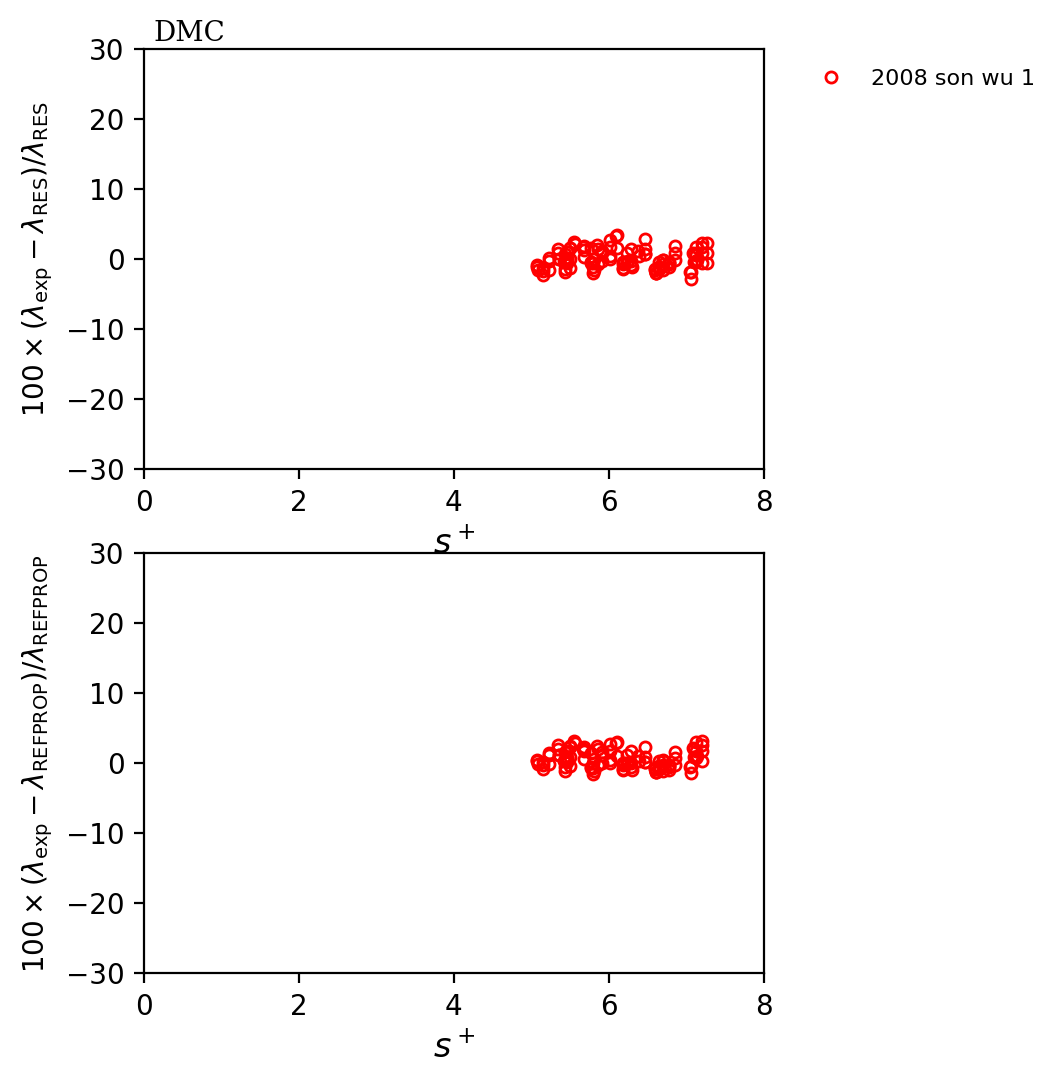

Supplement: Supplementary file 2 — ao4c10815_si_002.zip [file ao4c10815_si_002.zip › Supporting Information/Fig. TC2 - relative deviation - analyzable data - YFR EoS/DMC.png]

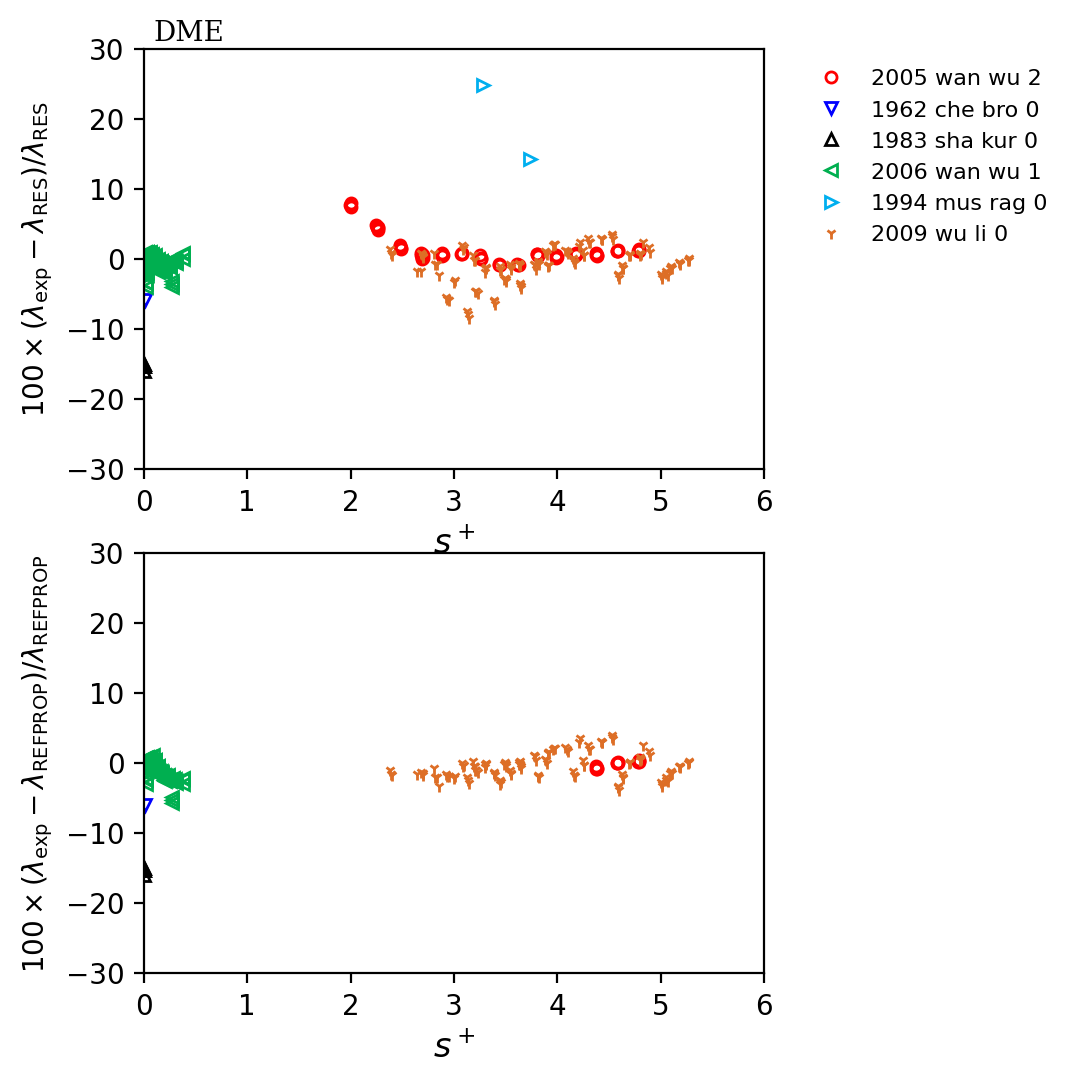

Supplement: Supplementary file 2 — ao4c10815_si_002.zip [file ao4c10815_si_002.zip › Supporting Information/Fig. TC2 - relative deviation - analyzable data - YFR EoS/DME.png]

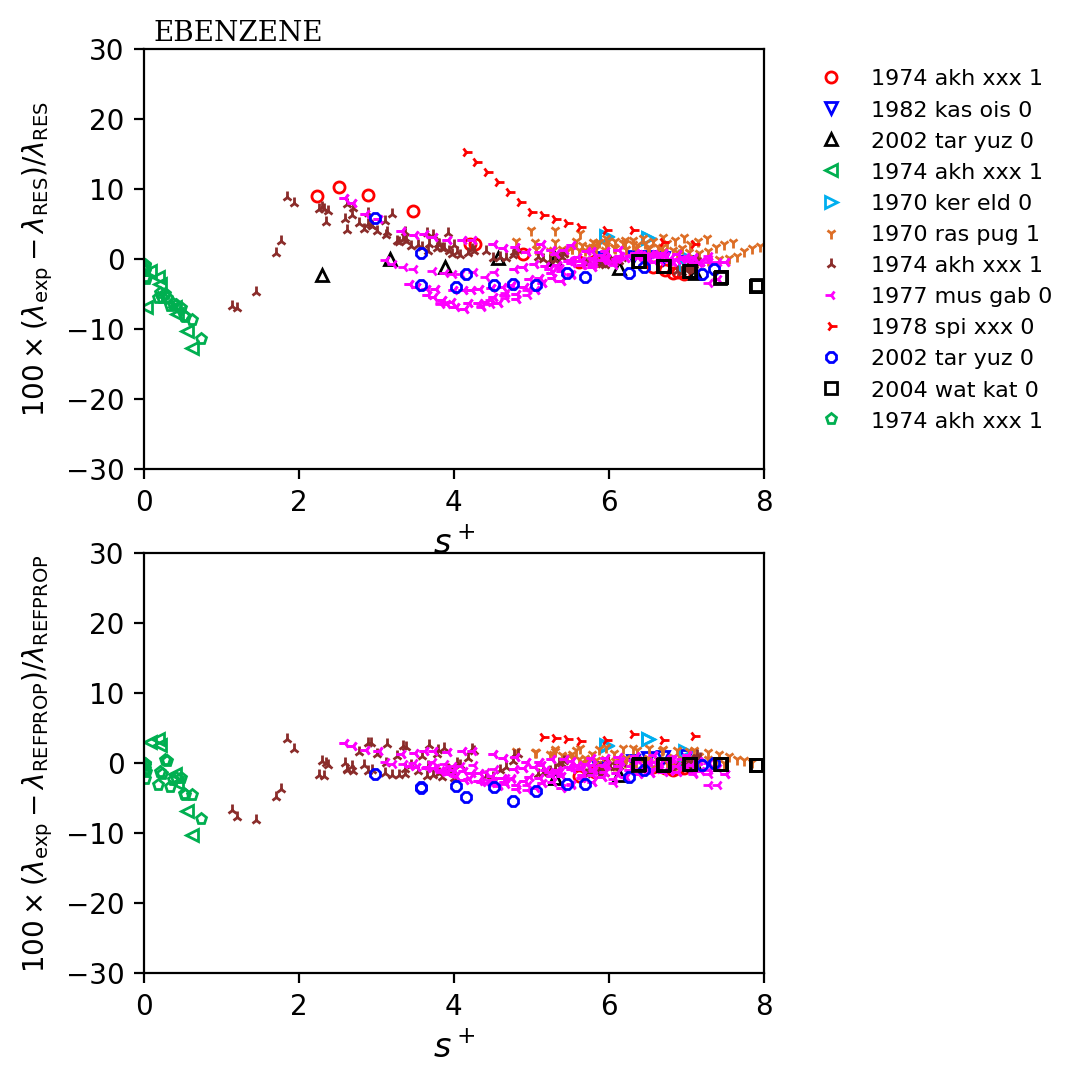

Supplement: Supplementary file 2 — ao4c10815_si_002.zip [file ao4c10815_si_002.zip › Supporting Information/Fig. TC2 - relative deviation - analyzable data - YFR EoS/EBENZENE.png]

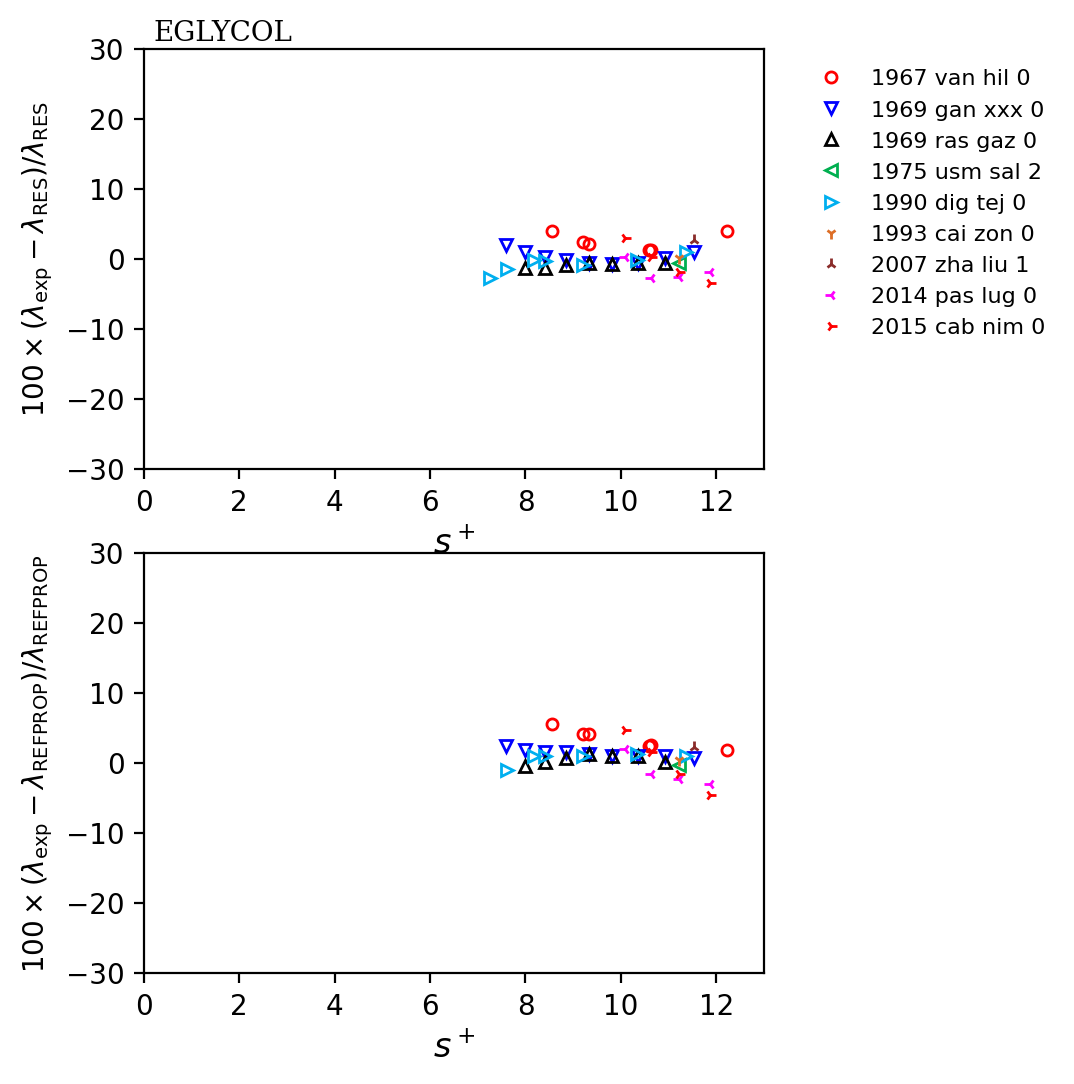

Supplement: Supplementary file 2 — ao4c10815_si_002.zip [file ao4c10815_si_002.zip › Supporting Information/Fig. TC2 - relative deviation - analyzable data - YFR EoS/EGLYCOL.png]

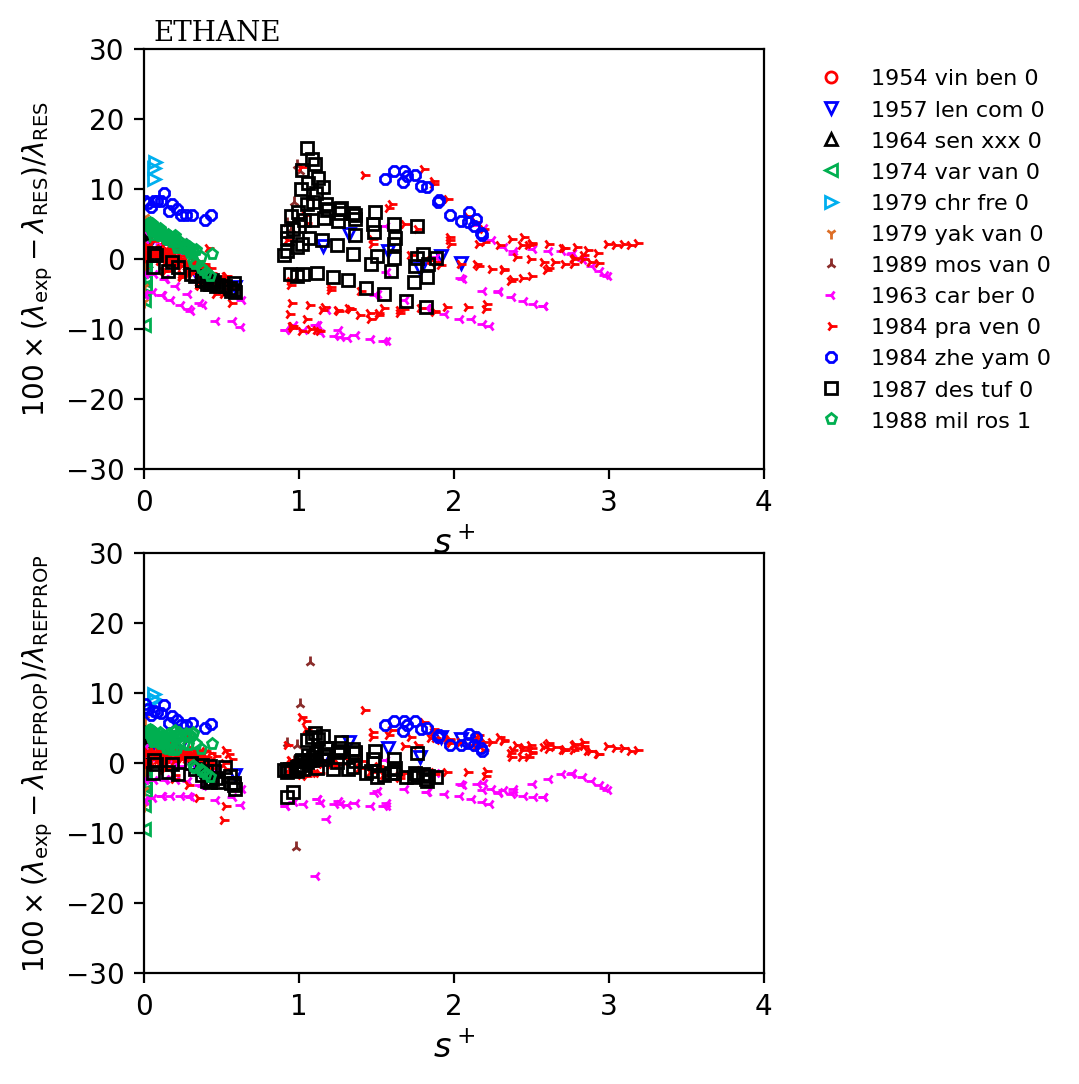

Supplement: Supplementary file 2 — ao4c10815_si_002.zip [file ao4c10815_si_002.zip › Supporting Information/Fig. TC2 - relative deviation - analyzable data - YFR EoS/ETHANE.png]

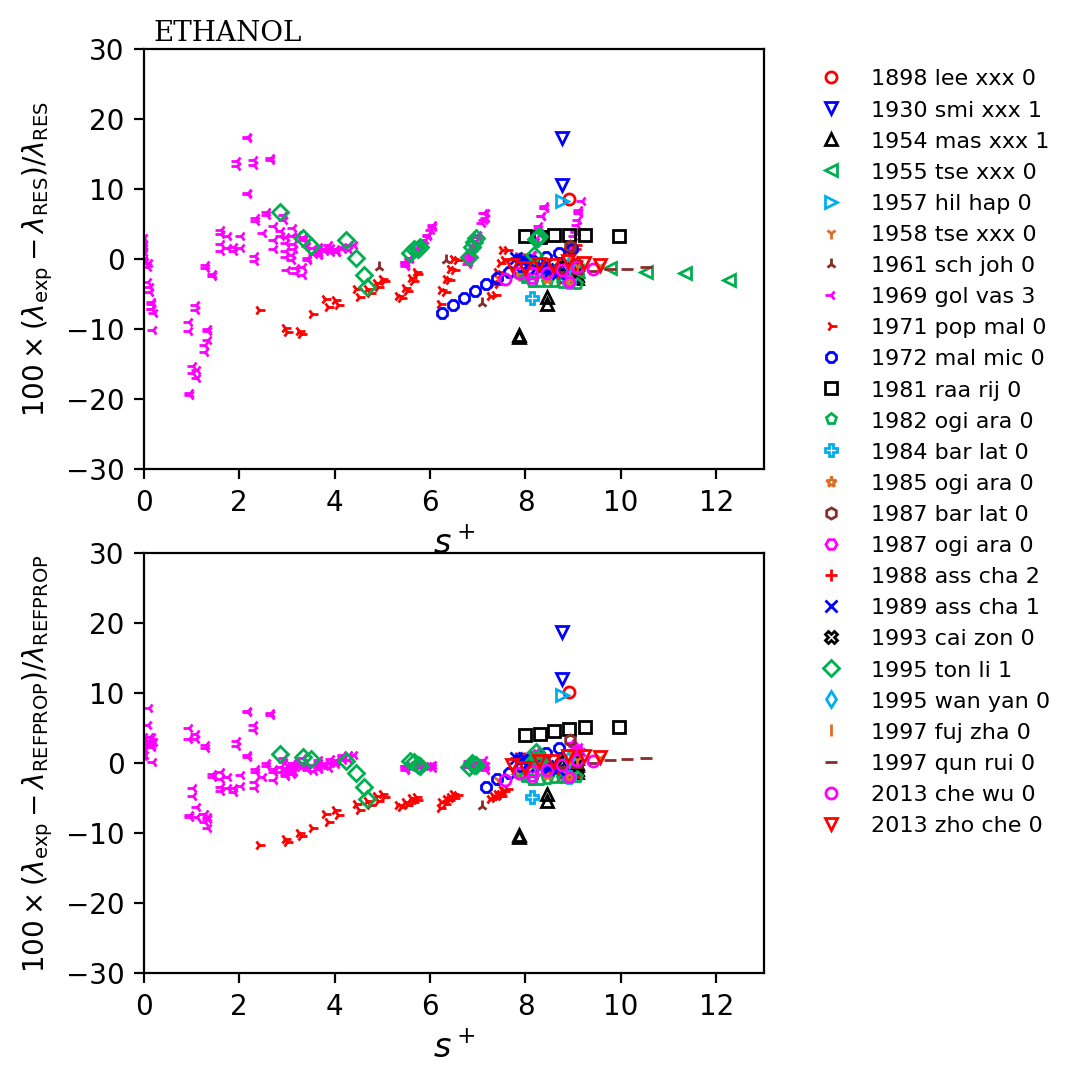

Supplement: Supplementary file 2 — ao4c10815_si_002.zip [file ao4c10815_si_002.zip › Supporting Information/Fig. TC2 - relative deviation - analyzable data - YFR EoS/ETHANOL.png]

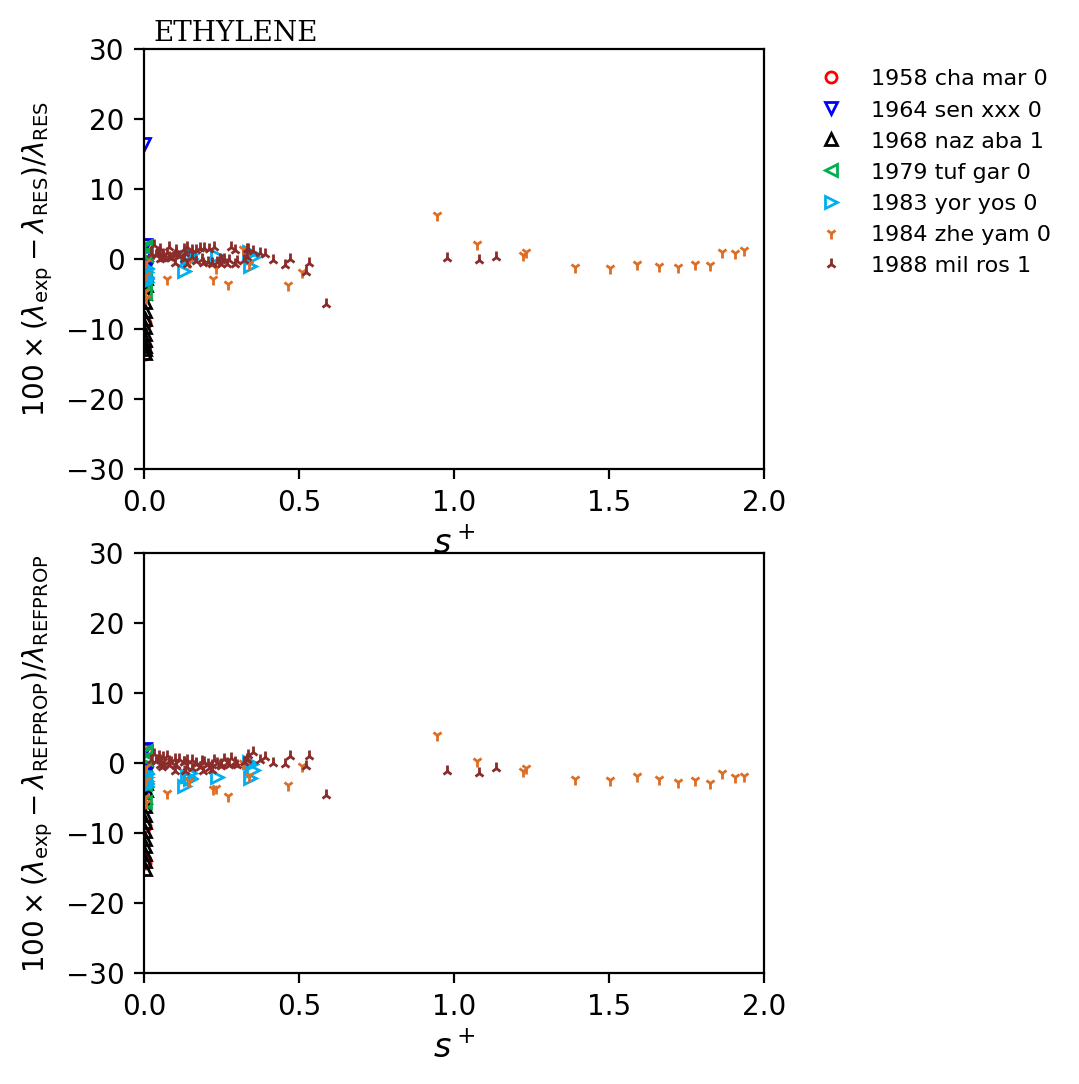

Supplement: Supplementary file 2 — ao4c10815_si_002.zip [file ao4c10815_si_002.zip › Supporting Information/Fig. TC2 - relative deviation - analyzable data - YFR EoS/ETHYLENE.png]

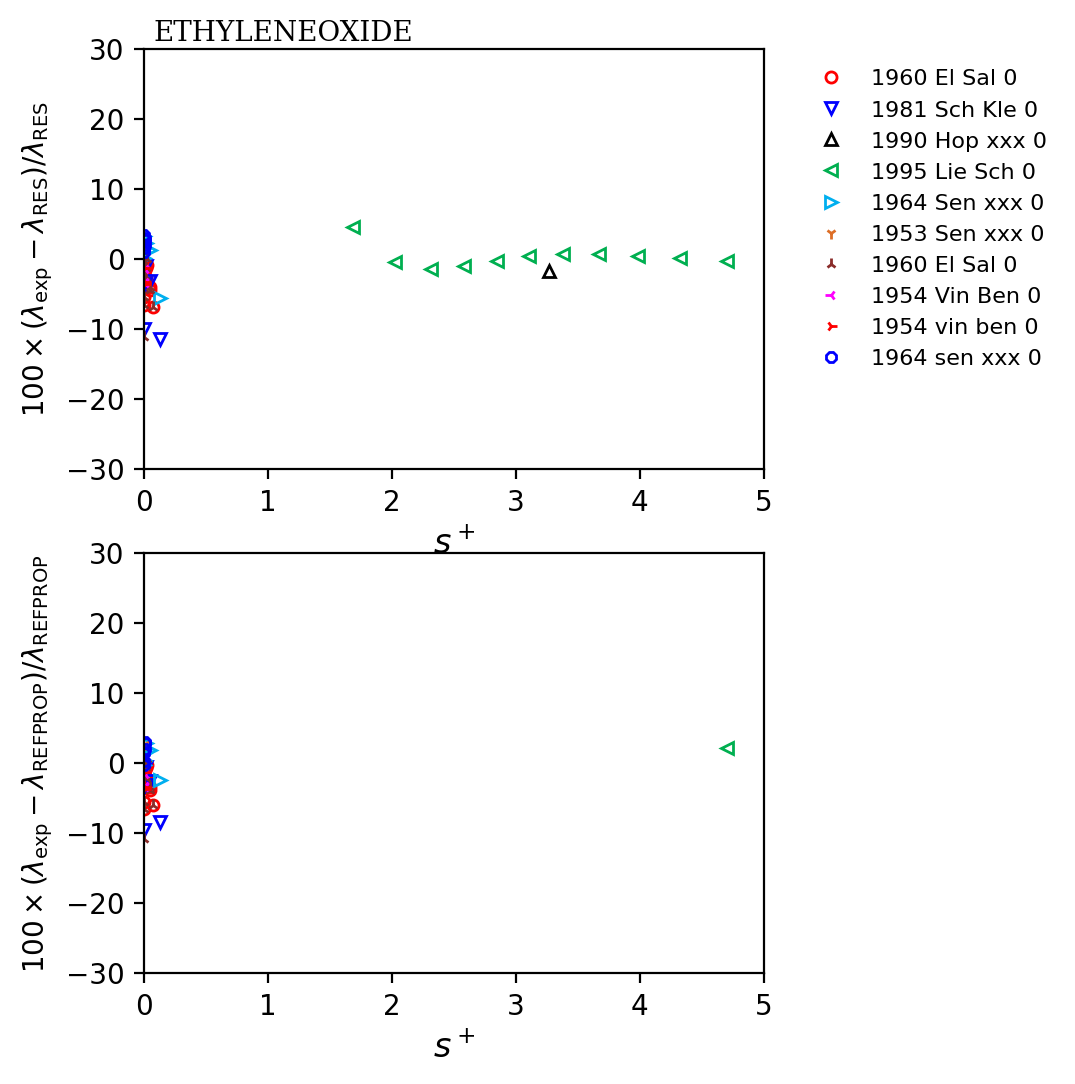

Supplement: Supplementary file 2 — ao4c10815_si_002.zip [file ao4c10815_si_002.zip › Supporting Information/Fig. TC2 - relative deviation - analyzable data - YFR EoS/ETHYLENEOXIDE.png]

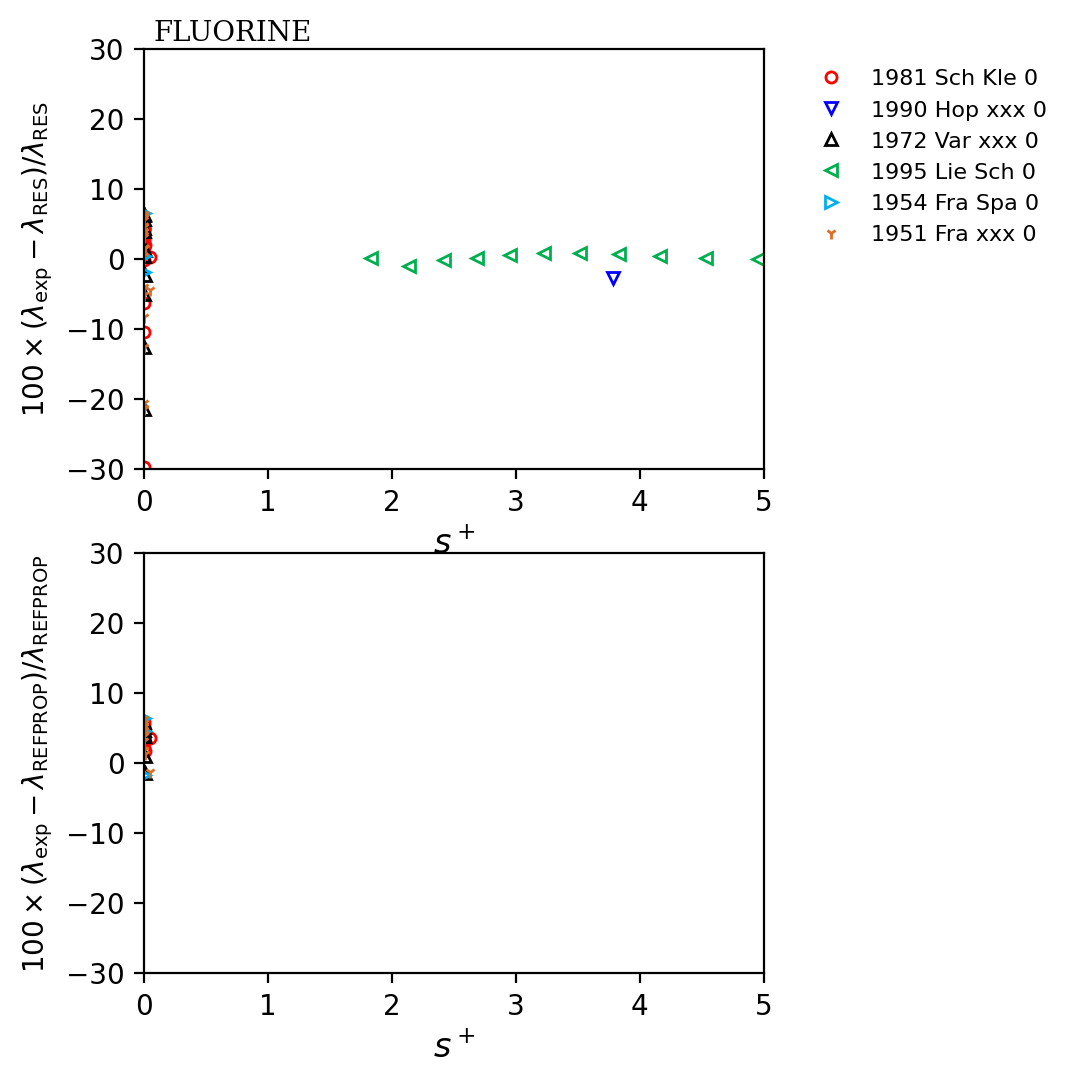

Supplement: Supplementary file 2 — ao4c10815_si_002.zip [file ao4c10815_si_002.zip › Supporting Information/Fig. TC2 - relative deviation - analyzable data - YFR EoS/FLUORINE.png]

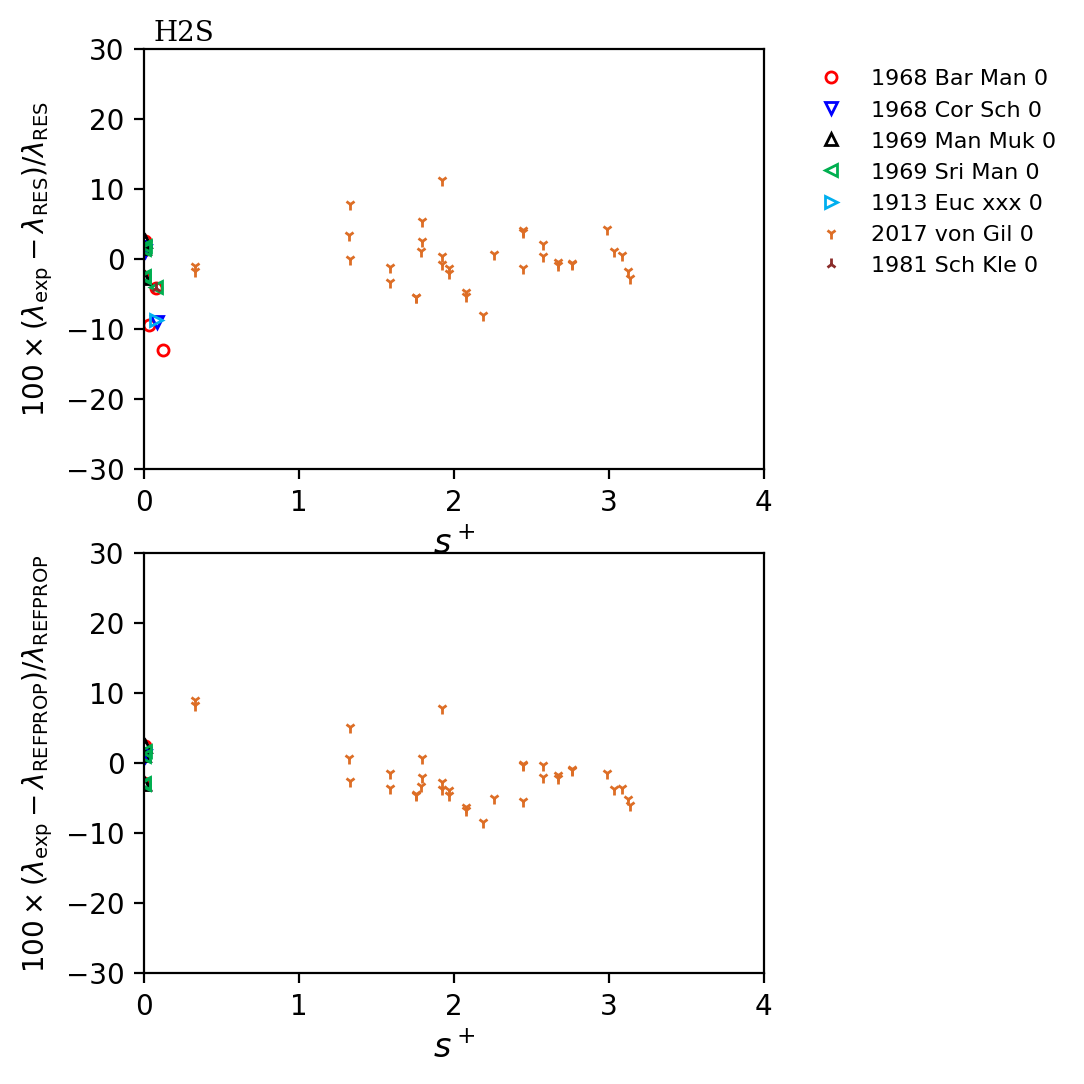

Supplement: Supplementary file 2 — ao4c10815_si_002.zip [file ao4c10815_si_002.zip › Supporting Information/Fig. TC2 - relative deviation - analyzable data - YFR EoS/H2S.png]

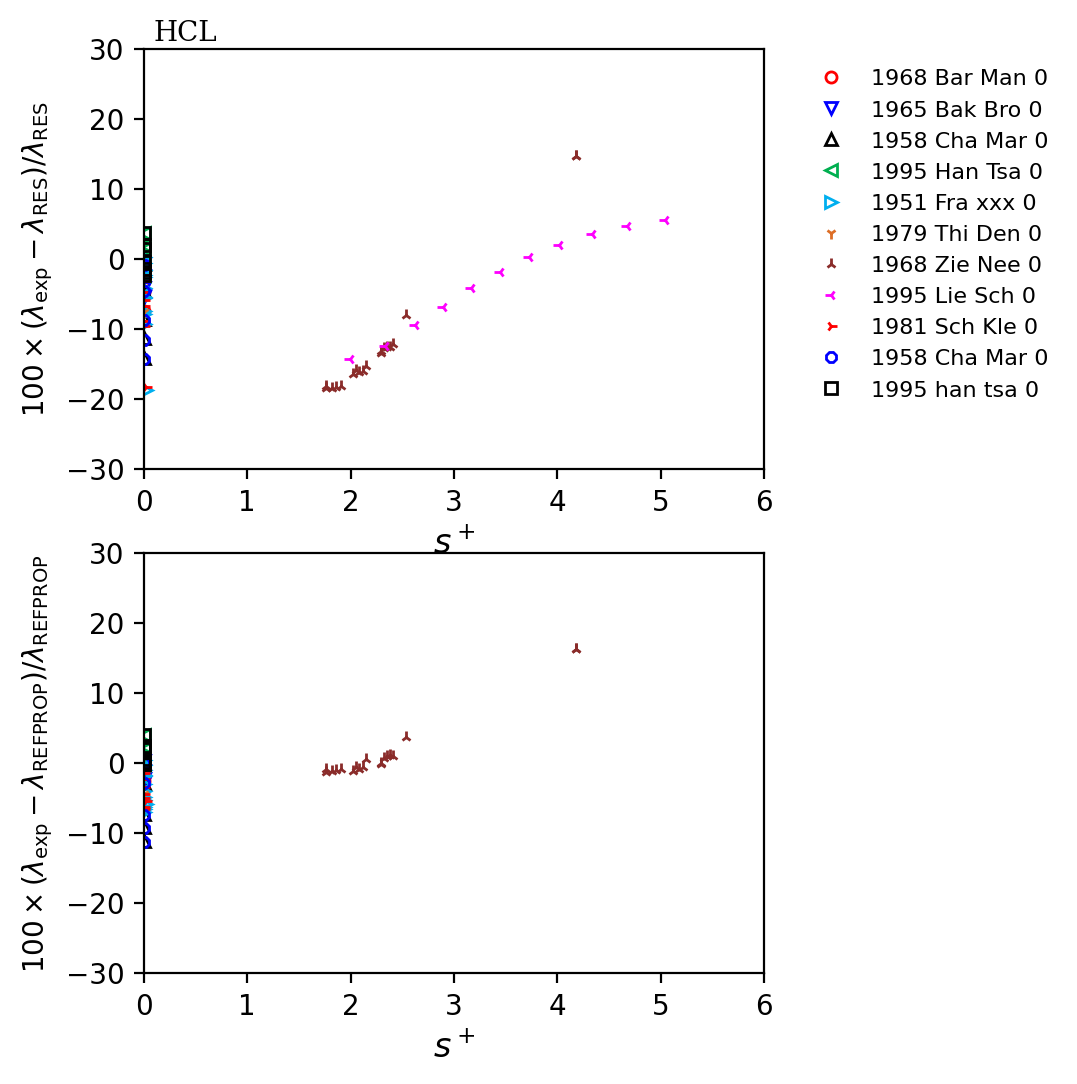

Supplement: Supplementary file 2 — ao4c10815_si_002.zip [file ao4c10815_si_002.zip › Supporting Information/Fig. TC2 - relative deviation - analyzable data - YFR EoS/HCL.png]

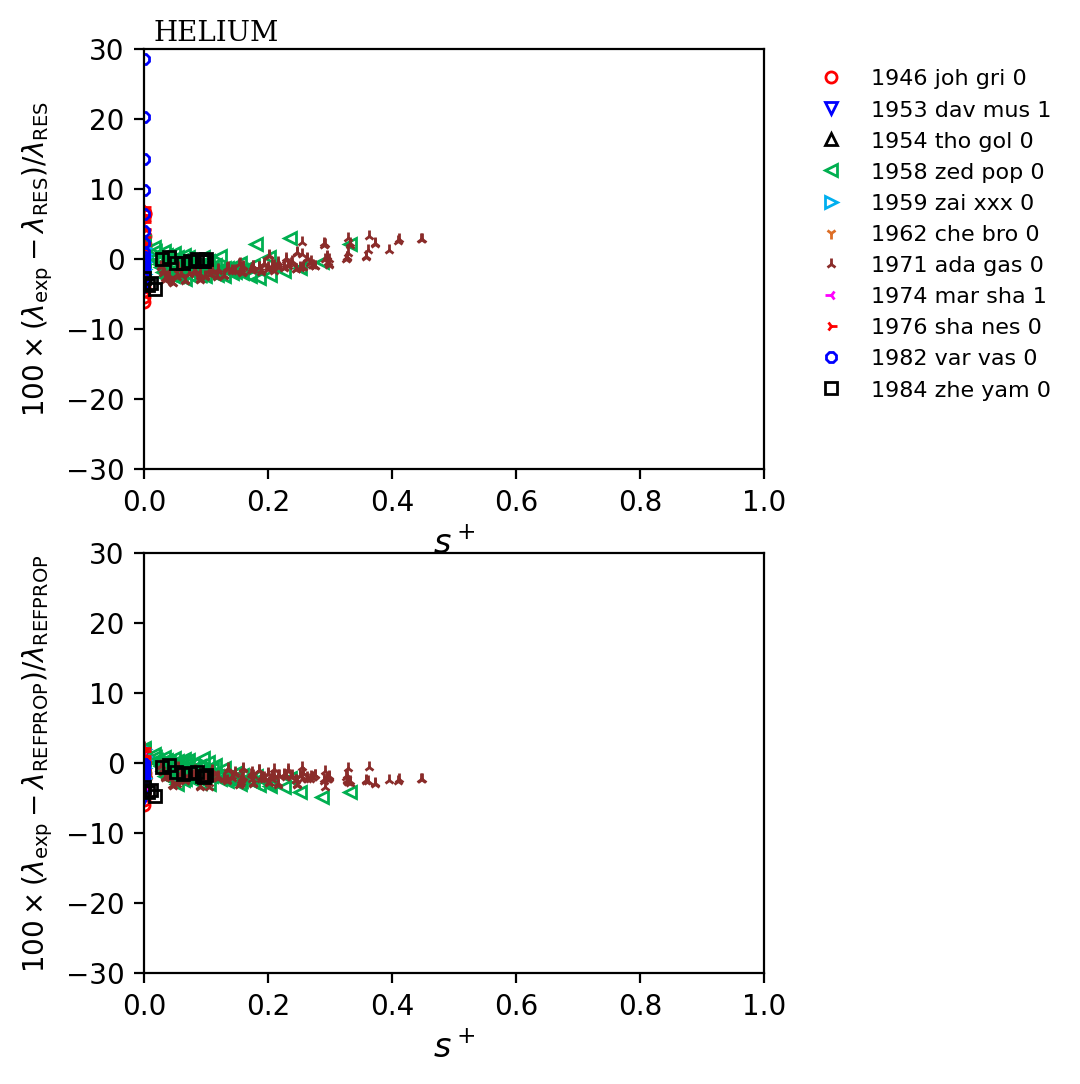

Supplement: Supplementary file 2 — ao4c10815_si_002.zip [file ao4c10815_si_002.zip › Supporting Information/Fig. TC2 - relative deviation - analyzable data - YFR EoS/HELIUM.png]

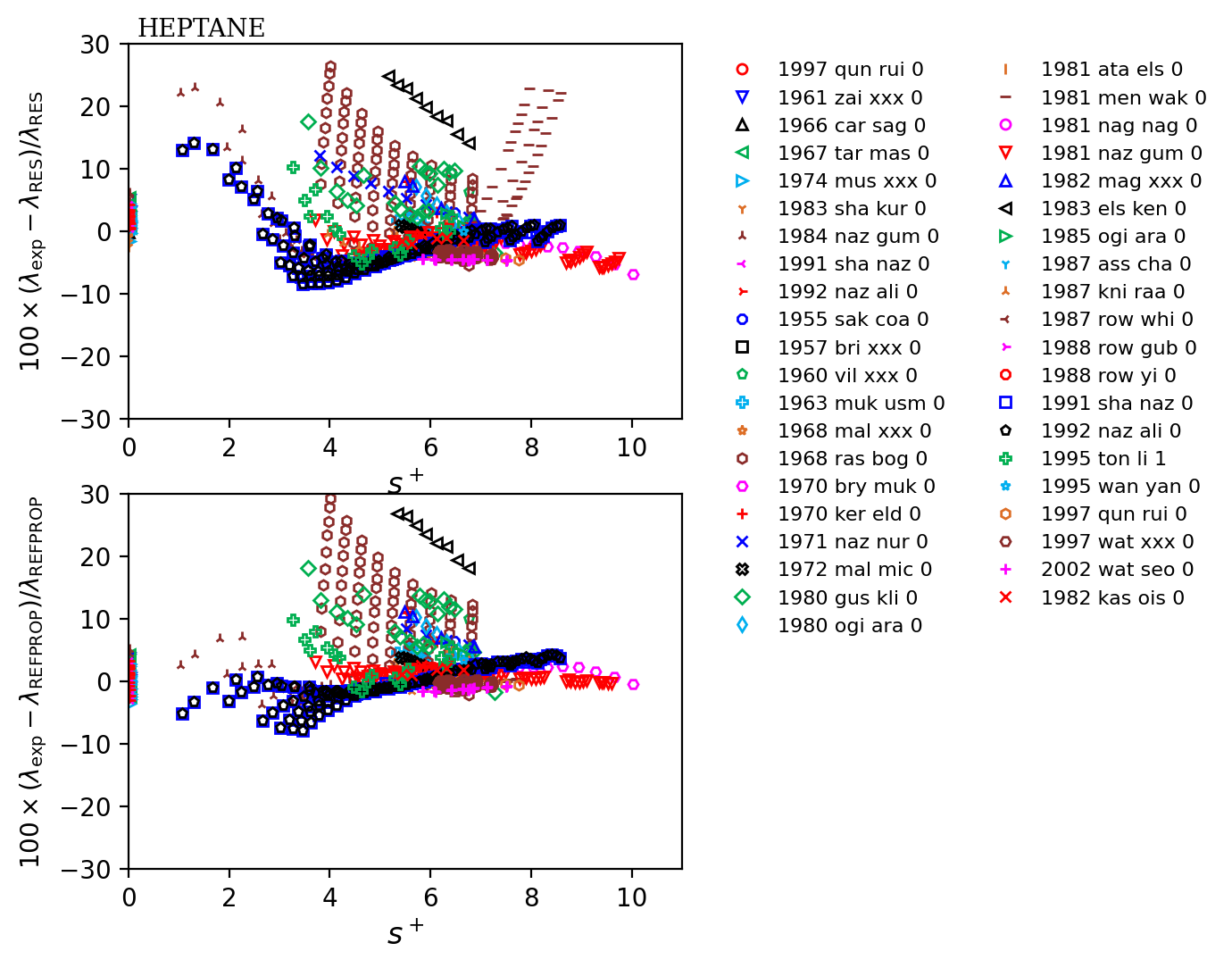

Supplement: Supplementary file 2 — ao4c10815_si_002.zip [file ao4c10815_si_002.zip › Supporting Information/Fig. TC2 - relative deviation - analyzable data - YFR EoS/HEPTANE.png]

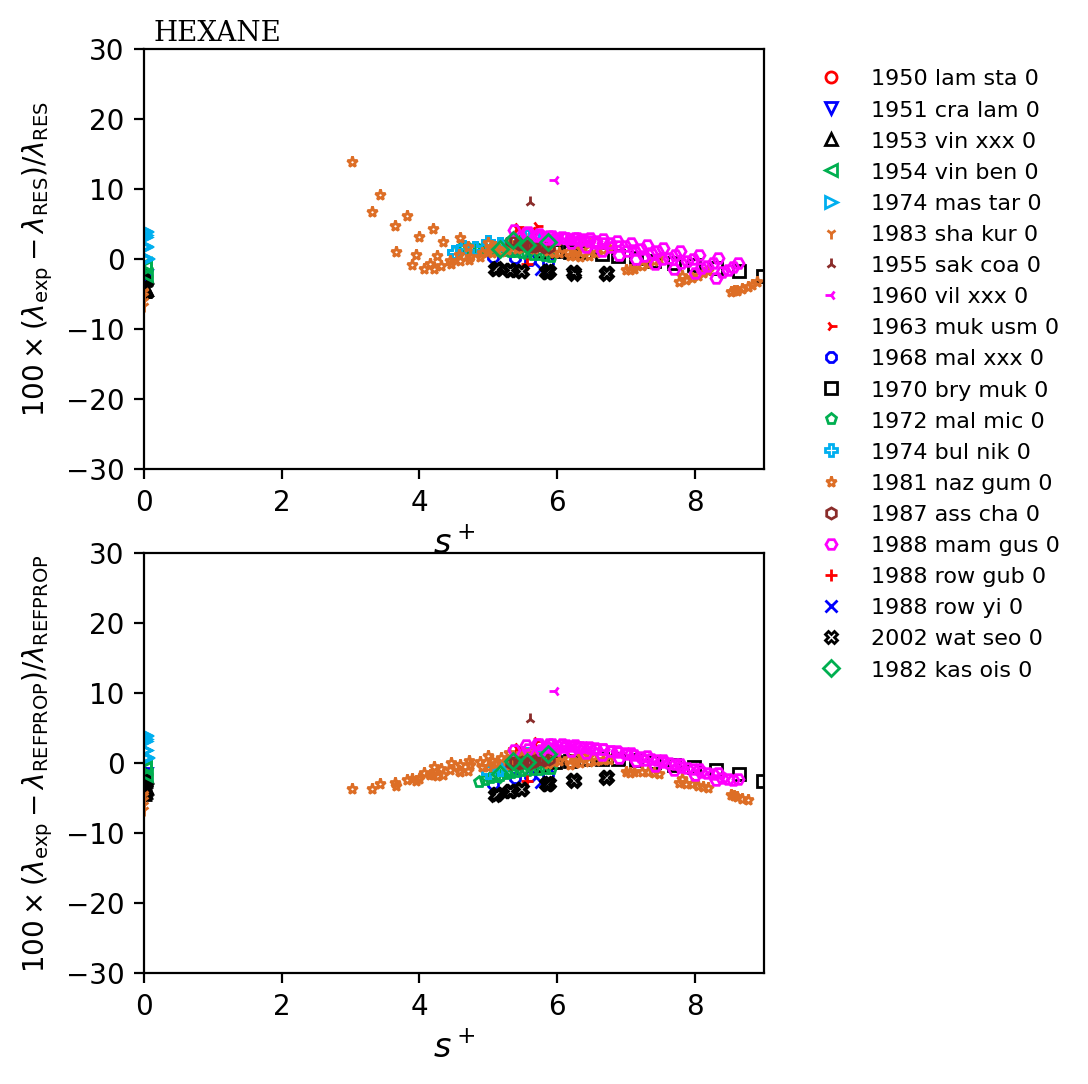

Supplement: Supplementary file 2 — ao4c10815_si_002.zip [file ao4c10815_si_002.zip › Supporting Information/Fig. TC2 - relative deviation - analyzable data - YFR EoS/HEXANE.png]

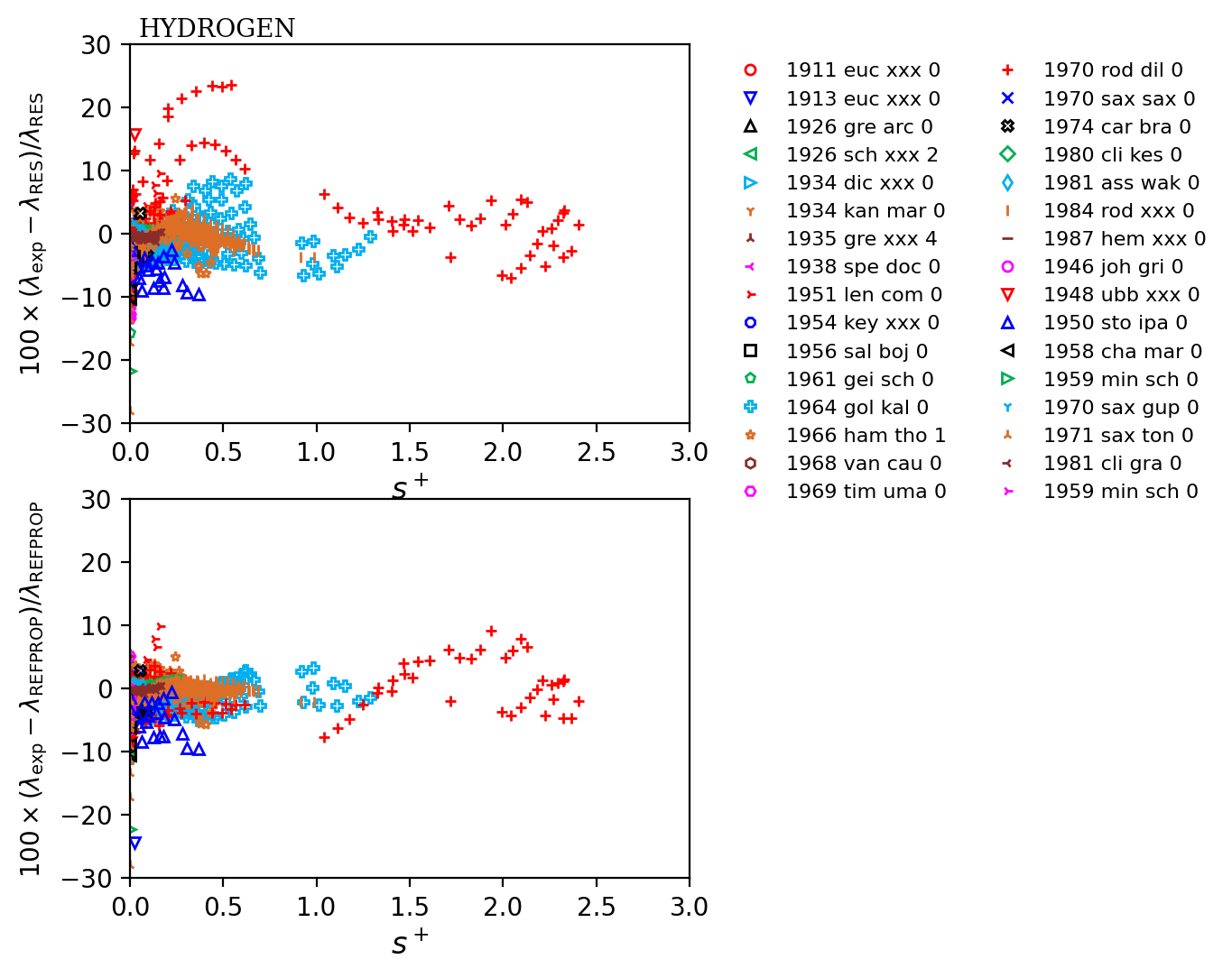

Supplement: Supplementary file 2 — ao4c10815_si_002.zip [file ao4c10815_si_002.zip › Supporting Information/Fig. TC2 - relative deviation - analyzable data - YFR EoS/HYDROGEN.png]

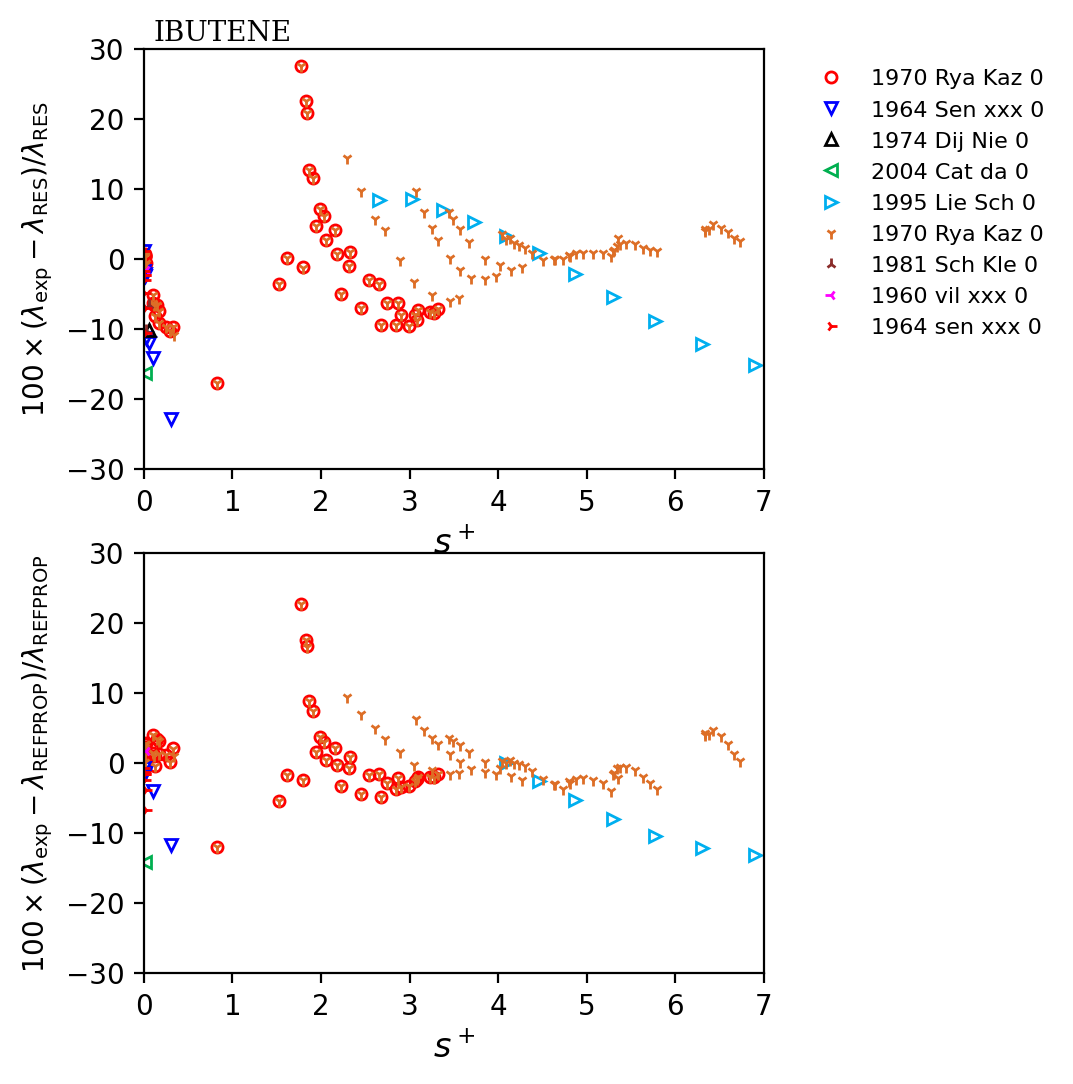

Supplement: Supplementary file 2 — ao4c10815_si_002.zip [file ao4c10815_si_002.zip › Supporting Information/Fig. TC2 - relative deviation - analyzable data - YFR EoS/IBUTENE.png]

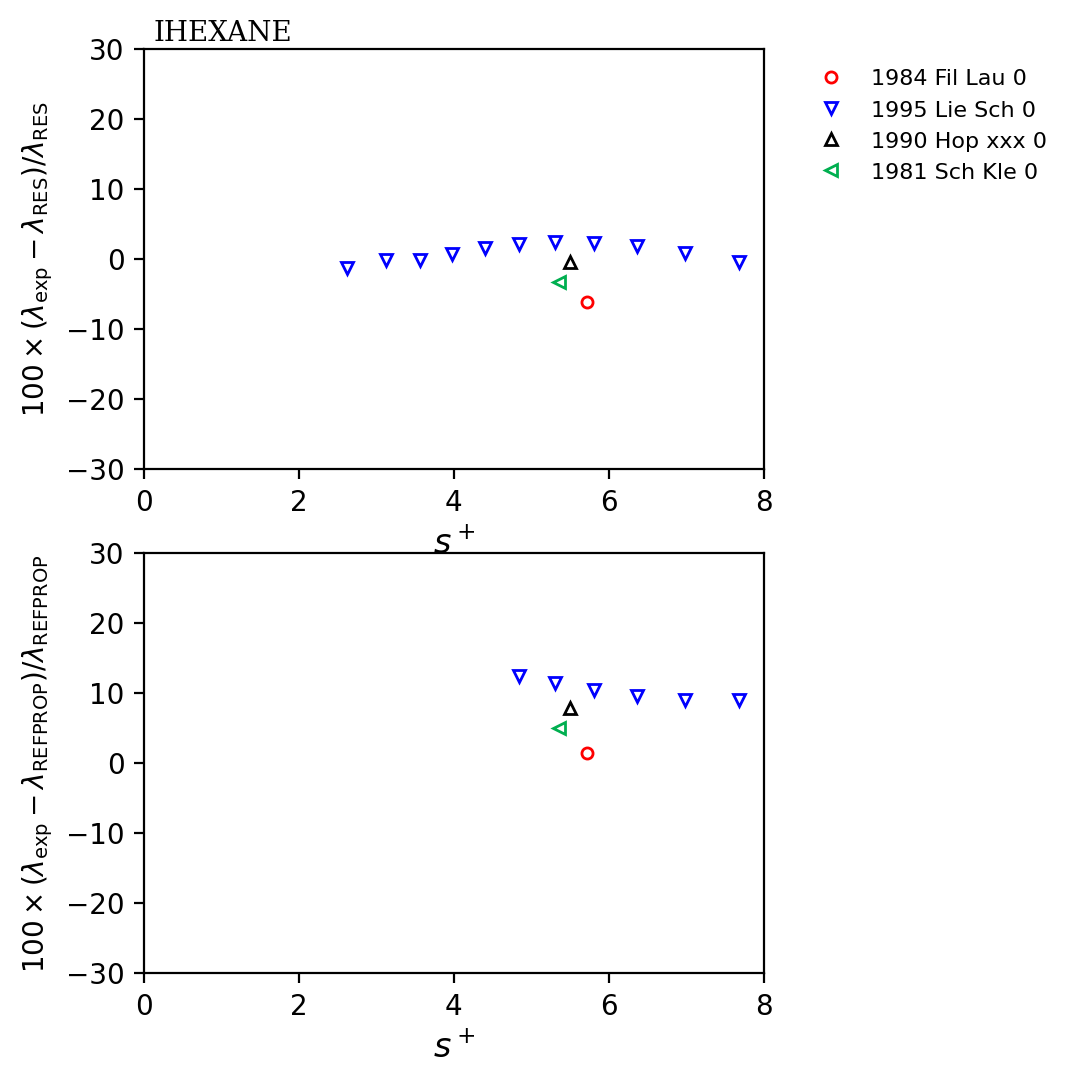

Supplement: Supplementary file 2 — ao4c10815_si_002.zip [file ao4c10815_si_002.zip › Supporting Information/Fig. TC2 - relative deviation - analyzable data - YFR EoS/IHEXANE.png]

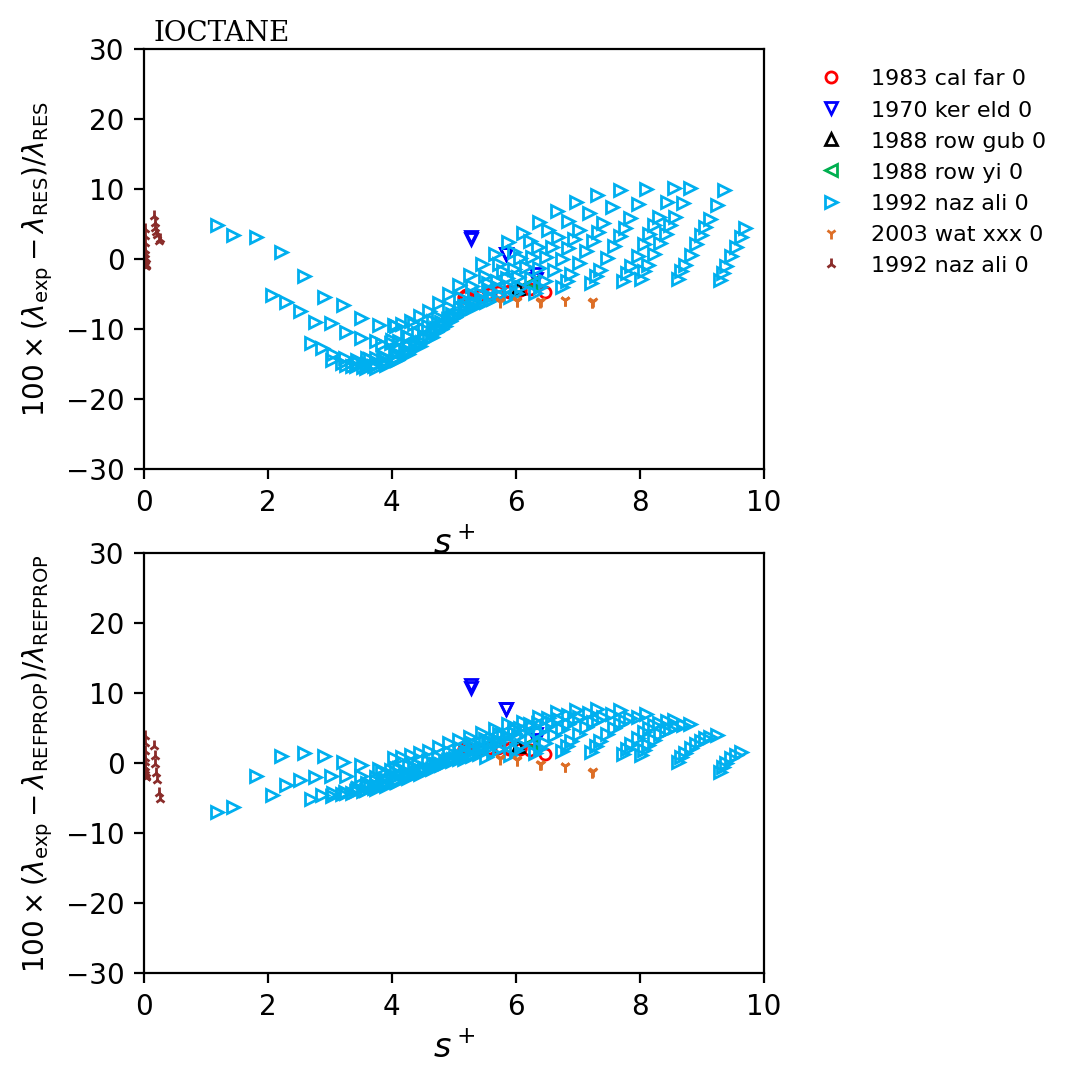

Supplement: Supplementary file 2 — ao4c10815_si_002.zip [file ao4c10815_si_002.zip › Supporting Information/Fig. TC2 - relative deviation - analyzable data - YFR EoS/IOCTANE.png]

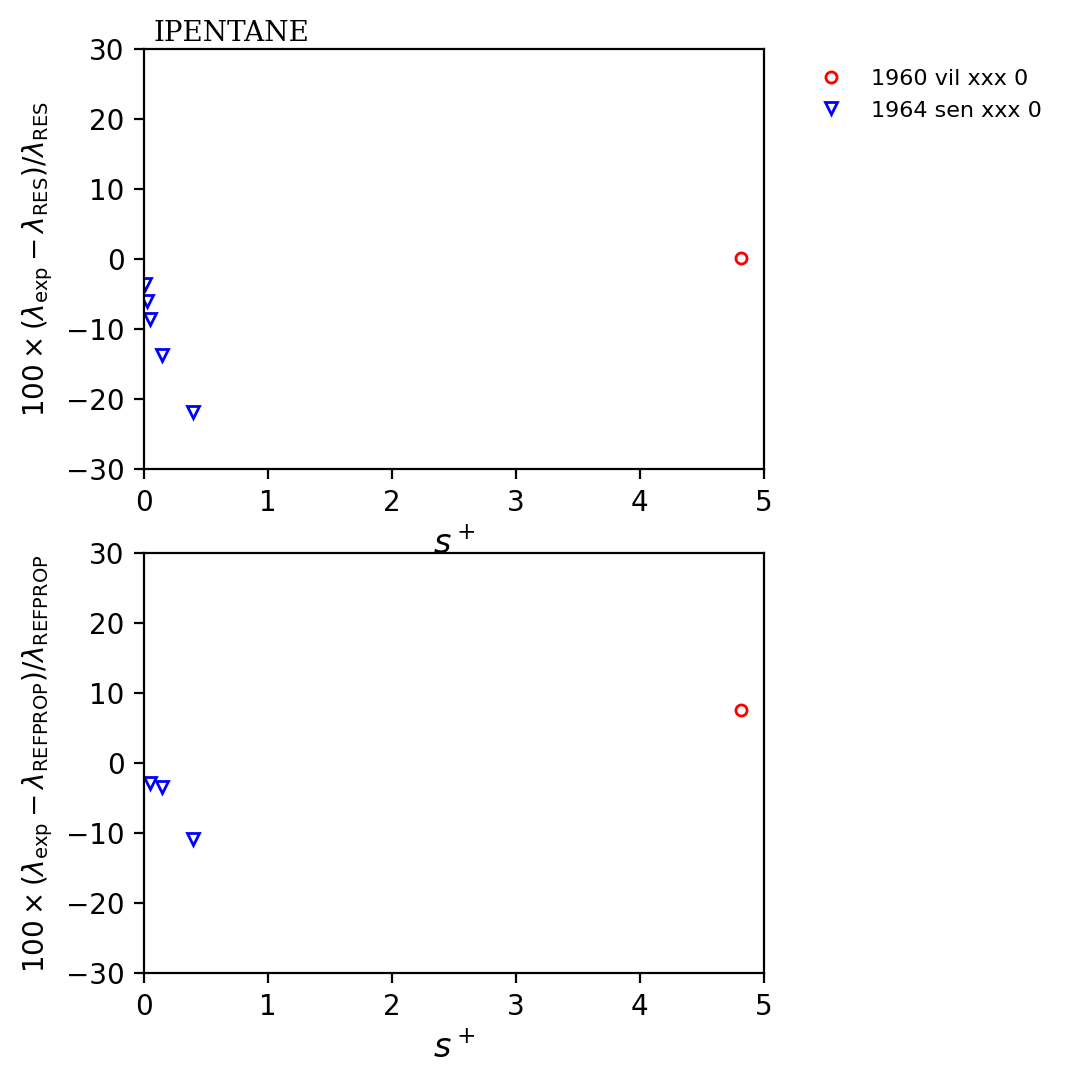

Supplement: Supplementary file 2 — ao4c10815_si_002.zip [file ao4c10815_si_002.zip › Supporting Information/Fig. TC2 - relative deviation - analyzable data - YFR EoS/IPENTANE.png]

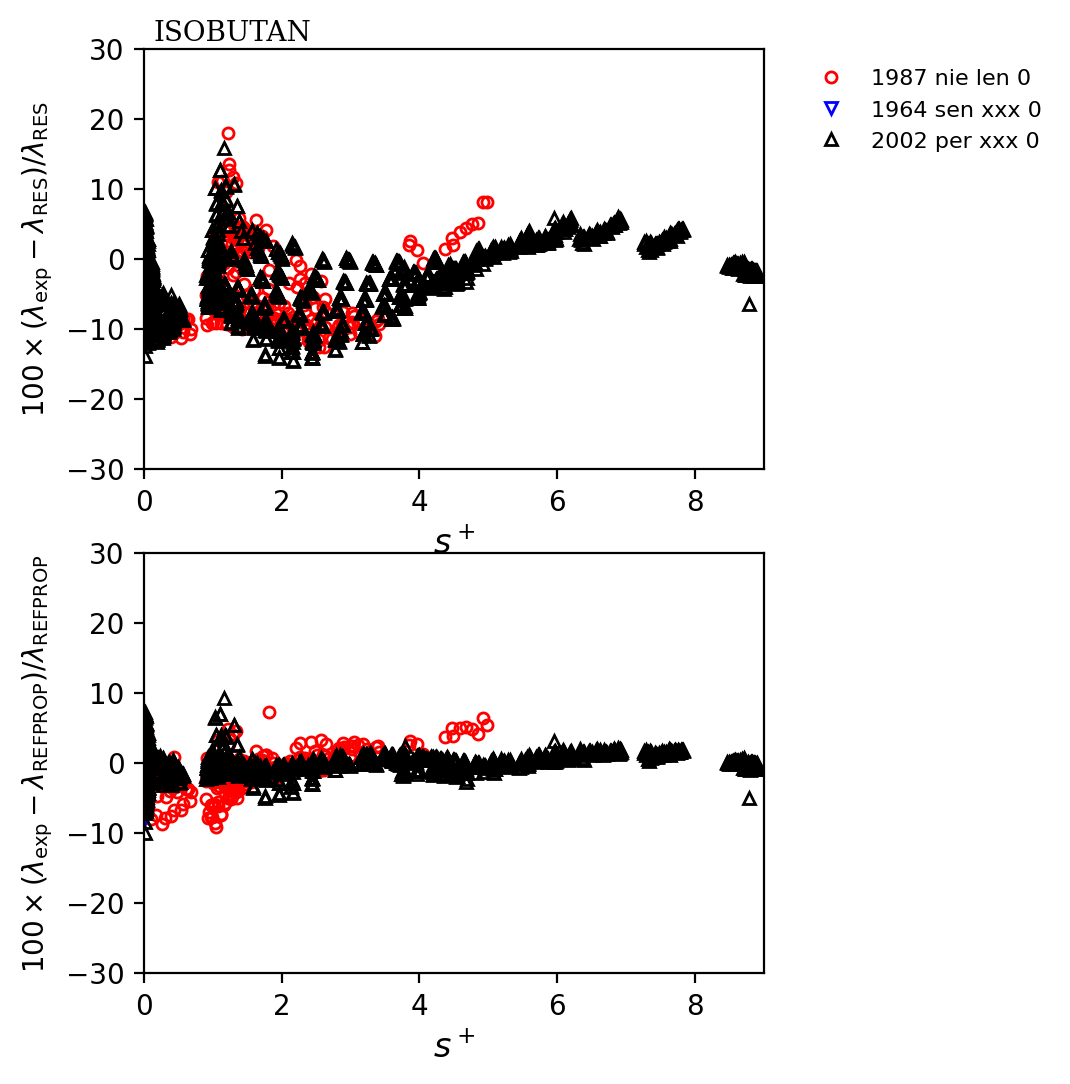

Supplement: Supplementary file 2 — ao4c10815_si_002.zip [file ao4c10815_si_002.zip › Supporting Information/Fig. TC2 - relative deviation - analyzable data - YFR EoS/ISOBUTAN.png]

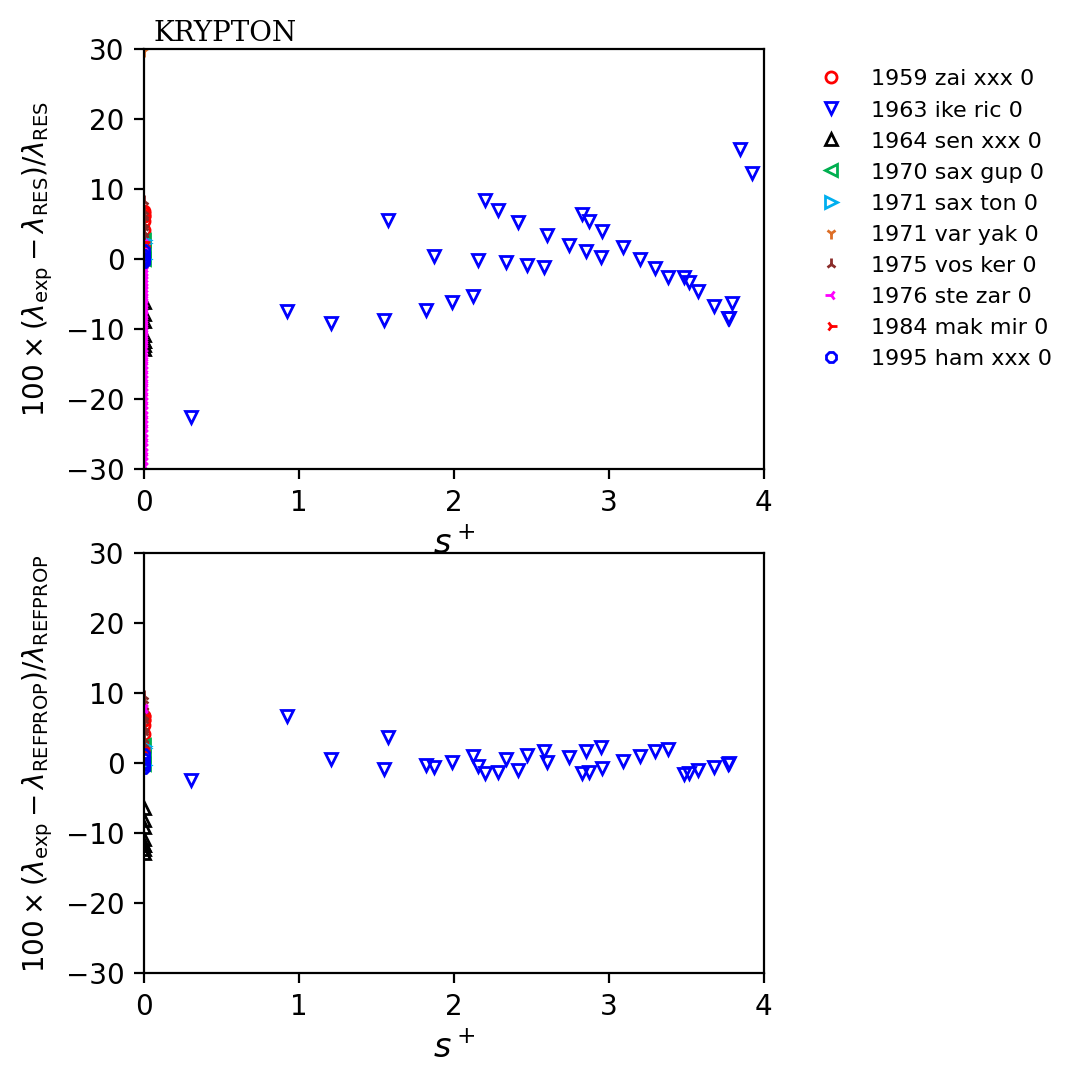

Supplement: Supplementary file 2 — ao4c10815_si_002.zip [file ao4c10815_si_002.zip › Supporting Information/Fig. TC2 - relative deviation - analyzable data - YFR EoS/KRYPTON.png]

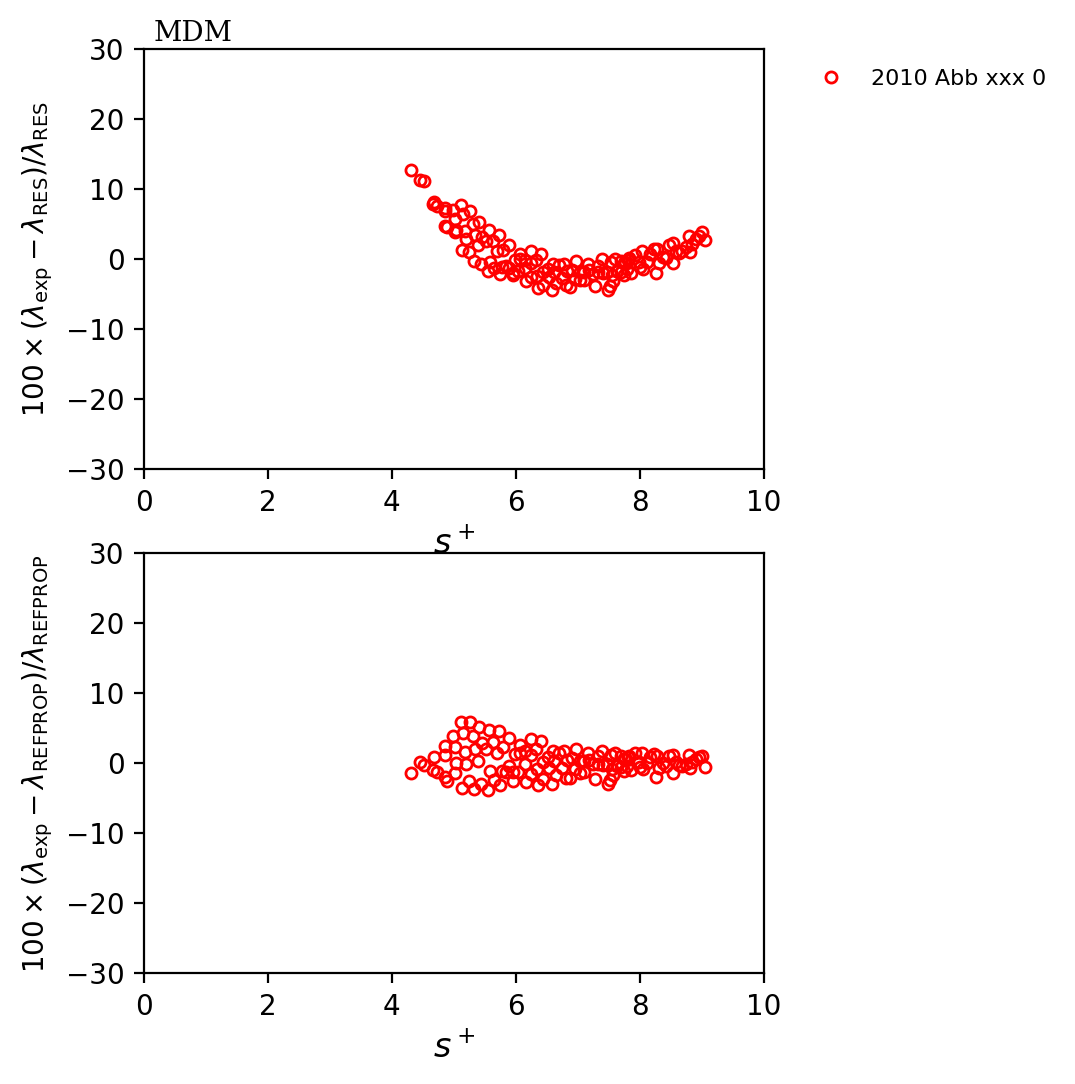

Supplement: Supplementary file 2 — ao4c10815_si_002.zip [file ao4c10815_si_002.zip › Supporting Information/Fig. TC2 - relative deviation - analyzable data - YFR EoS/MDM.png]

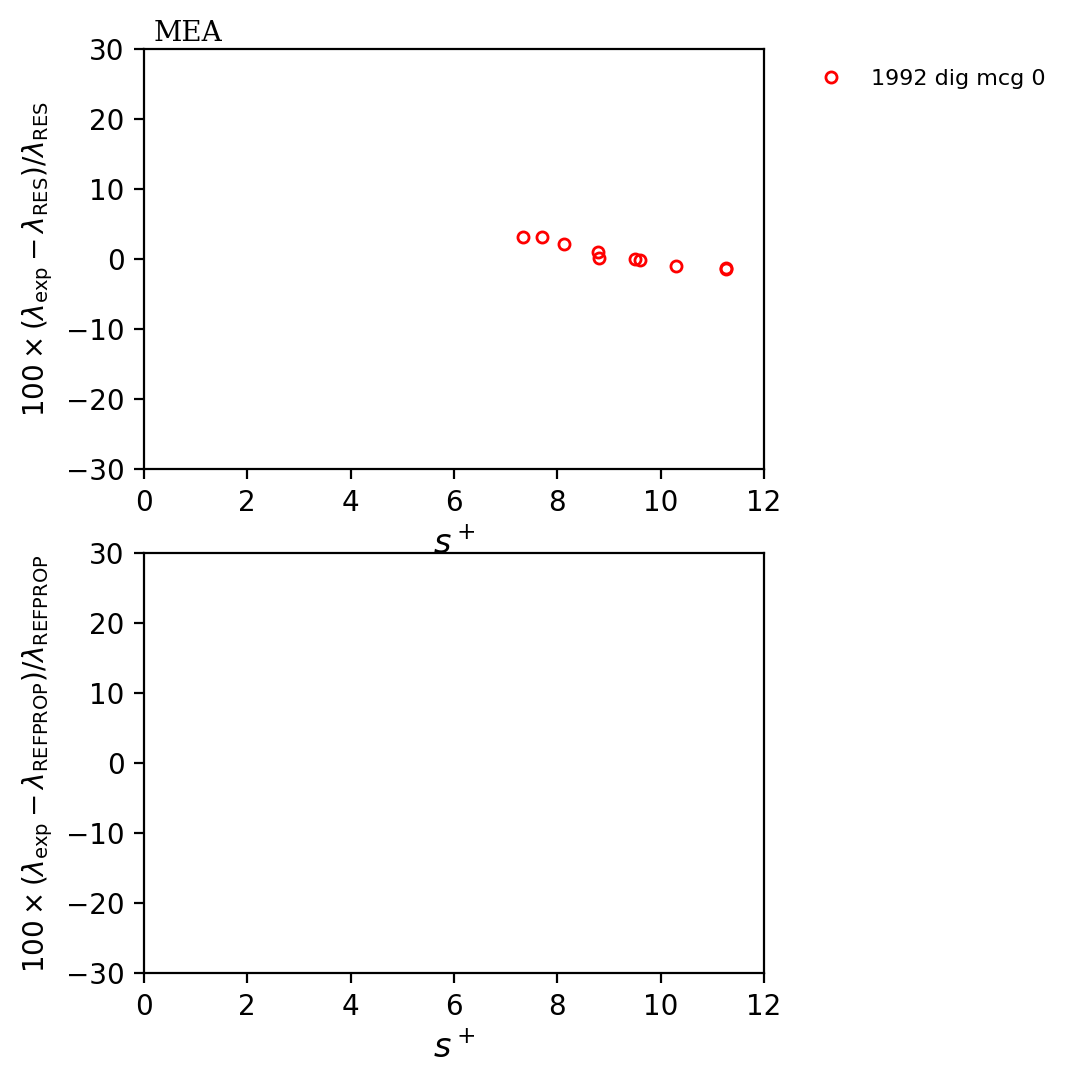

Supplement: Supplementary file 2 — ao4c10815_si_002.zip [file ao4c10815_si_002.zip › Supporting Information/Fig. TC2 - relative deviation - analyzable data - YFR EoS/MEA.png]

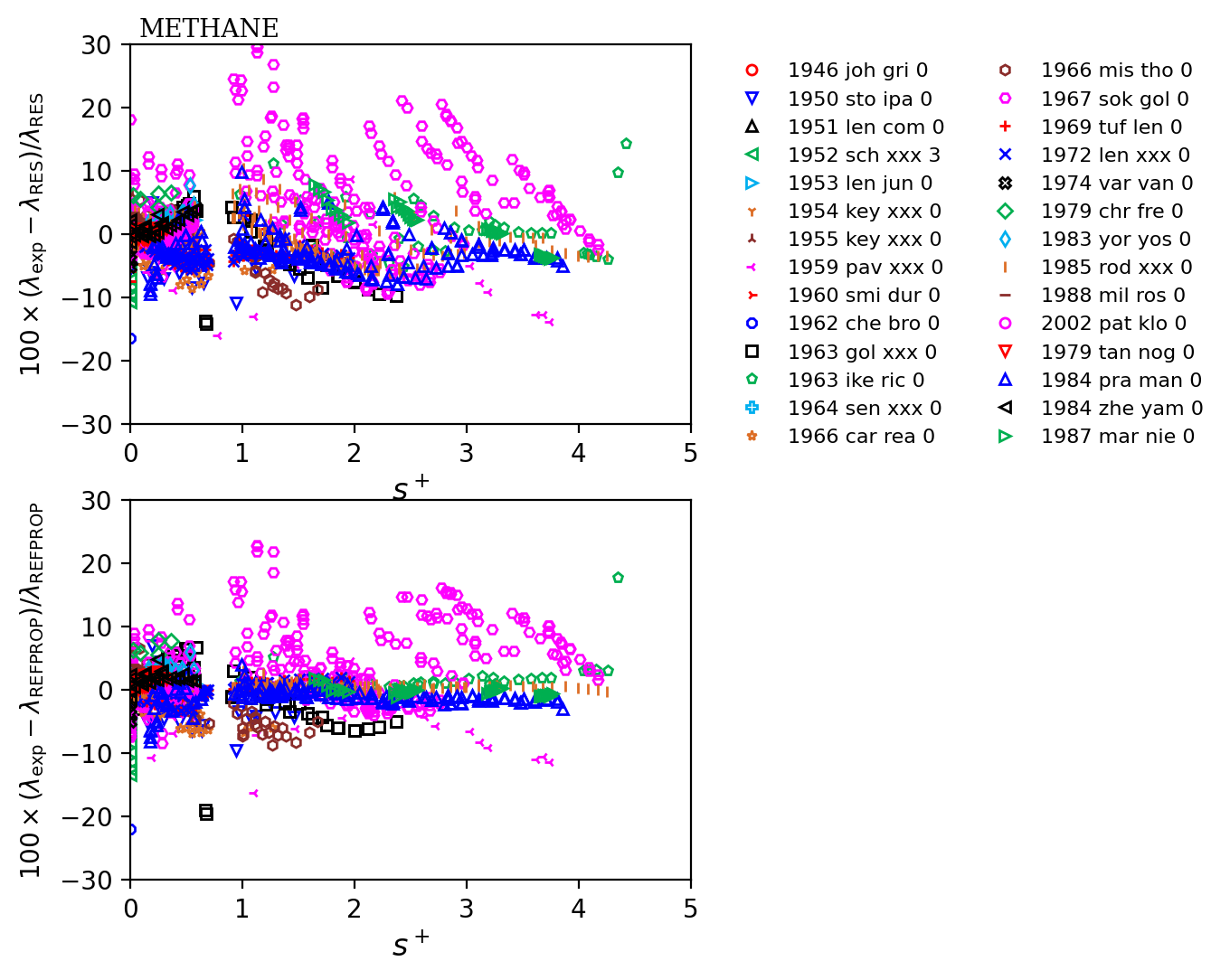

Supplement: Supplementary file 2 — ao4c10815_si_002.zip [file ao4c10815_si_002.zip › Supporting Information/Fig. TC2 - relative deviation - analyzable data - YFR EoS/METHANE.png]

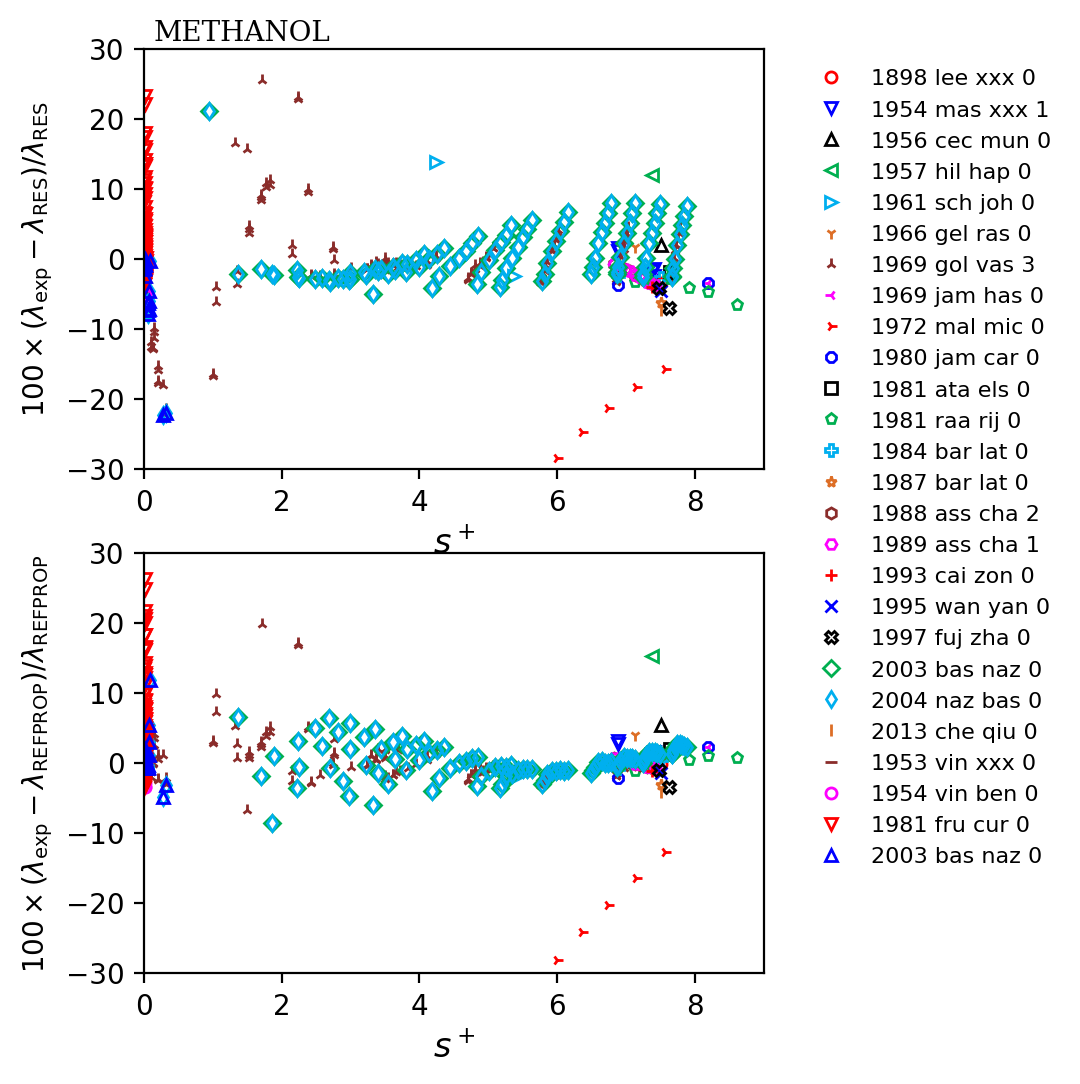

Supplement: Supplementary file 2 — ao4c10815_si_002.zip [file ao4c10815_si_002.zip › Supporting Information/Fig. TC2 - relative deviation - analyzable data - YFR EoS/METHANOL.png]

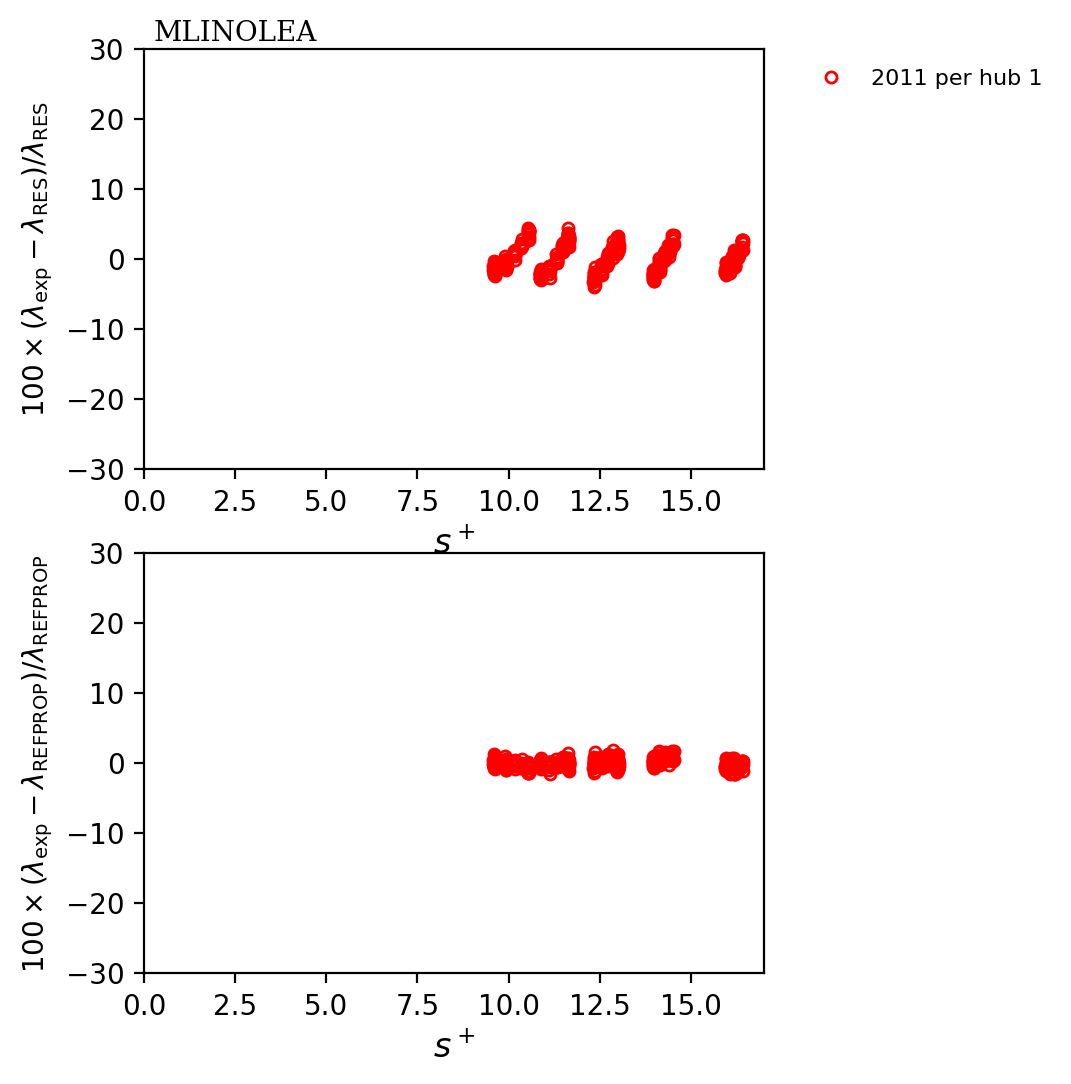

Supplement: Supplementary file 2 — ao4c10815_si_002.zip [file ao4c10815_si_002.zip › Supporting Information/Fig. TC2 - relative deviation - analyzable data - YFR EoS/MLINOLEA.png]

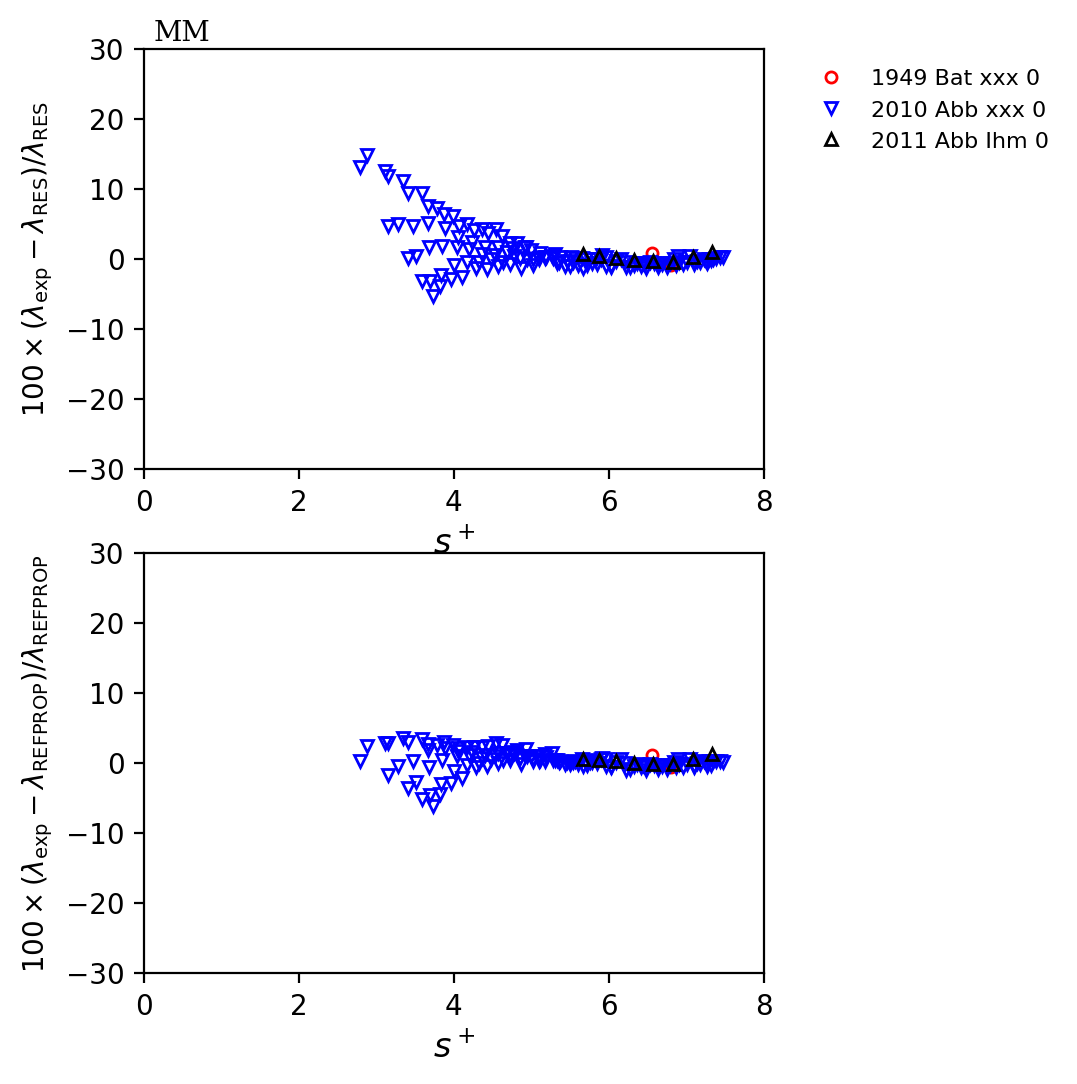

Supplement: Supplementary file 2 — ao4c10815_si_002.zip [file ao4c10815_si_002.zip › Supporting Information/Fig. TC2 - relative deviation - analyzable data - YFR EoS/MM.png]

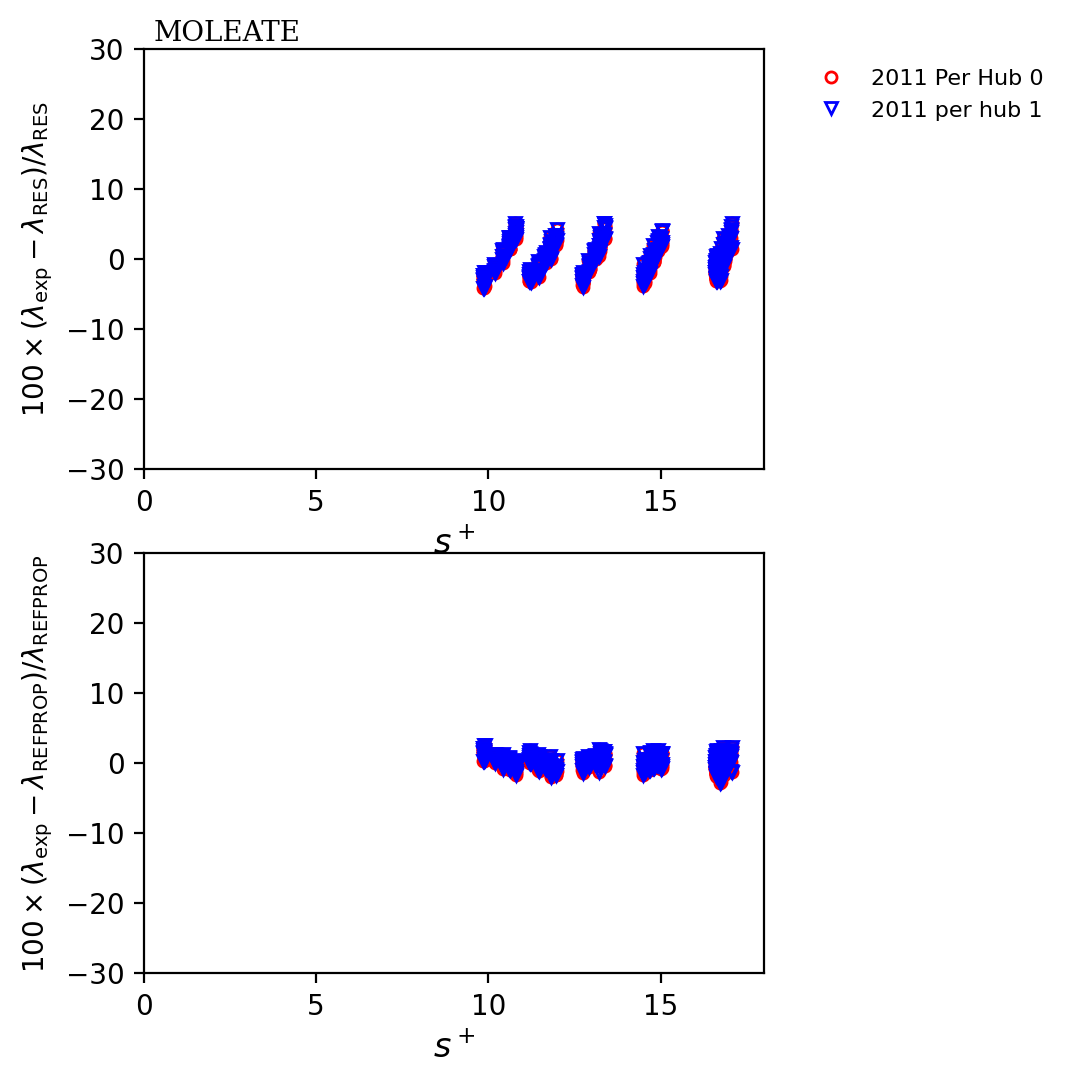

Supplement: Supplementary file 2 — ao4c10815_si_002.zip [file ao4c10815_si_002.zip › Supporting Information/Fig. TC2 - relative deviation - analyzable data - YFR EoS/MOLEATE.png]

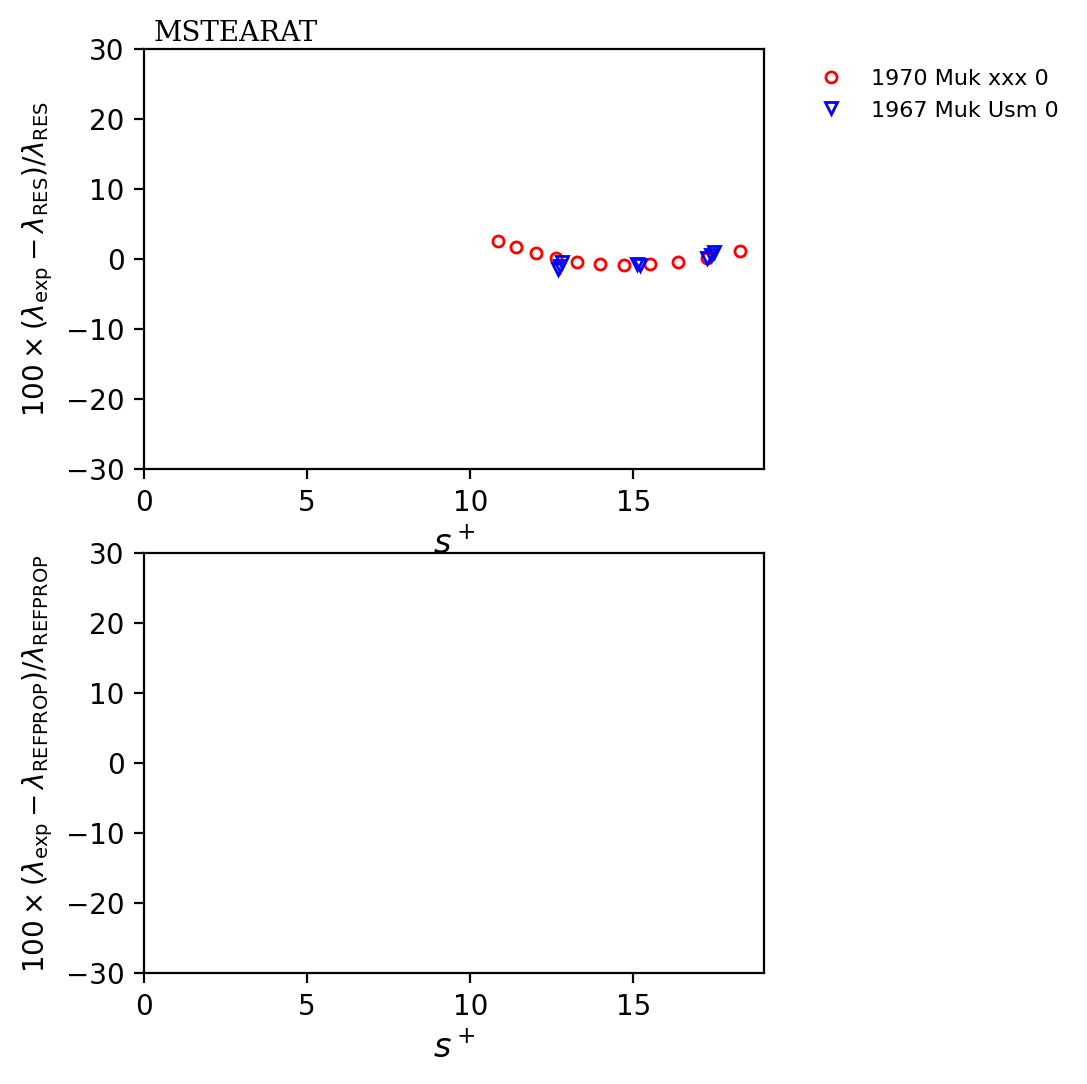

Supplement: Supplementary file 2 — ao4c10815_si_002.zip [file ao4c10815_si_002.zip › Supporting Information/Fig. TC2 - relative deviation - analyzable data - YFR EoS/MSTEARAT.png]

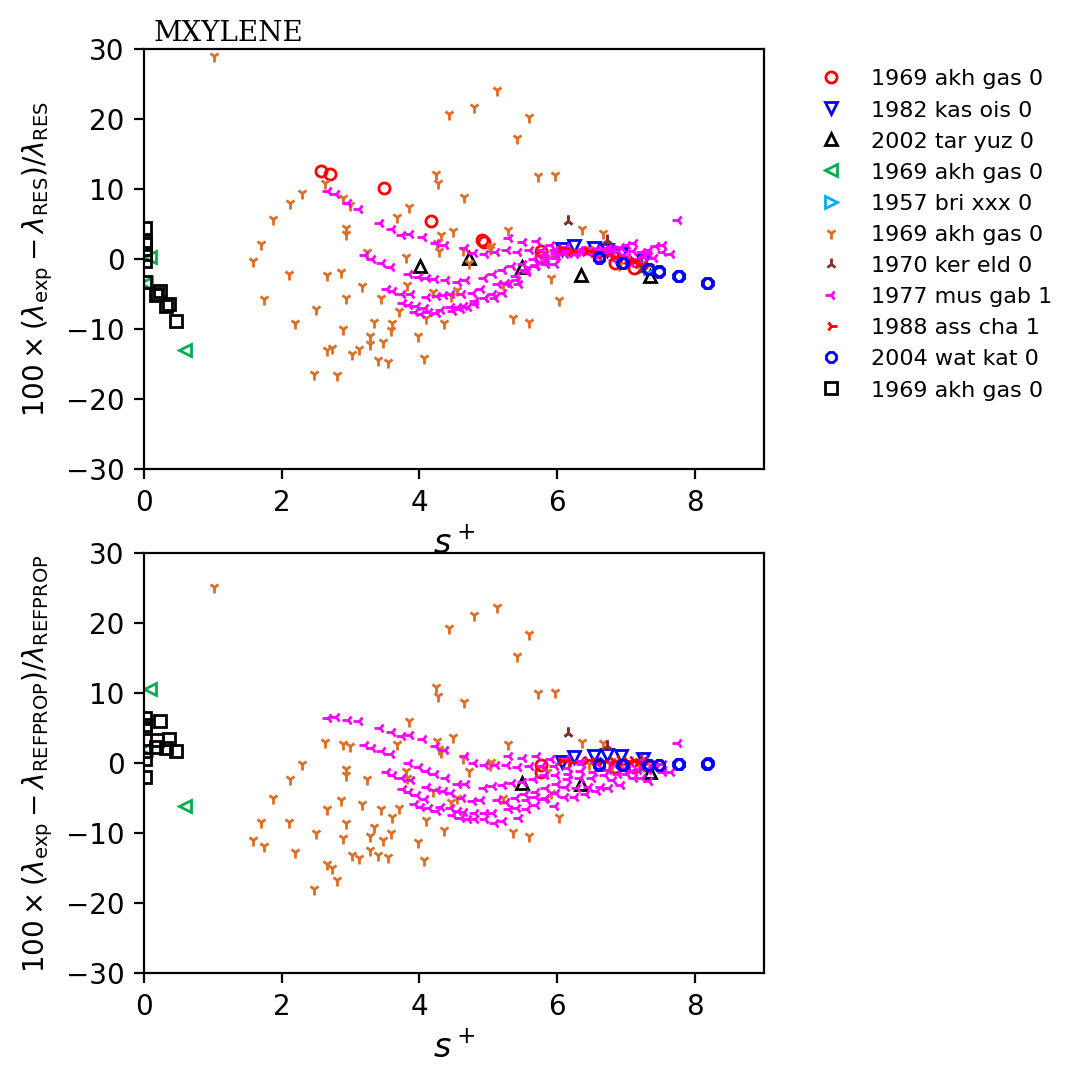

Supplement: Supplementary file 2 — ao4c10815_si_002.zip [file ao4c10815_si_002.zip › Supporting Information/Fig. TC2 - relative deviation - analyzable data - YFR EoS/MXYLENE.png]

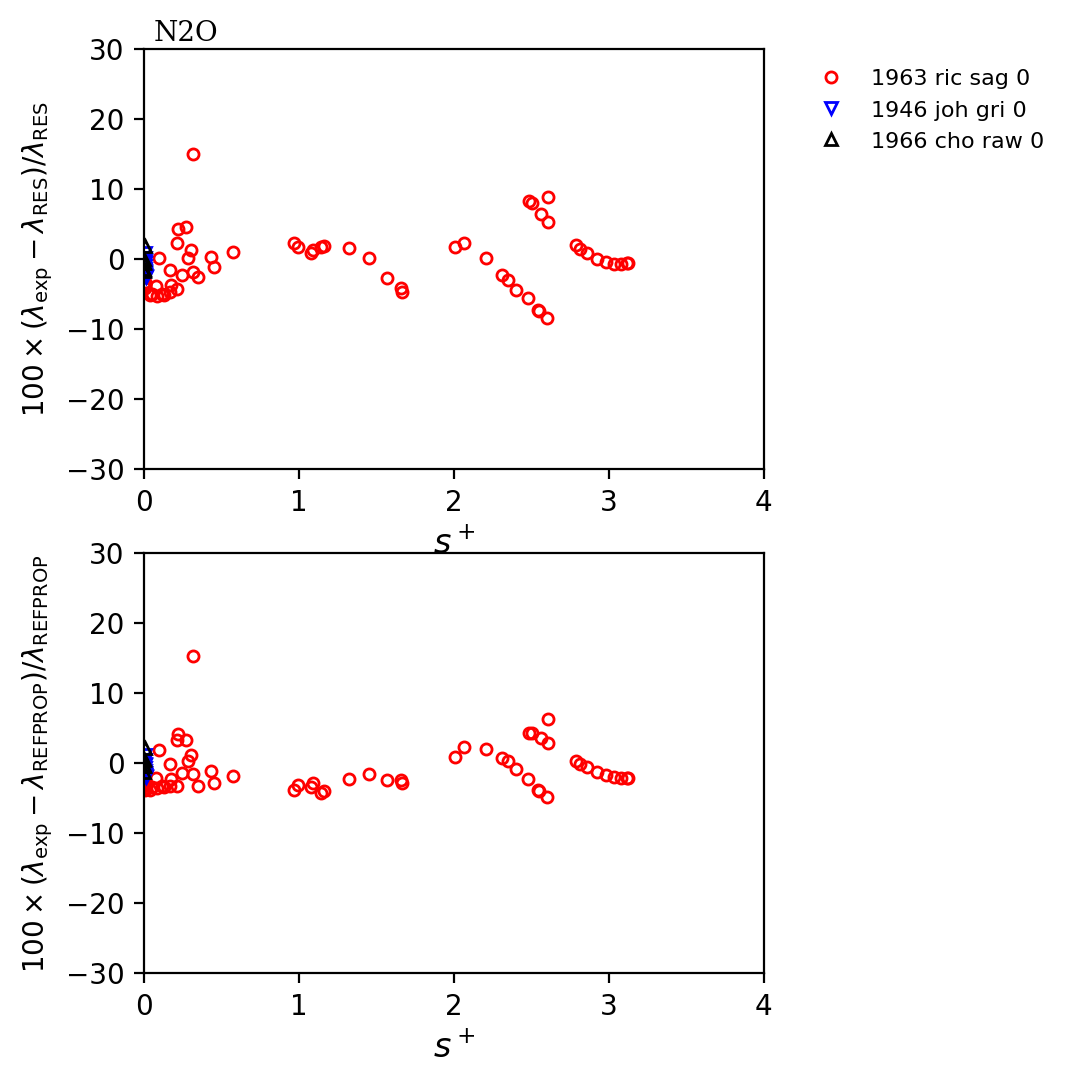

Supplement: Supplementary file 2 — ao4c10815_si_002.zip [file ao4c10815_si_002.zip › Supporting Information/Fig. TC2 - relative deviation - analyzable data - YFR EoS/N2O.png]

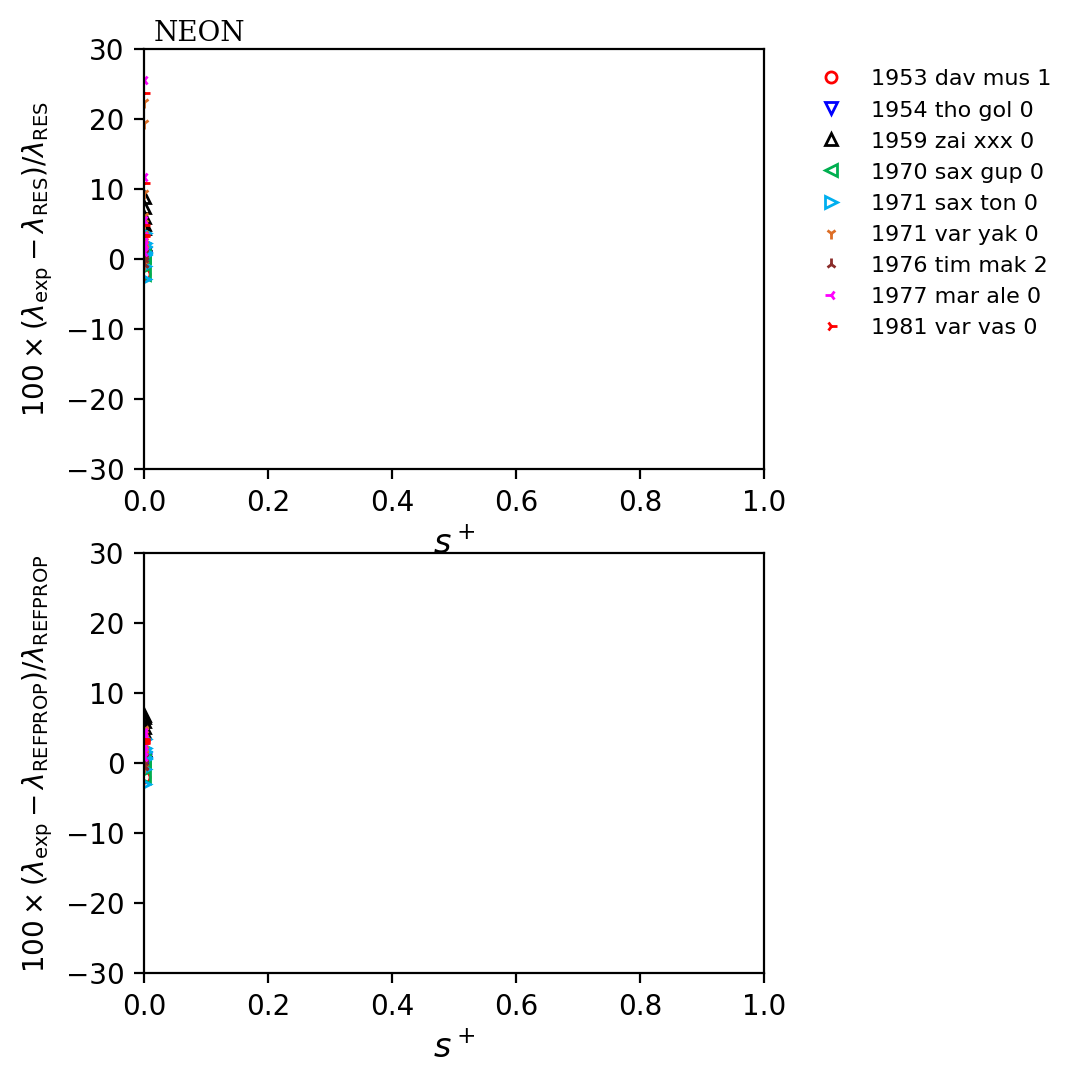

Supplement: Supplementary file 2 — ao4c10815_si_002.zip [file ao4c10815_si_002.zip › Supporting Information/Fig. TC2 - relative deviation - analyzable data - YFR EoS/NEON.png]

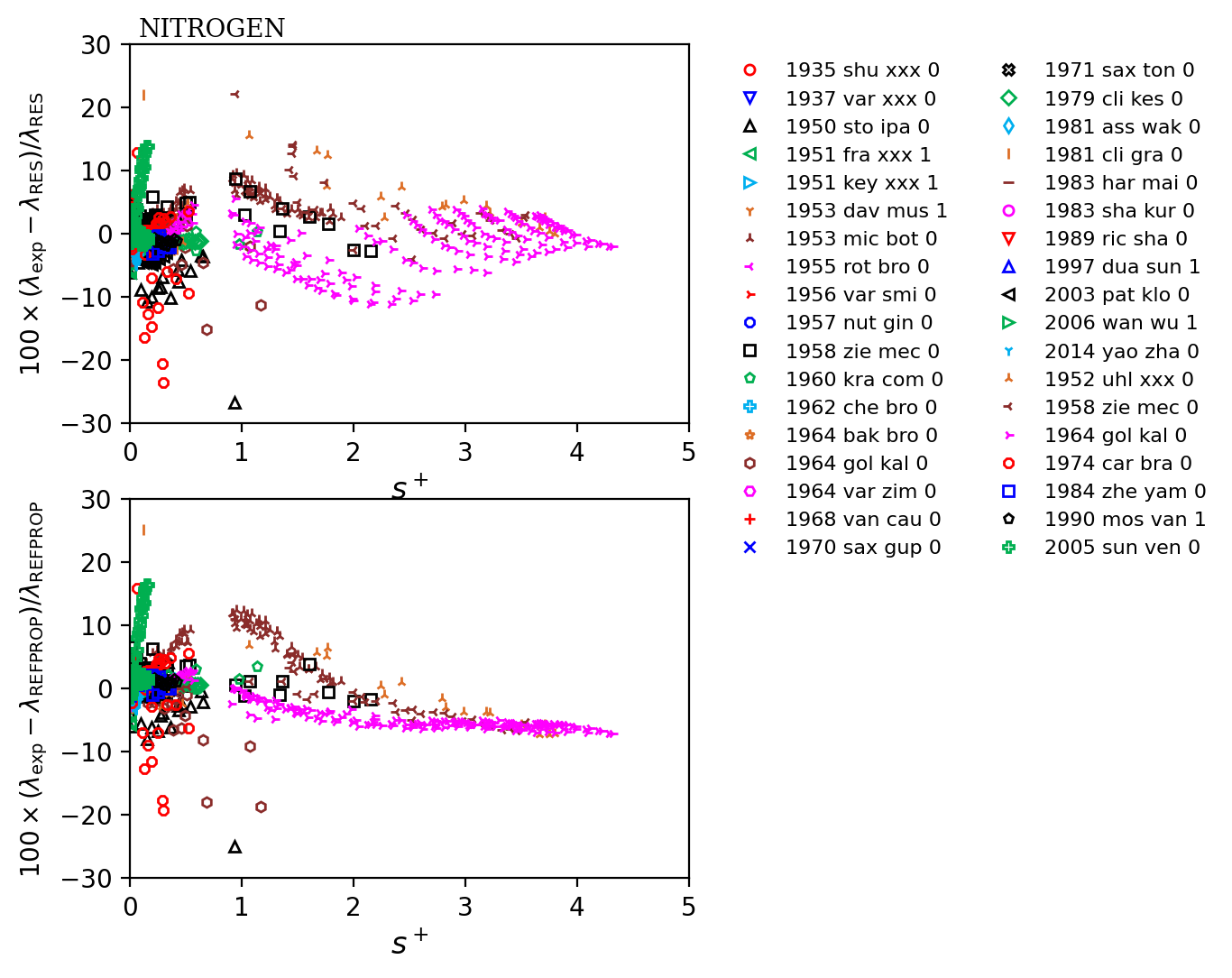

Supplement: Supplementary file 2 — ao4c10815_si_002.zip [file ao4c10815_si_002.zip › Supporting Information/Fig. TC2 - relative deviation - analyzable data - YFR EoS/NITROGEN.png]

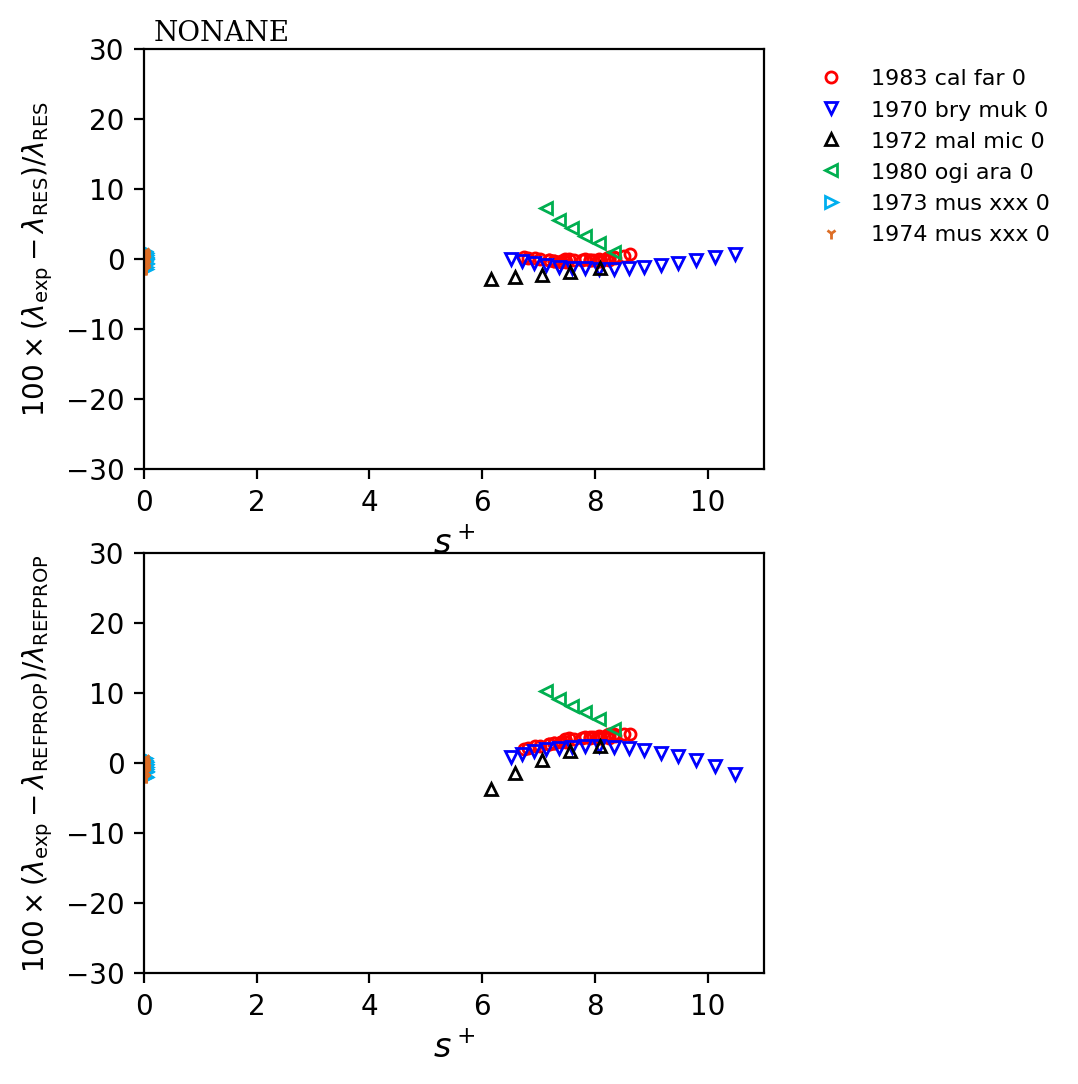

Supplement: Supplementary file 2 — ao4c10815_si_002.zip [file ao4c10815_si_002.zip › Supporting Information/Fig. TC2 - relative deviation - analyzable data - YFR EoS/NONANE.png]

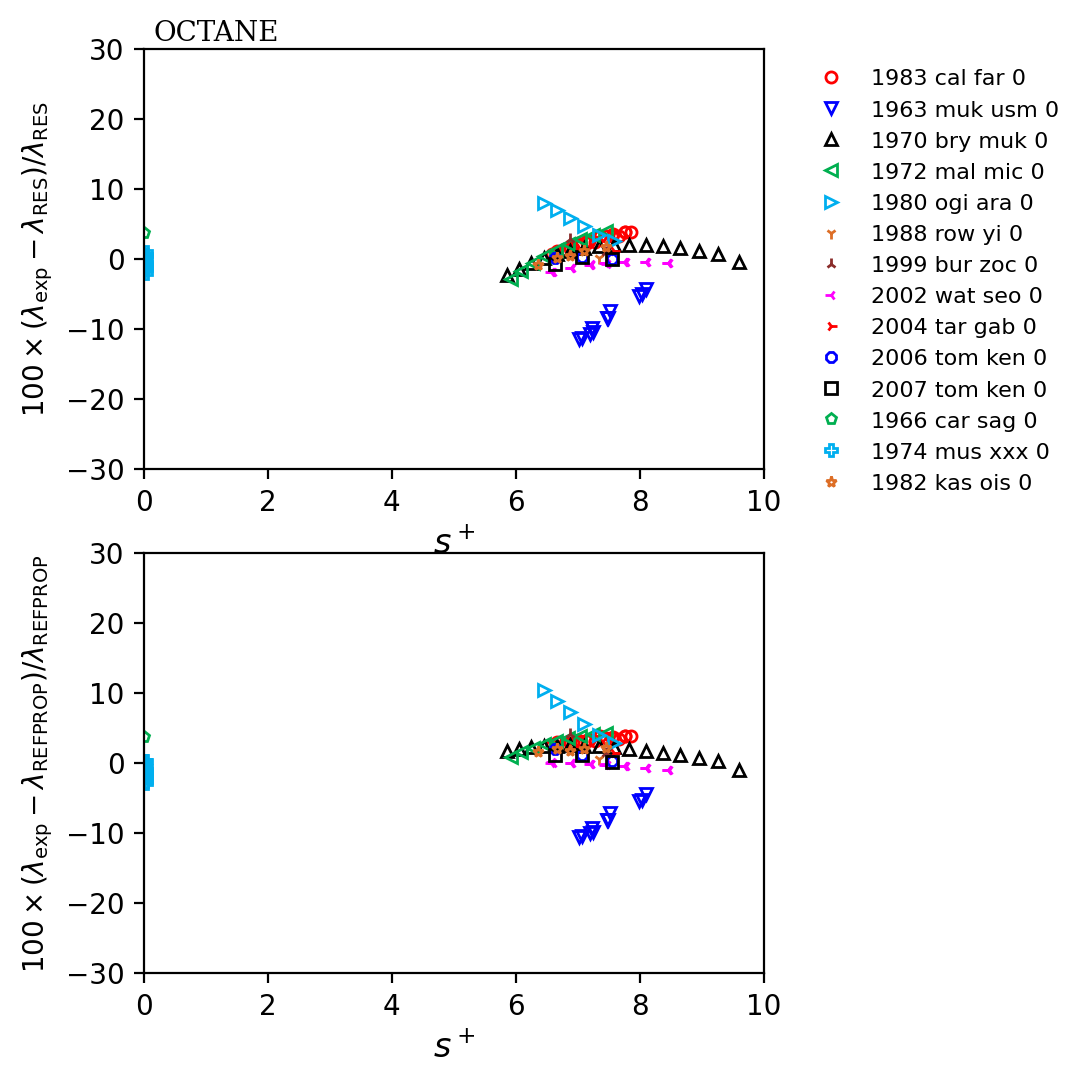

Supplement: Supplementary file 2 — ao4c10815_si_002.zip [file ao4c10815_si_002.zip › Supporting Information/Fig. TC2 - relative deviation - analyzable data - YFR EoS/OCTANE.png]

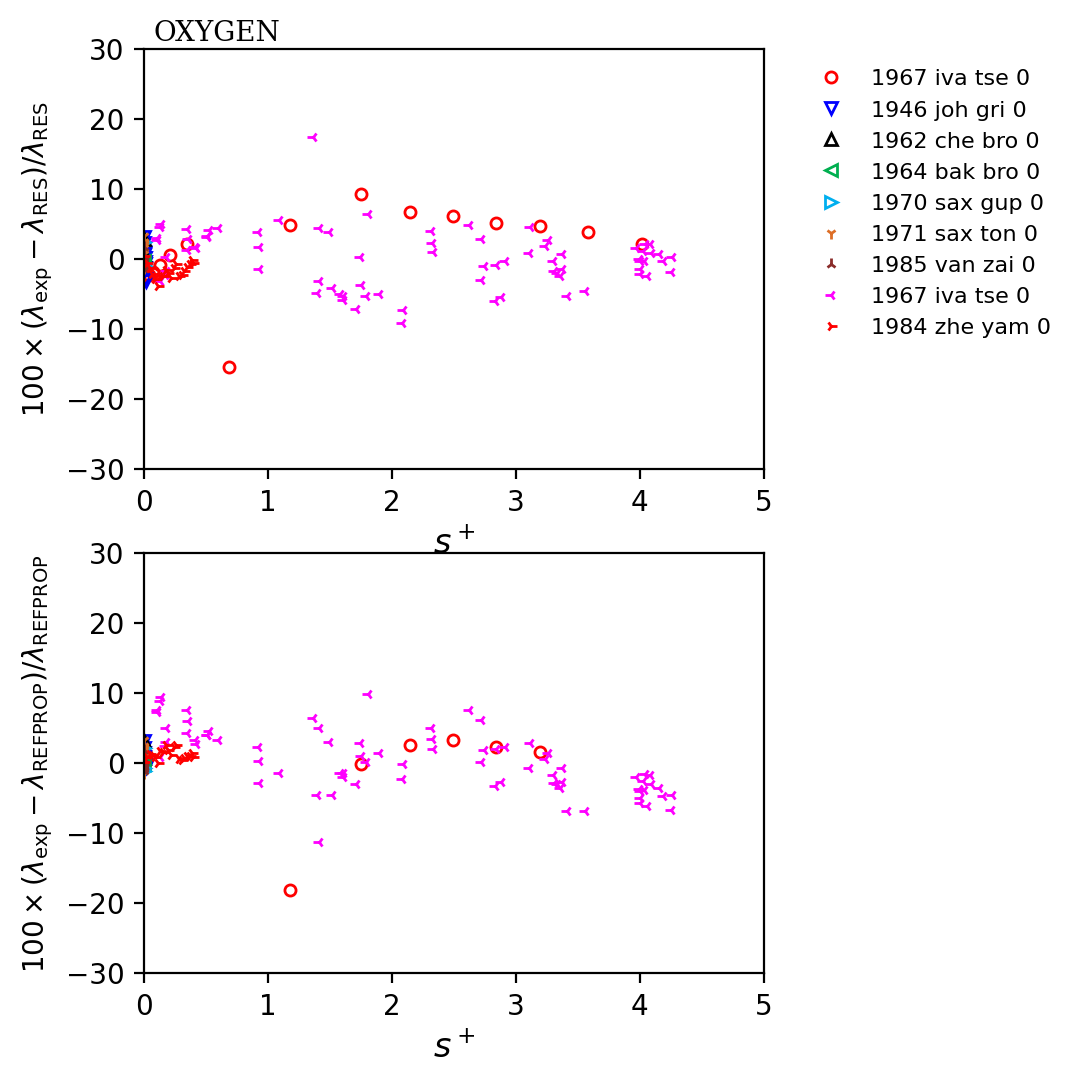

Supplement: Supplementary file 2 — ao4c10815_si_002.zip [file ao4c10815_si_002.zip › Supporting Information/Fig. TC2 - relative deviation - analyzable data - YFR EoS/OXYGEN.png]

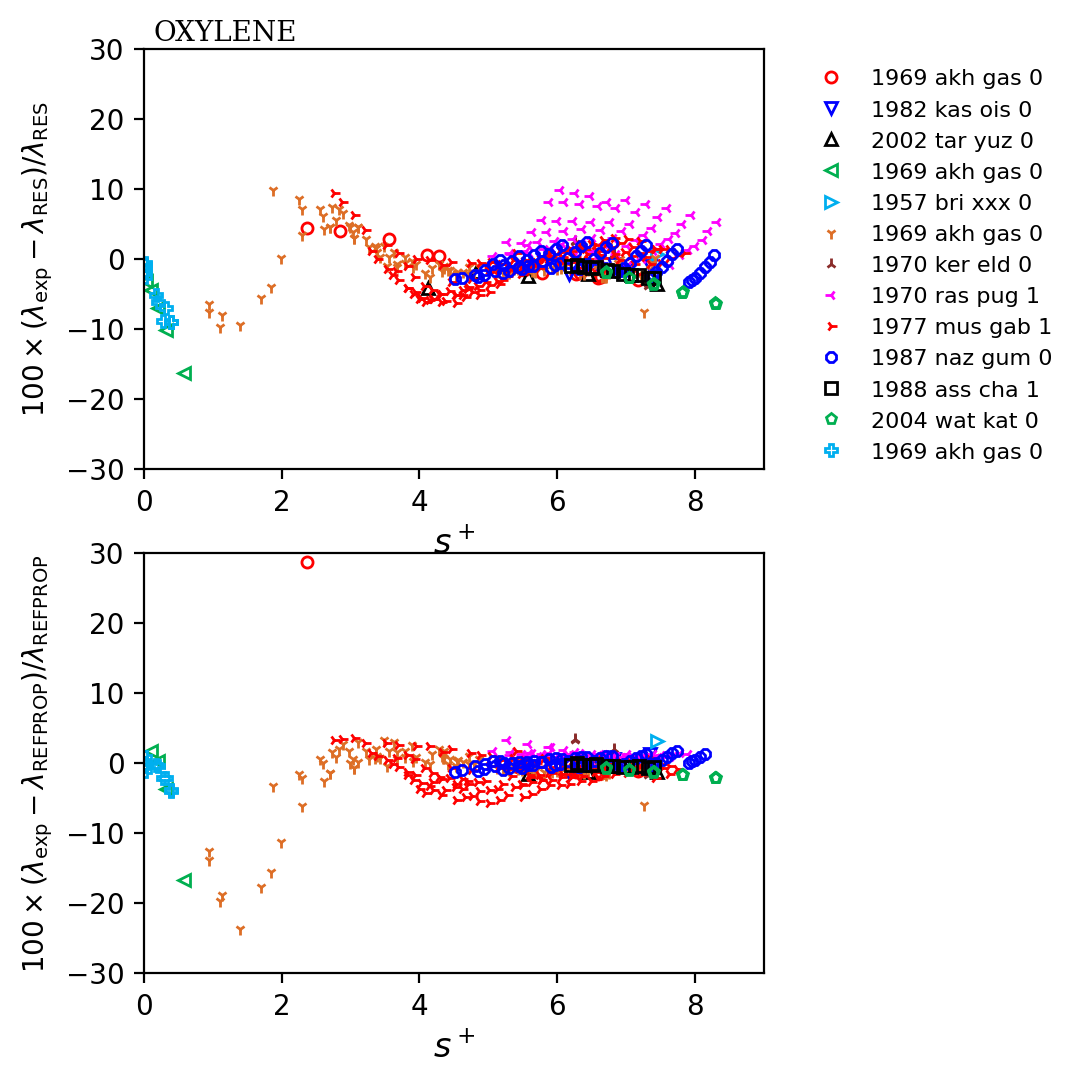

Supplement: Supplementary file 2 — ao4c10815_si_002.zip [file ao4c10815_si_002.zip › Supporting Information/Fig. TC2 - relative deviation - analyzable data - YFR EoS/OXYLENE.png]

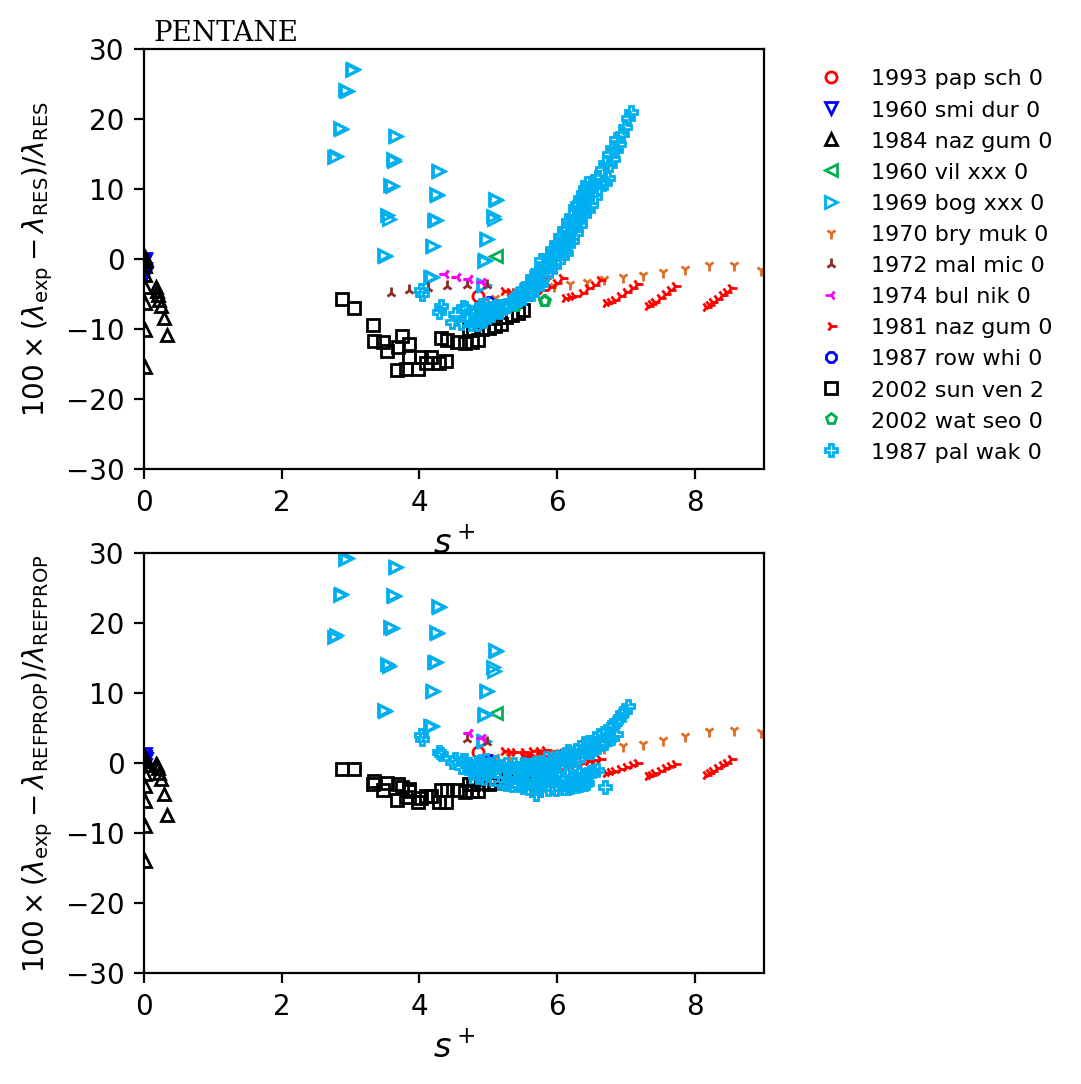

Supplement: Supplementary file 2 — ao4c10815_si_002.zip [file ao4c10815_si_002.zip › Supporting Information/Fig. TC2 - relative deviation - analyzable data - YFR EoS/PENTANE.png]

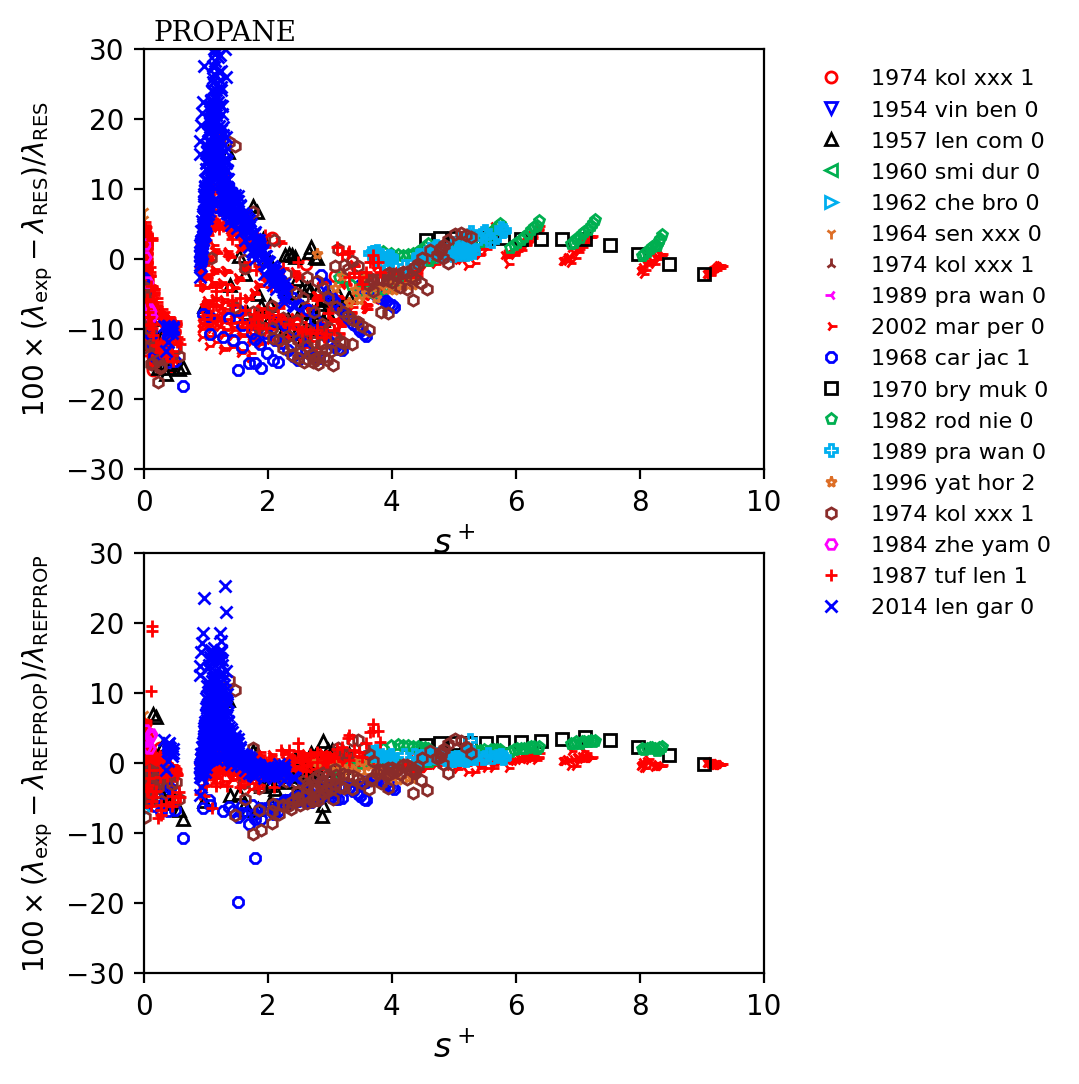

Supplement: Supplementary file 2 — ao4c10815_si_002.zip [file ao4c10815_si_002.zip › Supporting Information/Fig. TC2 - relative deviation - analyzable data - YFR EoS/PROPANE.png]
