# Supplementary figures and images for: Viscosity and Thermal Conductivity Models of 151 Common Fluids Based on Residual Entropy Scaling and Cubic Equations of State (part 1 of 2)
Source: ACS Omega. 2025 Feb 8;10(6):6124–34. doi: 10.1021/acsomega.4c10815 (PMC11840589; doi:10.1021/acsomega.4c10815)

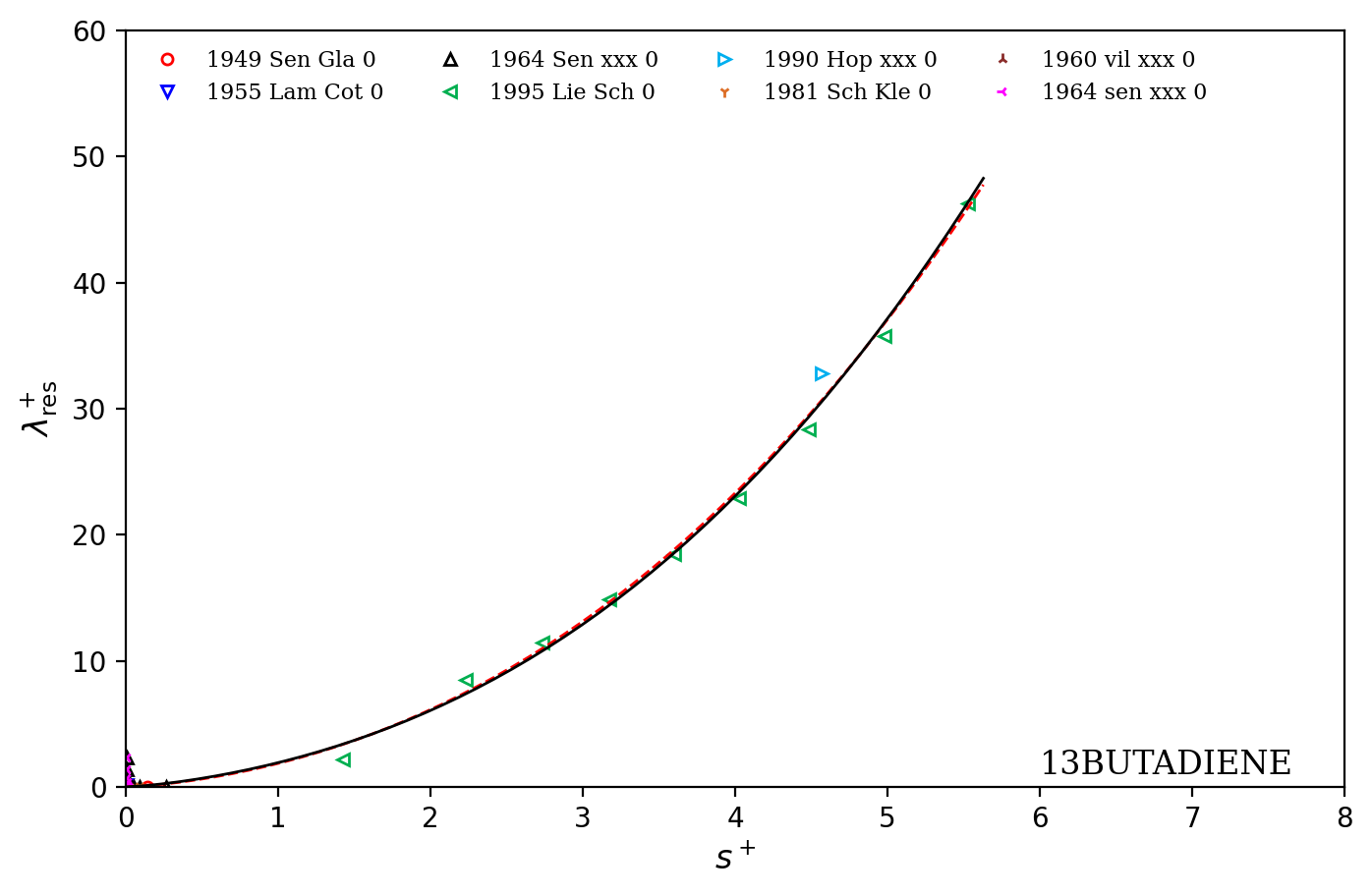

Supplement: Supplementary file 2 — ao4c10815_si_002.zip [file ao4c10815_si_002.zip › Supporting Information/Fig. TC1 - s_plus vs lambda_plus - all data - YFR EoS/13BUTADIENE.png]

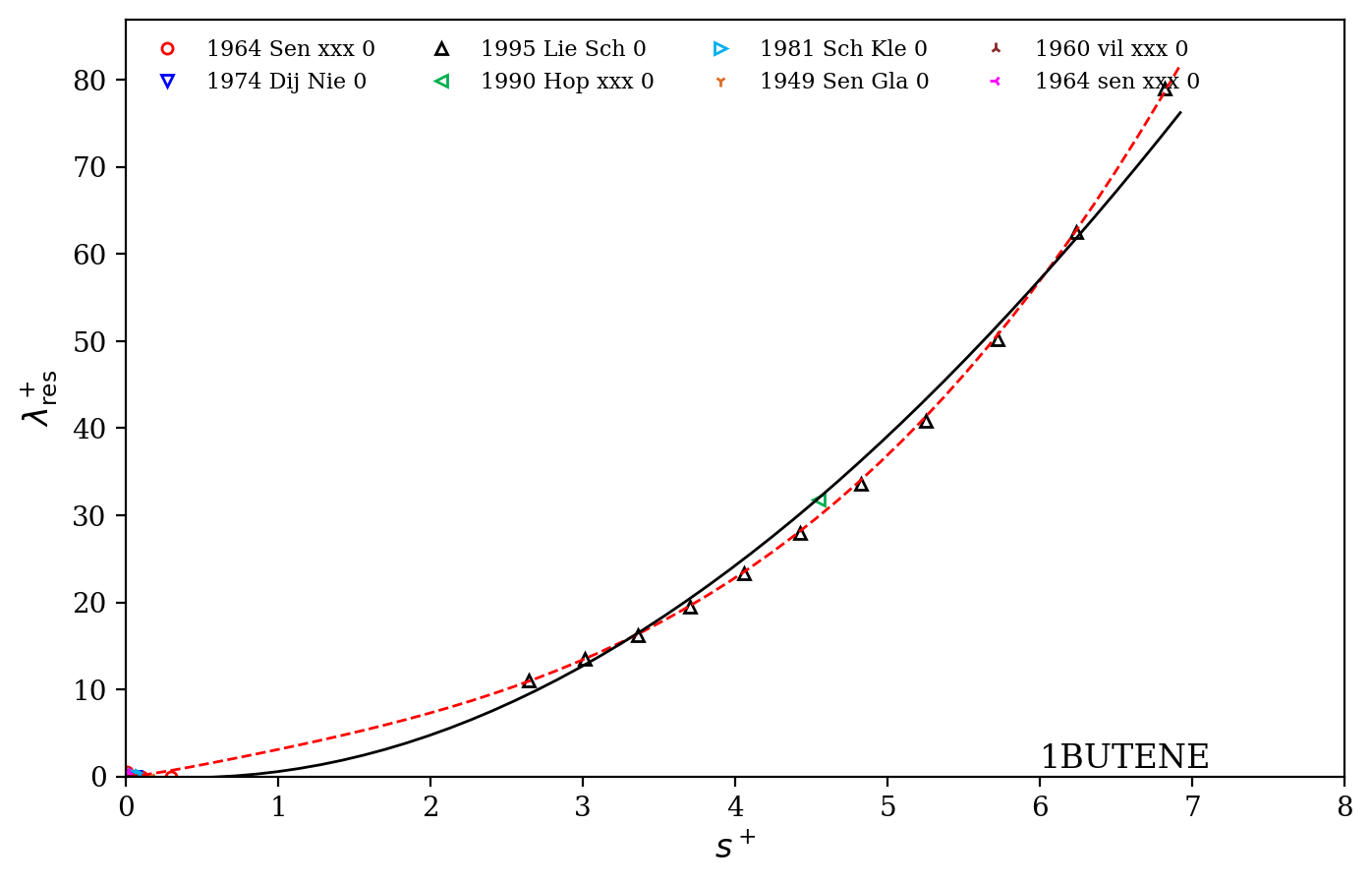

Supplement: Supplementary file 2 — ao4c10815_si_002.zip [file ao4c10815_si_002.zip › Supporting Information/Fig. TC1 - s_plus vs lambda_plus - all data - YFR EoS/1BUTENE.png]

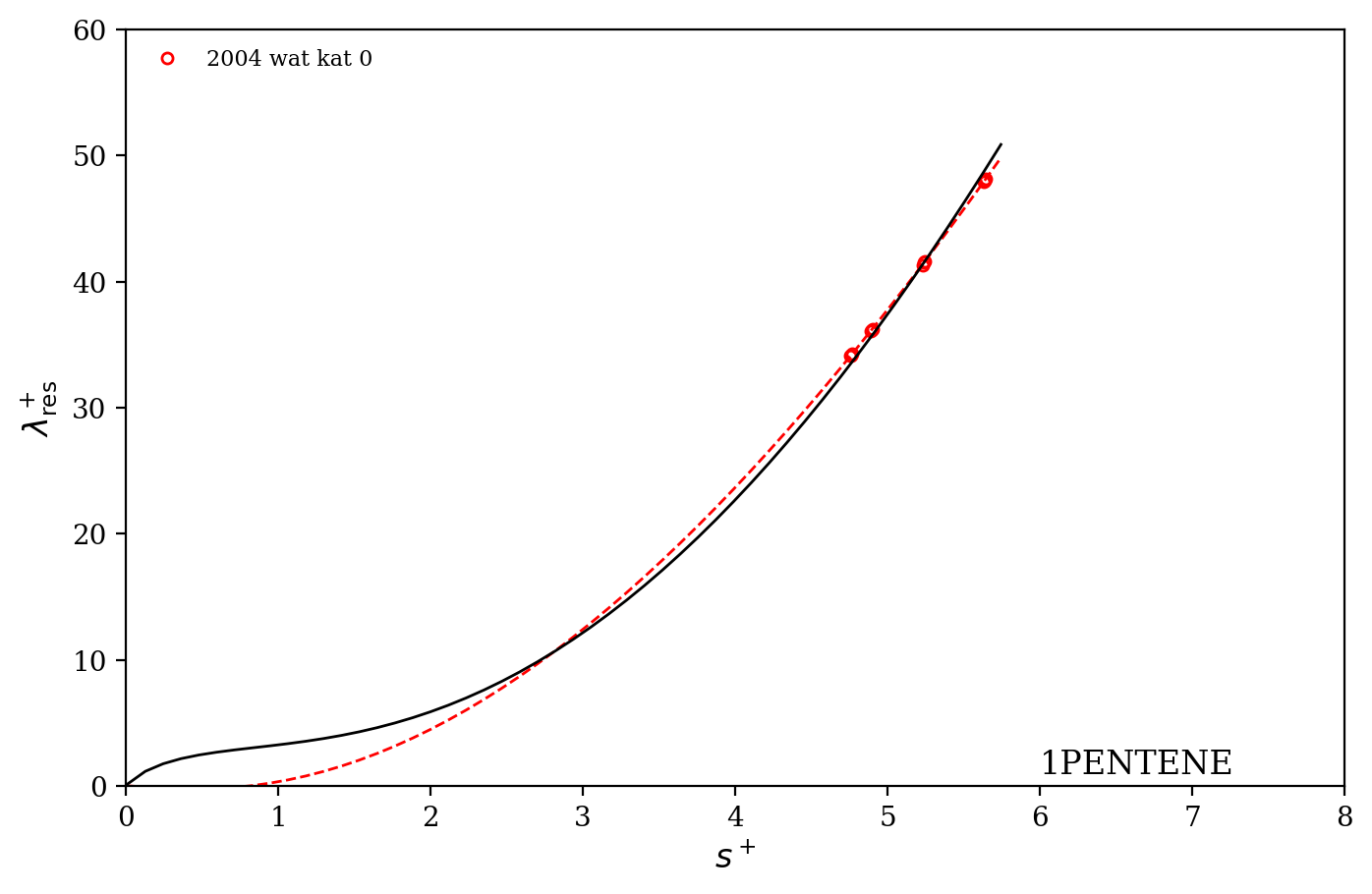

Supplement: Supplementary file 2 — ao4c10815_si_002.zip [file ao4c10815_si_002.zip › Supporting Information/Fig. TC1 - s_plus vs lambda_plus - all data - YFR EoS/1PENTENE.png]

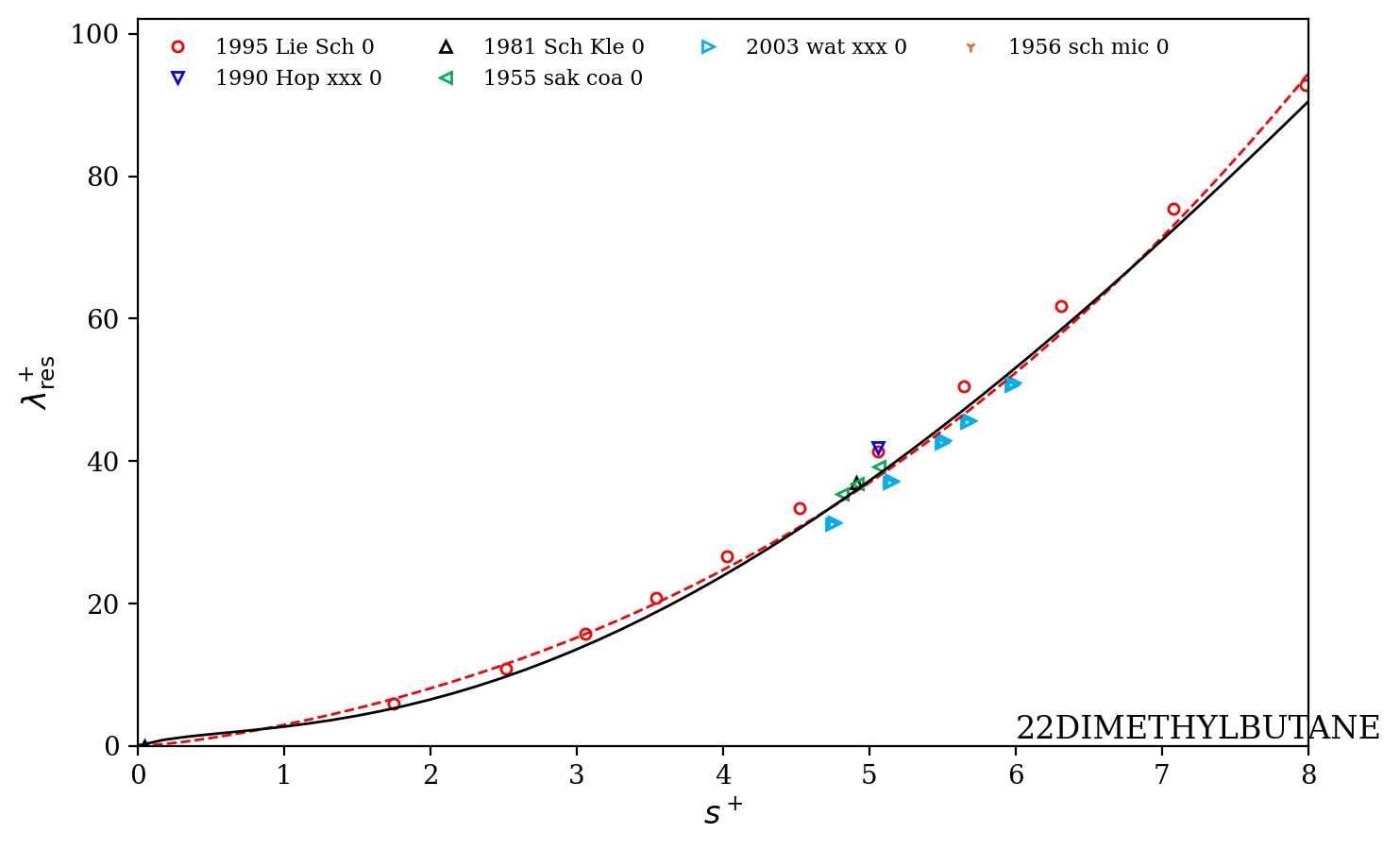

Supplement: Supplementary file 2 — ao4c10815_si_002.zip [file ao4c10815_si_002.zip › Supporting Information/Fig. TC1 - s_plus vs lambda_plus - all data - YFR EoS/22DIMETHYLBUTANE.png]

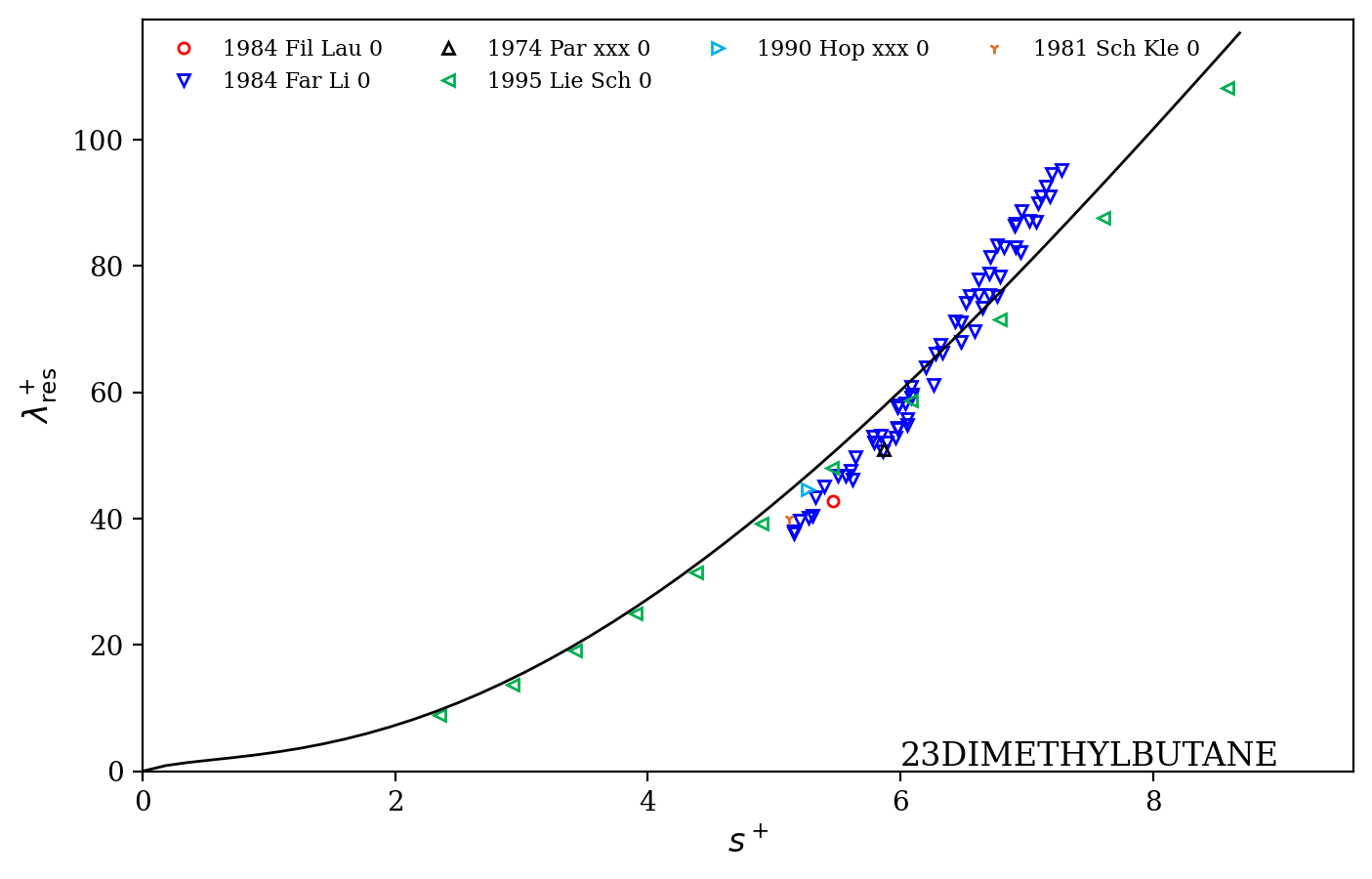

Supplement: Supplementary file 2 — ao4c10815_si_002.zip [file ao4c10815_si_002.zip › Supporting Information/Fig. TC1 - s_plus vs lambda_plus - all data - YFR EoS/23DIMETHYLBUTANE.png]

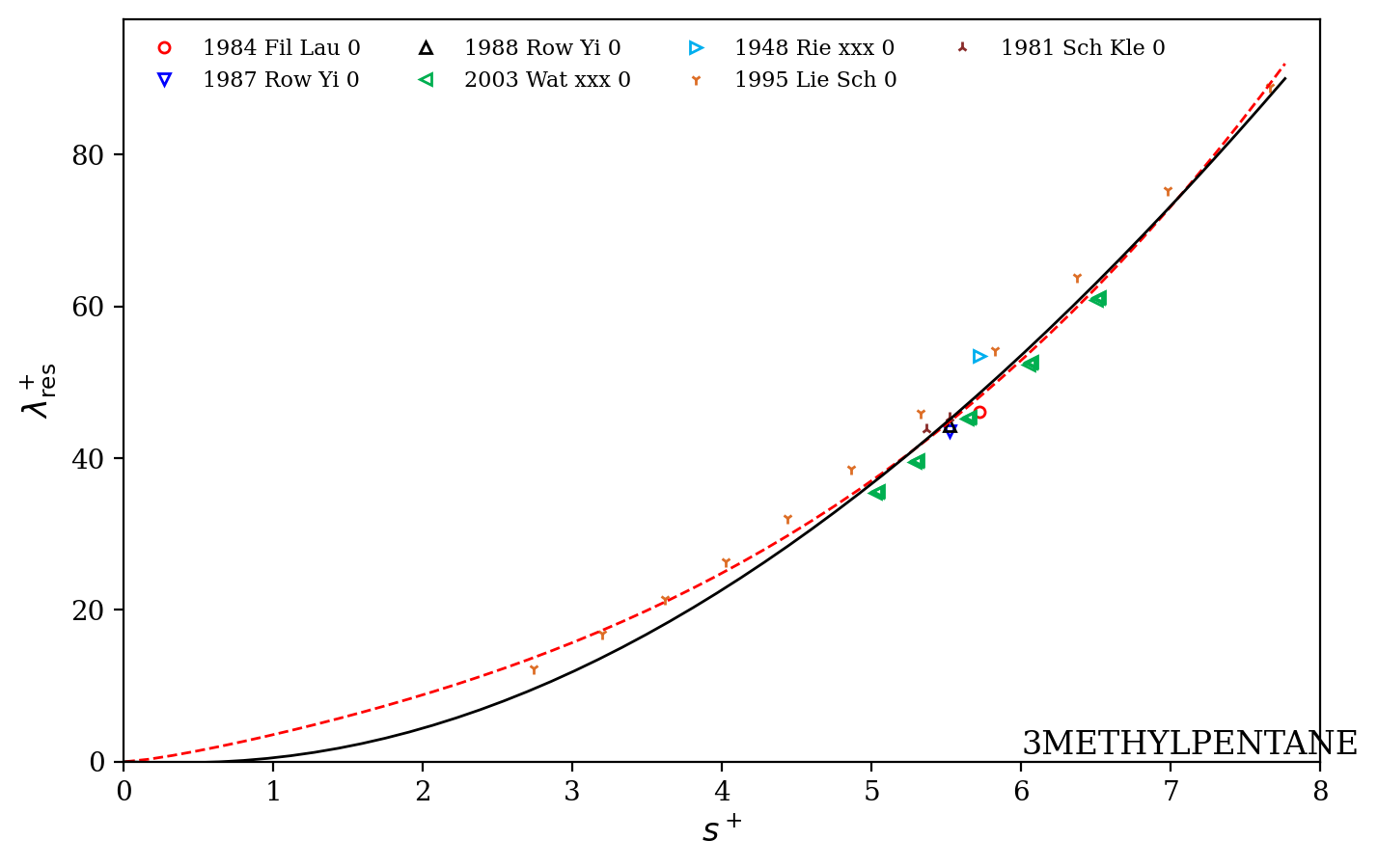

Supplement: Supplementary file 2 — ao4c10815_si_002.zip [file ao4c10815_si_002.zip › Supporting Information/Fig. TC1 - s_plus vs lambda_plus - all data - YFR EoS/3METHYLPENTANE.png]

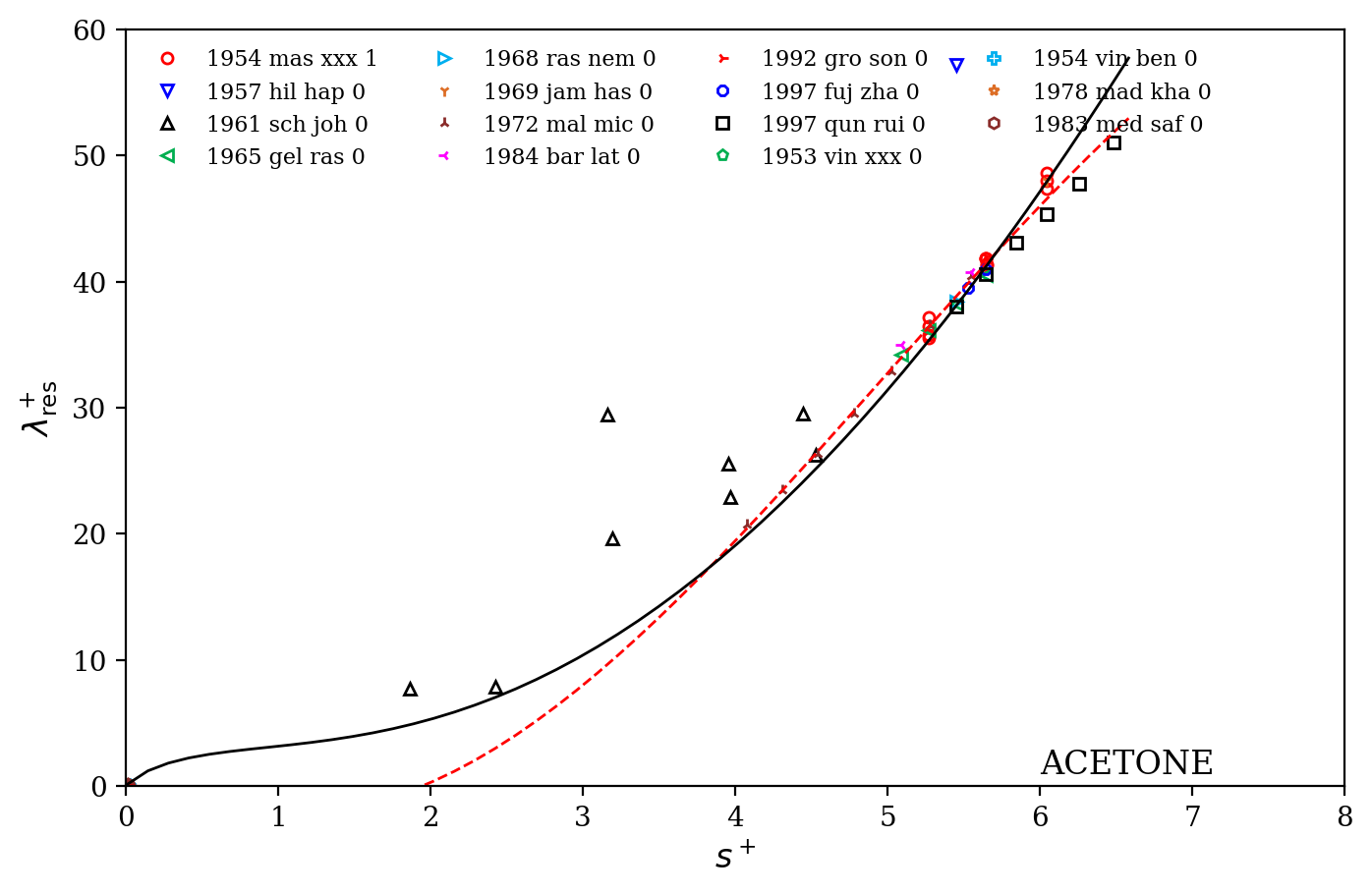

Supplement: Supplementary file 2 — ao4c10815_si_002.zip [file ao4c10815_si_002.zip › Supporting Information/Fig. TC1 - s_plus vs lambda_plus - all data - YFR EoS/ACETONE.png]

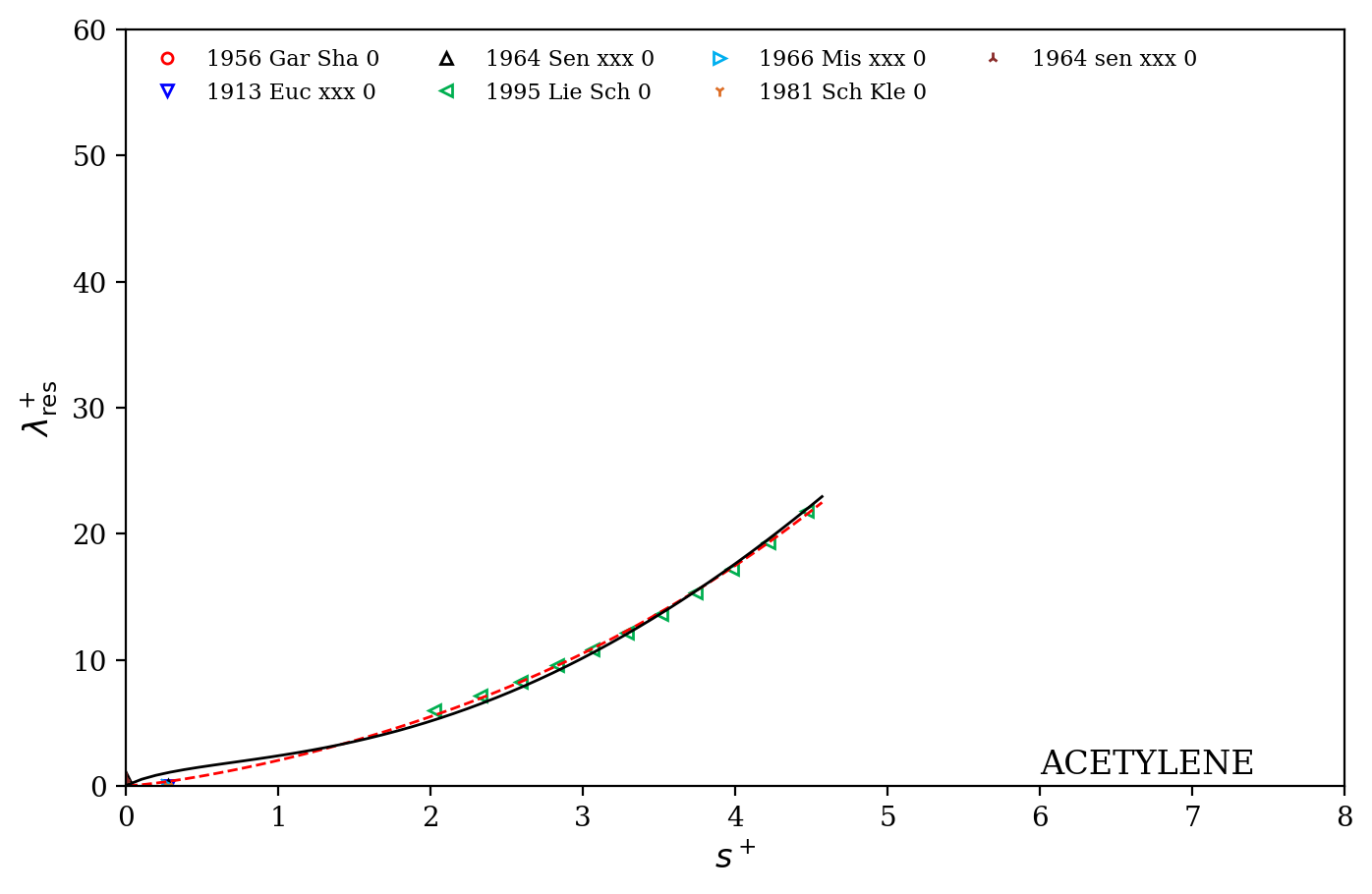

Supplement: Supplementary file 2 — ao4c10815_si_002.zip [file ao4c10815_si_002.zip › Supporting Information/Fig. TC1 - s_plus vs lambda_plus - all data - YFR EoS/ACETYLENE.png]

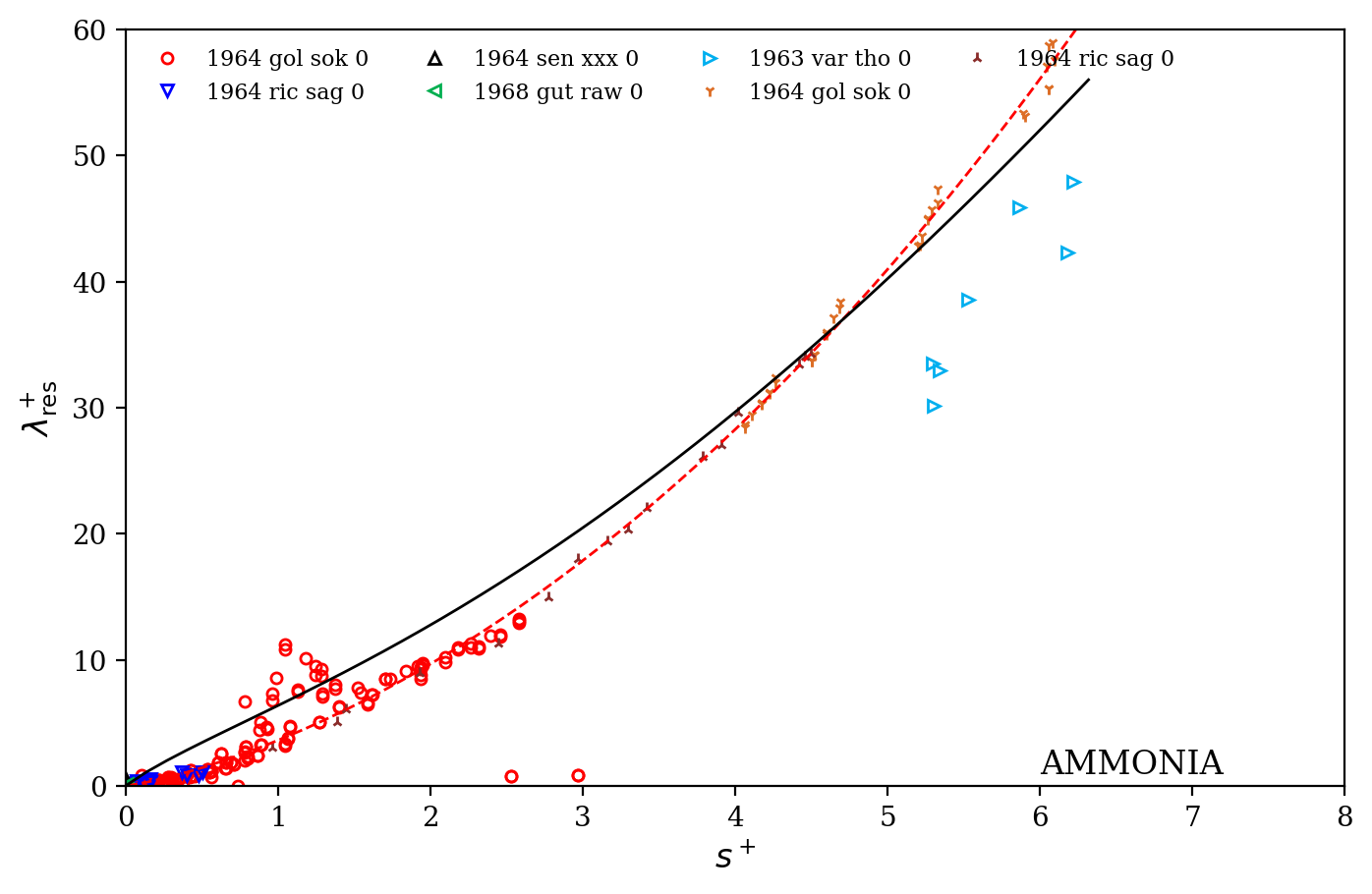

Supplement: Supplementary file 2 — ao4c10815_si_002.zip [file ao4c10815_si_002.zip › Supporting Information/Fig. TC1 - s_plus vs lambda_plus - all data - YFR EoS/AMMONIA.png]

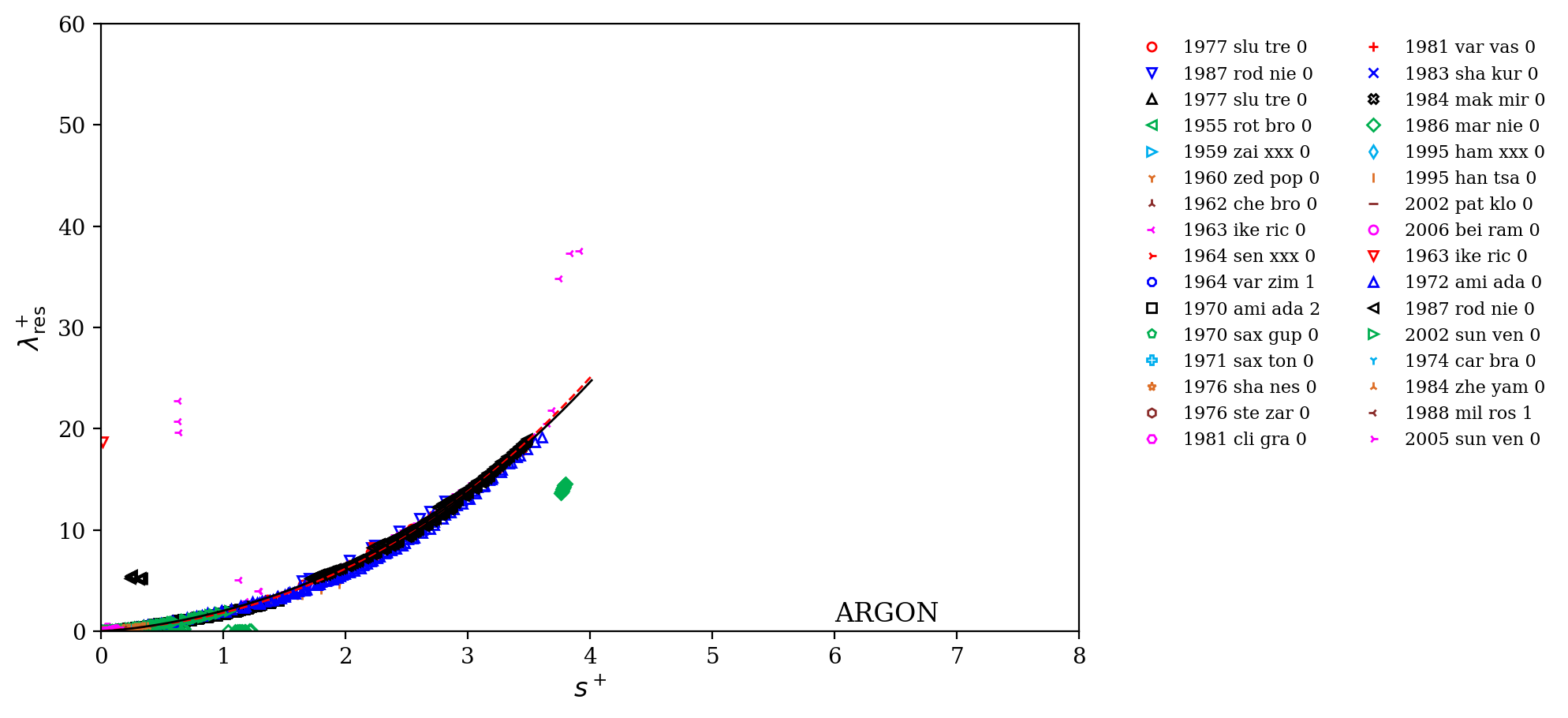

Supplement: Supplementary file 2 — ao4c10815_si_002.zip [file ao4c10815_si_002.zip › Supporting Information/Fig. TC1 - s_plus vs lambda_plus - all data - YFR EoS/ARGON.png]

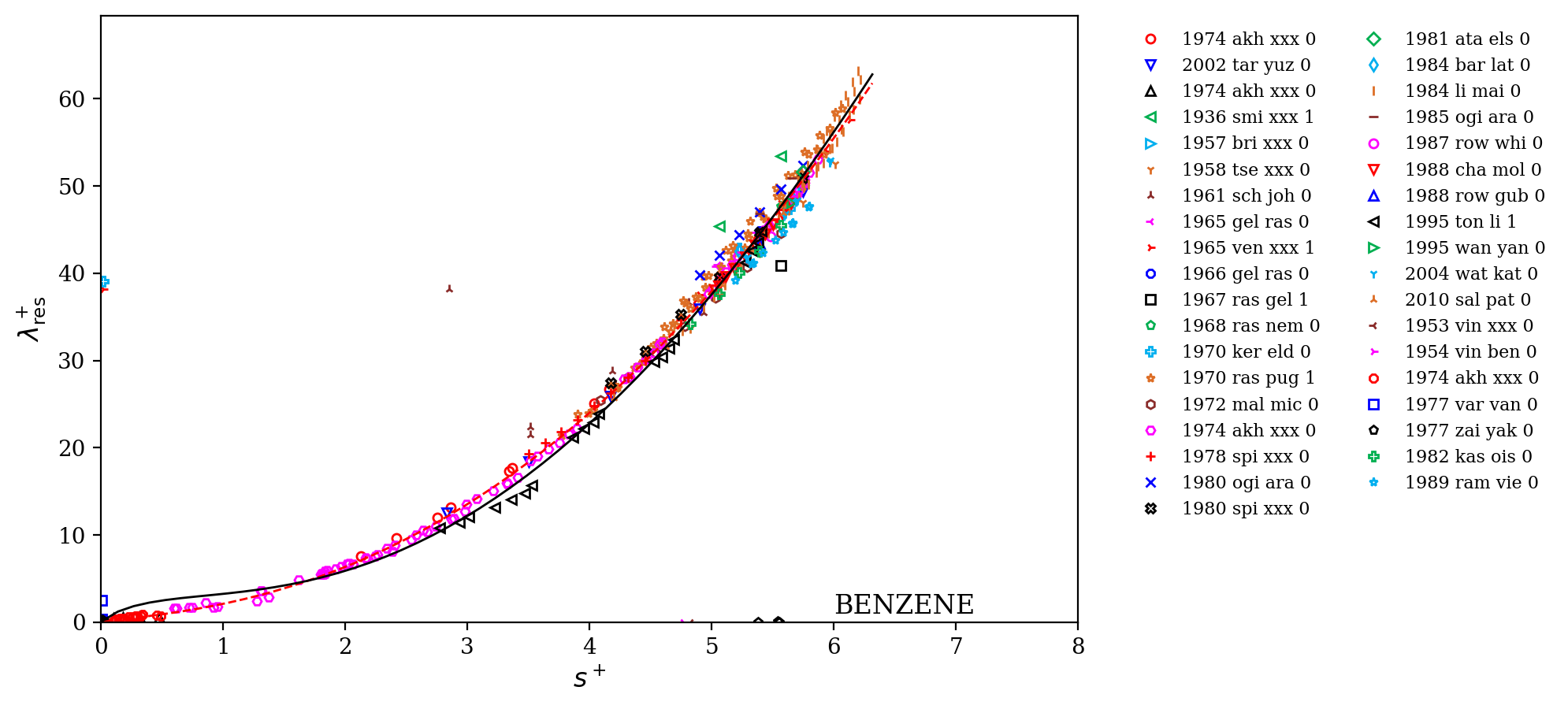

Supplement: Supplementary file 2 — ao4c10815_si_002.zip [file ao4c10815_si_002.zip › Supporting Information/Fig. TC1 - s_plus vs lambda_plus - all data - YFR EoS/BENZENE.png]

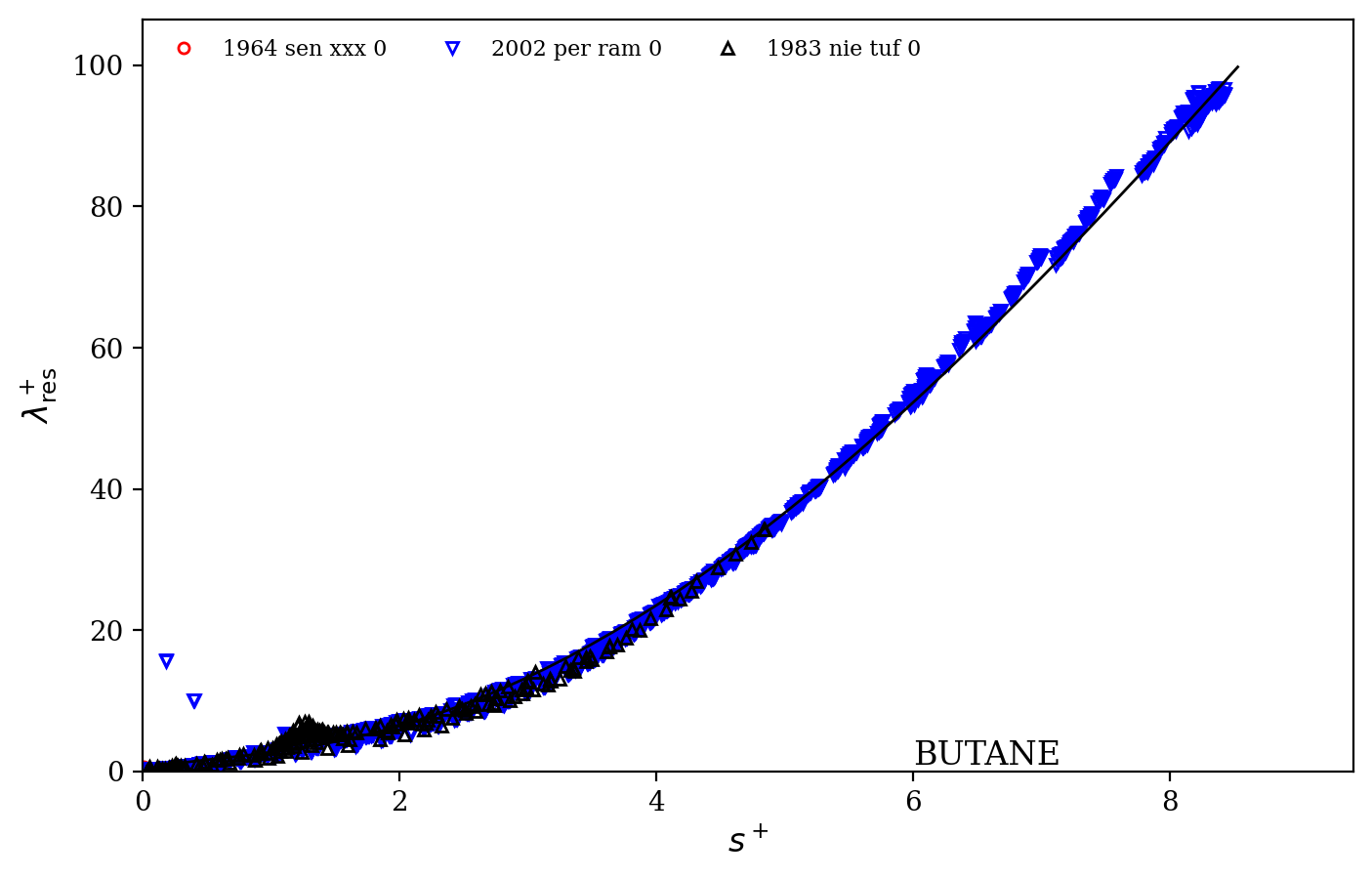

Supplement: Supplementary file 2 — ao4c10815_si_002.zip [file ao4c10815_si_002.zip › Supporting Information/Fig. TC1 - s_plus vs lambda_plus - all data - YFR EoS/BUTANE.png]

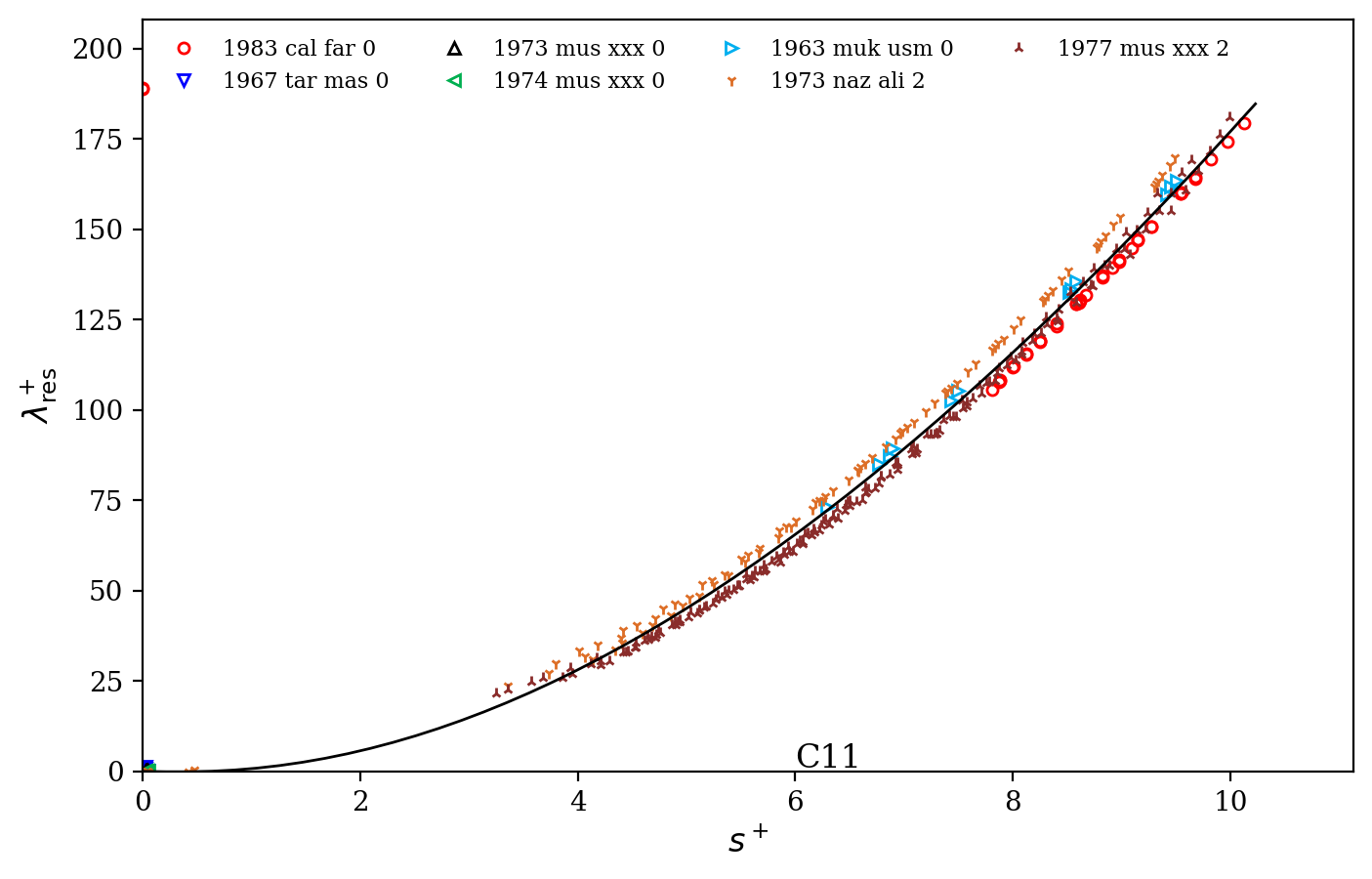

Supplement: Supplementary file 2 — ao4c10815_si_002.zip [file ao4c10815_si_002.zip › Supporting Information/Fig. TC1 - s_plus vs lambda_plus - all data - YFR EoS/C11.png]

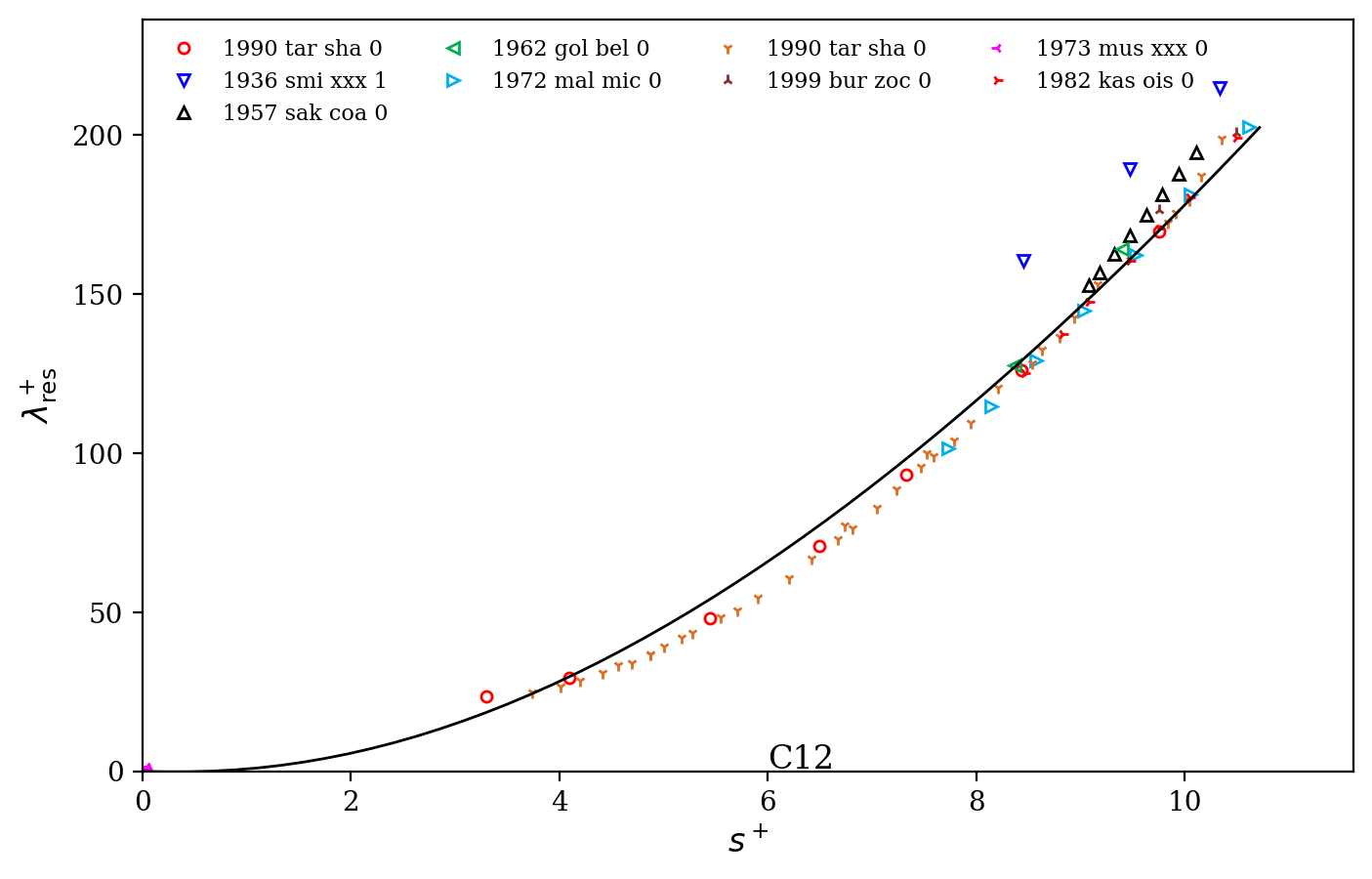

Supplement: Supplementary file 2 — ao4c10815_si_002.zip [file ao4c10815_si_002.zip › Supporting Information/Fig. TC1 - s_plus vs lambda_plus - all data - YFR EoS/C12.png]

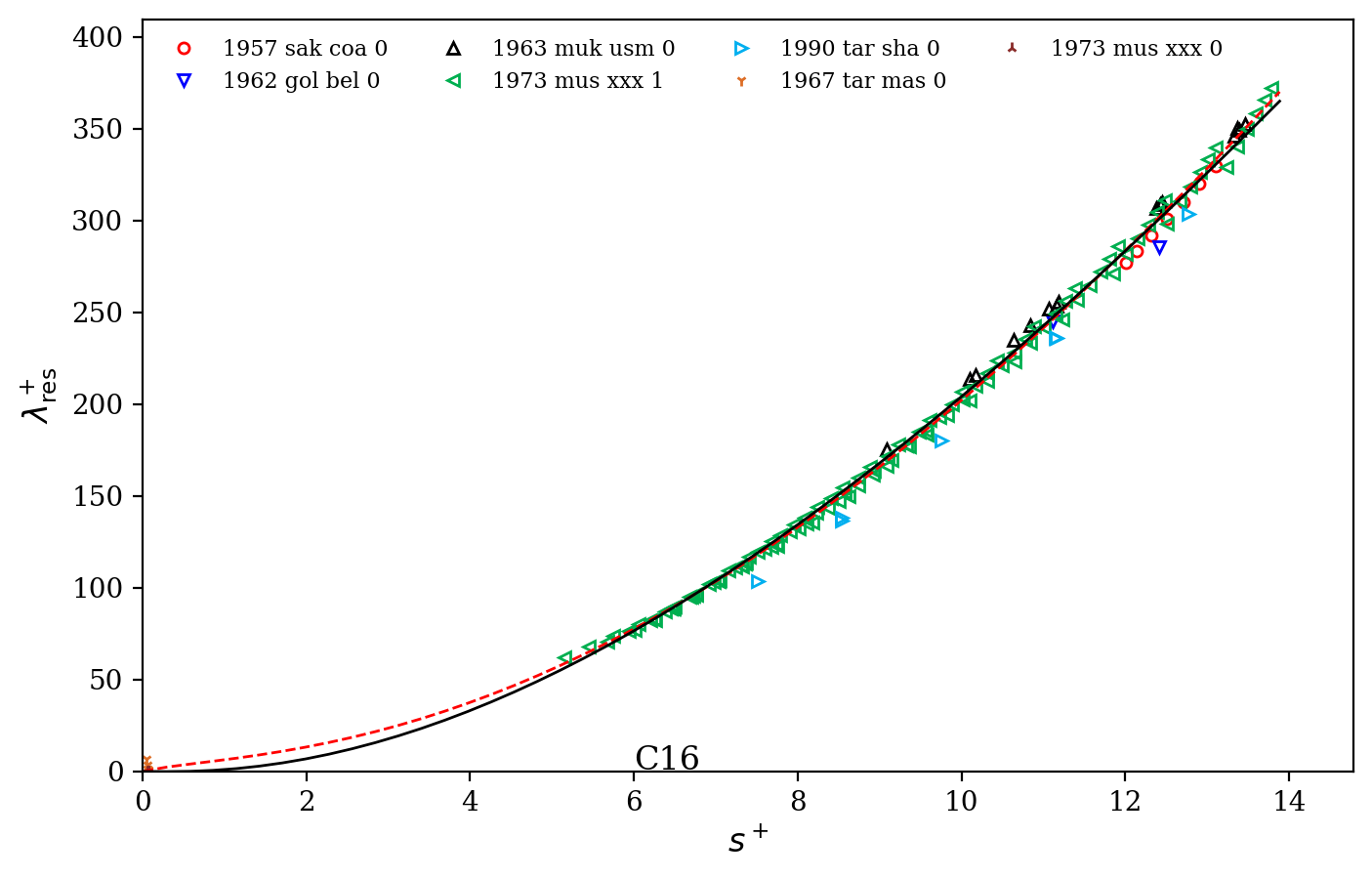

Supplement: Supplementary file 2 — ao4c10815_si_002.zip [file ao4c10815_si_002.zip › Supporting Information/Fig. TC1 - s_plus vs lambda_plus - all data - YFR EoS/C16.png]

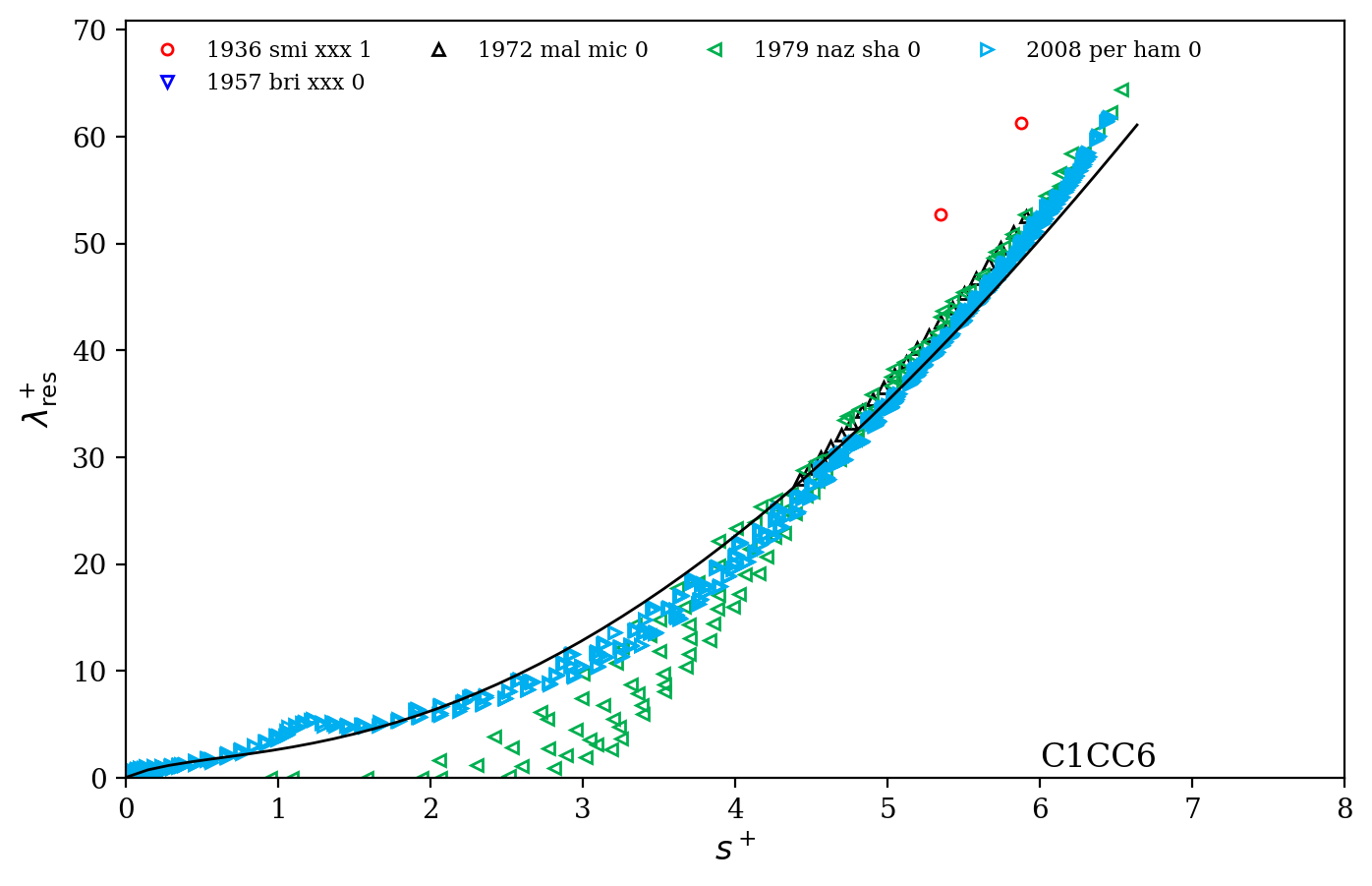

Supplement: Supplementary file 2 — ao4c10815_si_002.zip [file ao4c10815_si_002.zip › Supporting Information/Fig. TC1 - s_plus vs lambda_plus - all data - YFR EoS/C1CC6.png]

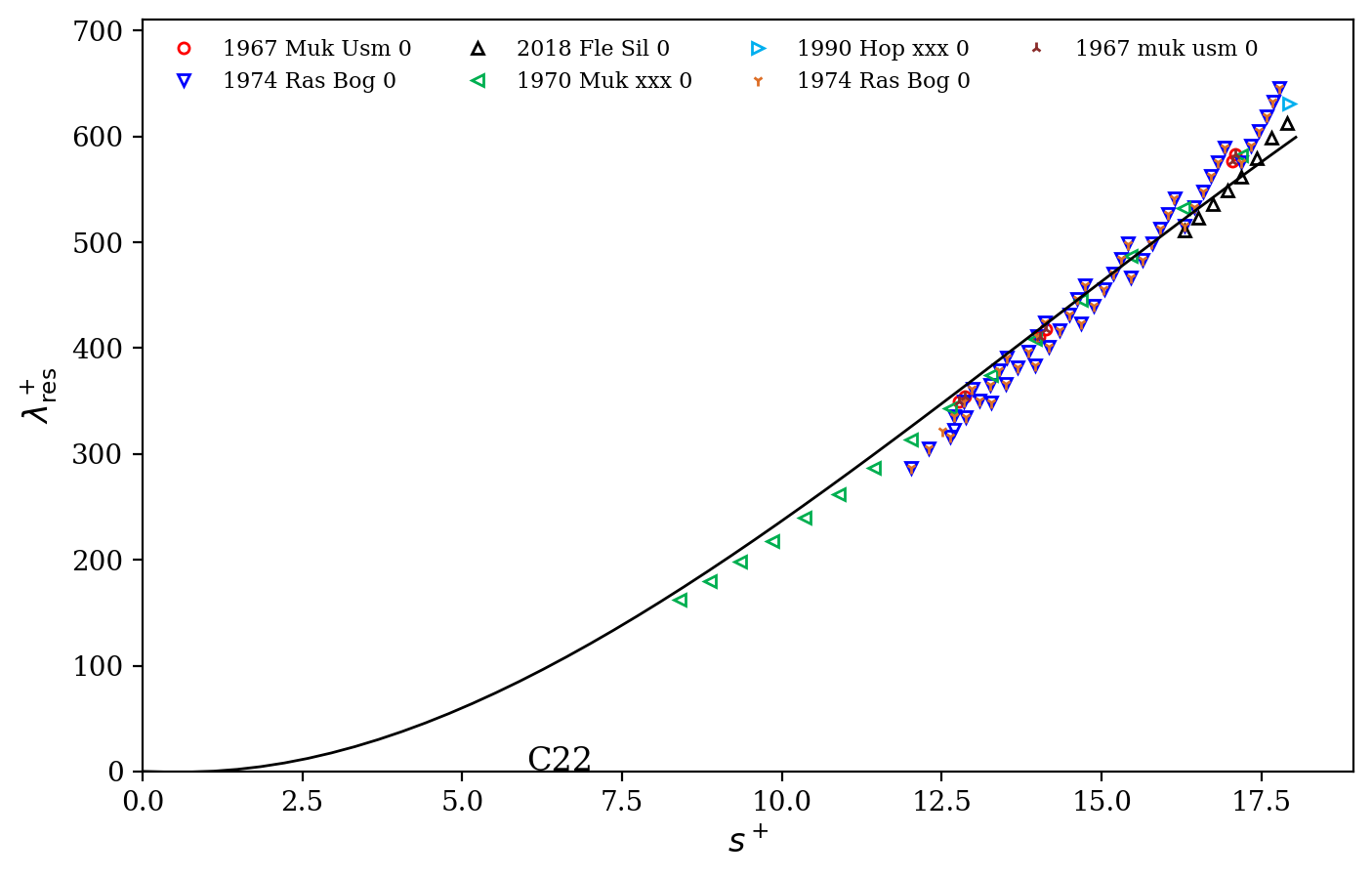

Supplement: Supplementary file 2 — ao4c10815_si_002.zip [file ao4c10815_si_002.zip › Supporting Information/Fig. TC1 - s_plus vs lambda_plus - all data - YFR EoS/C22.png]

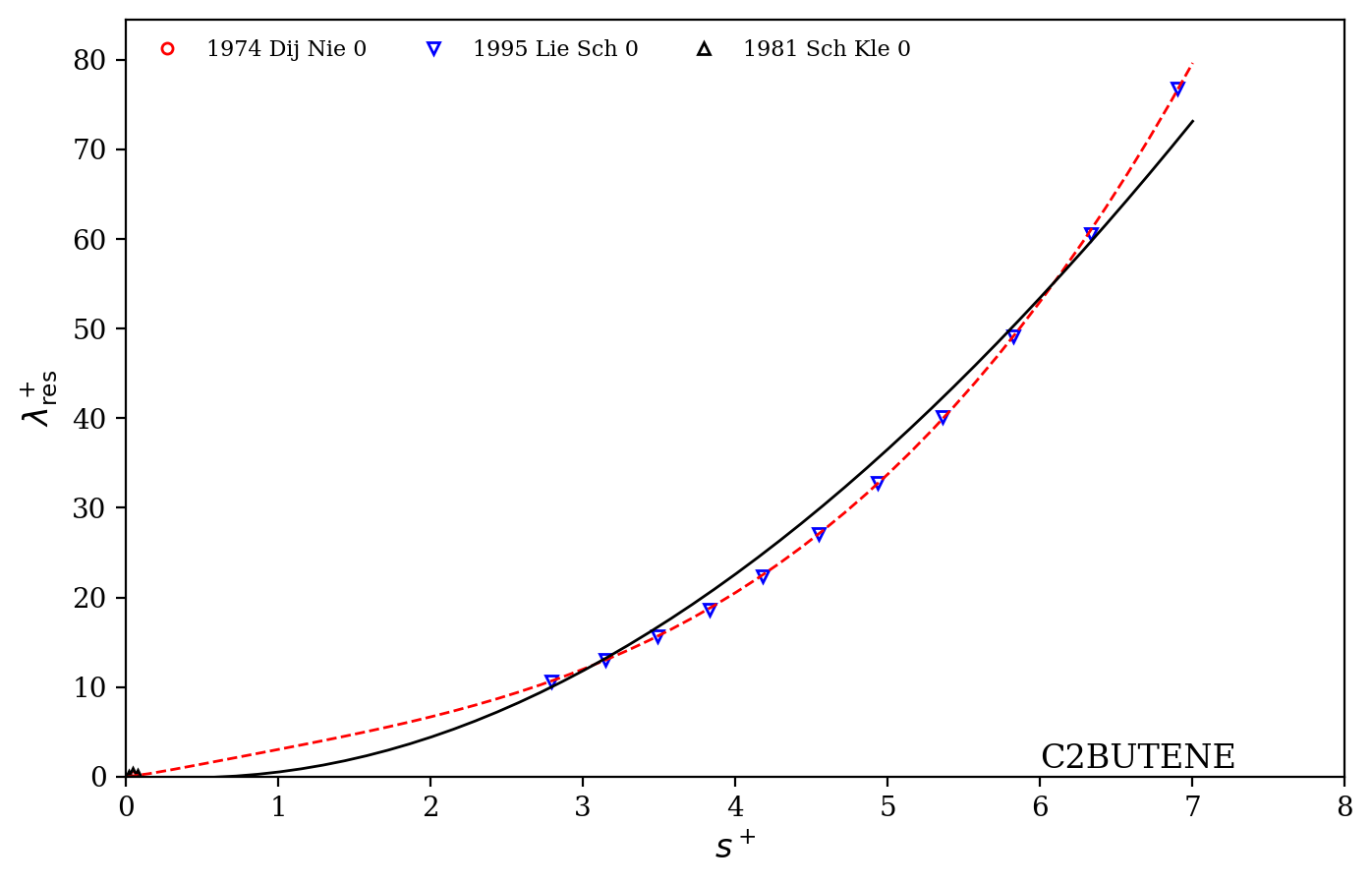

Supplement: Supplementary file 2 — ao4c10815_si_002.zip [file ao4c10815_si_002.zip › Supporting Information/Fig. TC1 - s_plus vs lambda_plus - all data - YFR EoS/C2BUTENE.png]

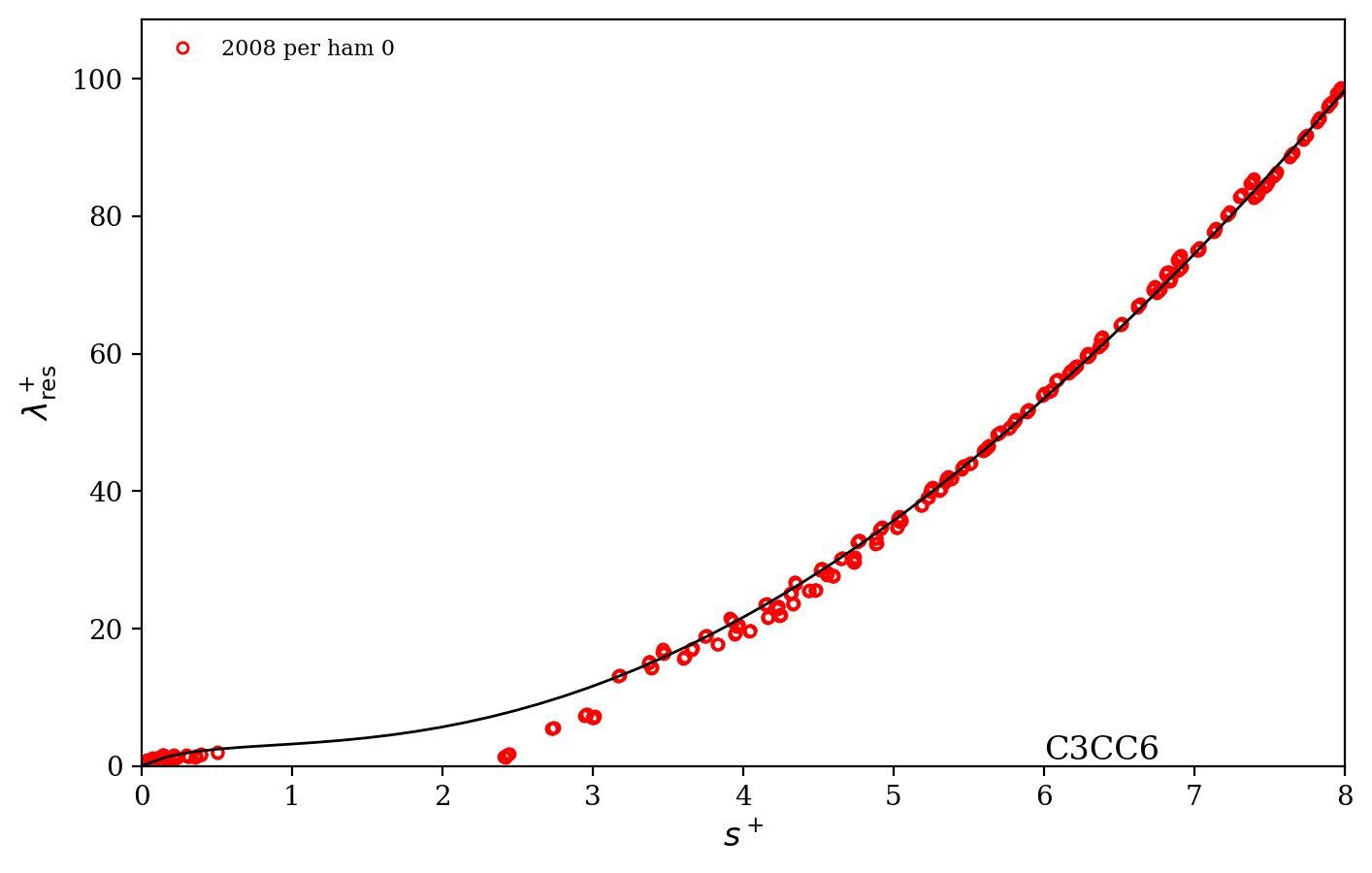

Supplement: Supplementary file 2 — ao4c10815_si_002.zip [file ao4c10815_si_002.zip › Supporting Information/Fig. TC1 - s_plus vs lambda_plus - all data - YFR EoS/C3CC6.png]

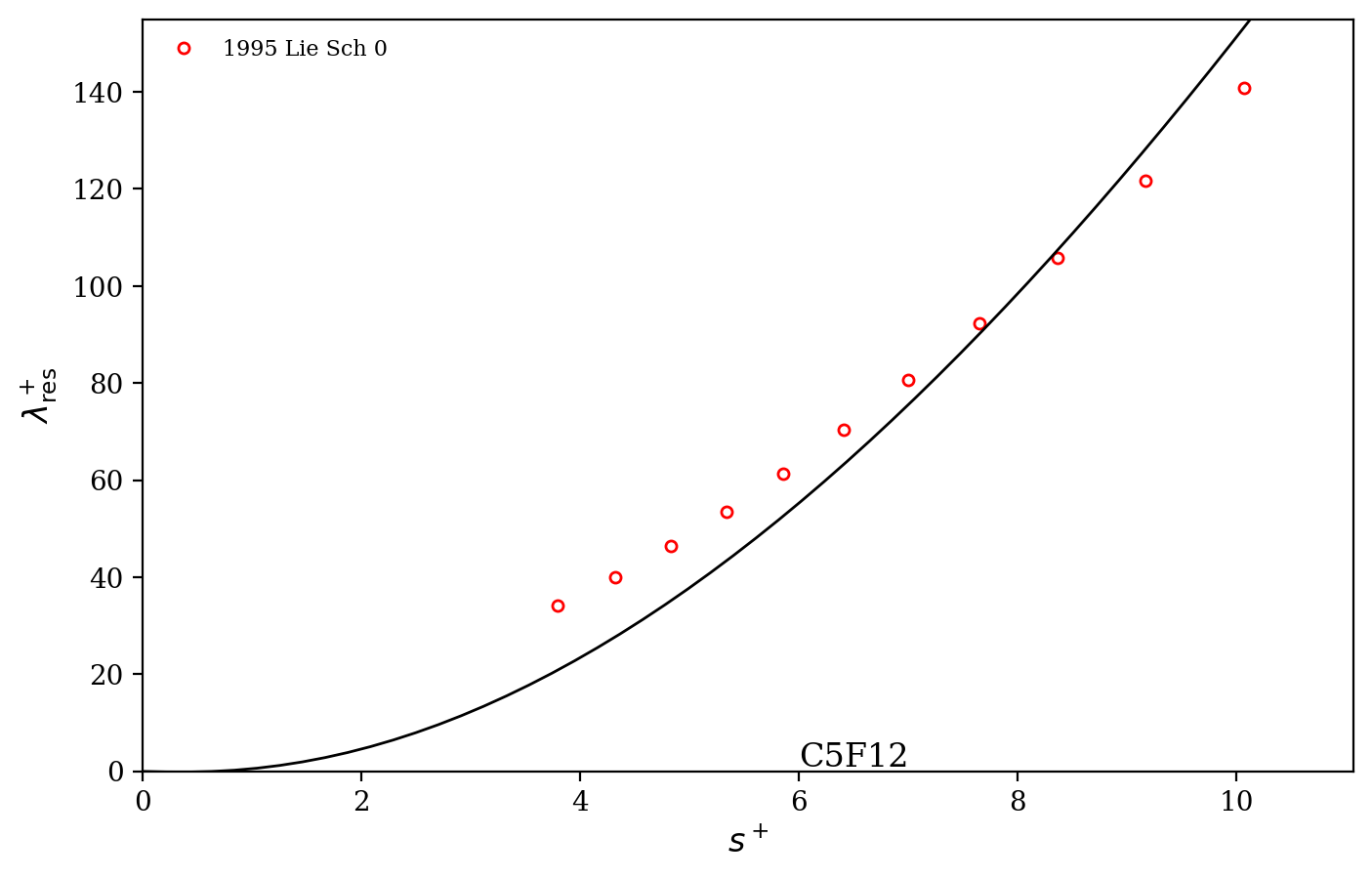

Supplement: Supplementary file 2 — ao4c10815_si_002.zip [file ao4c10815_si_002.zip › Supporting Information/Fig. TC1 - s_plus vs lambda_plus - all data - YFR EoS/C5F12.png]

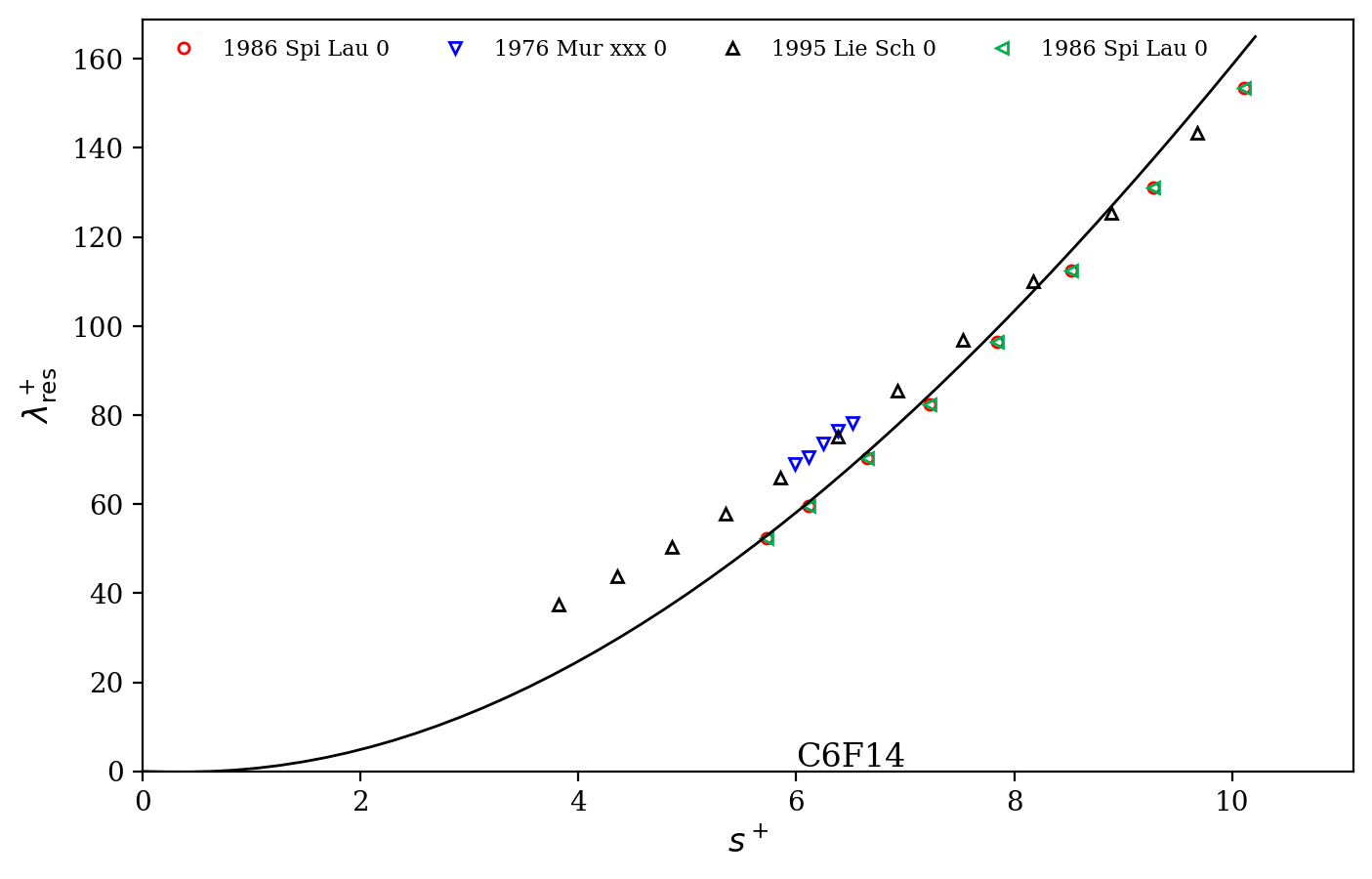

Supplement: Supplementary file 2 — ao4c10815_si_002.zip [file ao4c10815_si_002.zip › Supporting Information/Fig. TC1 - s_plus vs lambda_plus - all data - YFR EoS/C6F14.png]

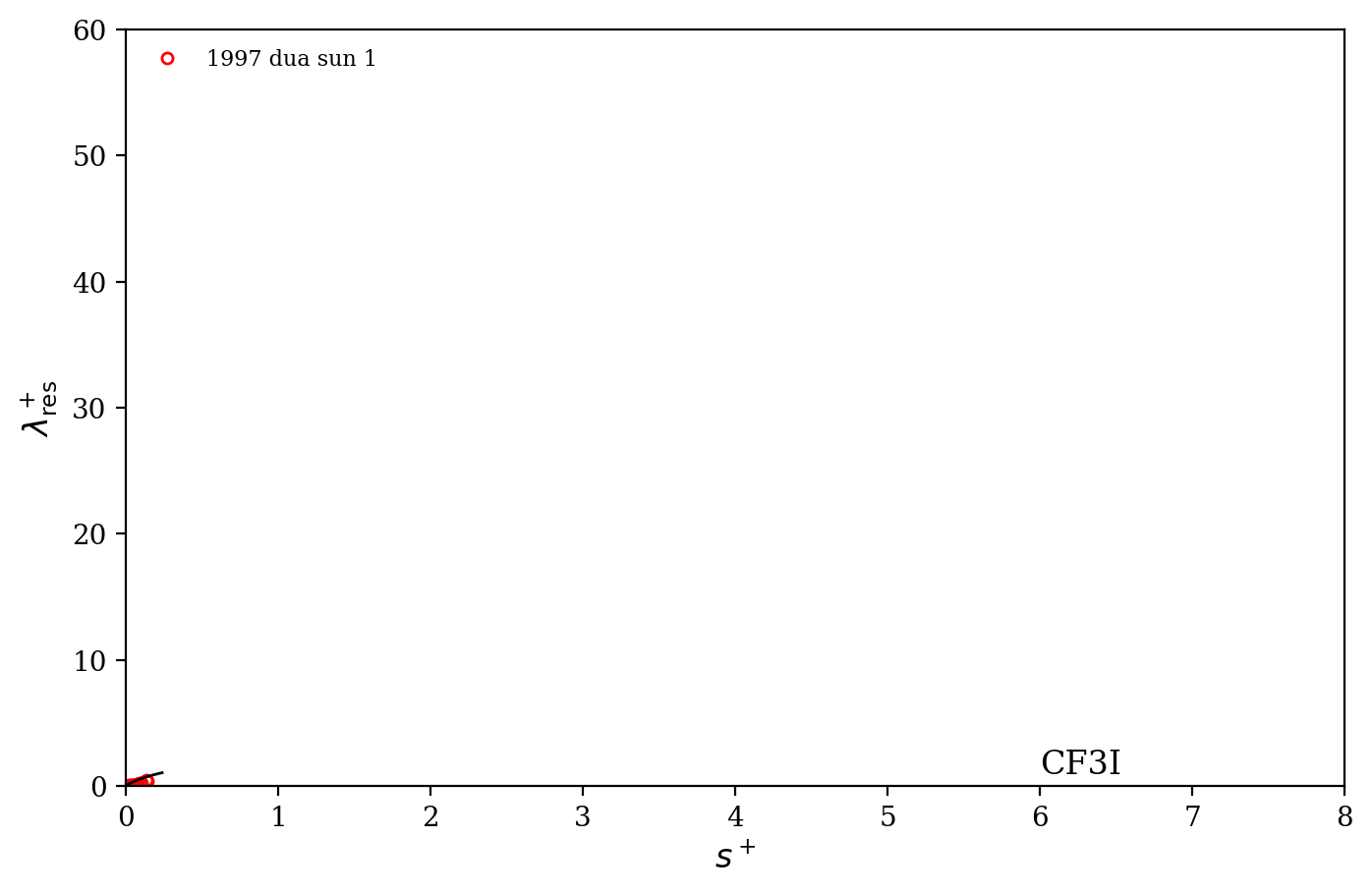

Supplement: Supplementary file 2 — ao4c10815_si_002.zip [file ao4c10815_si_002.zip › Supporting Information/Fig. TC1 - s_plus vs lambda_plus - all data - YFR EoS/CF3I.png]

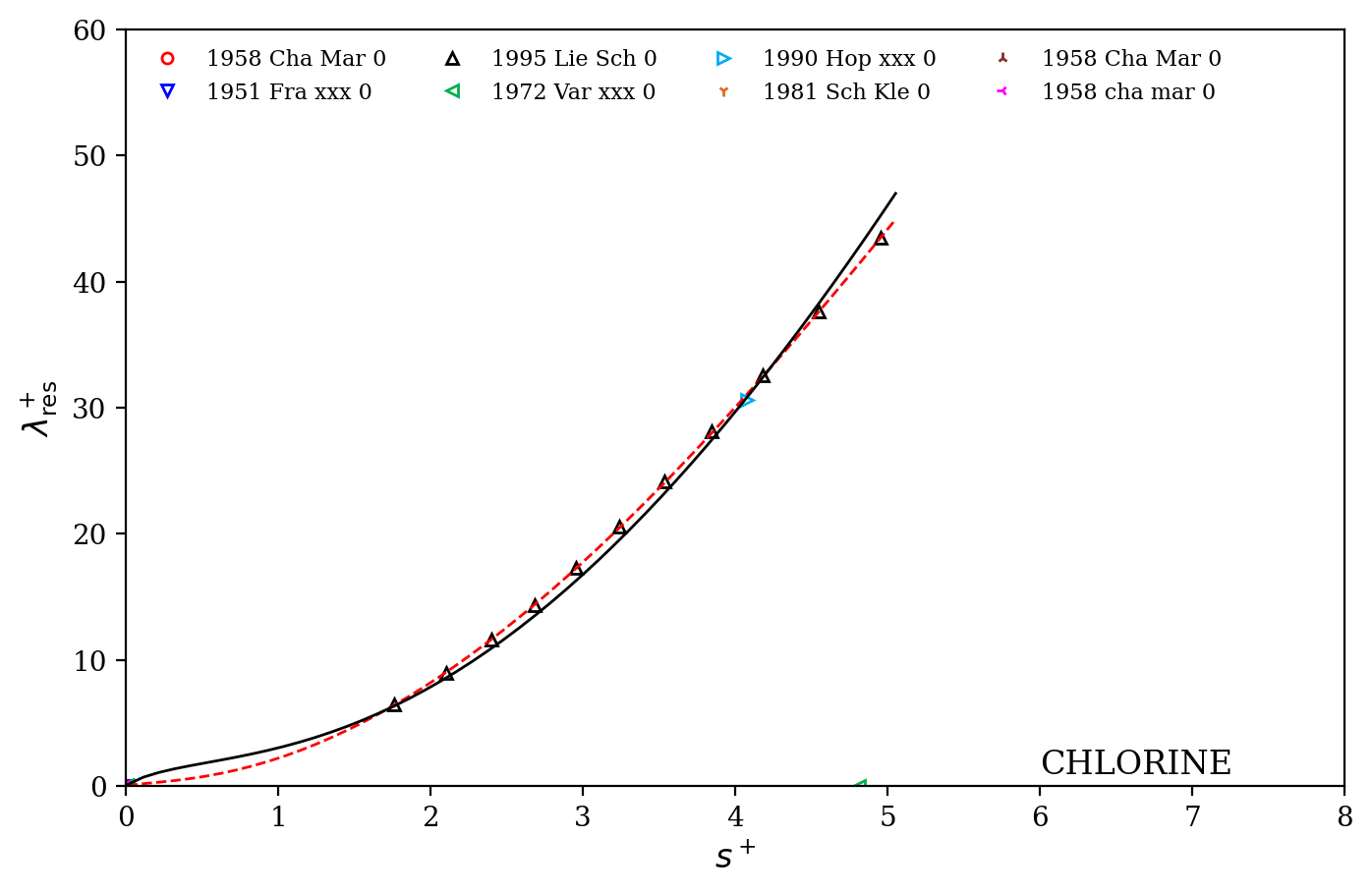

Supplement: Supplementary file 2 — ao4c10815_si_002.zip [file ao4c10815_si_002.zip › Supporting Information/Fig. TC1 - s_plus vs lambda_plus - all data - YFR EoS/CHLORINE.png]

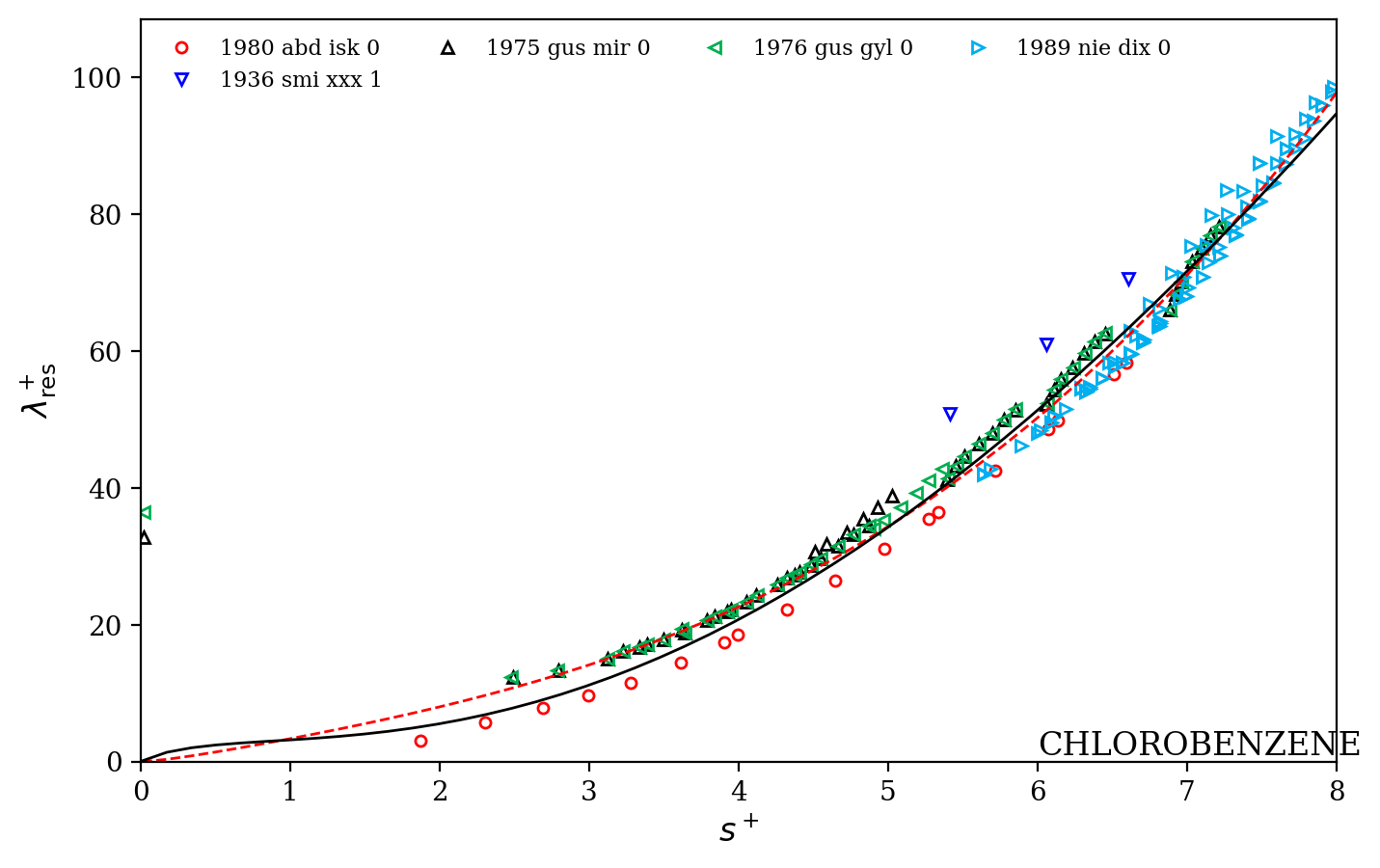

Supplement: Supplementary file 2 — ao4c10815_si_002.zip [file ao4c10815_si_002.zip › Supporting Information/Fig. TC1 - s_plus vs lambda_plus - all data - YFR EoS/CHLOROBENZENE.png]

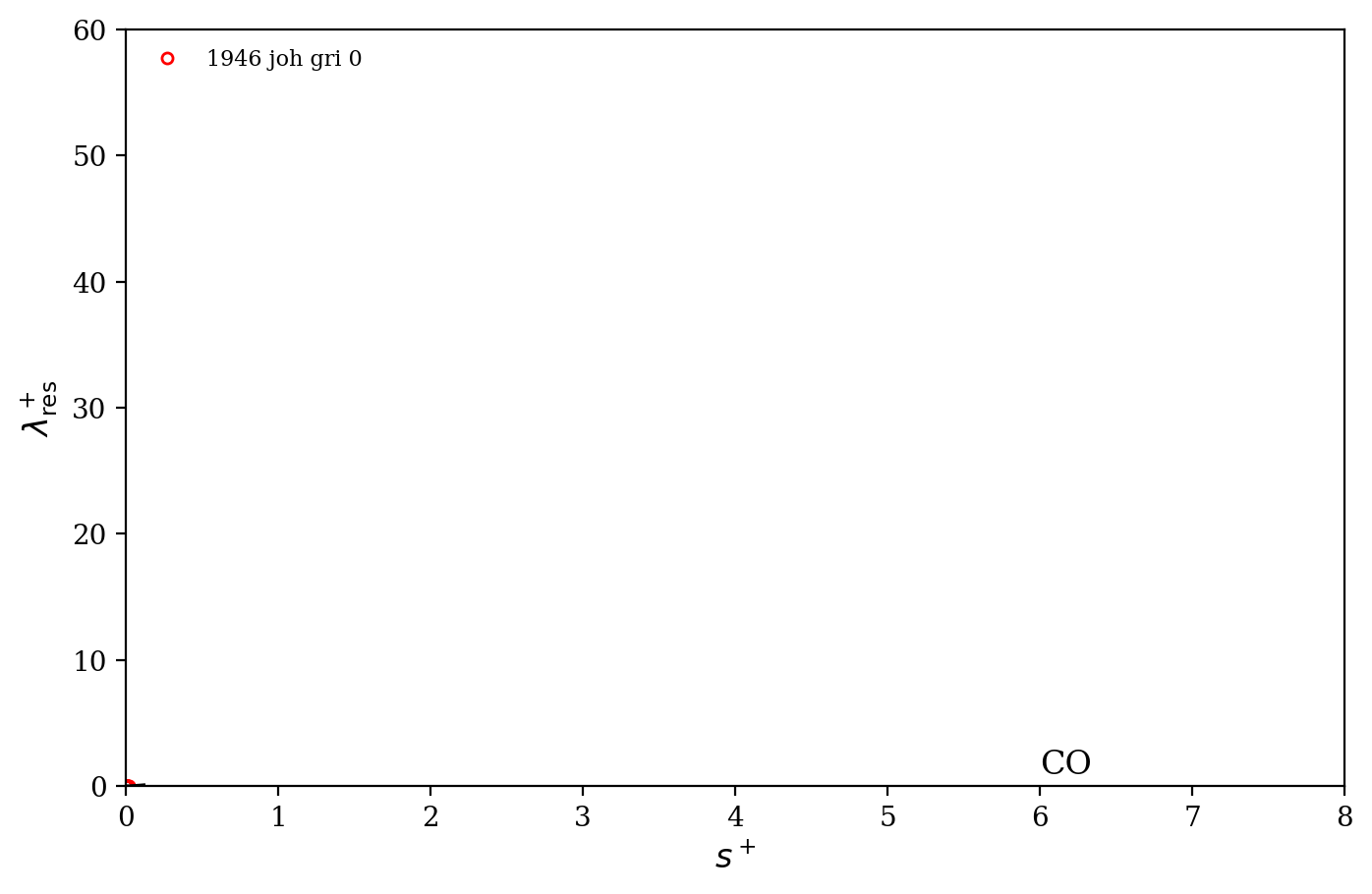

Supplement: Supplementary file 2 — ao4c10815_si_002.zip [file ao4c10815_si_002.zip › Supporting Information/Fig. TC1 - s_plus vs lambda_plus - all data - YFR EoS/CO.png]

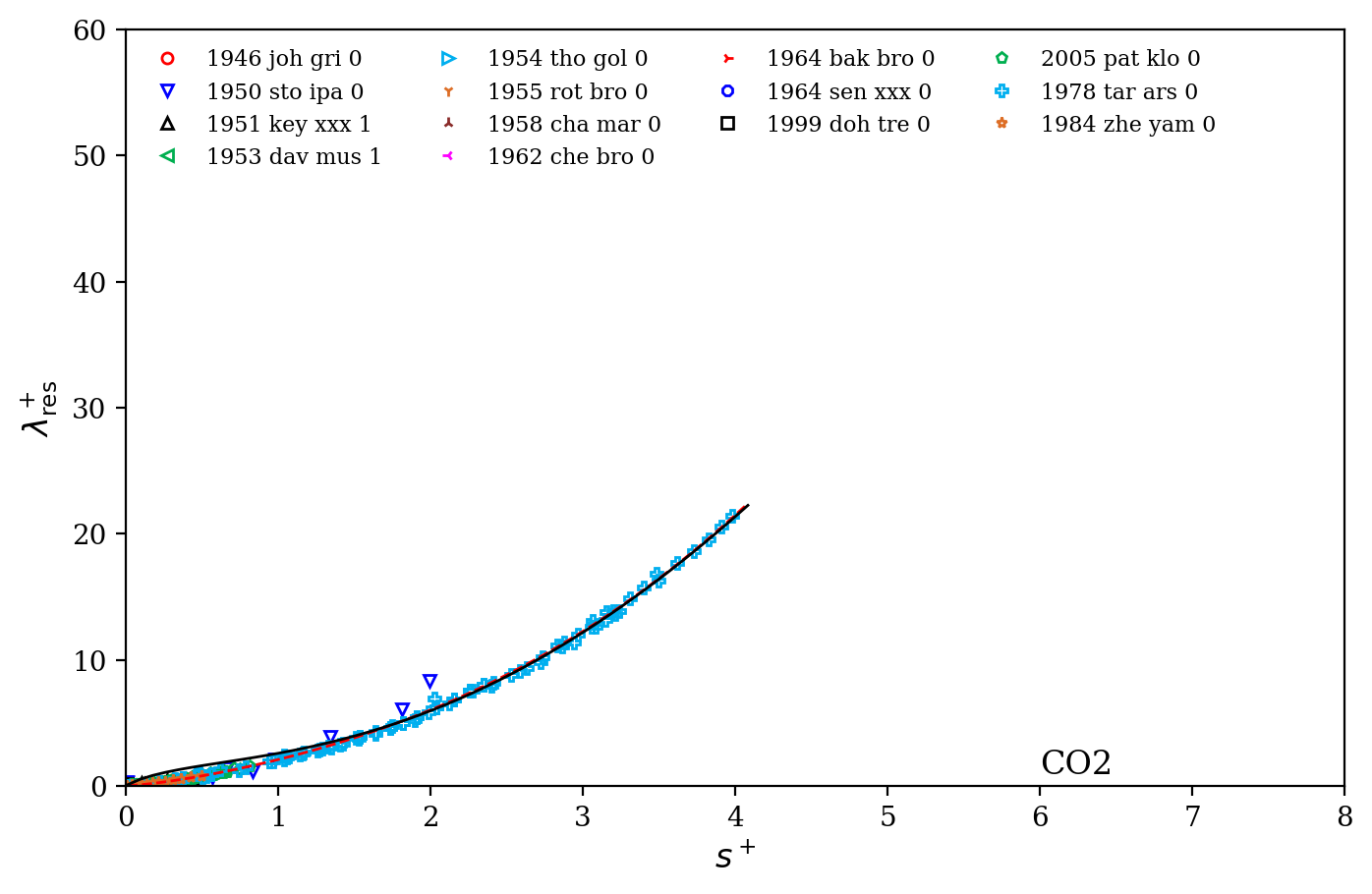

Supplement: Supplementary file 2 — ao4c10815_si_002.zip [file ao4c10815_si_002.zip › Supporting Information/Fig. TC1 - s_plus vs lambda_plus - all data - YFR EoS/CO2.png]

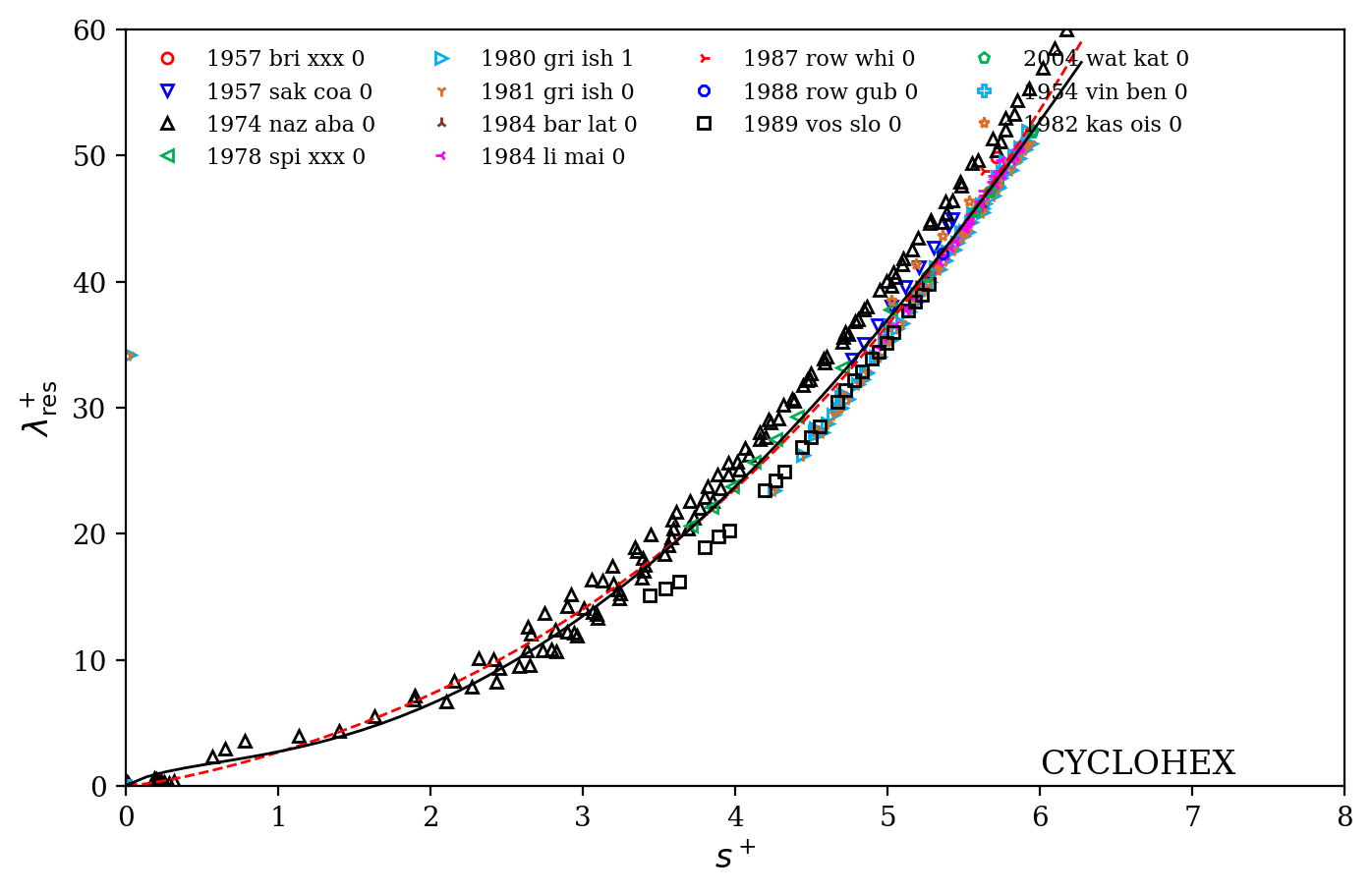

Supplement: Supplementary file 2 — ao4c10815_si_002.zip [file ao4c10815_si_002.zip › Supporting Information/Fig. TC1 - s_plus vs lambda_plus - all data - YFR EoS/CYCLOHEX.png]

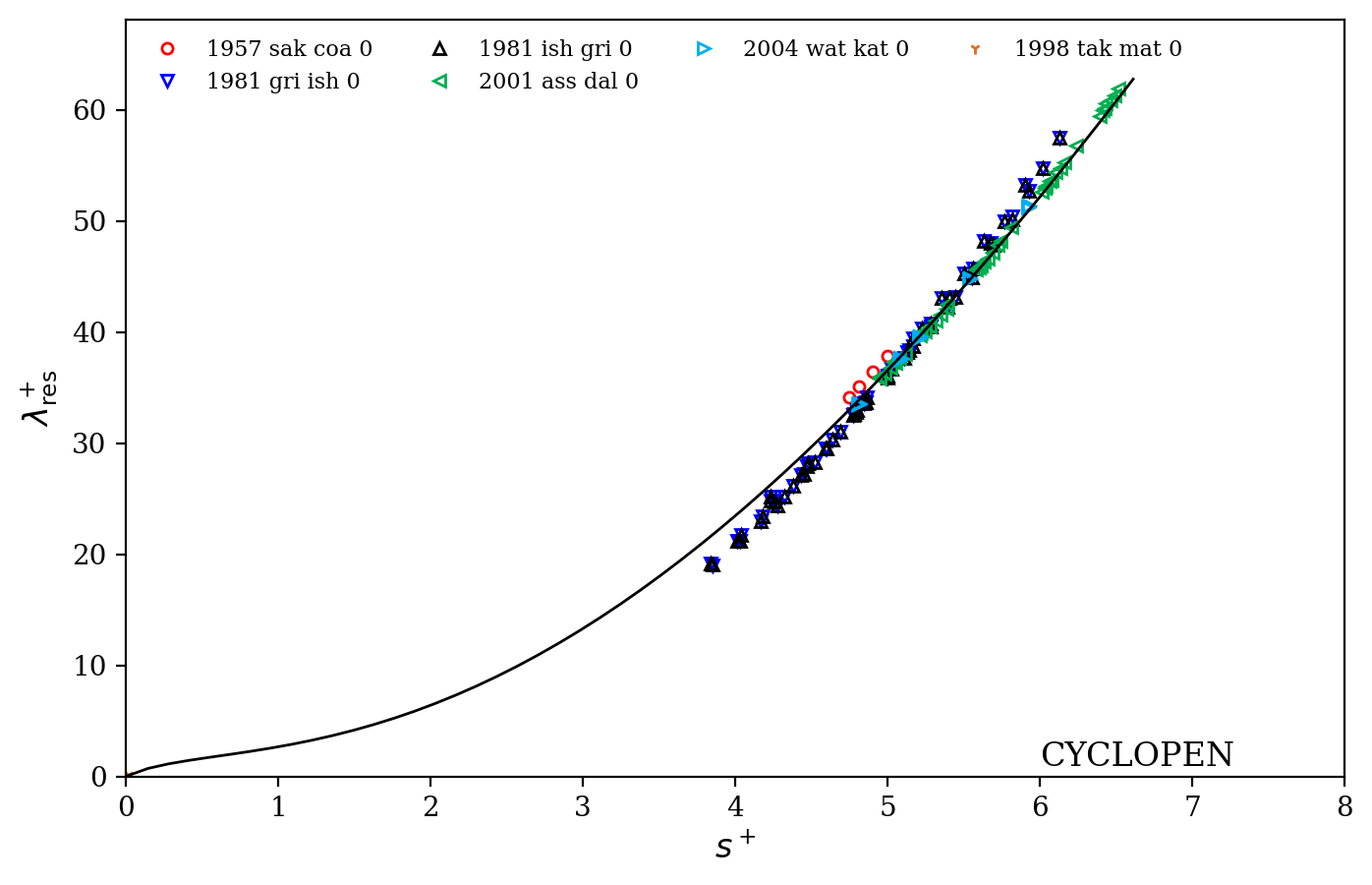

Supplement: Supplementary file 2 — ao4c10815_si_002.zip [file ao4c10815_si_002.zip › Supporting Information/Fig. TC1 - s_plus vs lambda_plus - all data - YFR EoS/CYCLOPEN.png]

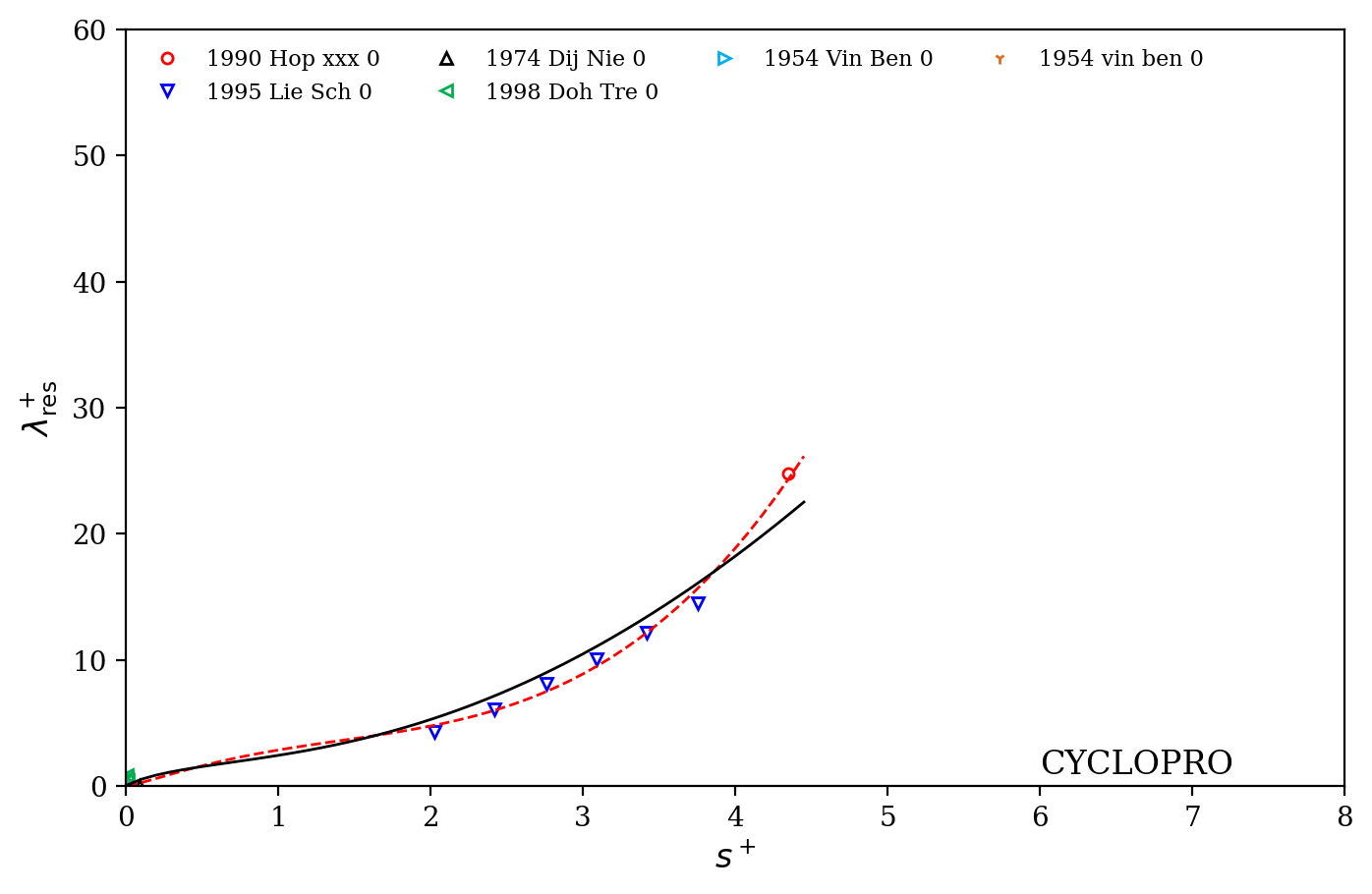

Supplement: Supplementary file 2 — ao4c10815_si_002.zip [file ao4c10815_si_002.zip › Supporting Information/Fig. TC1 - s_plus vs lambda_plus - all data - YFR EoS/CYCLOPRO.png]

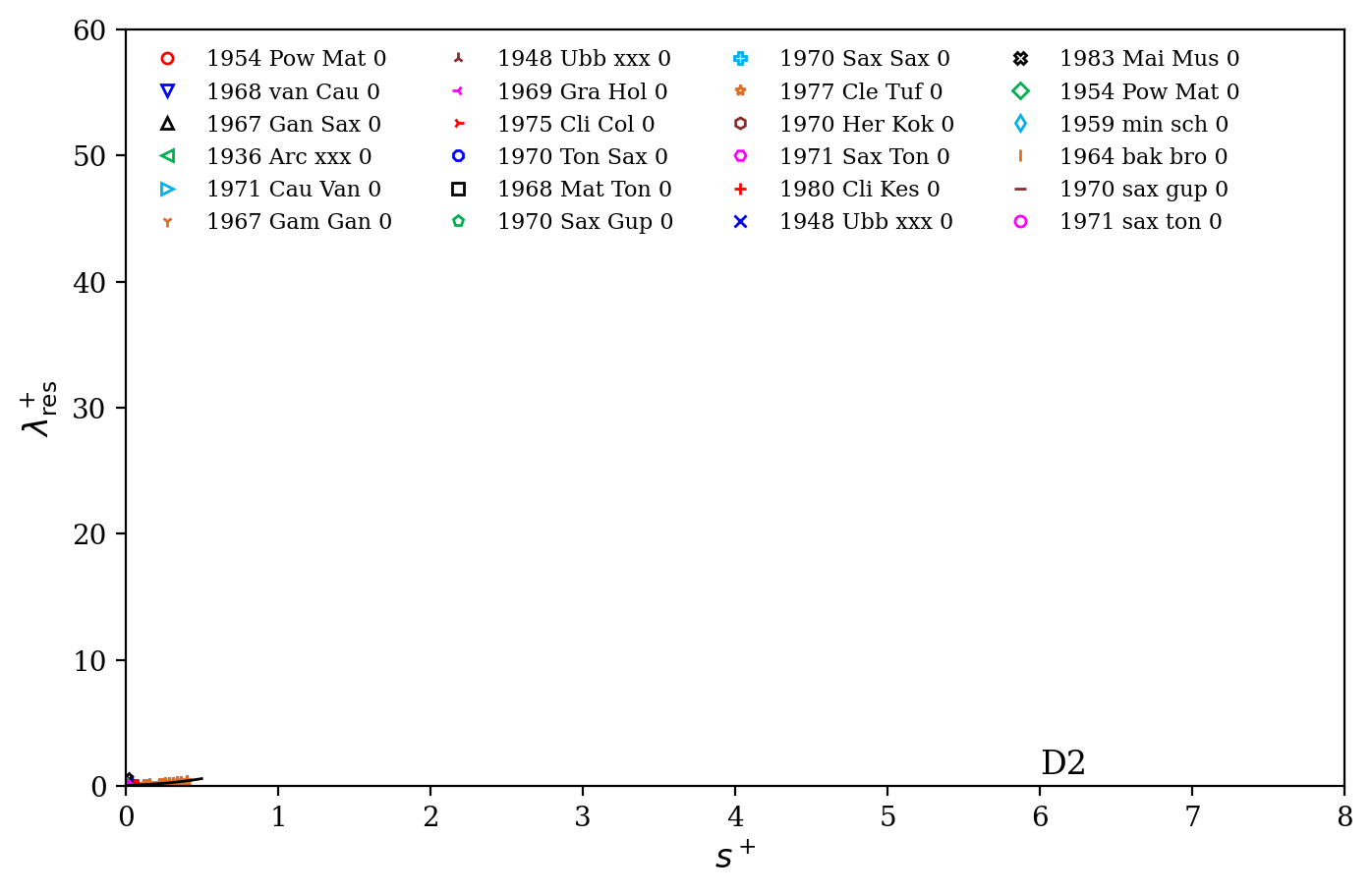

Supplement: Supplementary file 2 — ao4c10815_si_002.zip [file ao4c10815_si_002.zip › Supporting Information/Fig. TC1 - s_plus vs lambda_plus - all data - YFR EoS/D2.png]

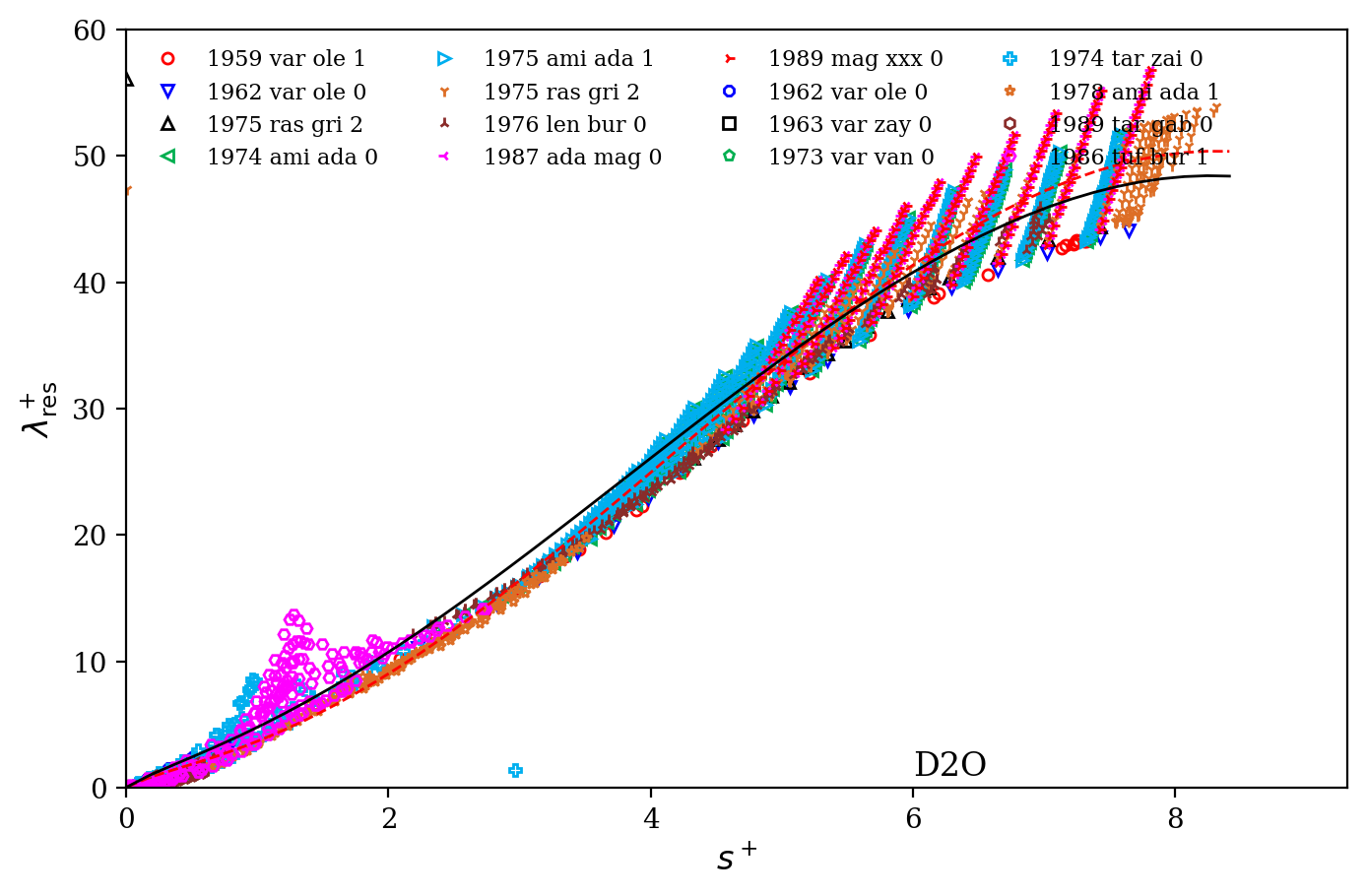

Supplement: Supplementary file 2 — ao4c10815_si_002.zip [file ao4c10815_si_002.zip › Supporting Information/Fig. TC1 - s_plus vs lambda_plus - all data - YFR EoS/D2O.png]

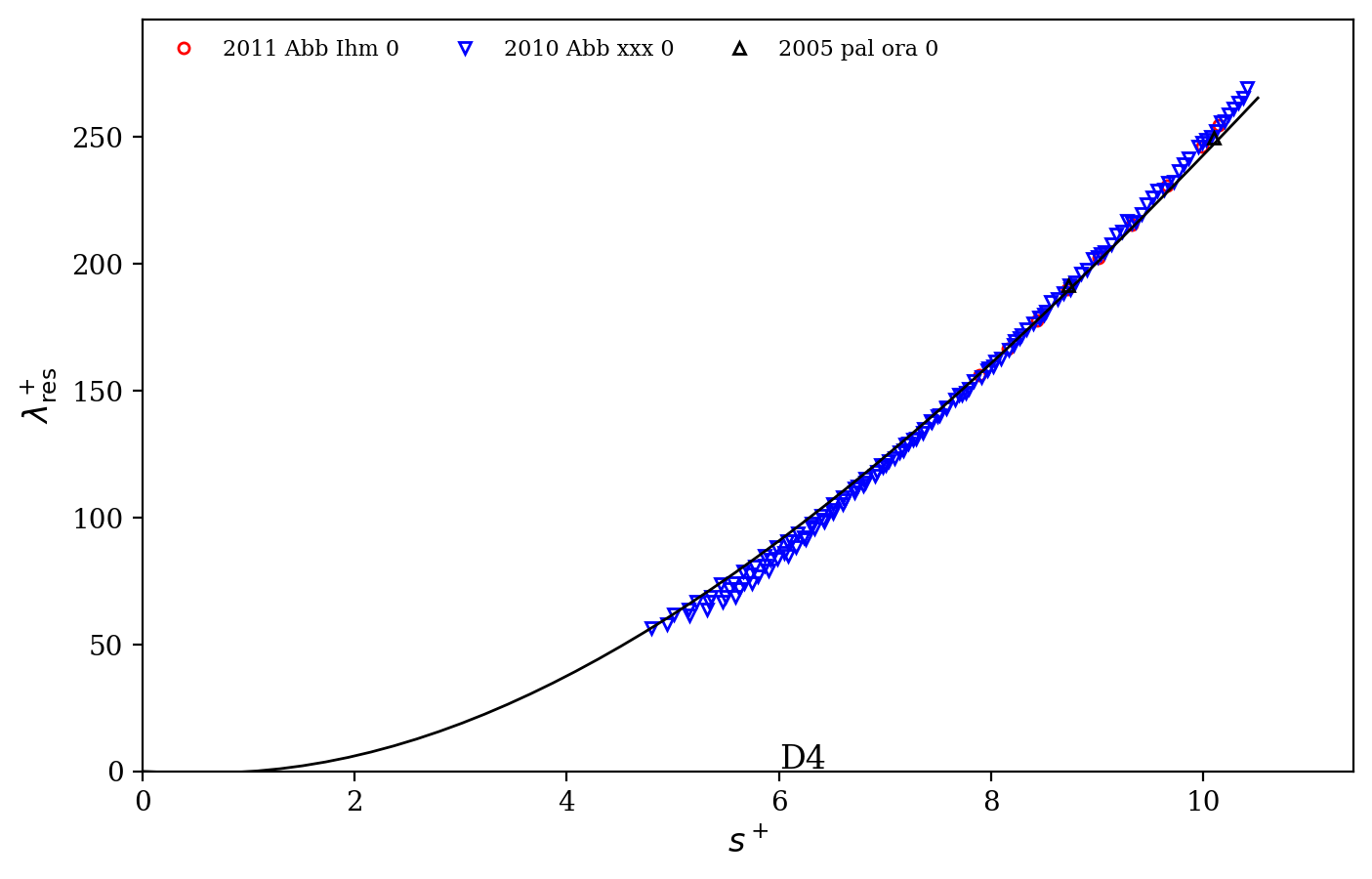

Supplement: Supplementary file 2 — ao4c10815_si_002.zip [file ao4c10815_si_002.zip › Supporting Information/Fig. TC1 - s_plus vs lambda_plus - all data - YFR EoS/D4.png]

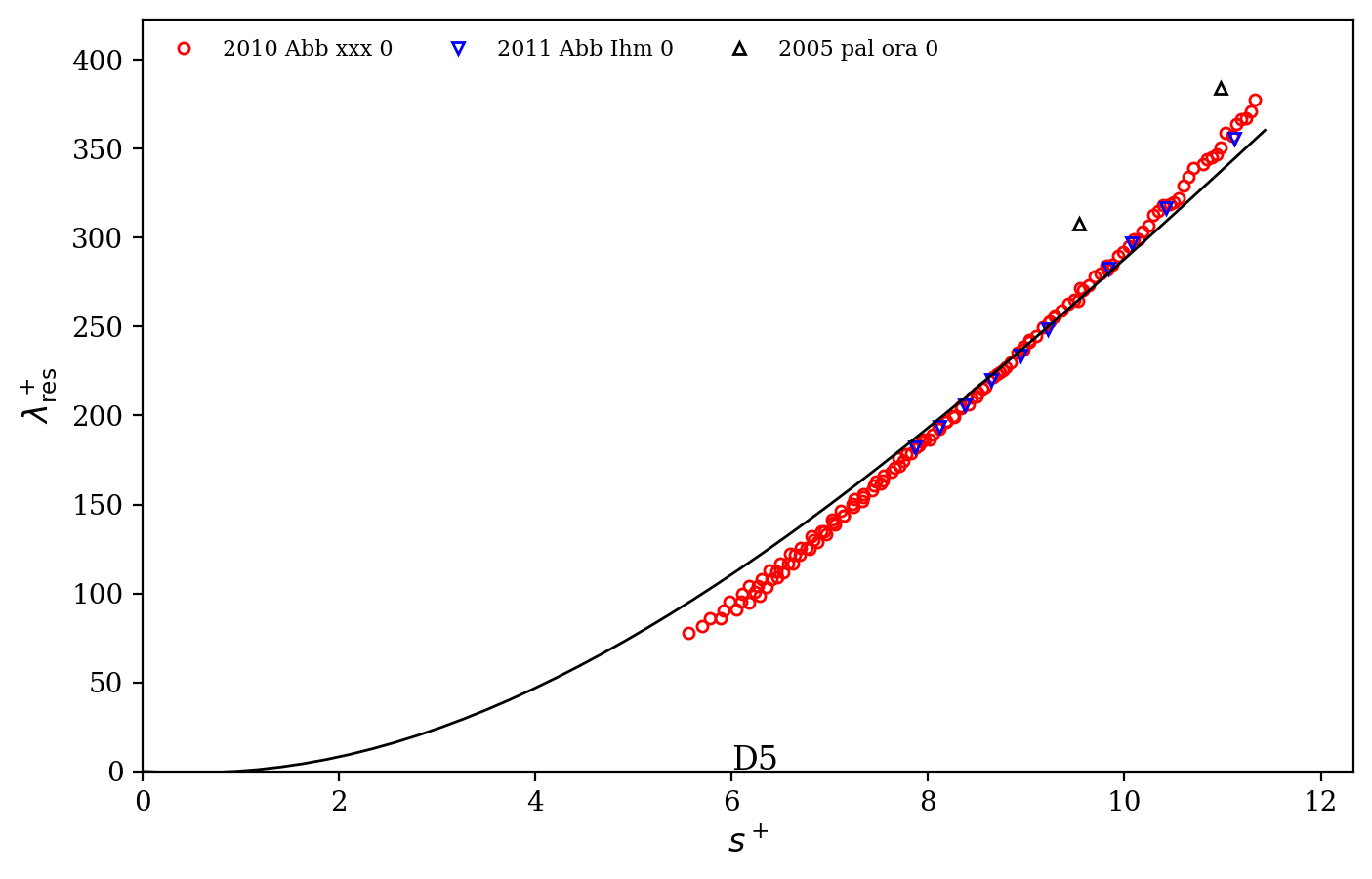

Supplement: Supplementary file 2 — ao4c10815_si_002.zip [file ao4c10815_si_002.zip › Supporting Information/Fig. TC1 - s_plus vs lambda_plus - all data - YFR EoS/D5.png]

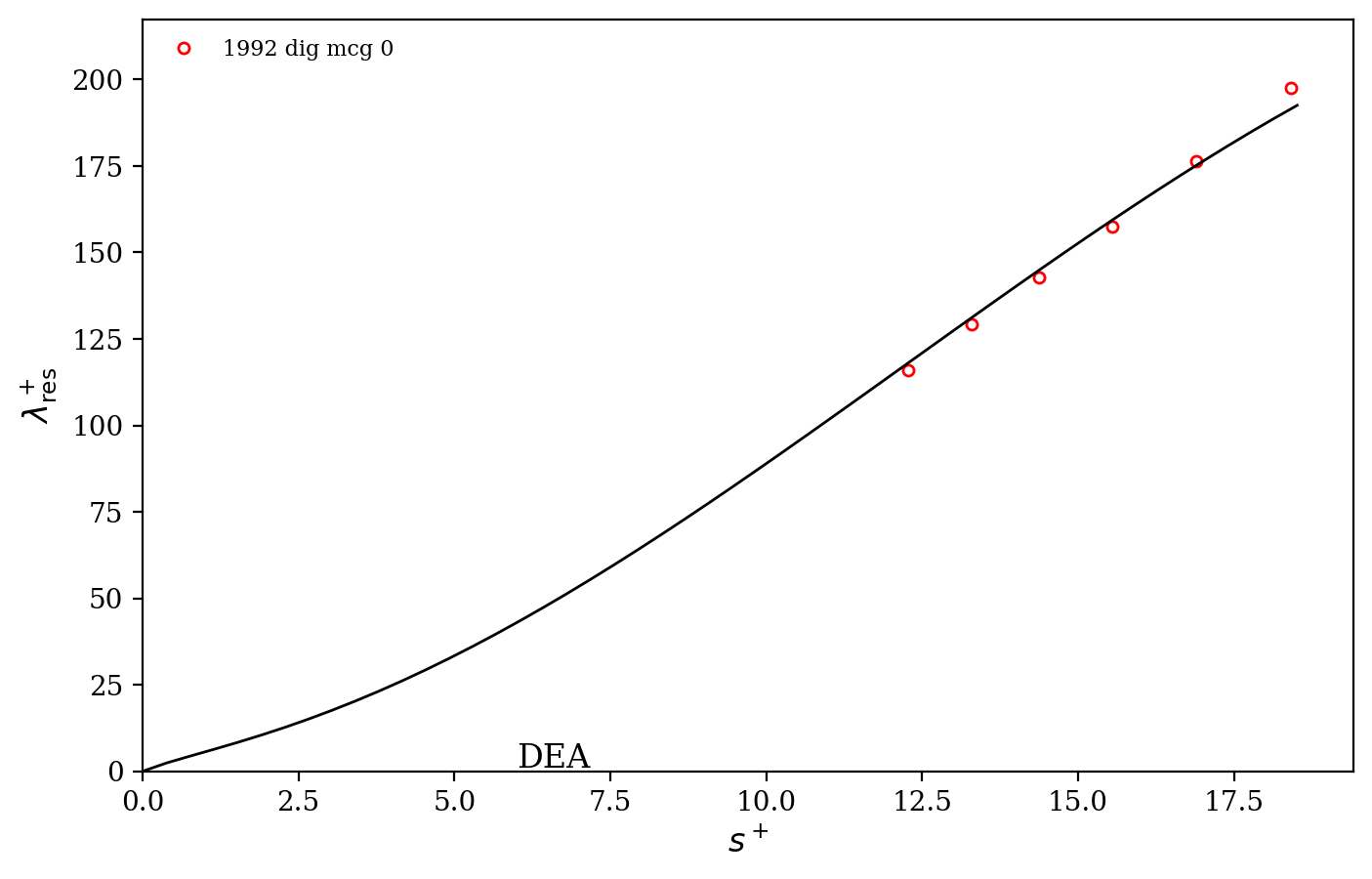

Supplement: Supplementary file 2 — ao4c10815_si_002.zip [file ao4c10815_si_002.zip › Supporting Information/Fig. TC1 - s_plus vs lambda_plus - all data - YFR EoS/DEA.png]

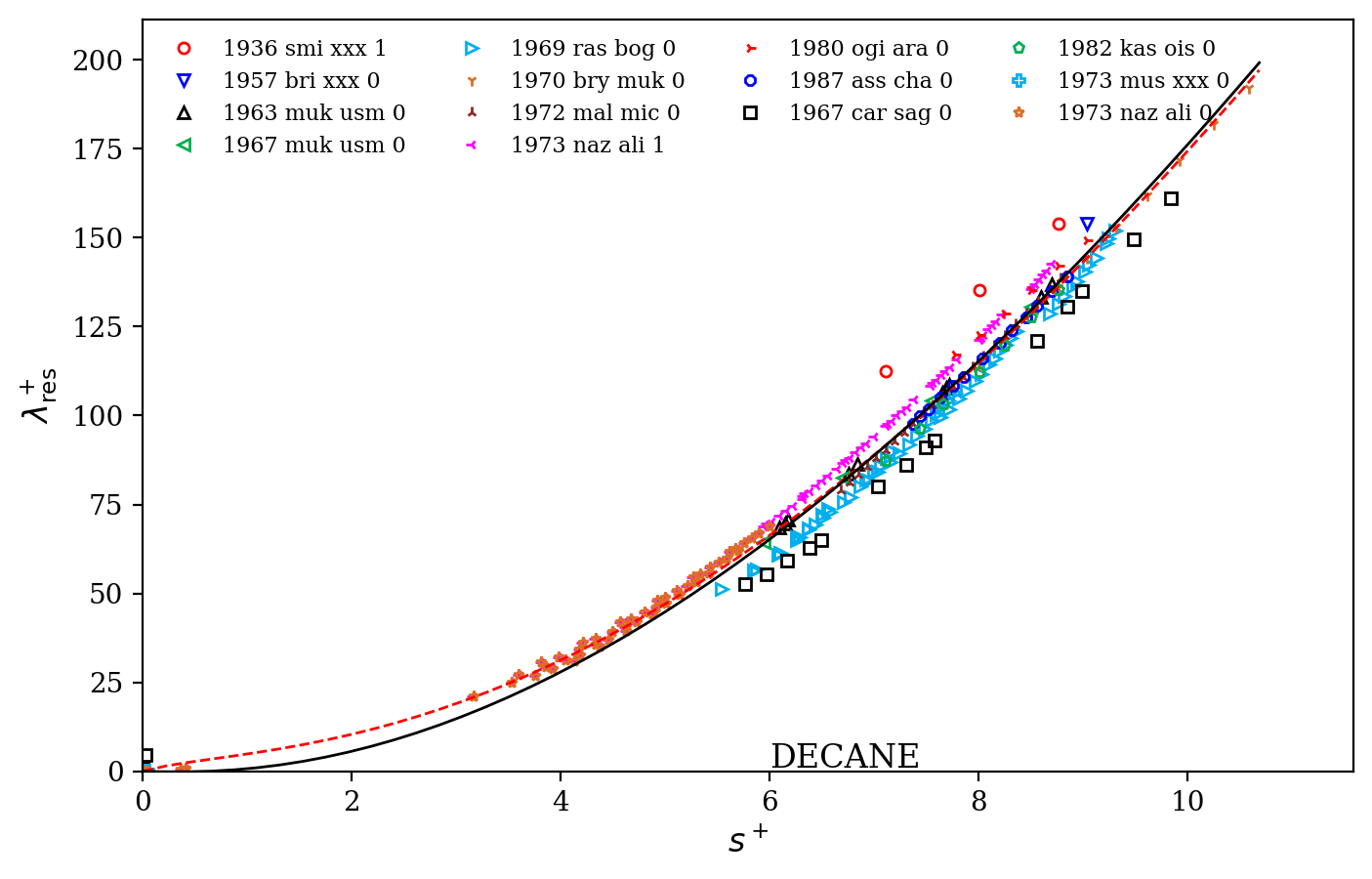

Supplement: Supplementary file 2 — ao4c10815_si_002.zip [file ao4c10815_si_002.zip › Supporting Information/Fig. TC1 - s_plus vs lambda_plus - all data - YFR EoS/DECANE.png]

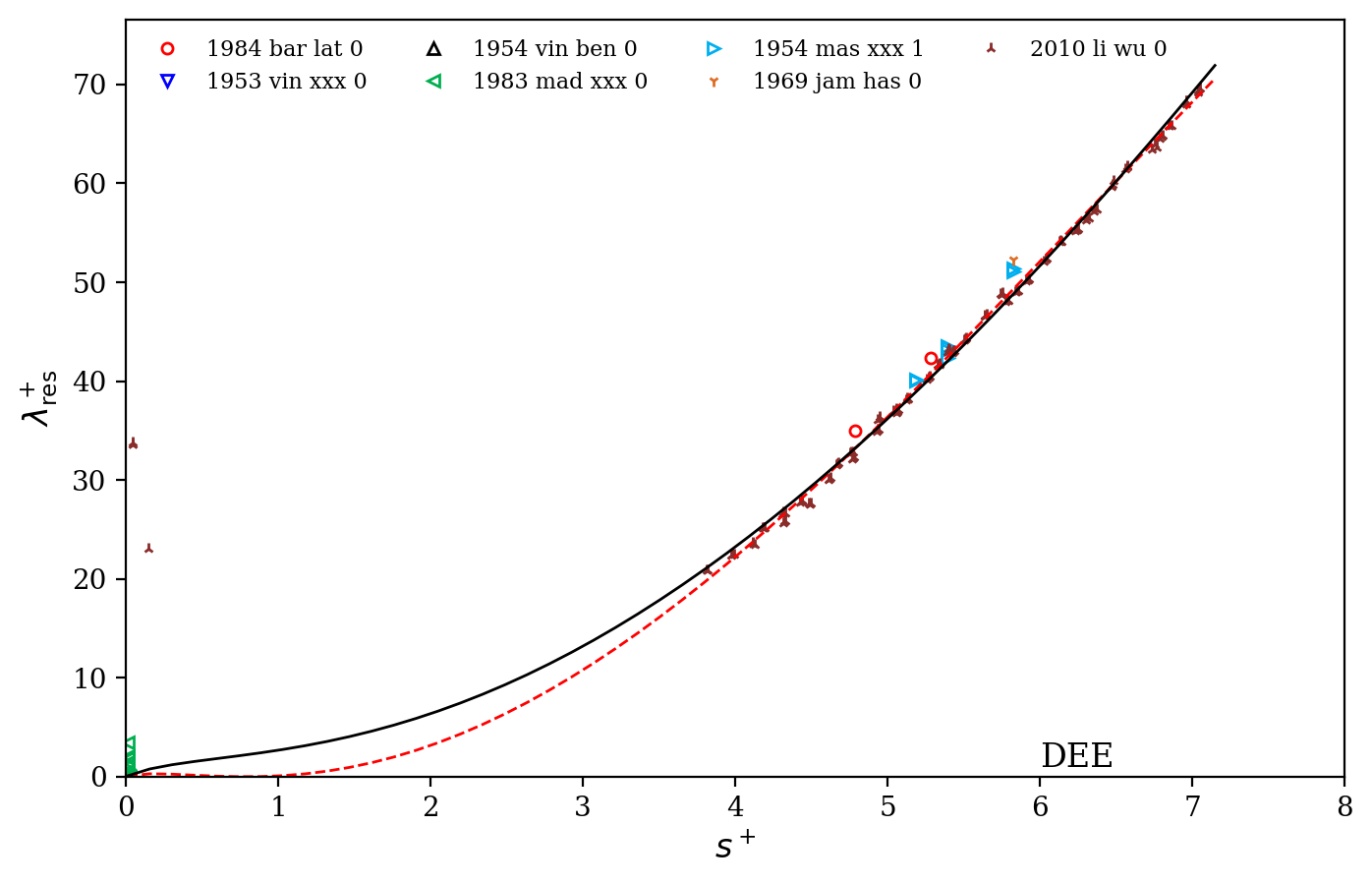

Supplement: Supplementary file 2 — ao4c10815_si_002.zip [file ao4c10815_si_002.zip › Supporting Information/Fig. TC1 - s_plus vs lambda_plus - all data - YFR EoS/DEE.png]

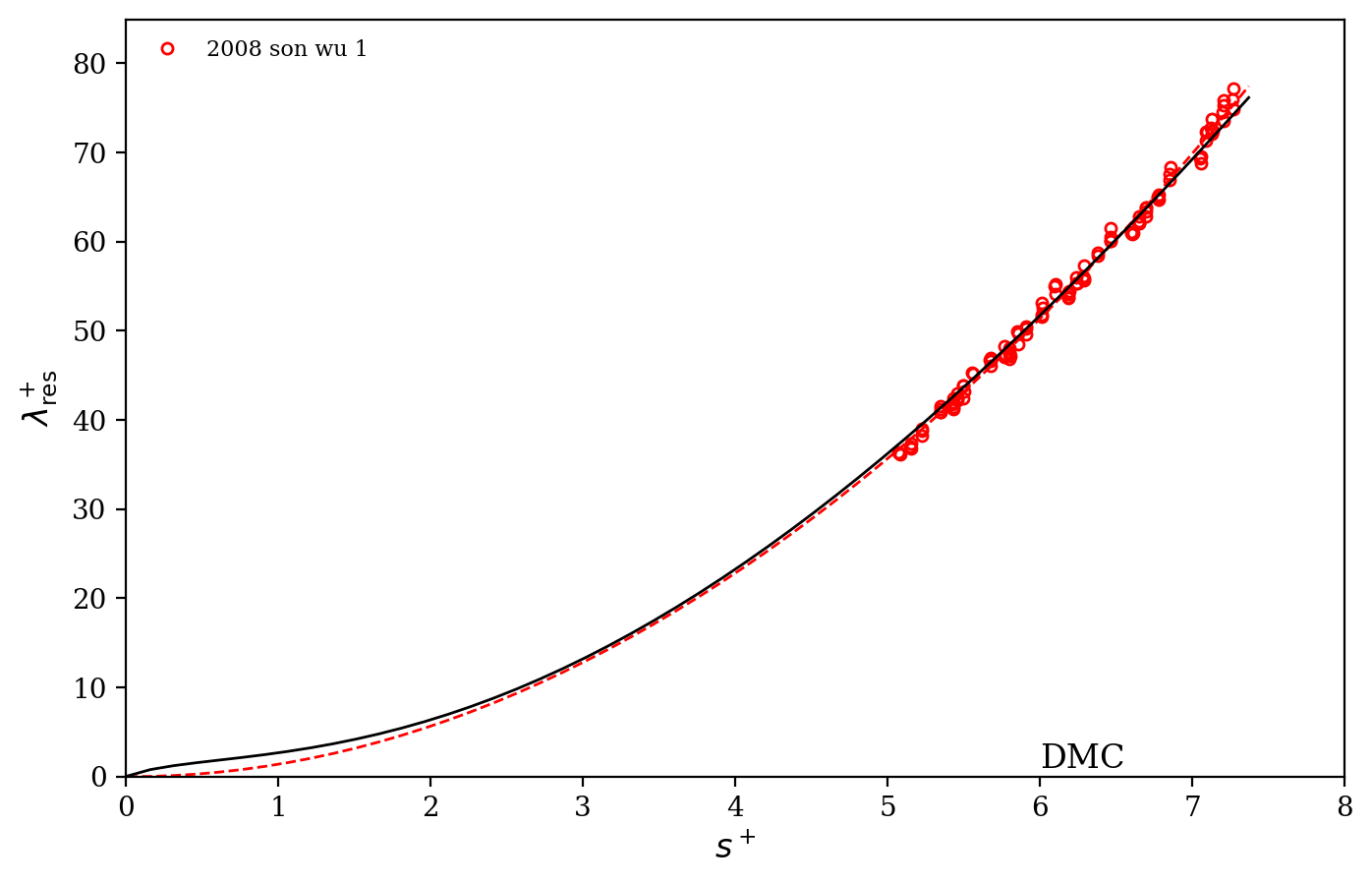

Supplement: Supplementary file 2 — ao4c10815_si_002.zip [file ao4c10815_si_002.zip › Supporting Information/Fig. TC1 - s_plus vs lambda_plus - all data - YFR EoS/DMC.png]

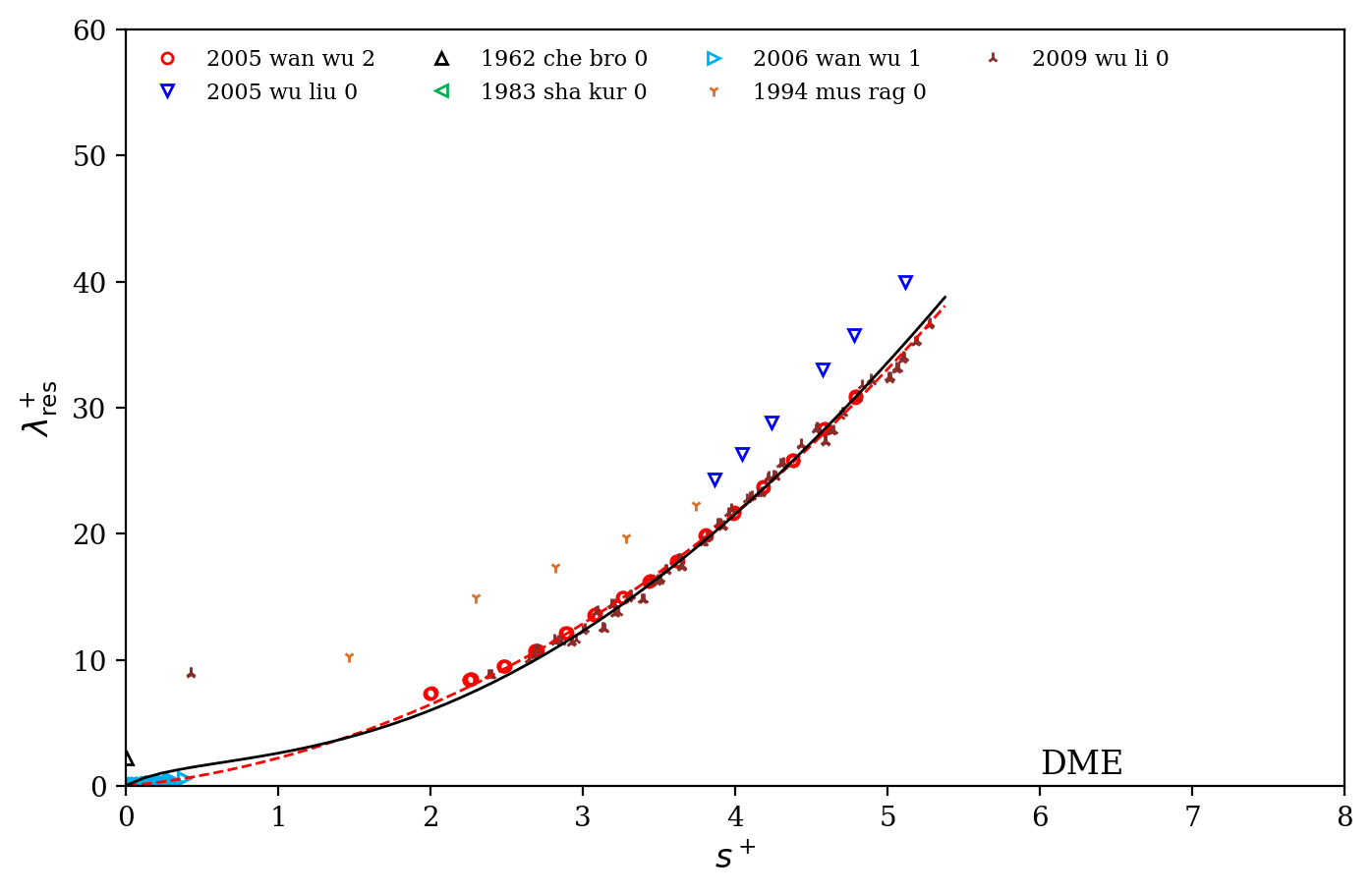

Supplement: Supplementary file 2 — ao4c10815_si_002.zip [file ao4c10815_si_002.zip › Supporting Information/Fig. TC1 - s_plus vs lambda_plus - all data - YFR EoS/DME.png]

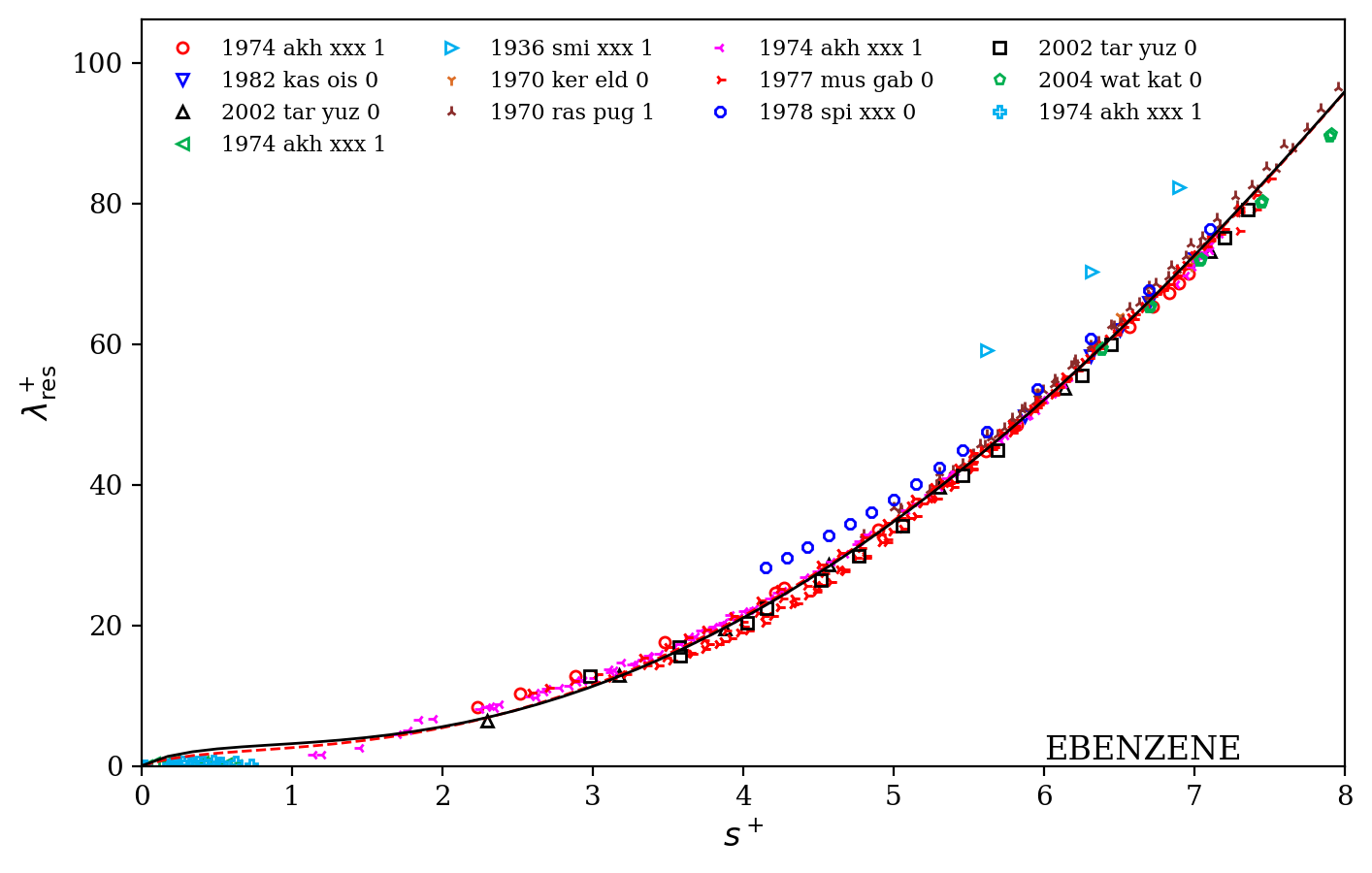

Supplement: Supplementary file 2 — ao4c10815_si_002.zip [file ao4c10815_si_002.zip › Supporting Information/Fig. TC1 - s_plus vs lambda_plus - all data - YFR EoS/EBENZENE.png]

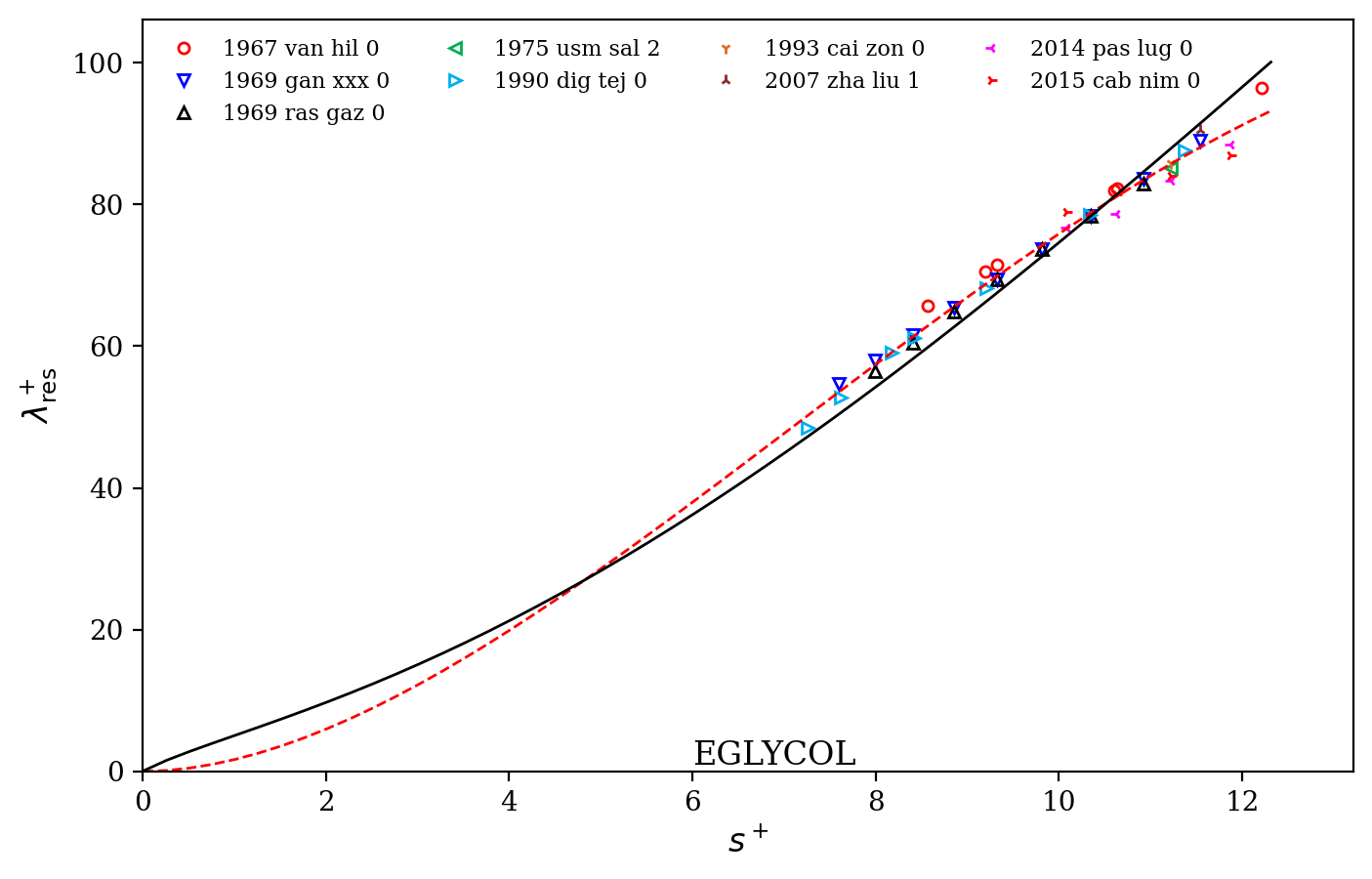

Supplement: Supplementary file 2 — ao4c10815_si_002.zip [file ao4c10815_si_002.zip › Supporting Information/Fig. TC1 - s_plus vs lambda_plus - all data - YFR EoS/EGLYCOL.png]

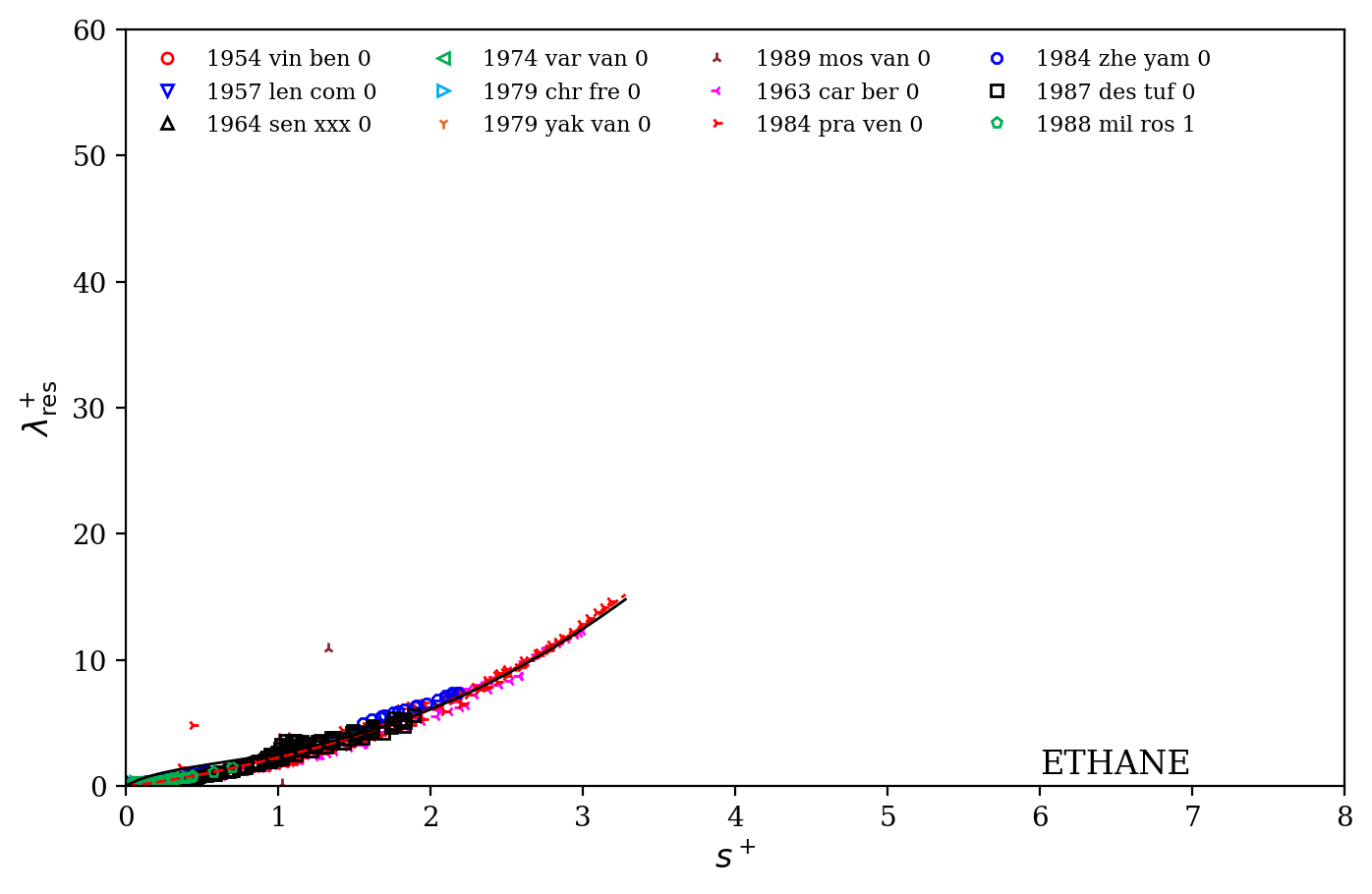

Supplement: Supplementary file 2 — ao4c10815_si_002.zip [file ao4c10815_si_002.zip › Supporting Information/Fig. TC1 - s_plus vs lambda_plus - all data - YFR EoS/ETHANE.png]

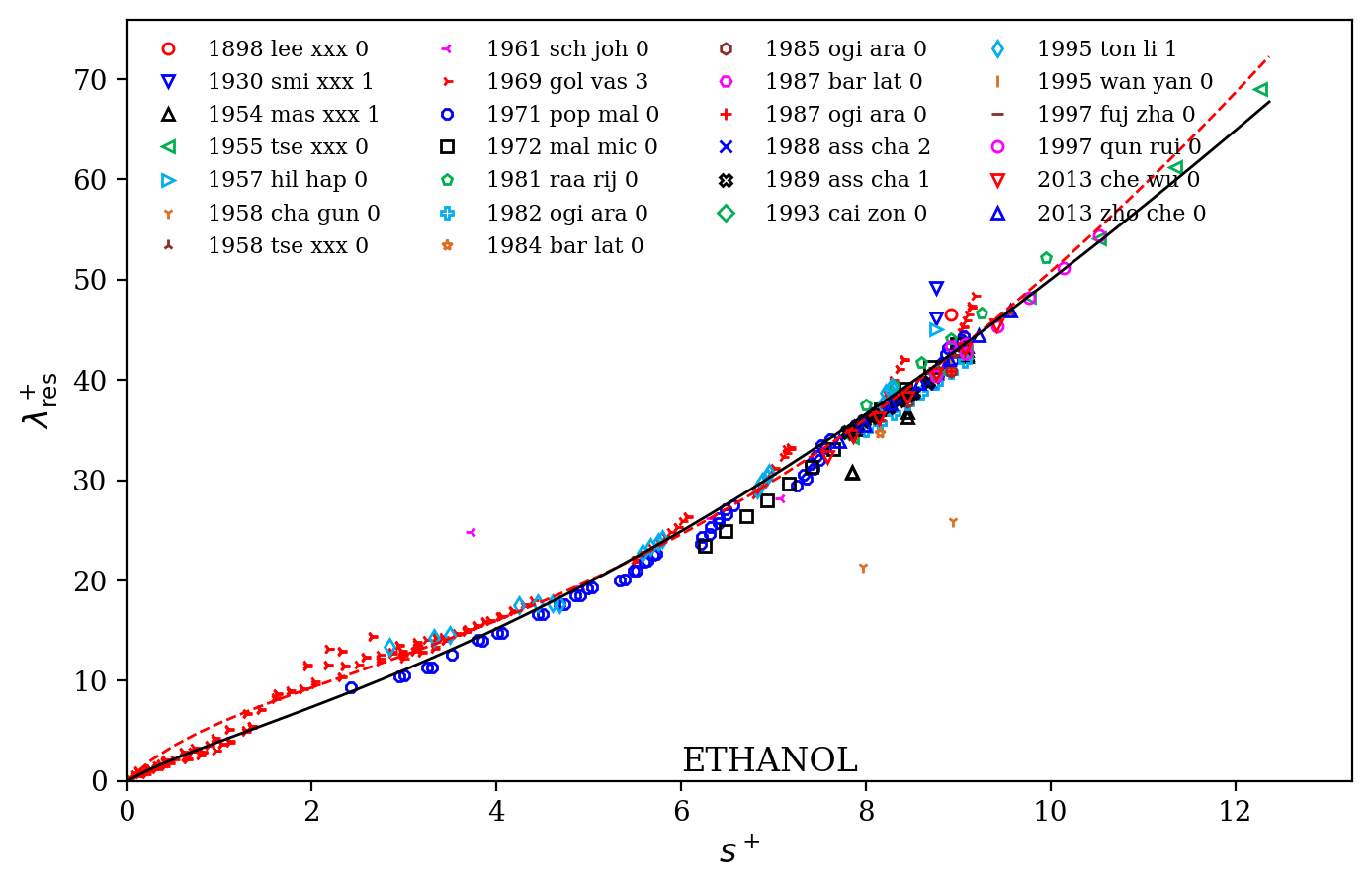

Supplement: Supplementary file 2 — ao4c10815_si_002.zip [file ao4c10815_si_002.zip › Supporting Information/Fig. TC1 - s_plus vs lambda_plus - all data - YFR EoS/ETHANOL.png]

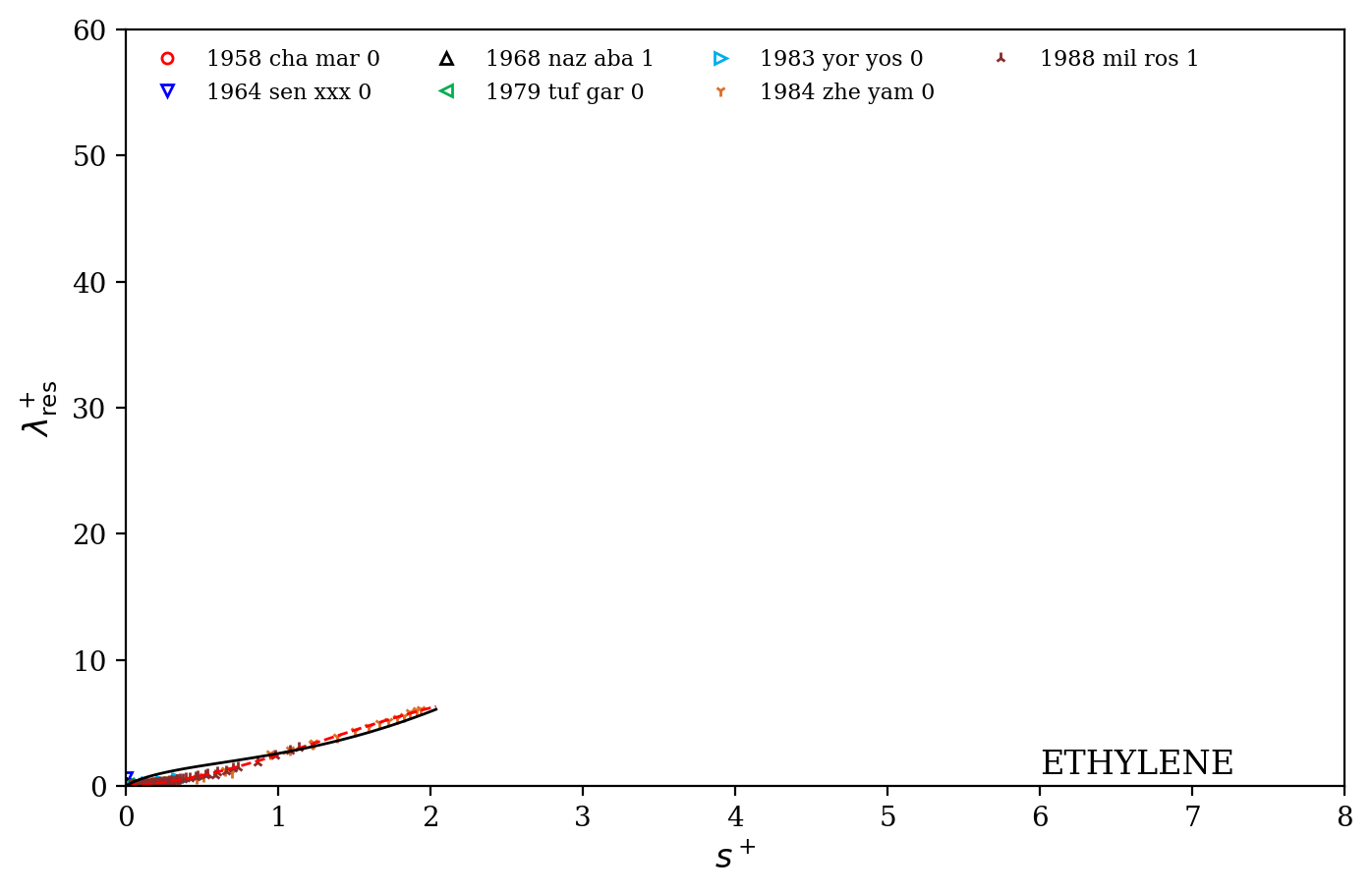

Supplement: Supplementary file 2 — ao4c10815_si_002.zip [file ao4c10815_si_002.zip › Supporting Information/Fig. TC1 - s_plus vs lambda_plus - all data - YFR EoS/ETHYLENE.png]

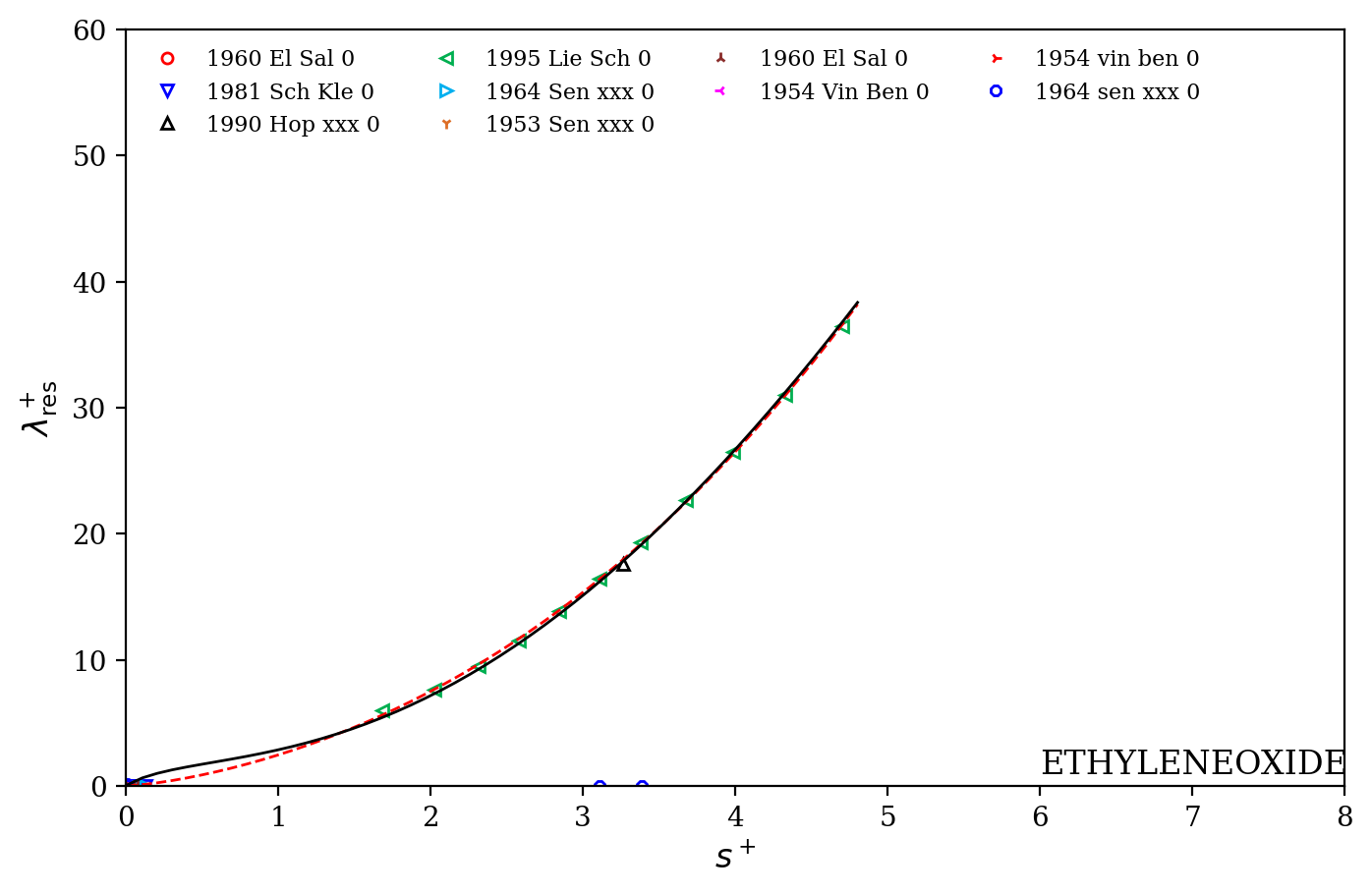

Supplement: Supplementary file 2 — ao4c10815_si_002.zip [file ao4c10815_si_002.zip › Supporting Information/Fig. TC1 - s_plus vs lambda_plus - all data - YFR EoS/ETHYLENEOXIDE.png]

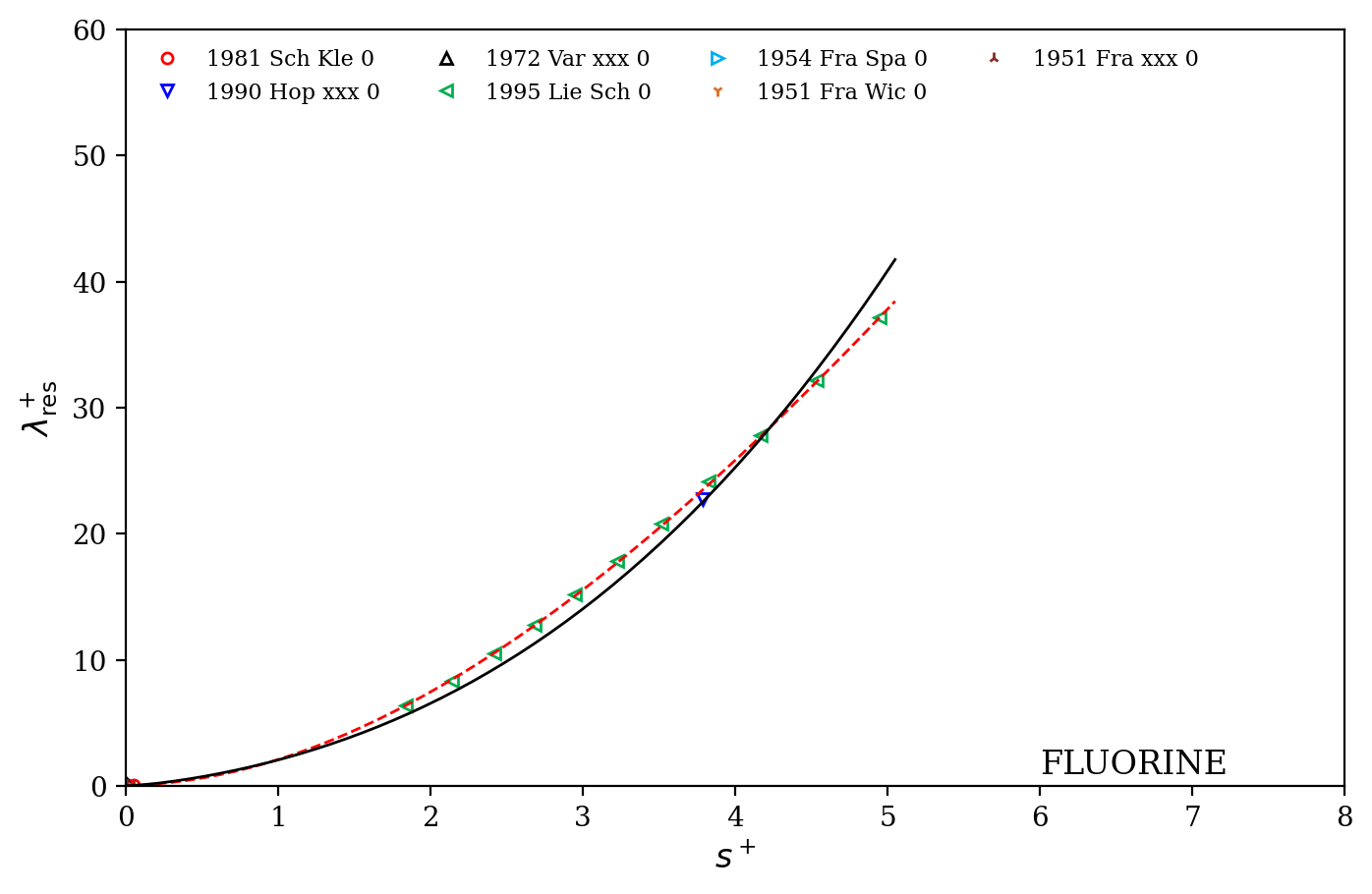

Supplement: Supplementary file 2 — ao4c10815_si_002.zip [file ao4c10815_si_002.zip › Supporting Information/Fig. TC1 - s_plus vs lambda_plus - all data - YFR EoS/FLUORINE.png]

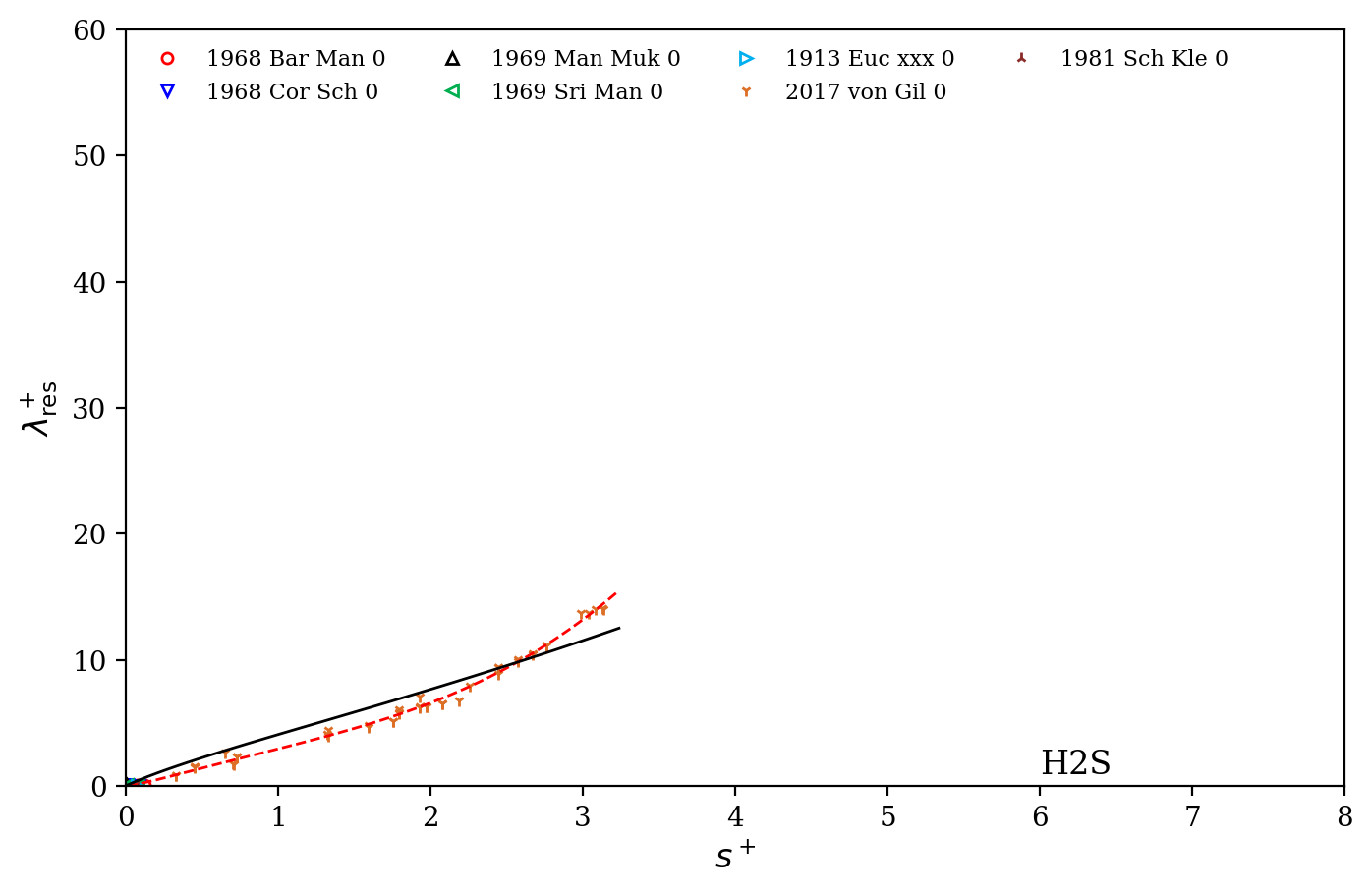

Supplement: Supplementary file 2 — ao4c10815_si_002.zip [file ao4c10815_si_002.zip › Supporting Information/Fig. TC1 - s_plus vs lambda_plus - all data - YFR EoS/H2S.png]

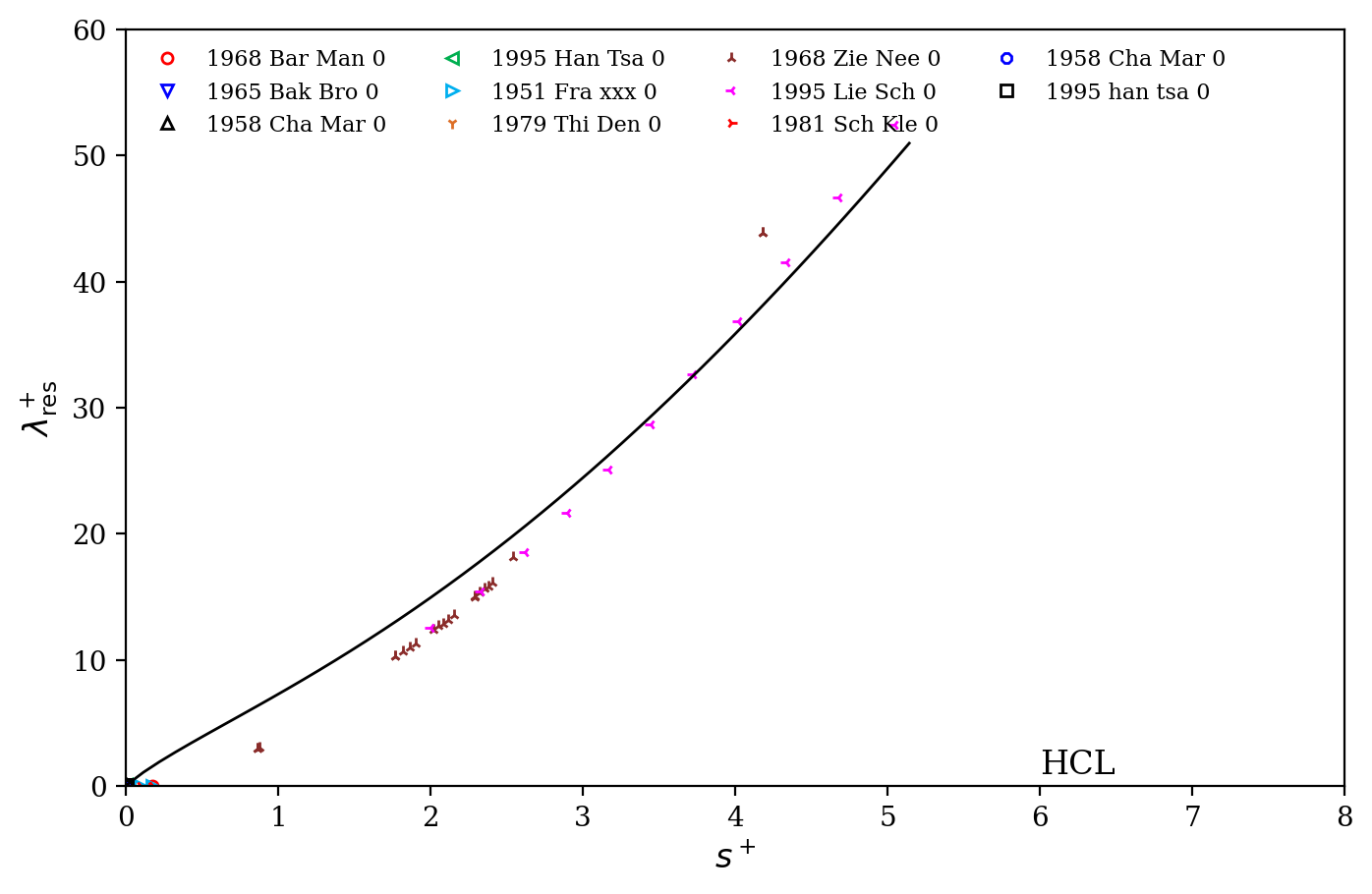

Supplement: Supplementary file 2 — ao4c10815_si_002.zip [file ao4c10815_si_002.zip › Supporting Information/Fig. TC1 - s_plus vs lambda_plus - all data - YFR EoS/HCL.png]

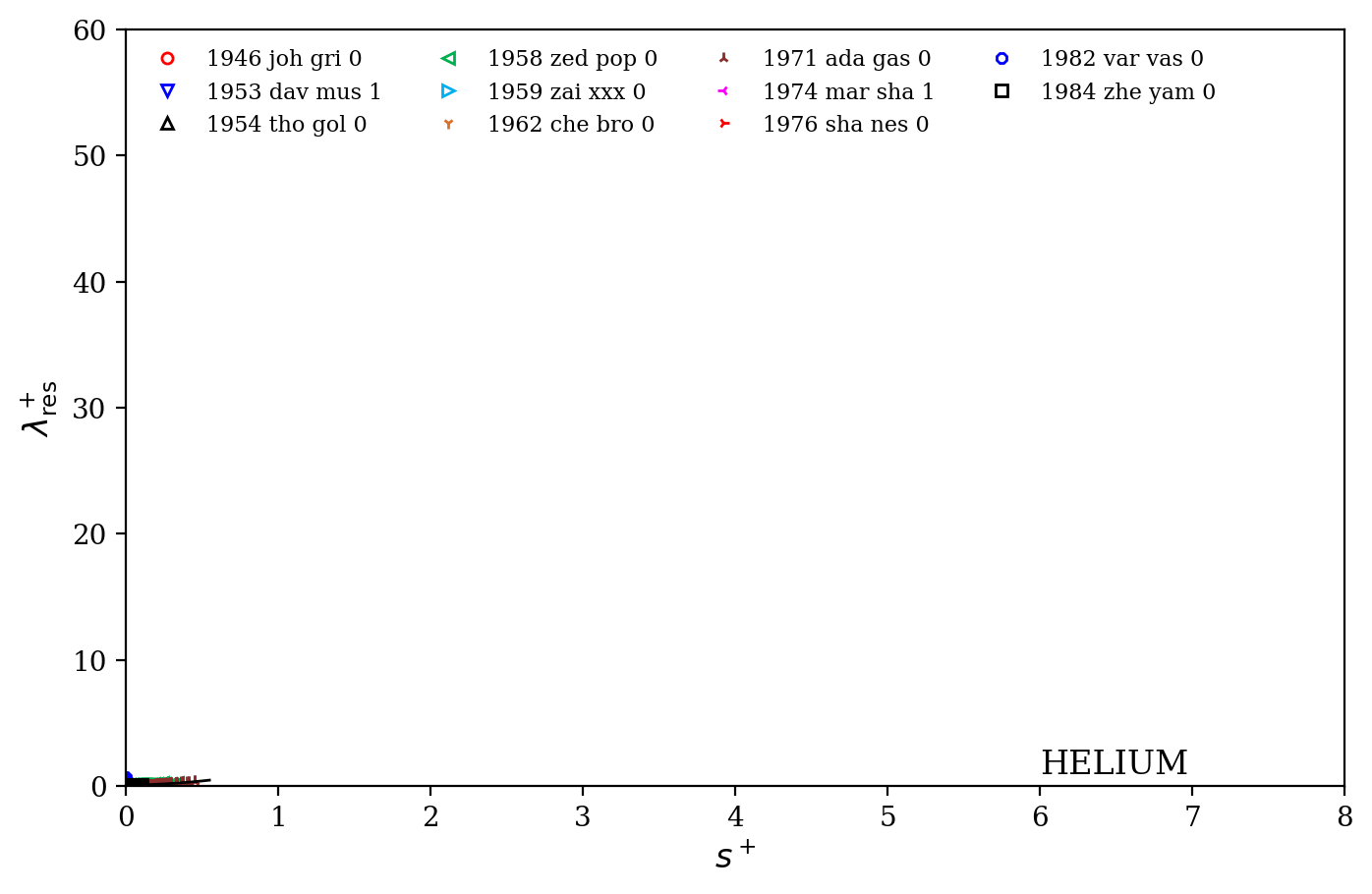

Supplement: Supplementary file 2 — ao4c10815_si_002.zip [file ao4c10815_si_002.zip › Supporting Information/Fig. TC1 - s_plus vs lambda_plus - all data - YFR EoS/HELIUM.png]

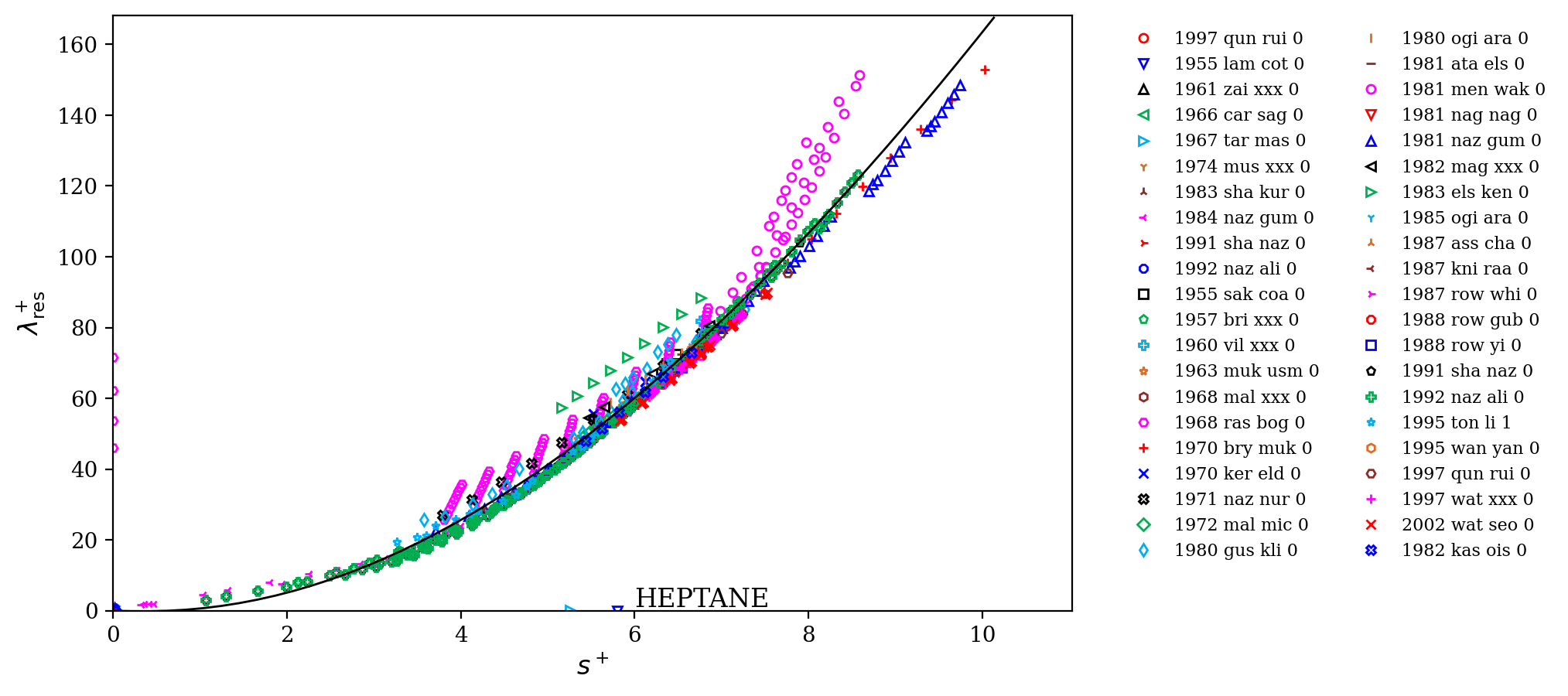

Supplement: Supplementary file 2 — ao4c10815_si_002.zip [file ao4c10815_si_002.zip › Supporting Information/Fig. TC1 - s_plus vs lambda_plus - all data - YFR EoS/HEPTANE.png]

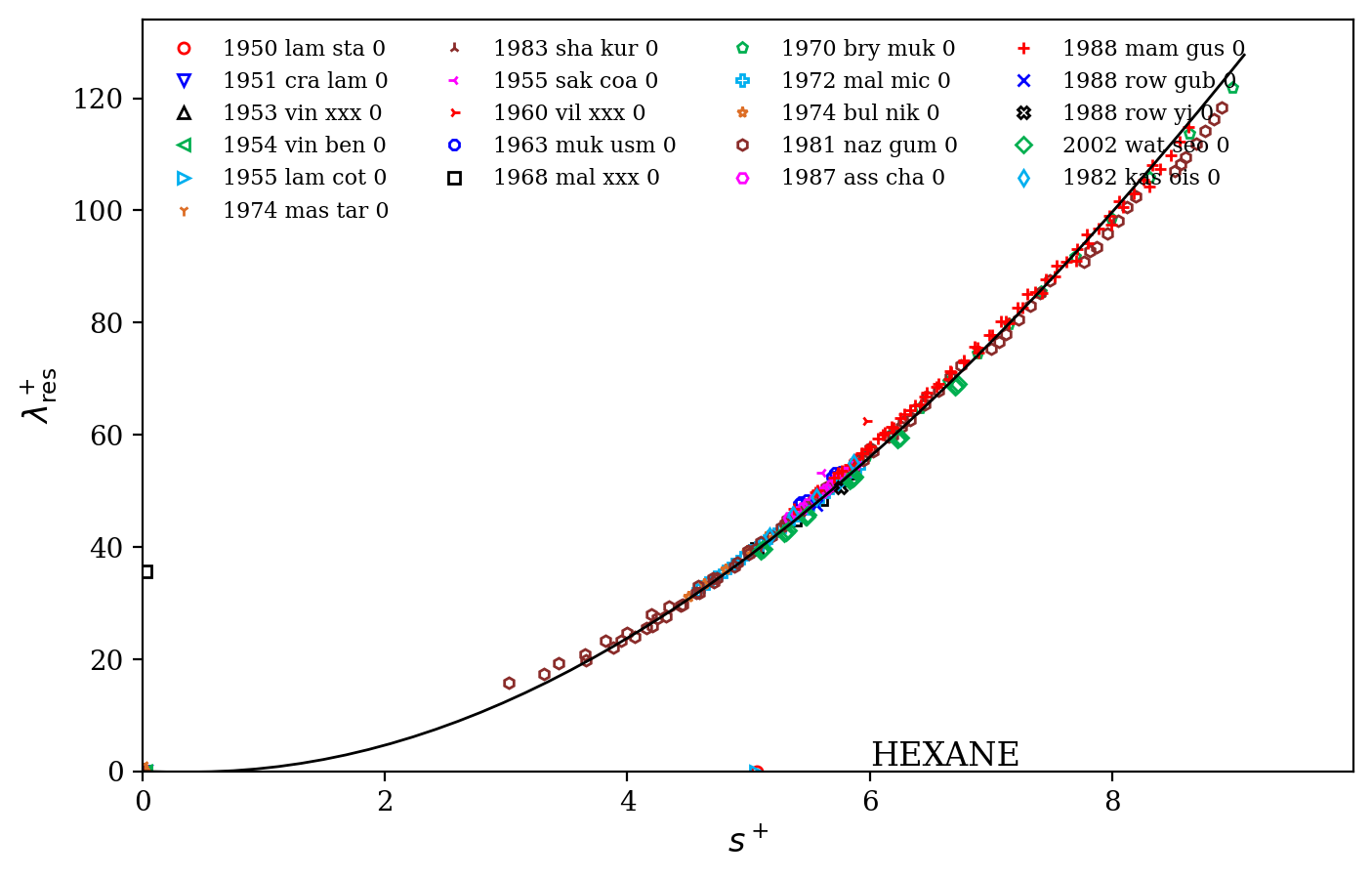

Supplement: Supplementary file 2 — ao4c10815_si_002.zip [file ao4c10815_si_002.zip › Supporting Information/Fig. TC1 - s_plus vs lambda_plus - all data - YFR EoS/HEXANE.png]

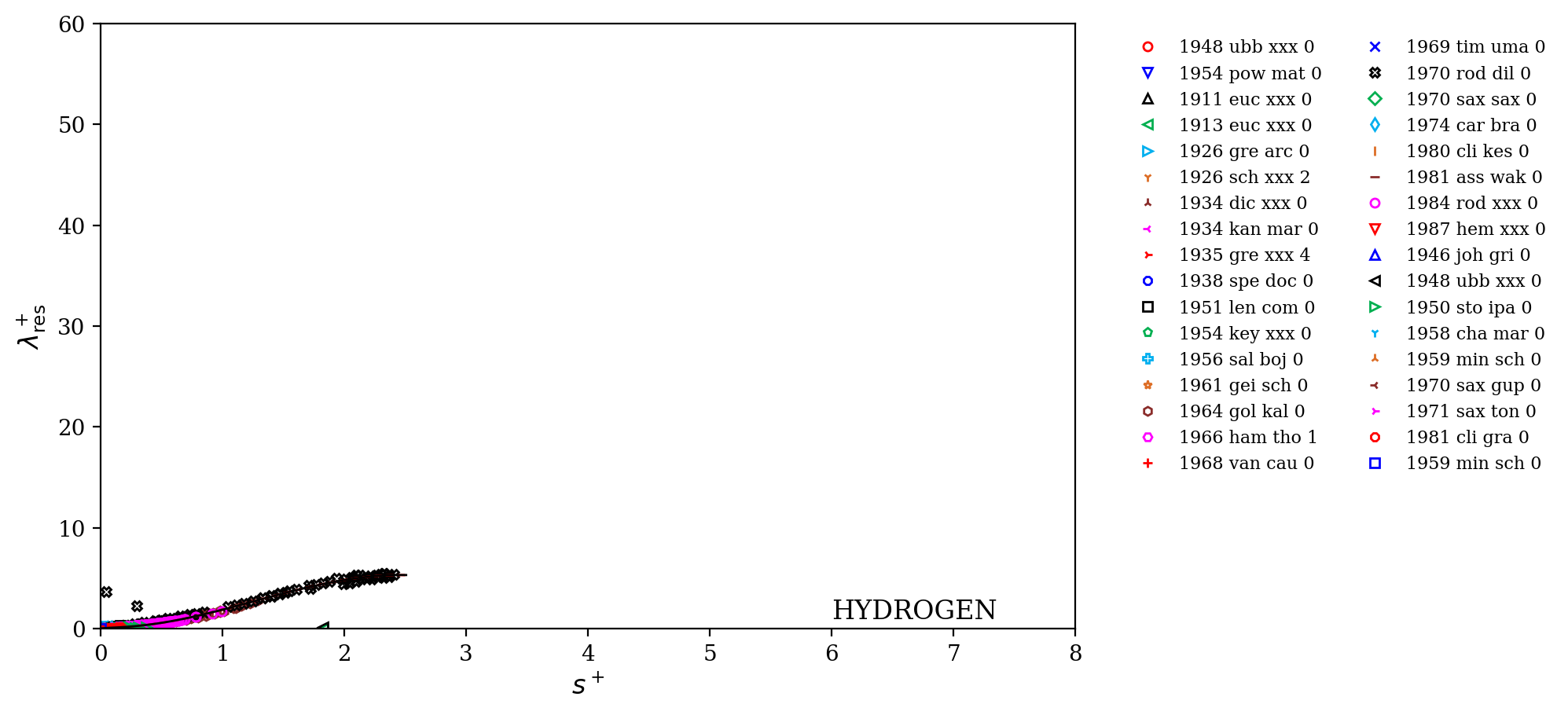

Supplement: Supplementary file 2 — ao4c10815_si_002.zip [file ao4c10815_si_002.zip › Supporting Information/Fig. TC1 - s_plus vs lambda_plus - all data - YFR EoS/HYDROGEN.png]

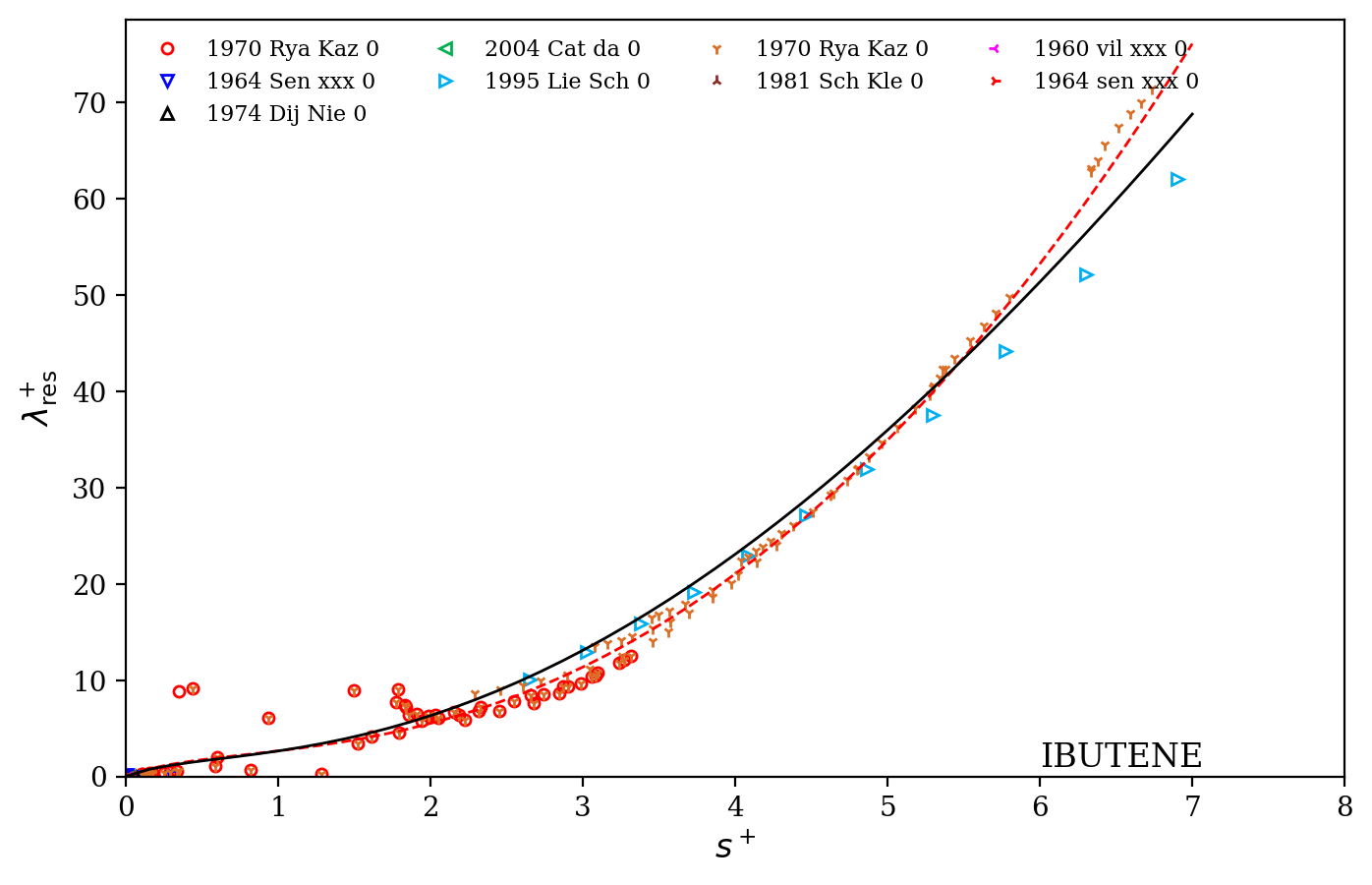

Supplement: Supplementary file 2 — ao4c10815_si_002.zip [file ao4c10815_si_002.zip › Supporting Information/Fig. TC1 - s_plus vs lambda_plus - all data - YFR EoS/IBUTENE.png]

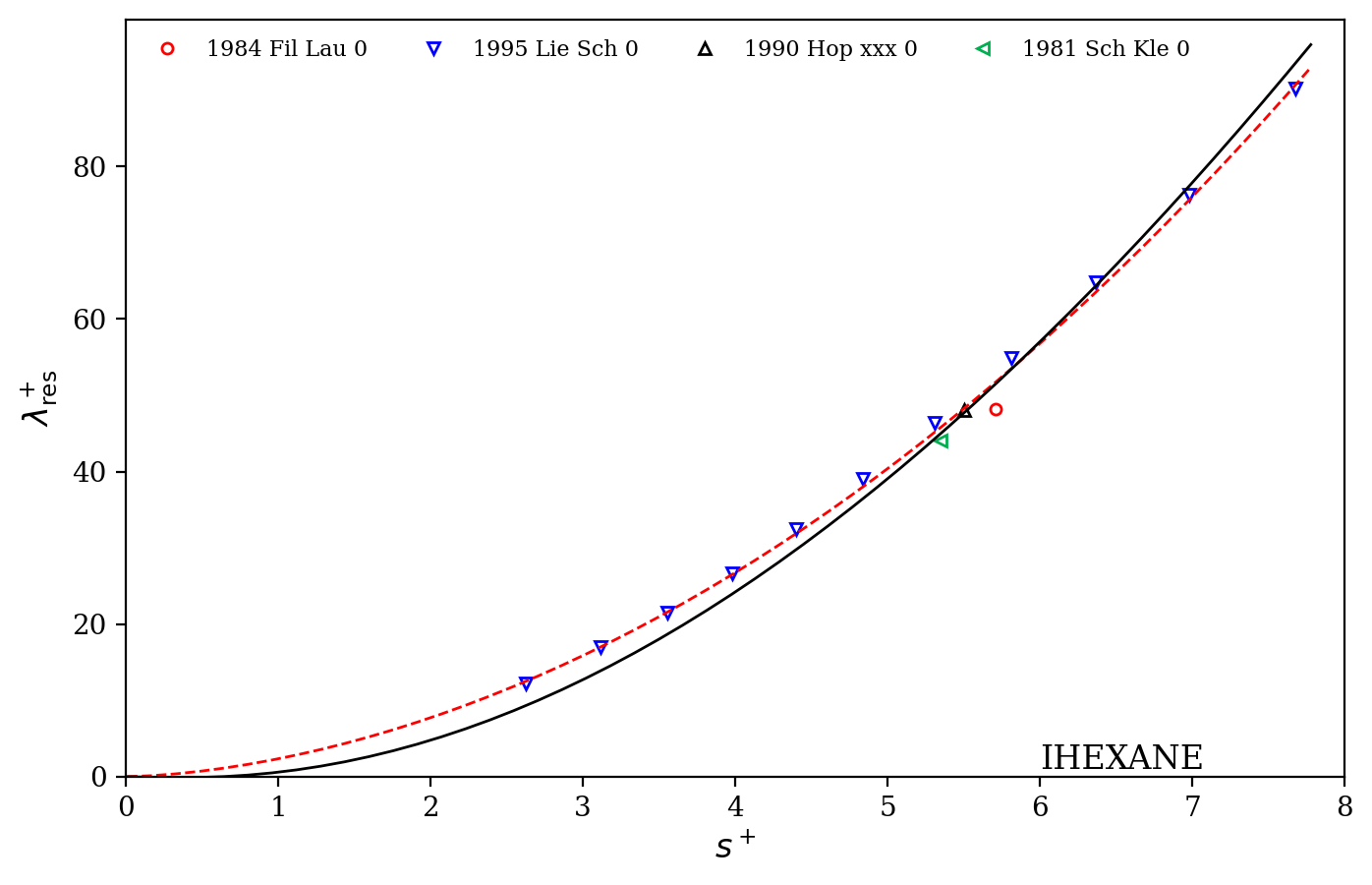

Supplement: Supplementary file 2 — ao4c10815_si_002.zip [file ao4c10815_si_002.zip › Supporting Information/Fig. TC1 - s_plus vs lambda_plus - all data - YFR EoS/IHEXANE.png]

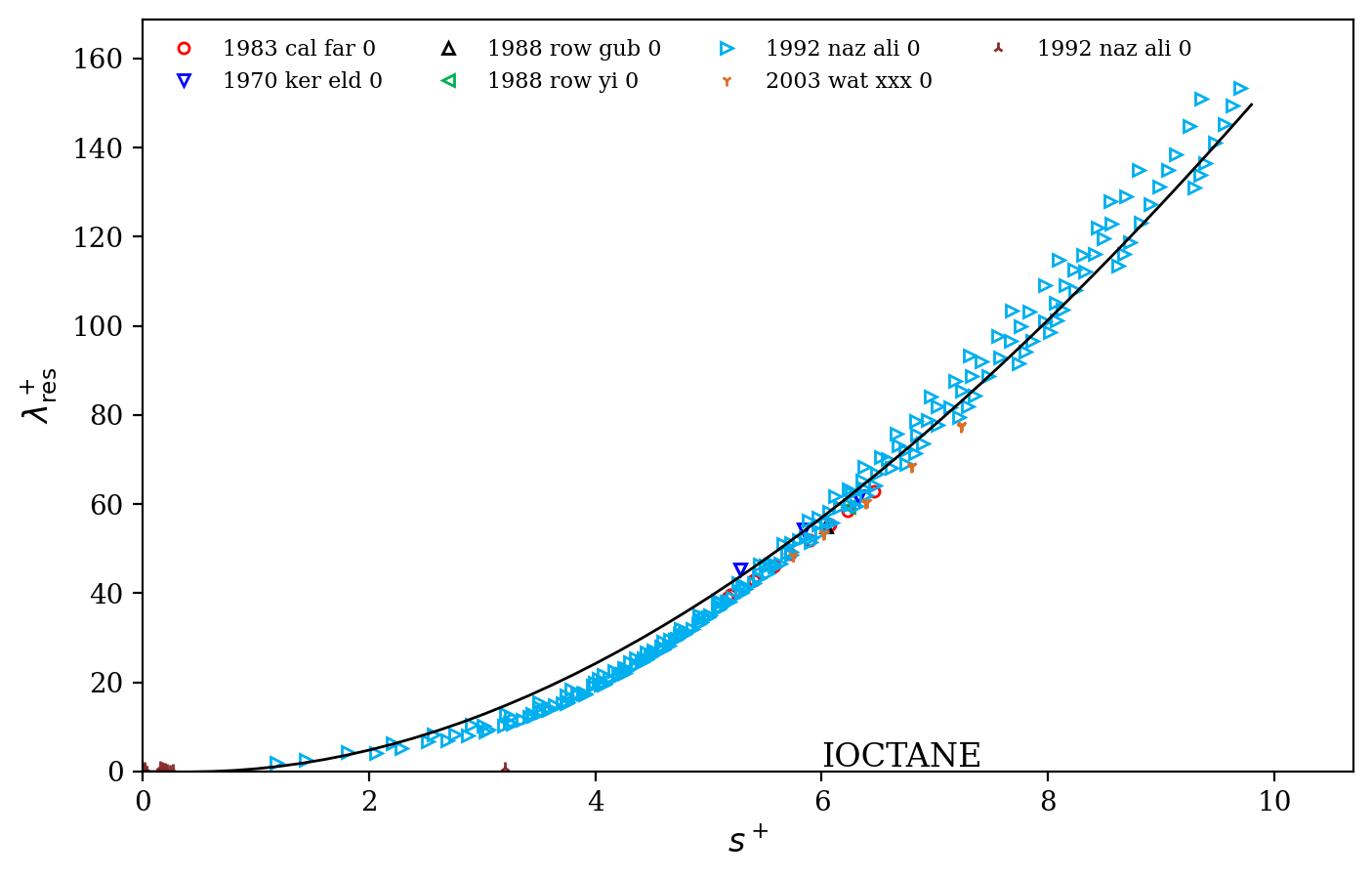

Supplement: Supplementary file 2 — ao4c10815_si_002.zip [file ao4c10815_si_002.zip › Supporting Information/Fig. TC1 - s_plus vs lambda_plus - all data - YFR EoS/IOCTANE.png]

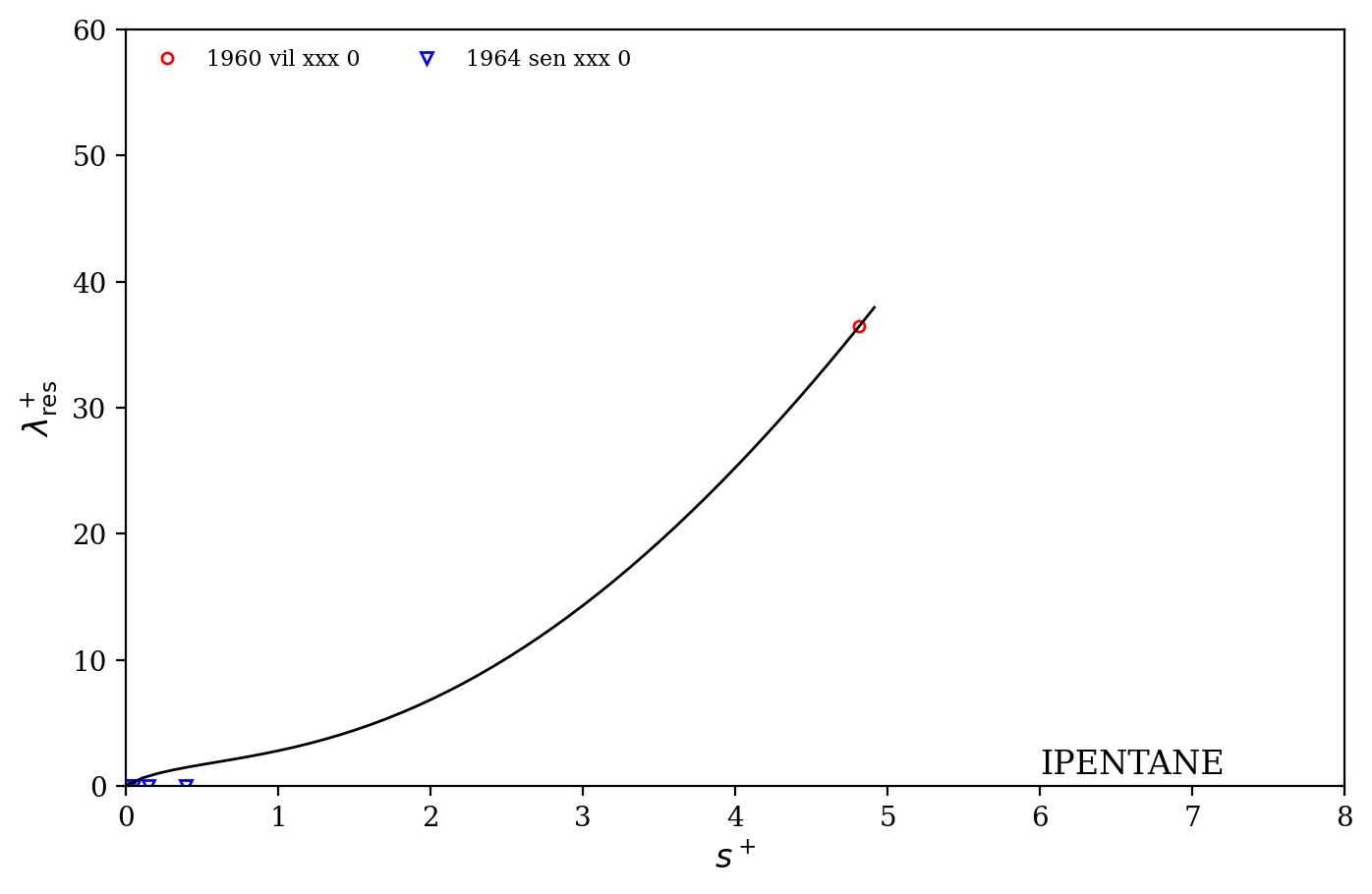

Supplement: Supplementary file 2 — ao4c10815_si_002.zip [file ao4c10815_si_002.zip › Supporting Information/Fig. TC1 - s_plus vs lambda_plus - all data - YFR EoS/IPENTANE.png]

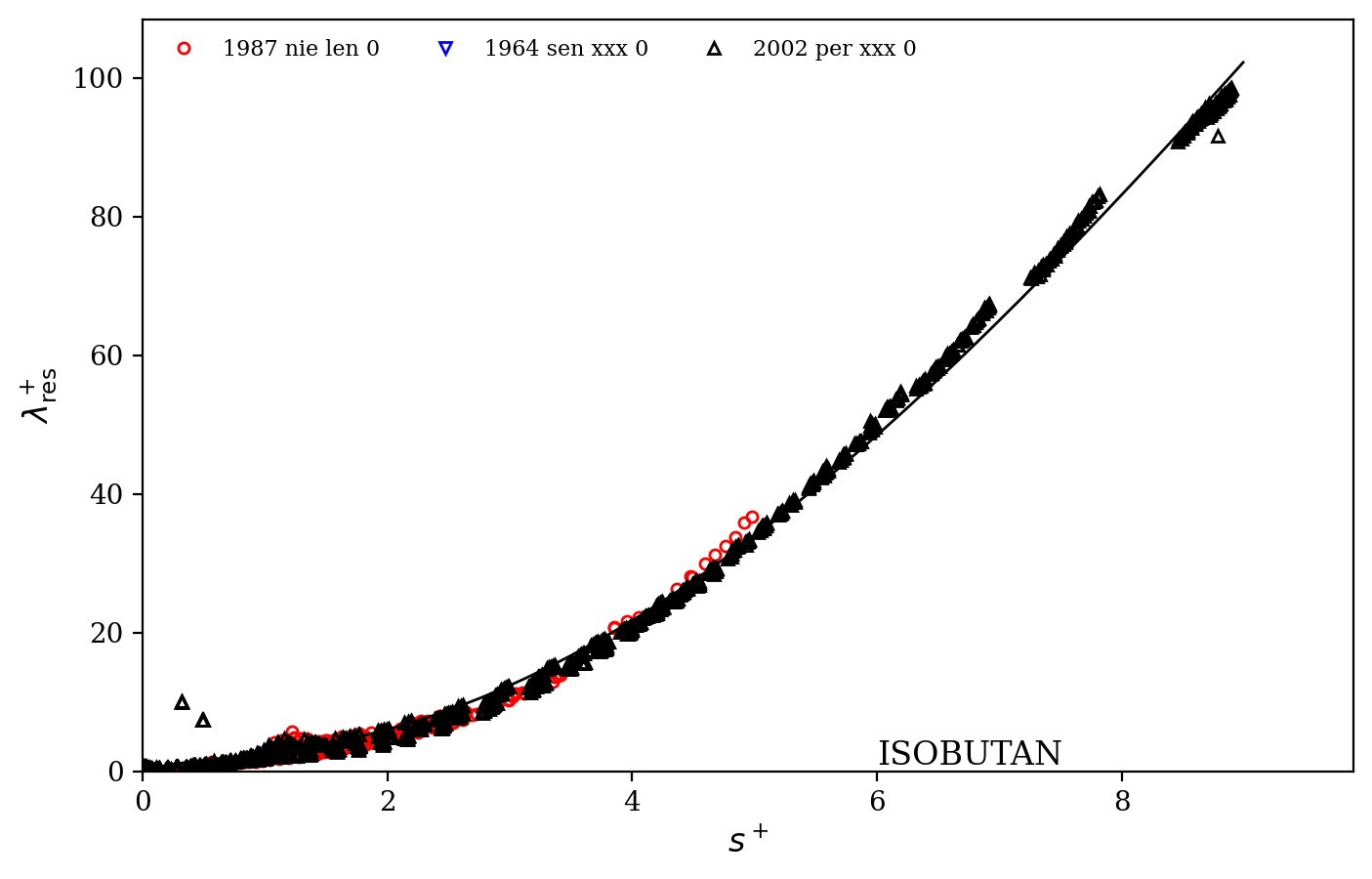

Supplement: Supplementary file 2 — ao4c10815_si_002.zip [file ao4c10815_si_002.zip › Supporting Information/Fig. TC1 - s_plus vs lambda_plus - all data - YFR EoS/ISOBUTAN.png]

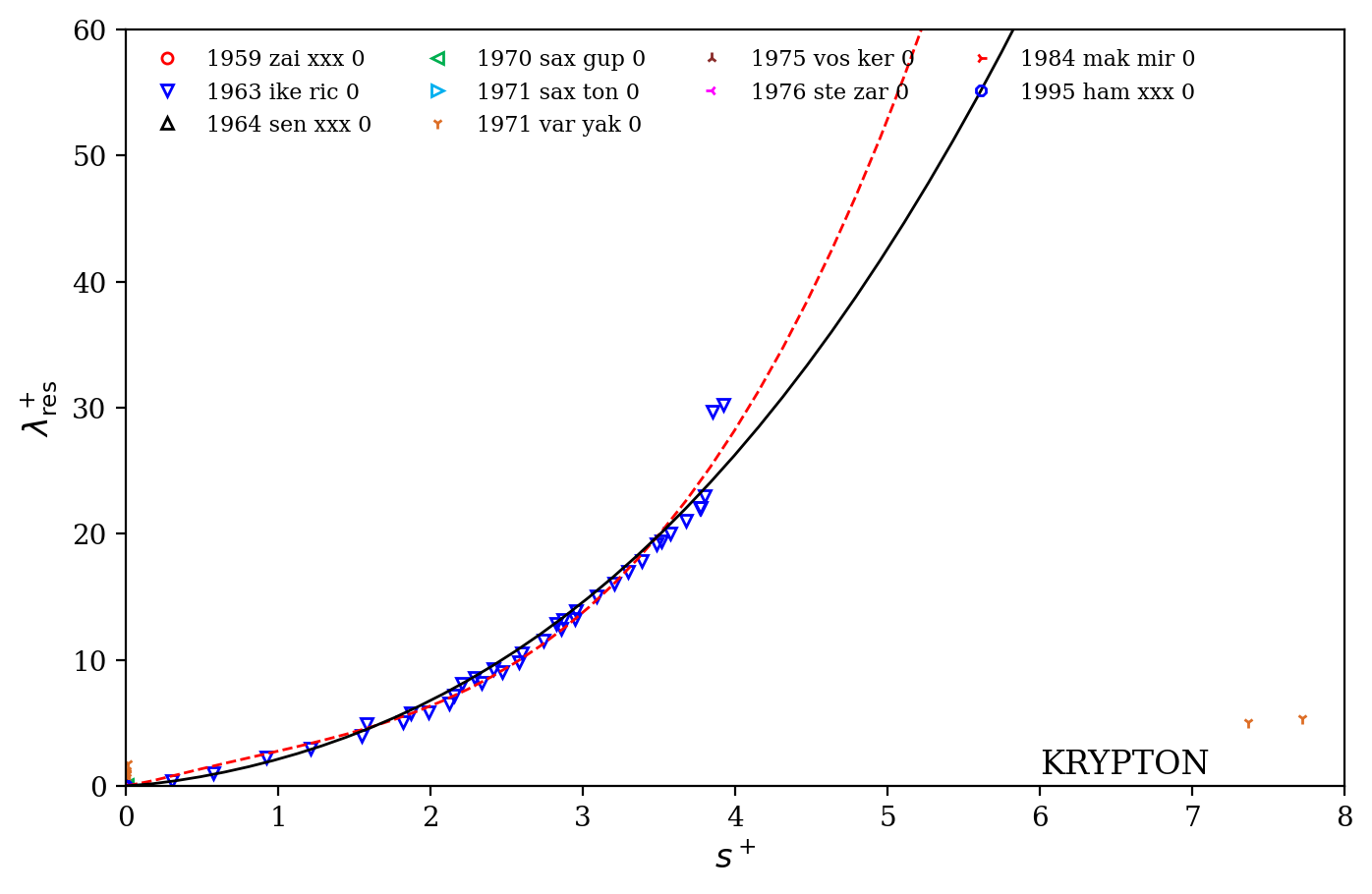

Supplement: Supplementary file 2 — ao4c10815_si_002.zip [file ao4c10815_si_002.zip › Supporting Information/Fig. TC1 - s_plus vs lambda_plus - all data - YFR EoS/KRYPTON.png]

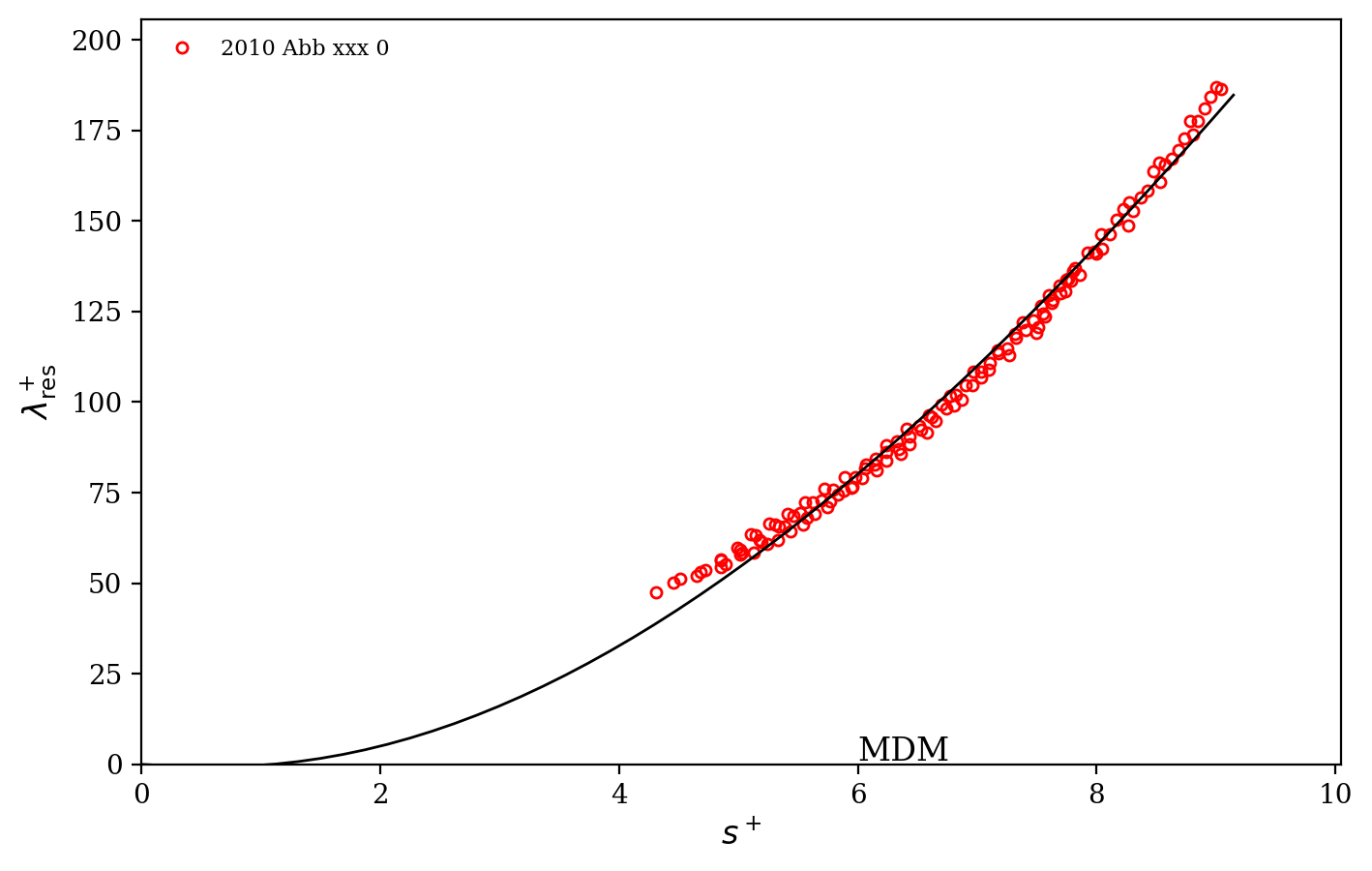

Supplement: Supplementary file 2 — ao4c10815_si_002.zip [file ao4c10815_si_002.zip › Supporting Information/Fig. TC1 - s_plus vs lambda_plus - all data - YFR EoS/MDM.png]

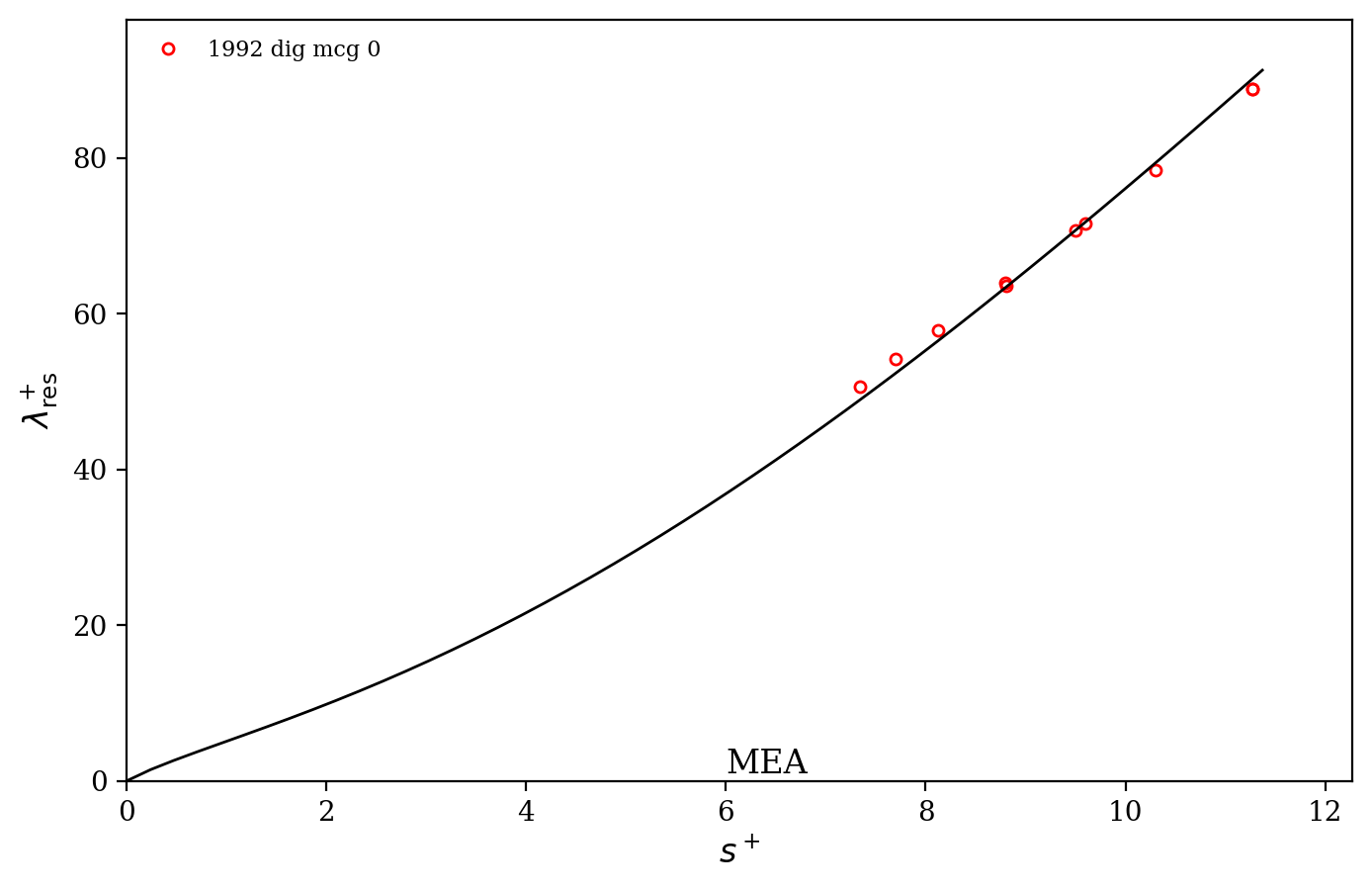

Supplement: Supplementary file 2 — ao4c10815_si_002.zip [file ao4c10815_si_002.zip › Supporting Information/Fig. TC1 - s_plus vs lambda_plus - all data - YFR EoS/MEA.png]

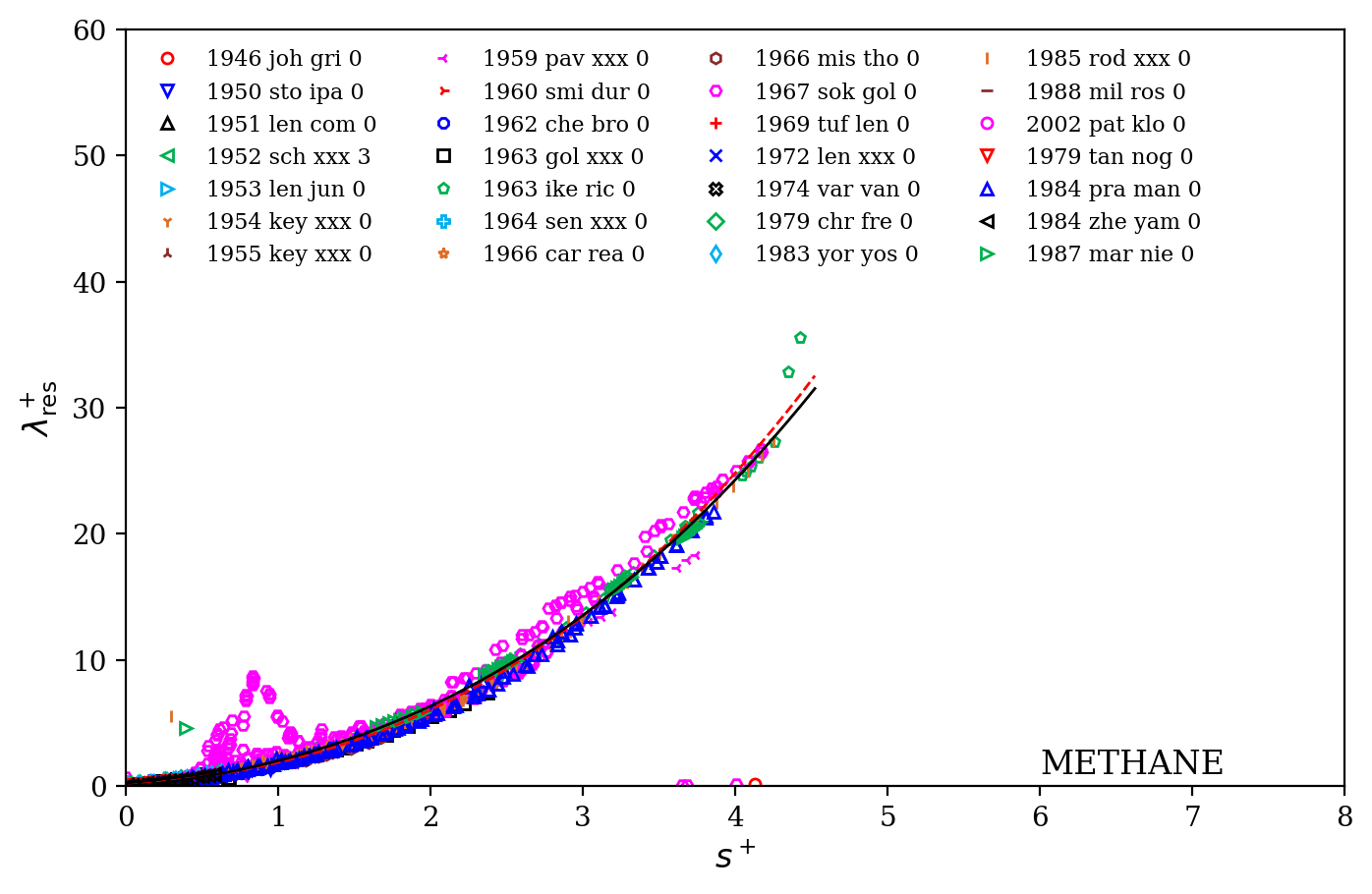

Supplement: Supplementary file 2 — ao4c10815_si_002.zip [file ao4c10815_si_002.zip › Supporting Information/Fig. TC1 - s_plus vs lambda_plus - all data - YFR EoS/METHANE.png]

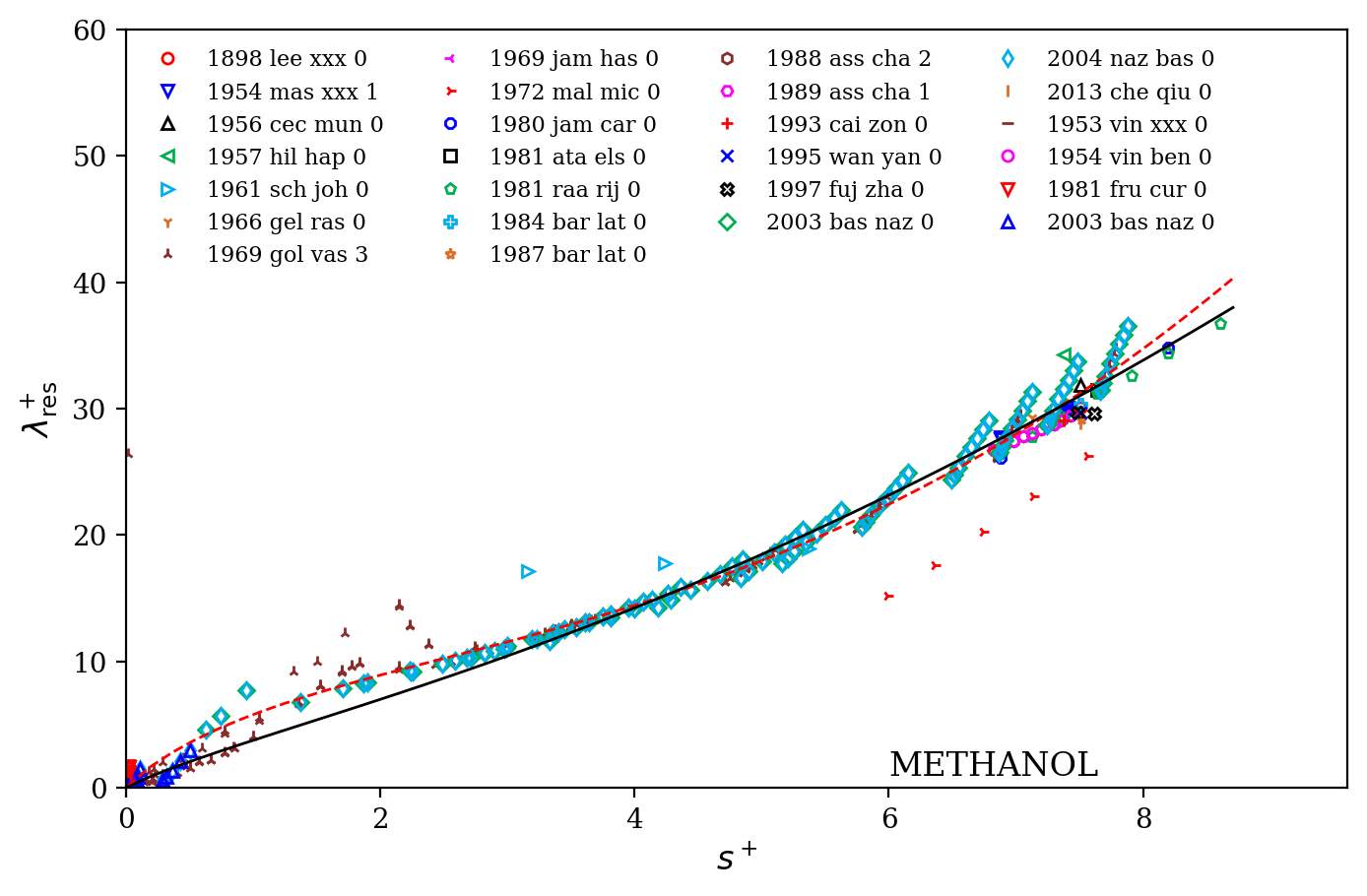

Supplement: Supplementary file 2 — ao4c10815_si_002.zip [file ao4c10815_si_002.zip › Supporting Information/Fig. TC1 - s_plus vs lambda_plus - all data - YFR EoS/METHANOL.png]

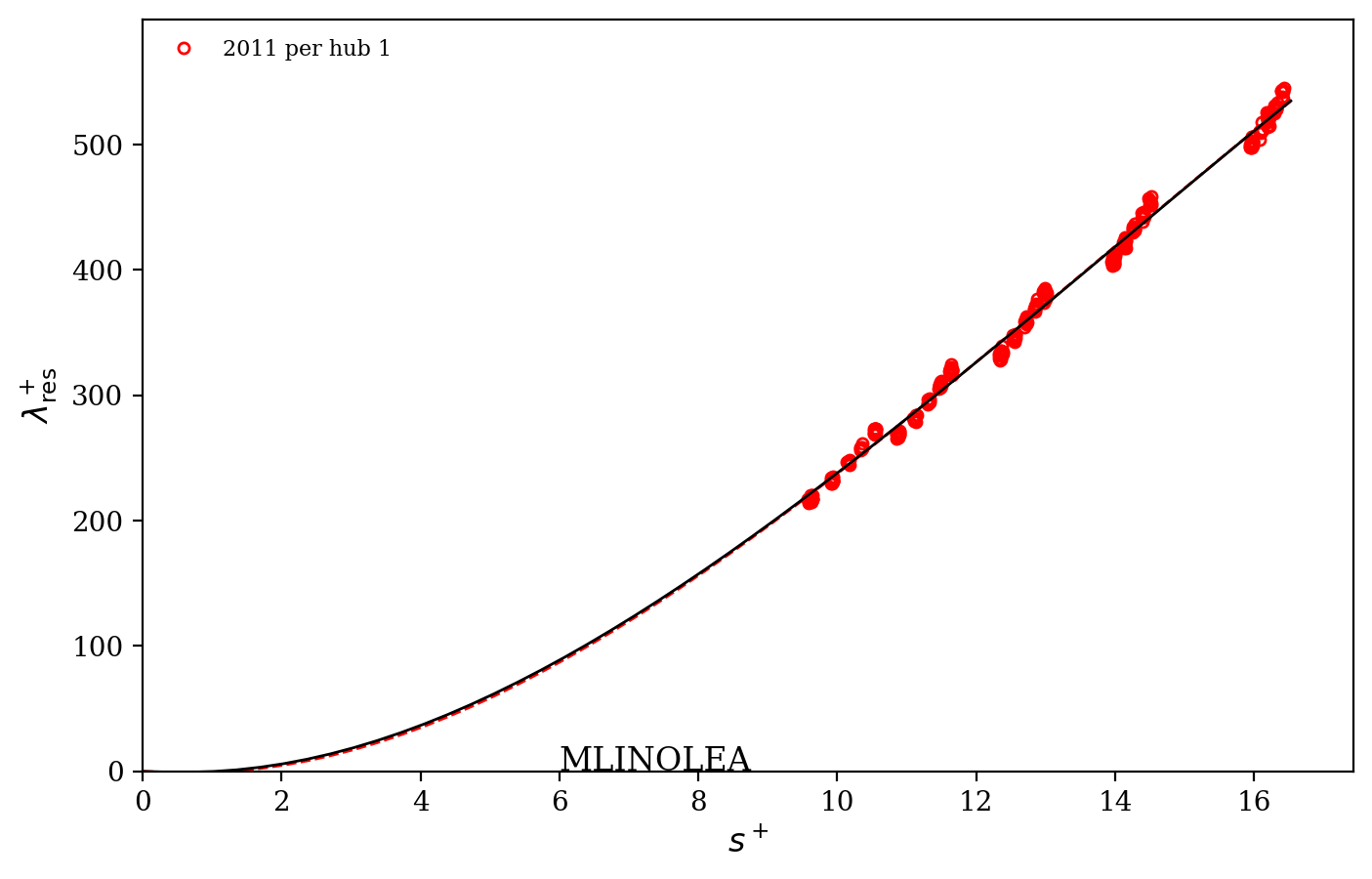

Supplement: Supplementary file 2 — ao4c10815_si_002.zip [file ao4c10815_si_002.zip › Supporting Information/Fig. TC1 - s_plus vs lambda_plus - all data - YFR EoS/MLINOLEA.png]

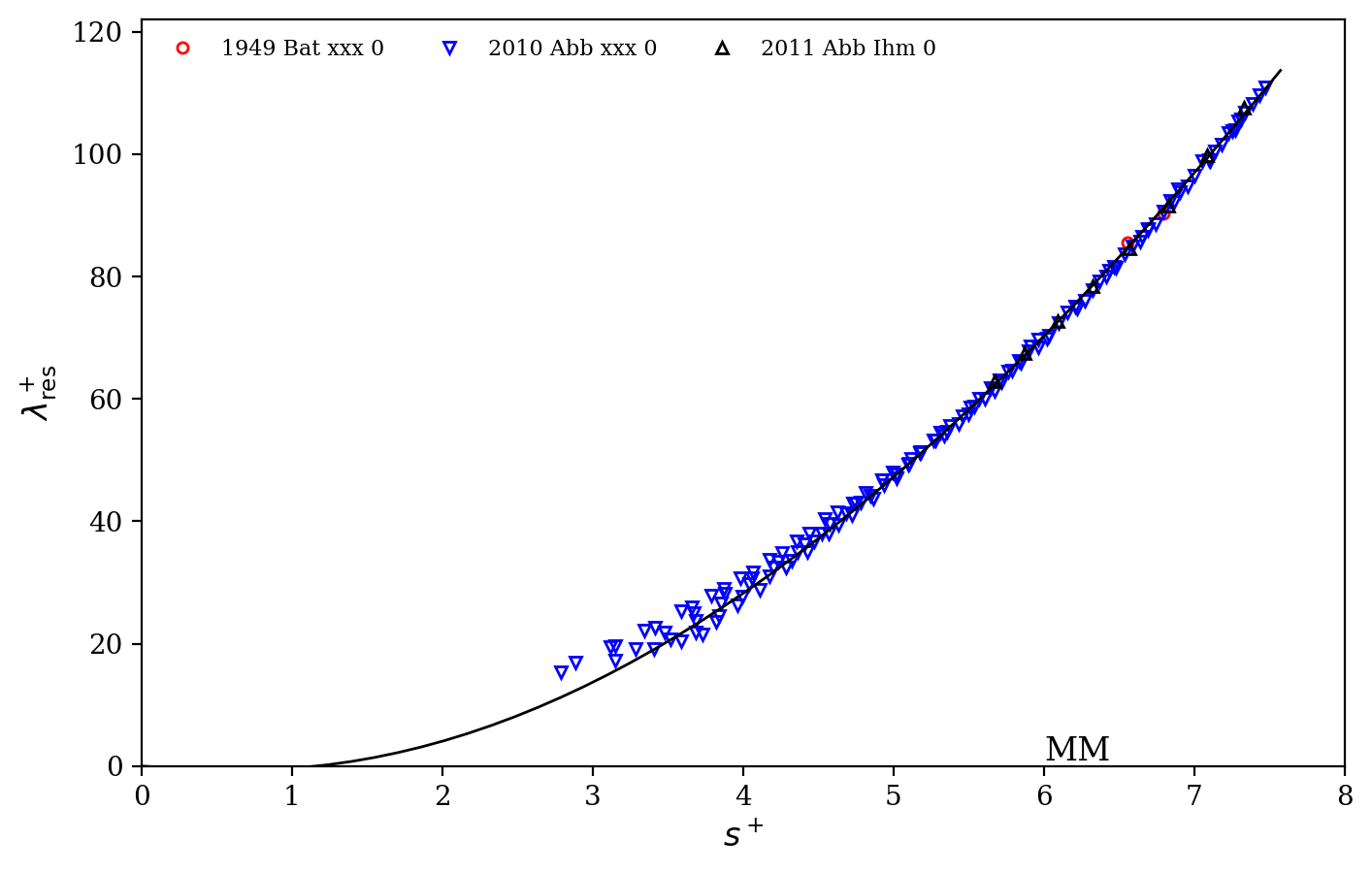

Supplement: Supplementary file 2 — ao4c10815_si_002.zip [file ao4c10815_si_002.zip › Supporting Information/Fig. TC1 - s_plus vs lambda_plus - all data - YFR EoS/MM.png]

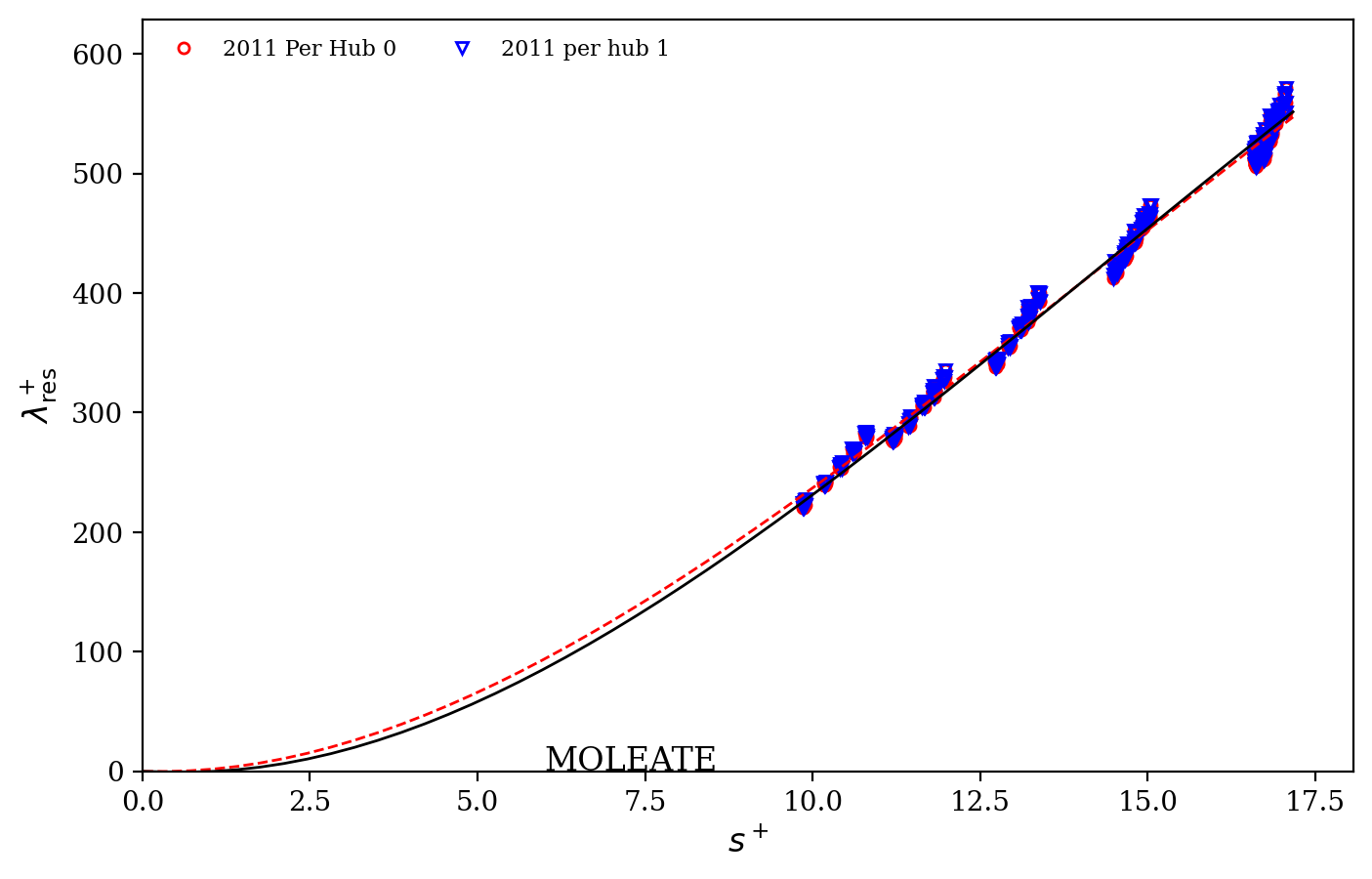

Supplement: Supplementary file 2 — ao4c10815_si_002.zip [file ao4c10815_si_002.zip › Supporting Information/Fig. TC1 - s_plus vs lambda_plus - all data - YFR EoS/MOLEATE.png]

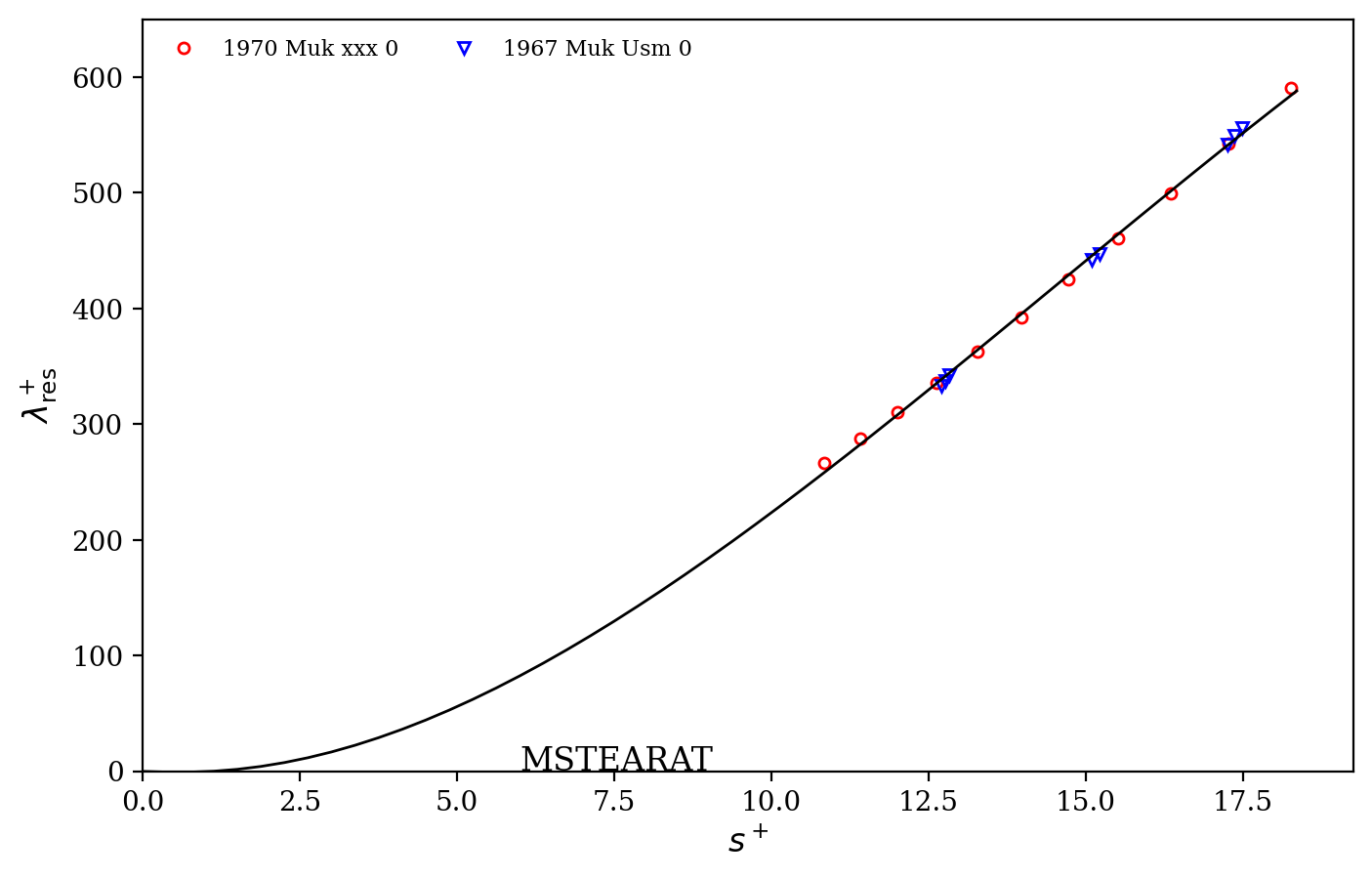

Supplement: Supplementary file 2 — ao4c10815_si_002.zip [file ao4c10815_si_002.zip › Supporting Information/Fig. TC1 - s_plus vs lambda_plus - all data - YFR EoS/MSTEARAT.png]

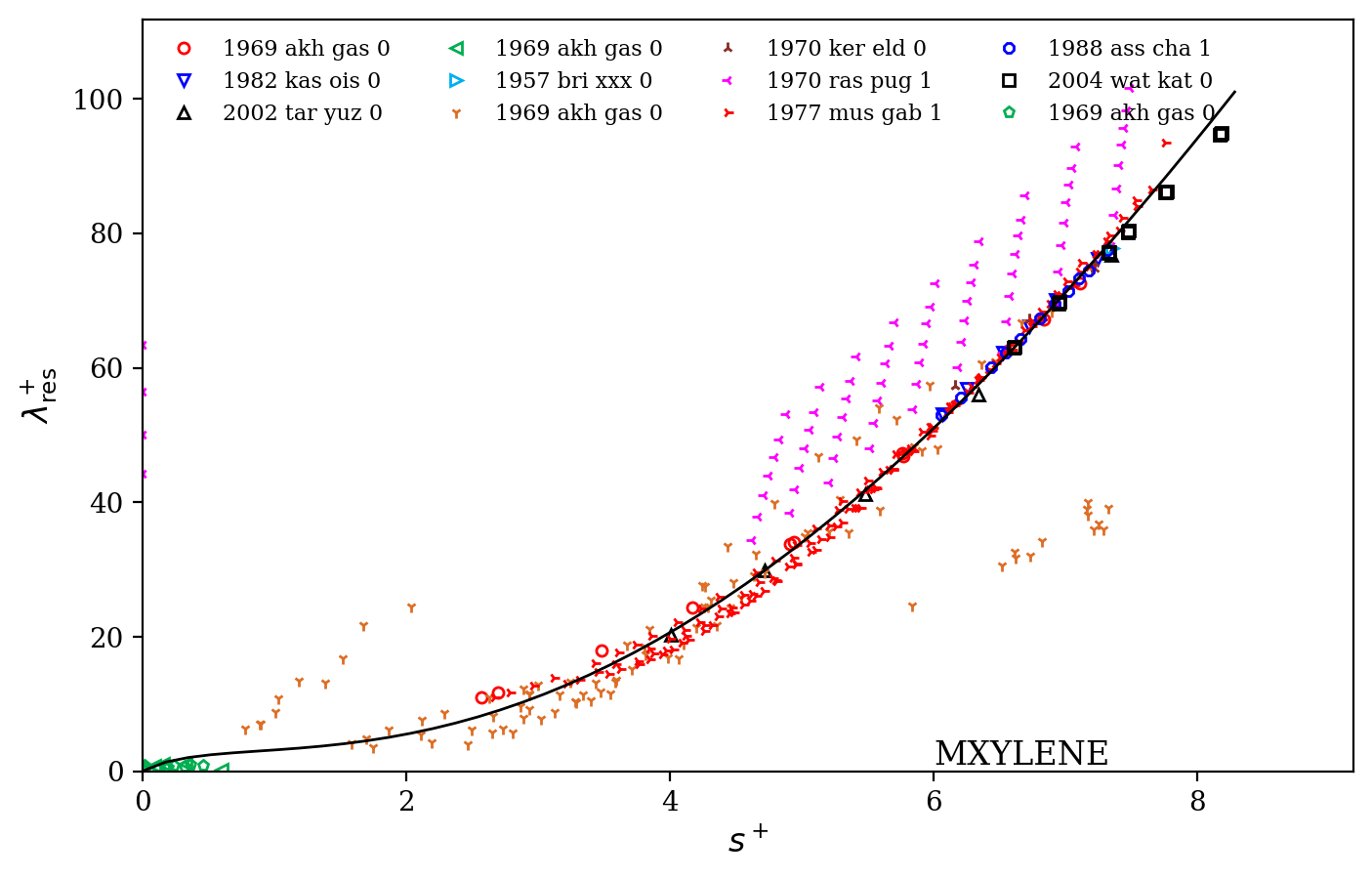

Supplement: Supplementary file 2 — ao4c10815_si_002.zip [file ao4c10815_si_002.zip › Supporting Information/Fig. TC1 - s_plus vs lambda_plus - all data - YFR EoS/MXYLENE.png]

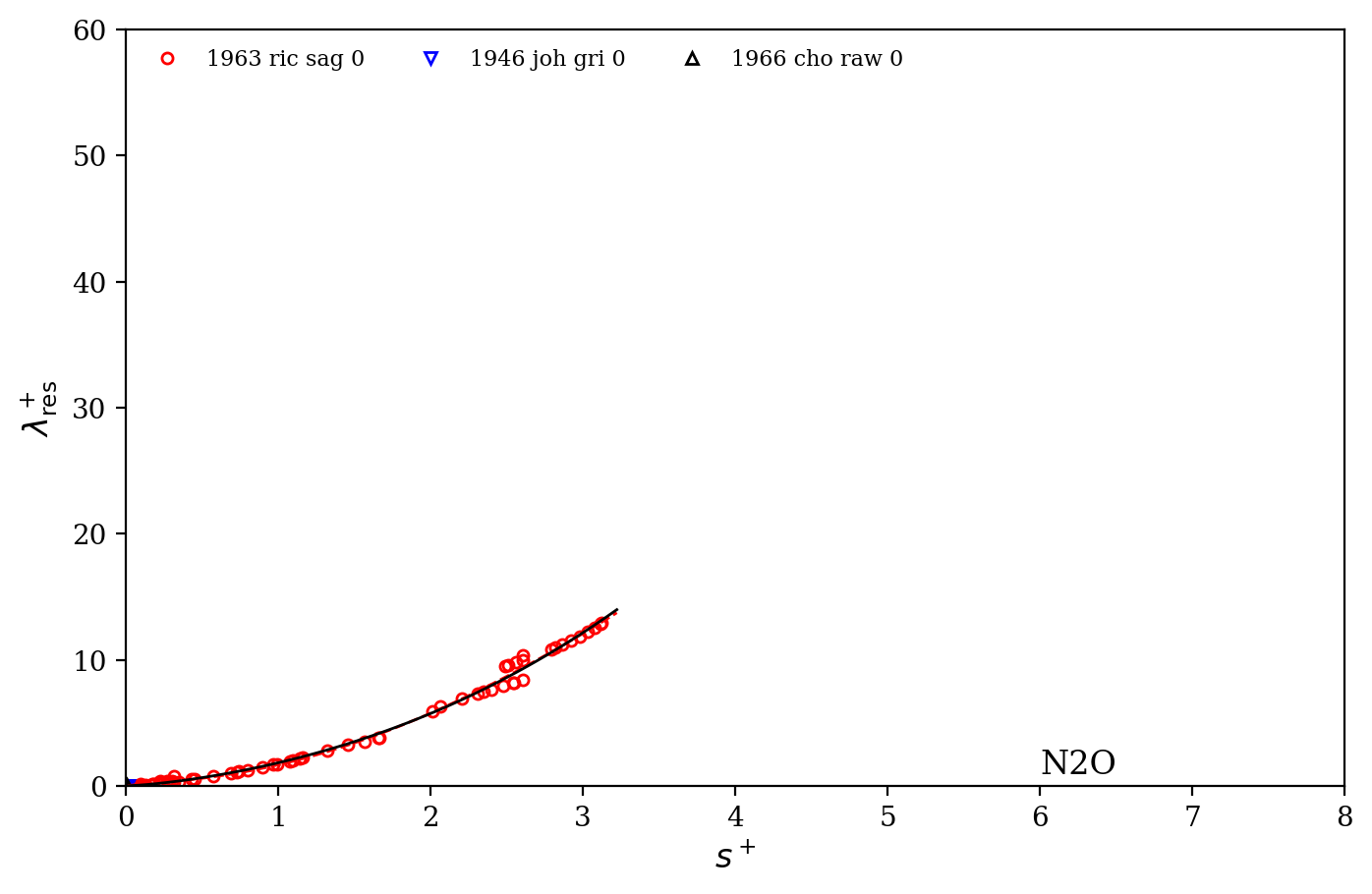

Supplement: Supplementary file 2 — ao4c10815_si_002.zip [file ao4c10815_si_002.zip › Supporting Information/Fig. TC1 - s_plus vs lambda_plus - all data - YFR EoS/N2O.png]

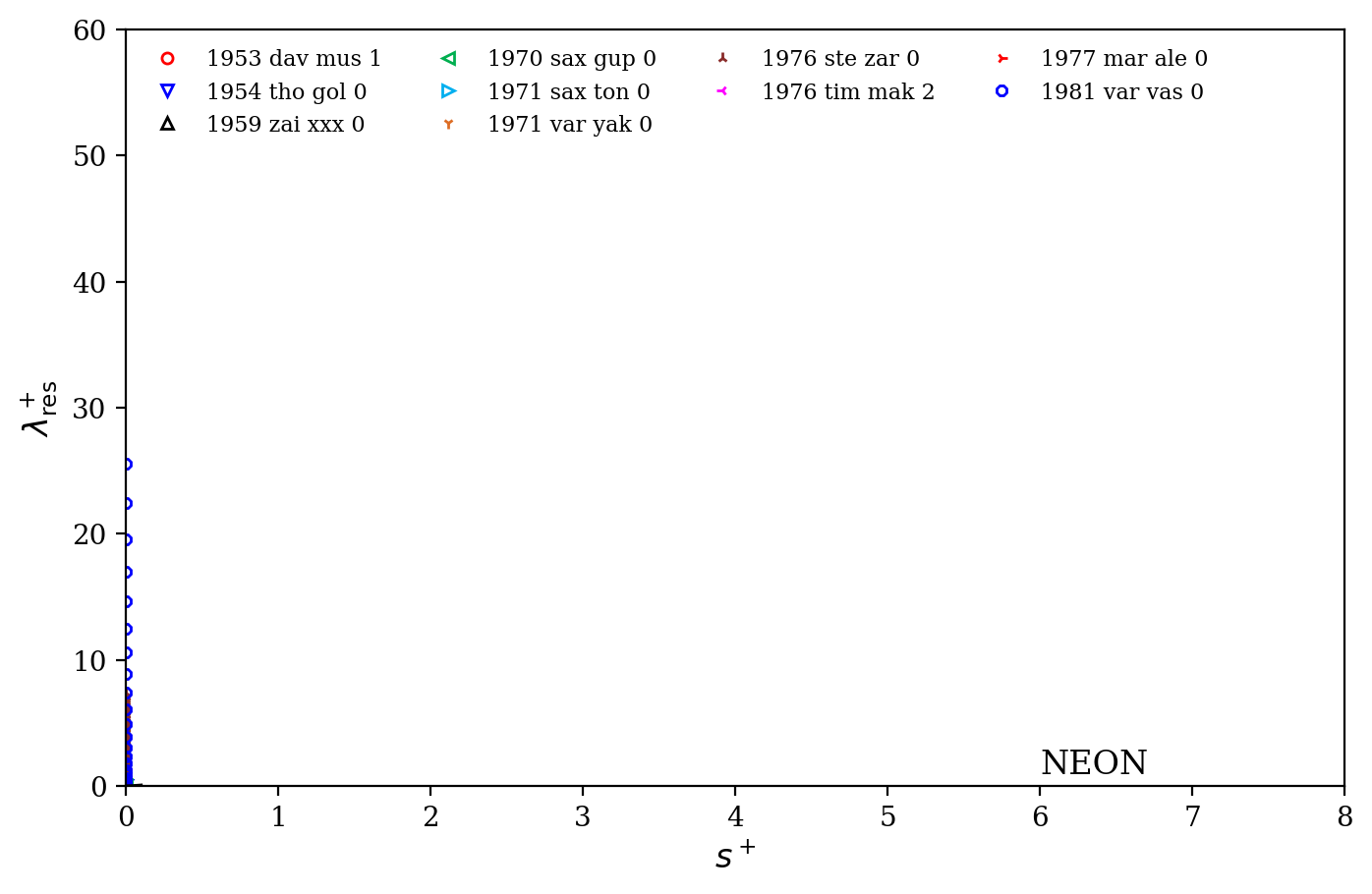

Supplement: Supplementary file 2 — ao4c10815_si_002.zip [file ao4c10815_si_002.zip › Supporting Information/Fig. TC1 - s_plus vs lambda_plus - all data - YFR EoS/NEON.png]

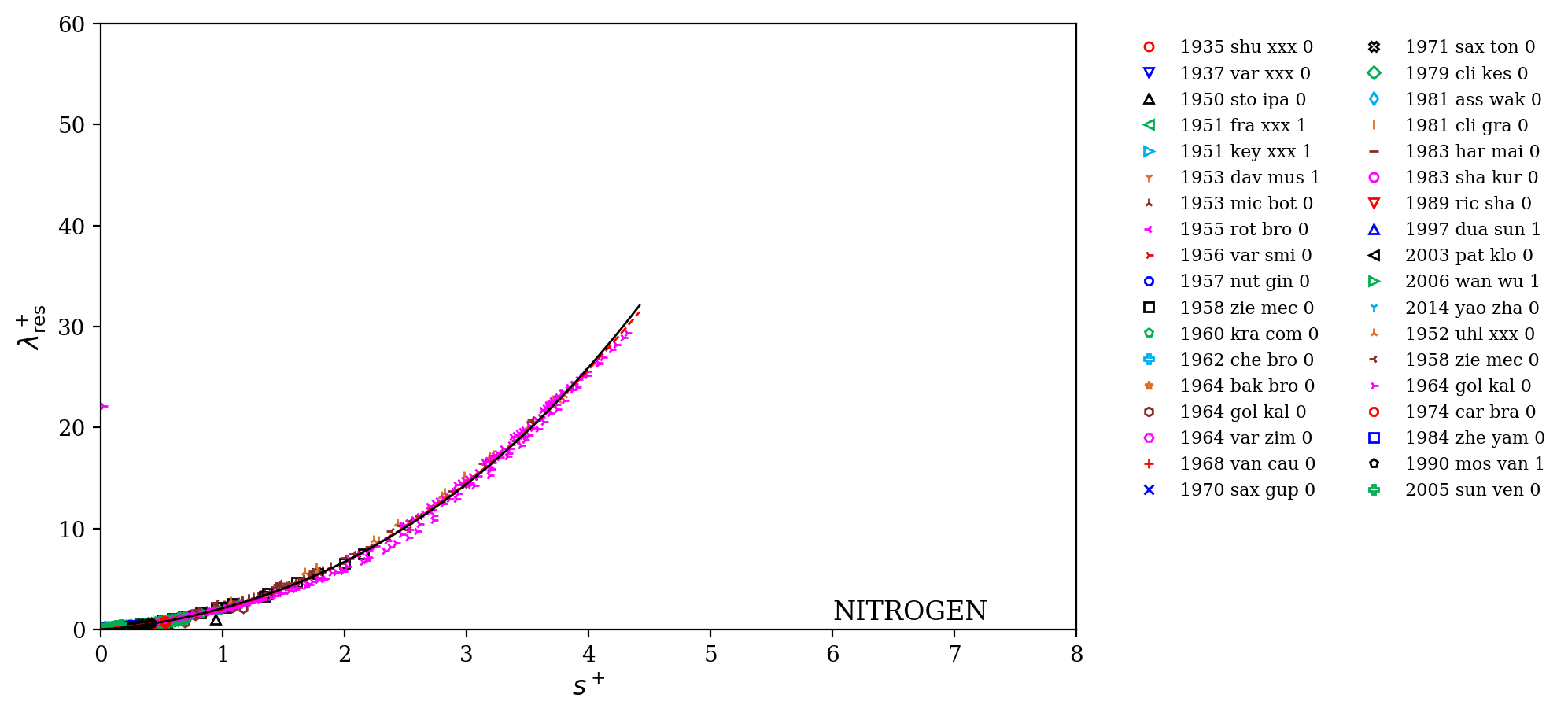

Supplement: Supplementary file 2 — ao4c10815_si_002.zip [file ao4c10815_si_002.zip › Supporting Information/Fig. TC1 - s_plus vs lambda_plus - all data - YFR EoS/NITROGEN.png]

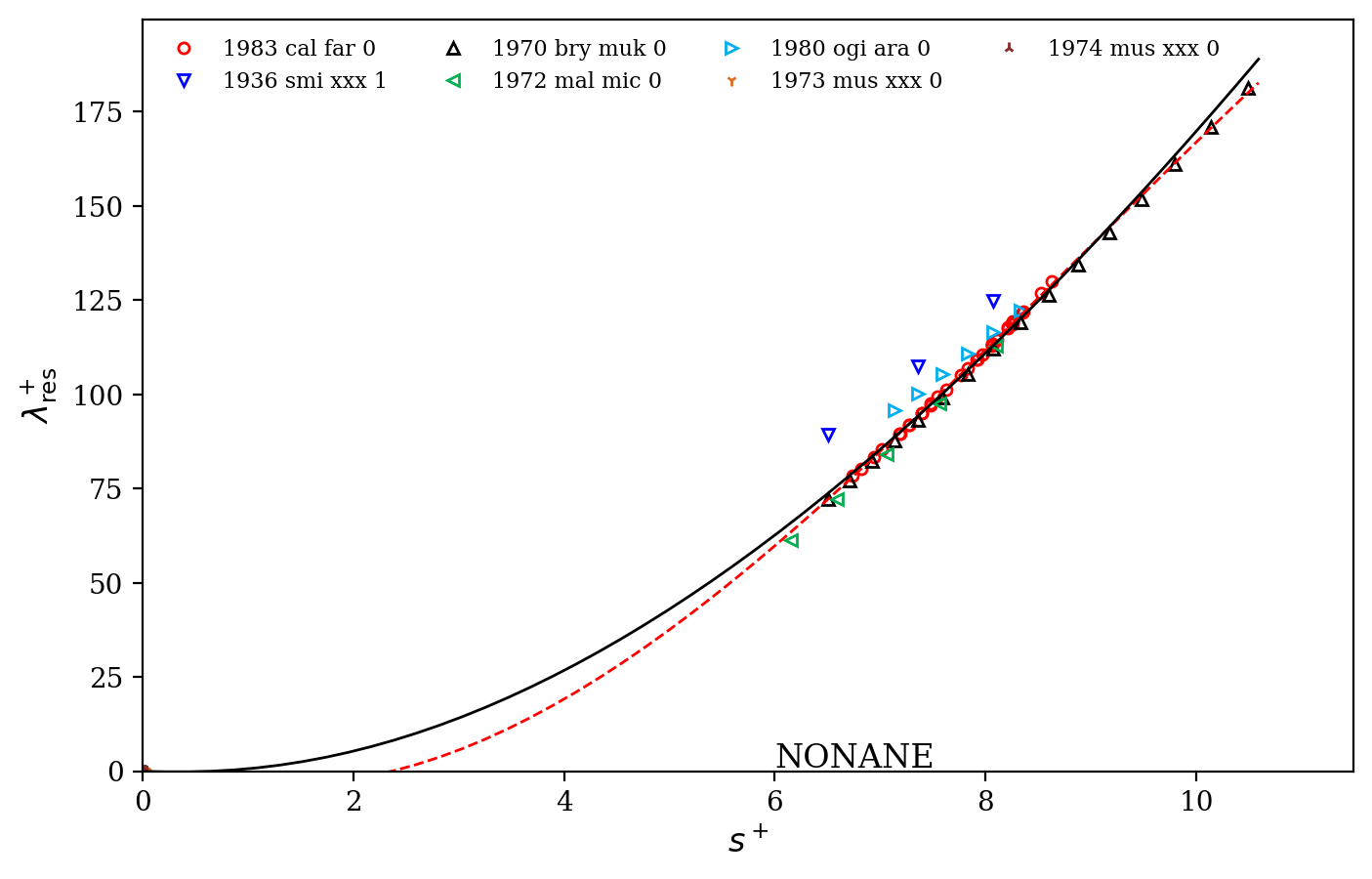

Supplement: Supplementary file 2 — ao4c10815_si_002.zip [file ao4c10815_si_002.zip › Supporting Information/Fig. TC1 - s_plus vs lambda_plus - all data - YFR EoS/NONANE.png]

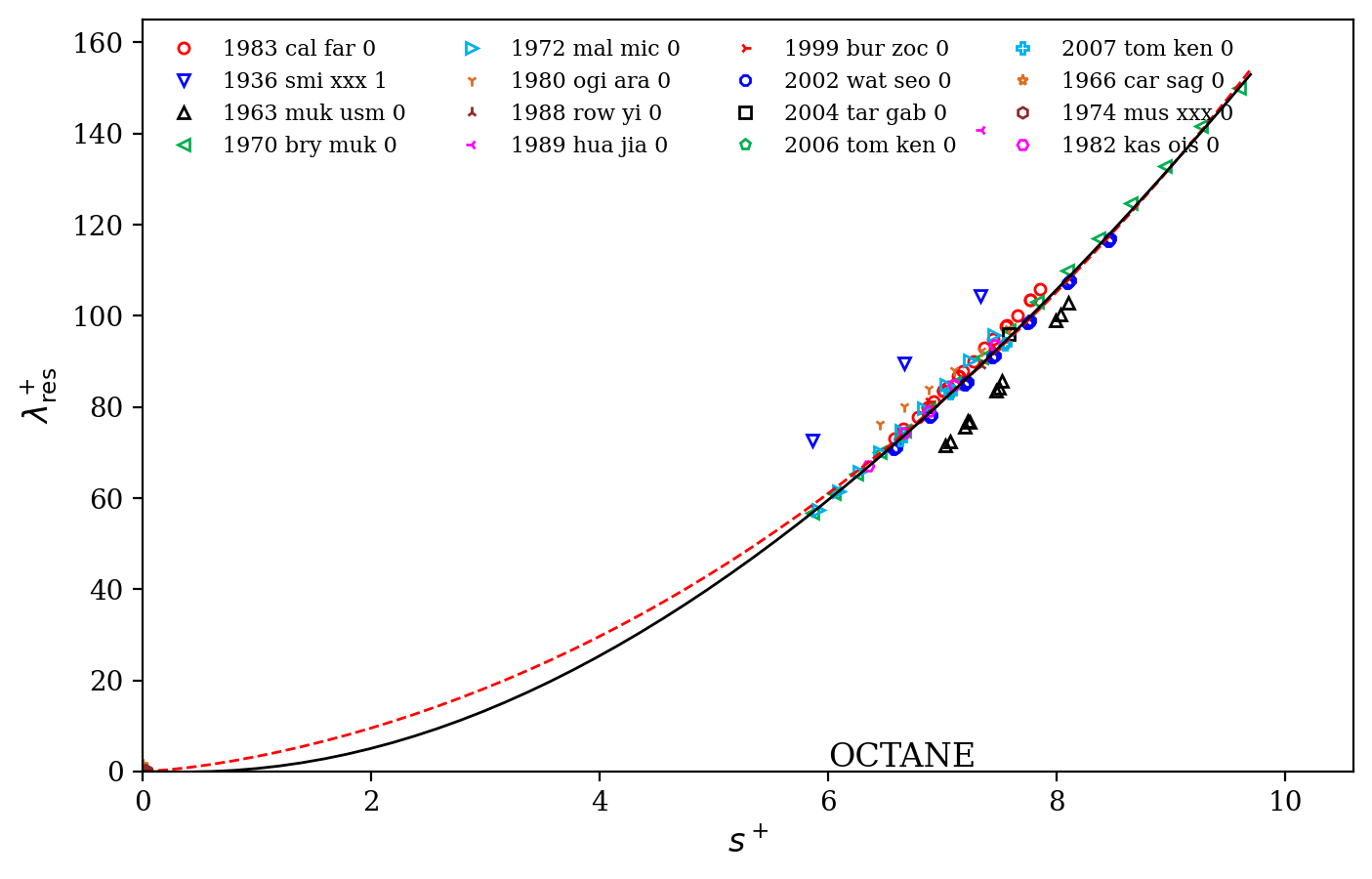

Supplement: Supplementary file 2 — ao4c10815_si_002.zip [file ao4c10815_si_002.zip › Supporting Information/Fig. TC1 - s_plus vs lambda_plus - all data - YFR EoS/OCTANE.png]

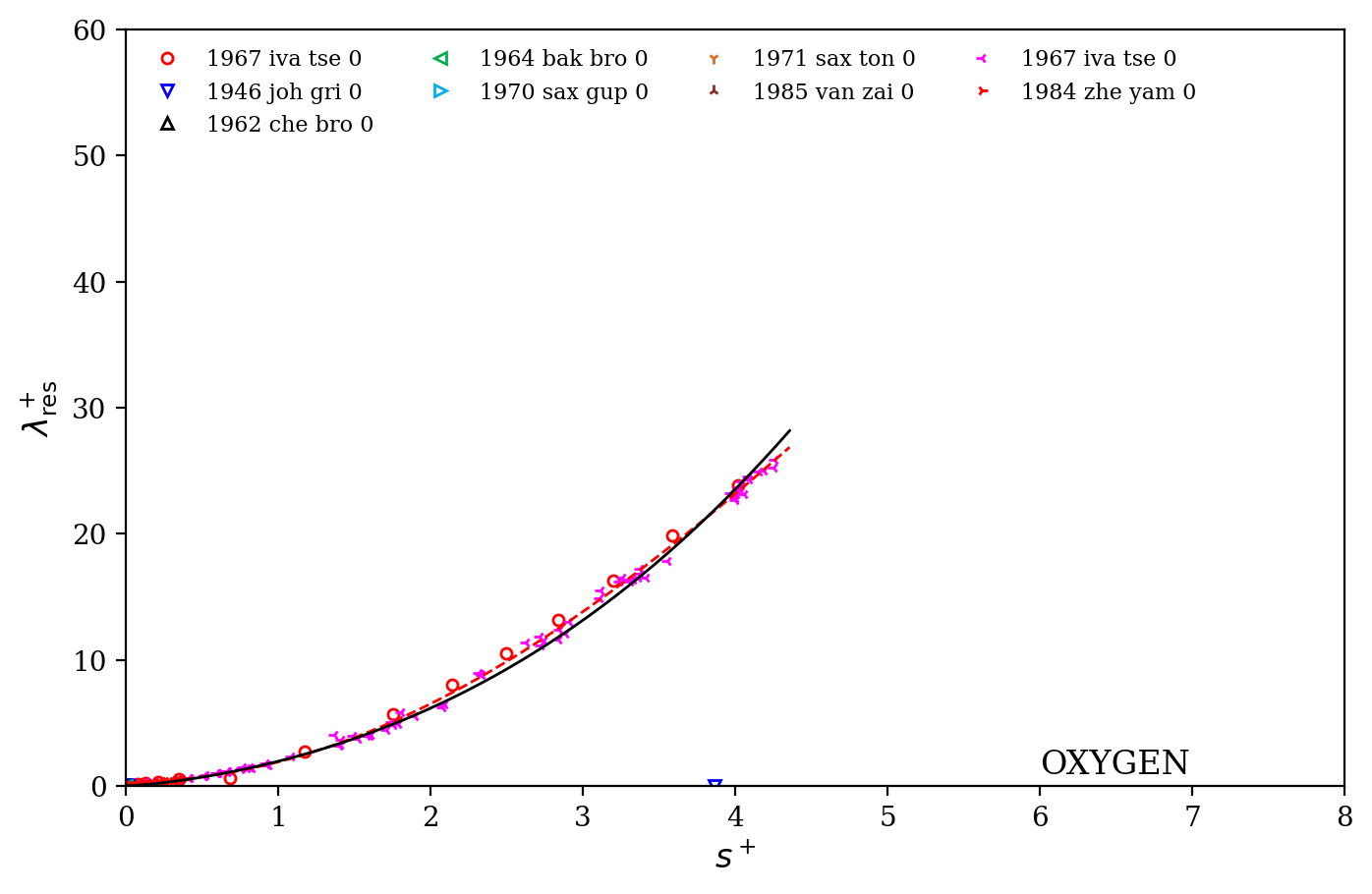

Supplement: Supplementary file 2 — ao4c10815_si_002.zip [file ao4c10815_si_002.zip › Supporting Information/Fig. TC1 - s_plus vs lambda_plus - all data - YFR EoS/OXYGEN.png]

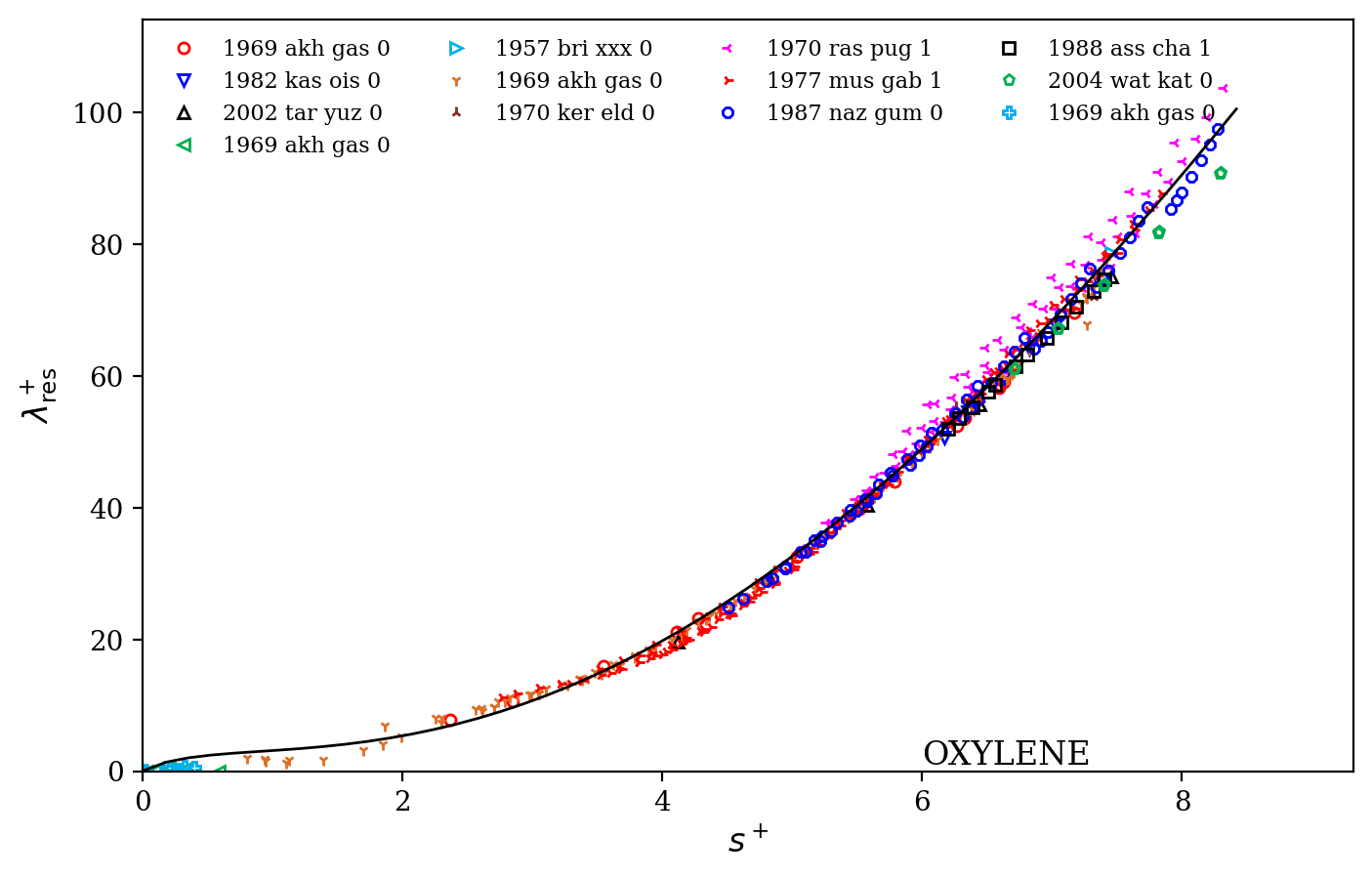

Supplement: Supplementary file 2 — ao4c10815_si_002.zip [file ao4c10815_si_002.zip › Supporting Information/Fig. TC1 - s_plus vs lambda_plus - all data - YFR EoS/OXYLENE.png]

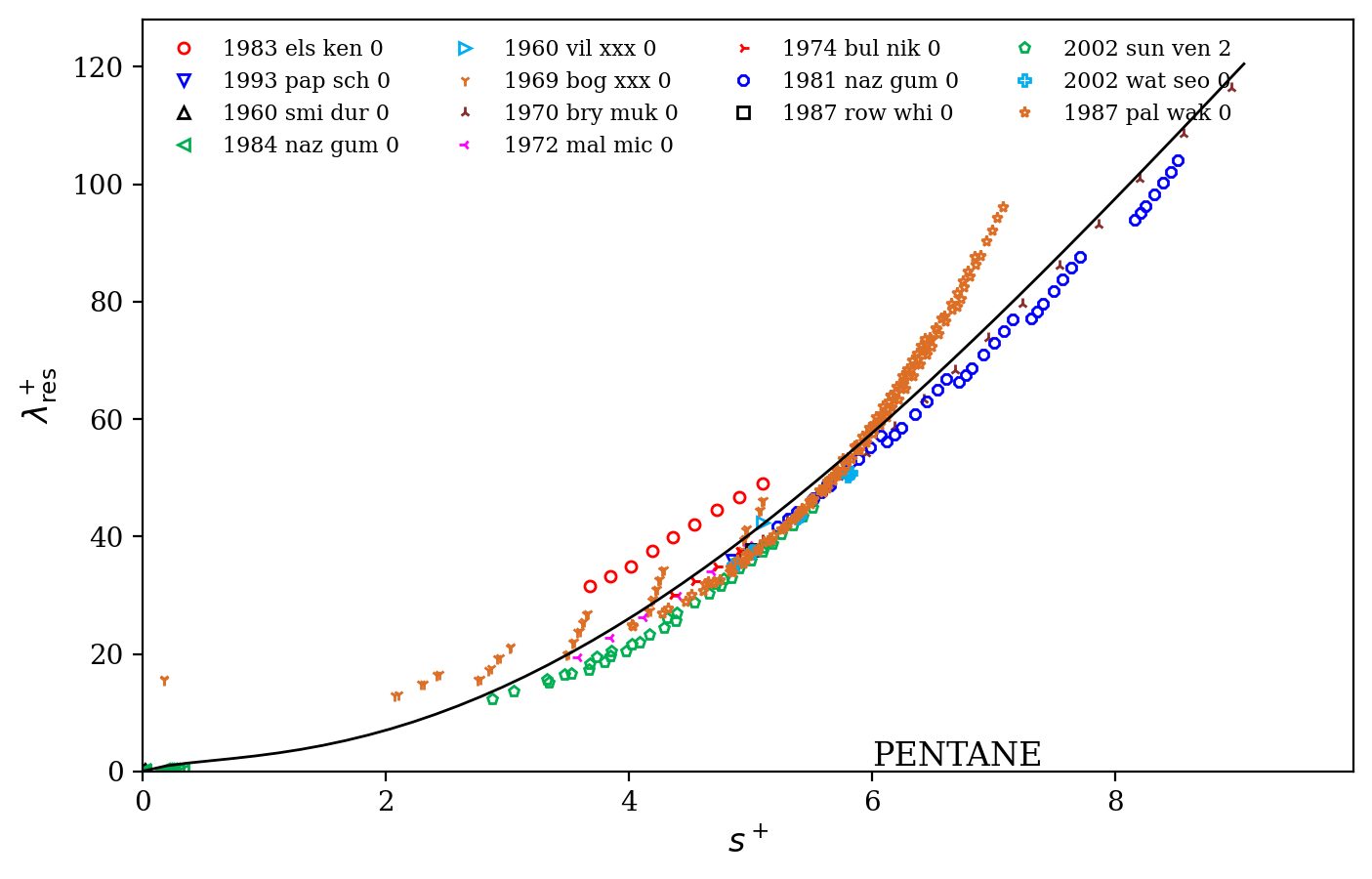

Supplement: Supplementary file 2 — ao4c10815_si_002.zip [file ao4c10815_si_002.zip › Supporting Information/Fig. TC1 - s_plus vs lambda_plus - all data - YFR EoS/PENTANE.png]

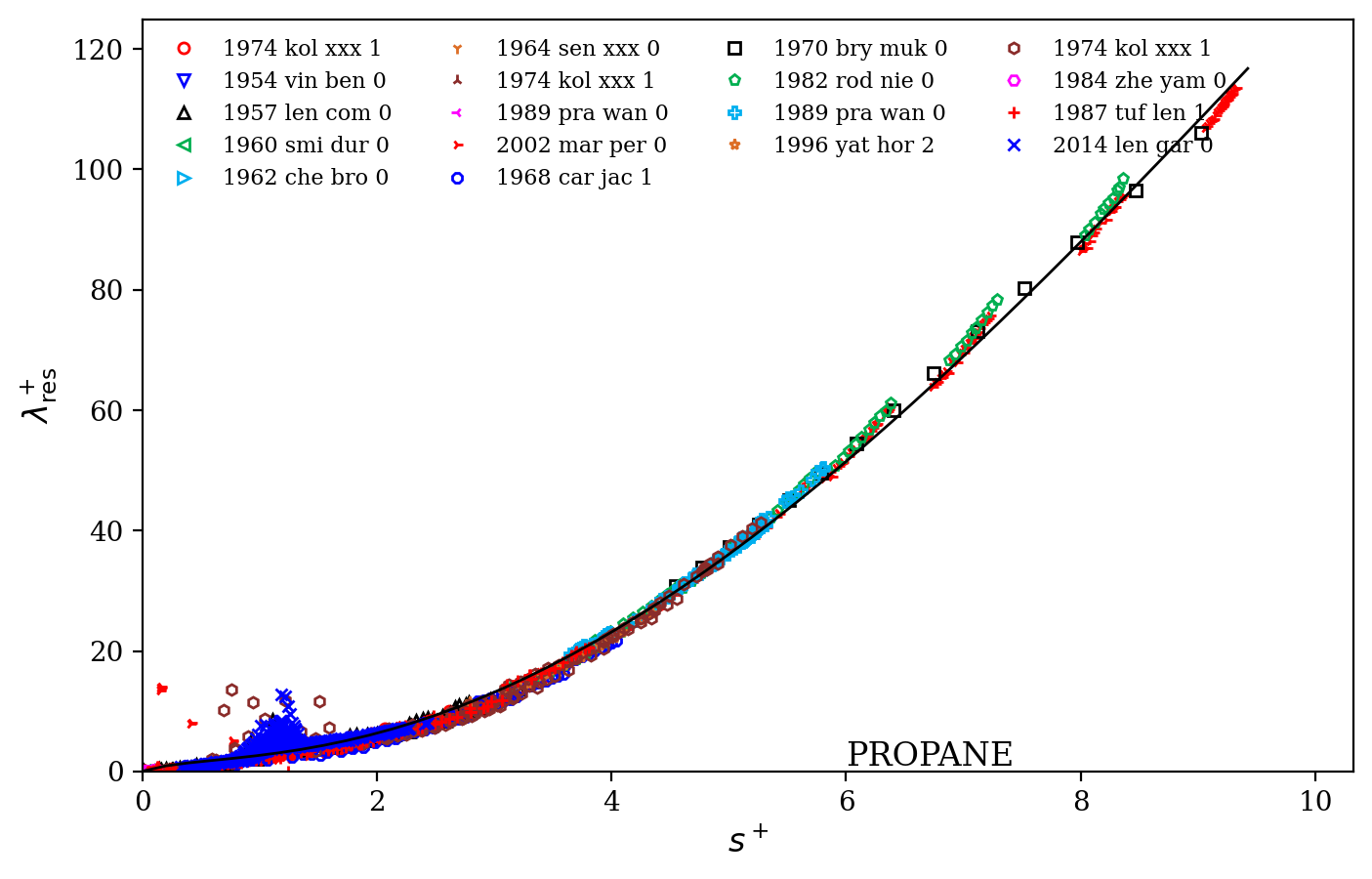

Supplement: Supplementary file 2 — ao4c10815_si_002.zip [file ao4c10815_si_002.zip › Supporting Information/Fig. TC1 - s_plus vs lambda_plus - all data - YFR EoS/PROPANE.png]

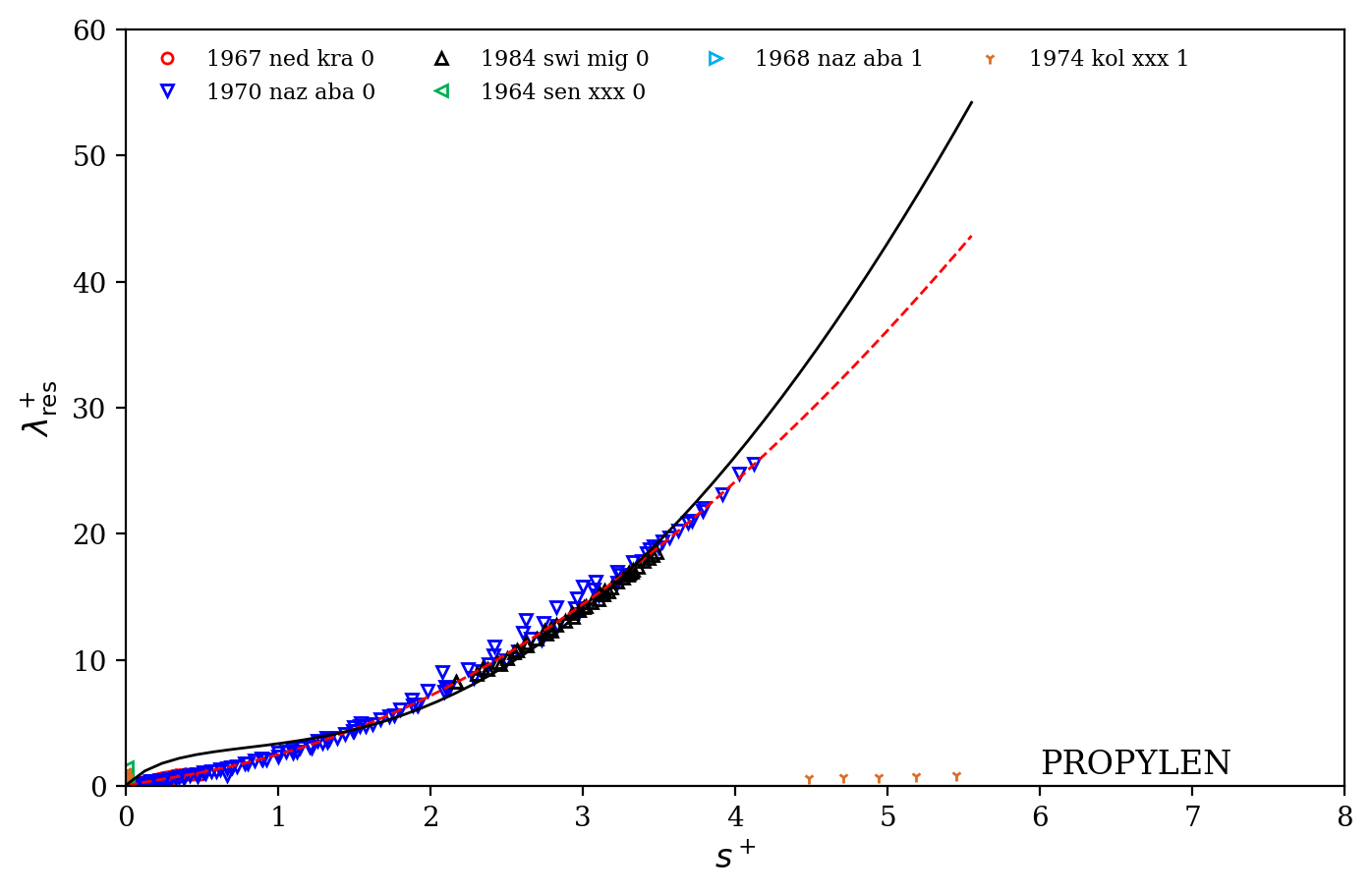

Supplement: Supplementary file 2 — ao4c10815_si_002.zip [file ao4c10815_si_002.zip › Supporting Information/Fig. TC1 - s_plus vs lambda_plus - all data - YFR EoS/PROPYLEN.png]

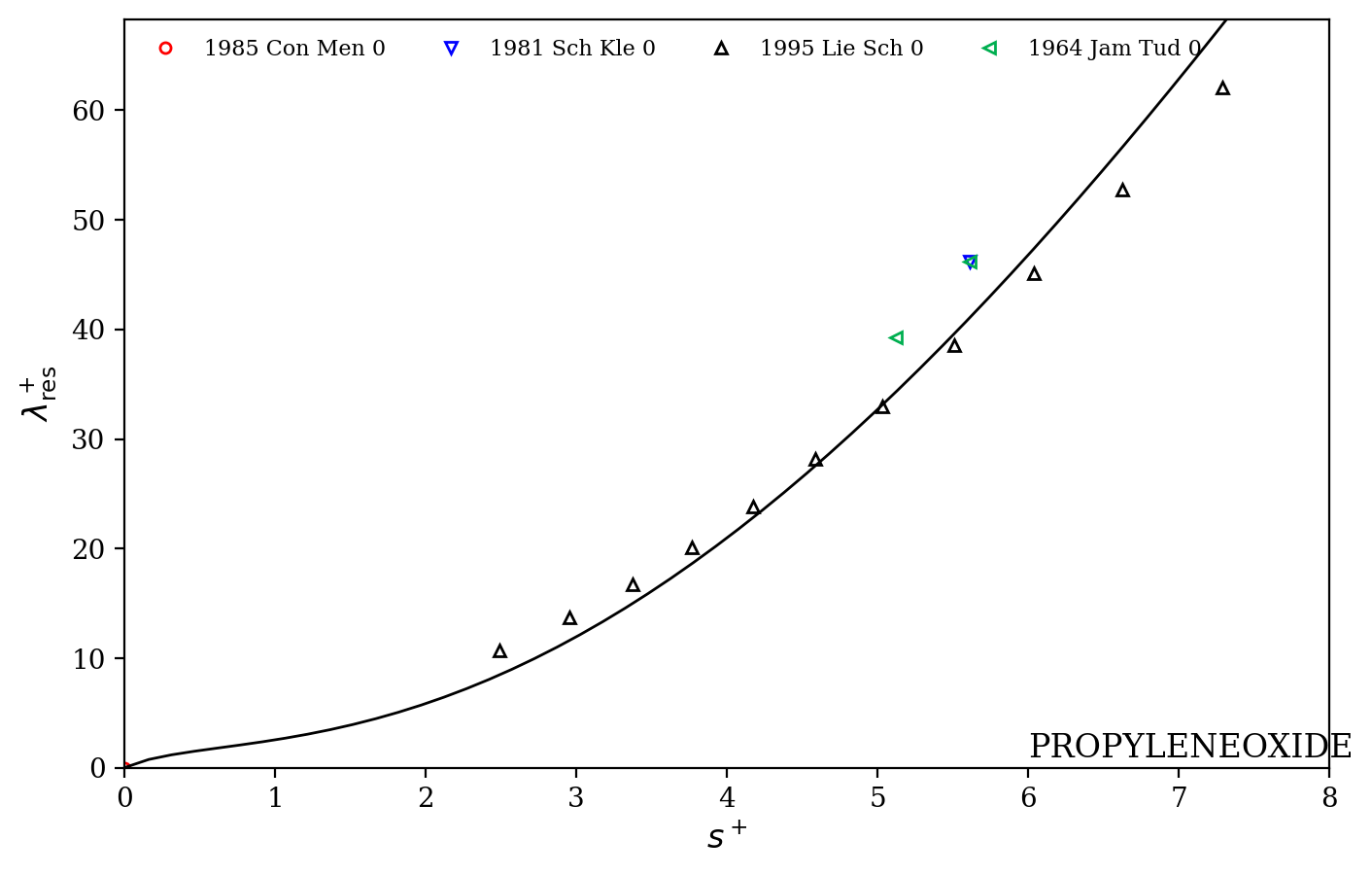

Supplement: Supplementary file 2 — ao4c10815_si_002.zip [file ao4c10815_si_002.zip › Supporting Information/Fig. TC1 - s_plus vs lambda_plus - all data - YFR EoS/PROPYLENEOXIDE.png]

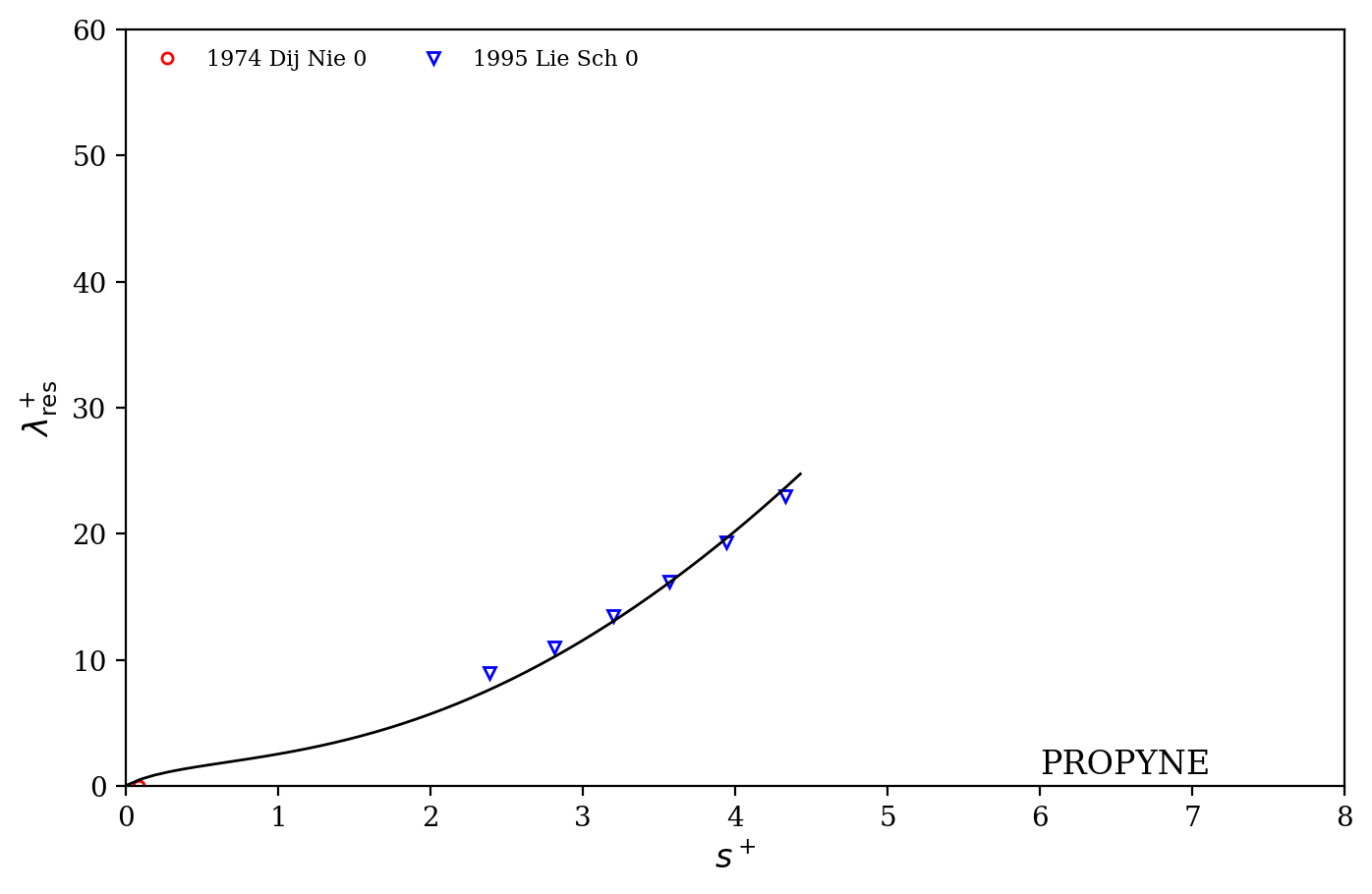

Supplement: Supplementary file 2 — ao4c10815_si_002.zip [file ao4c10815_si_002.zip › Supporting Information/Fig. TC1 - s_plus vs lambda_plus - all data - YFR EoS/PROPYNE.png]

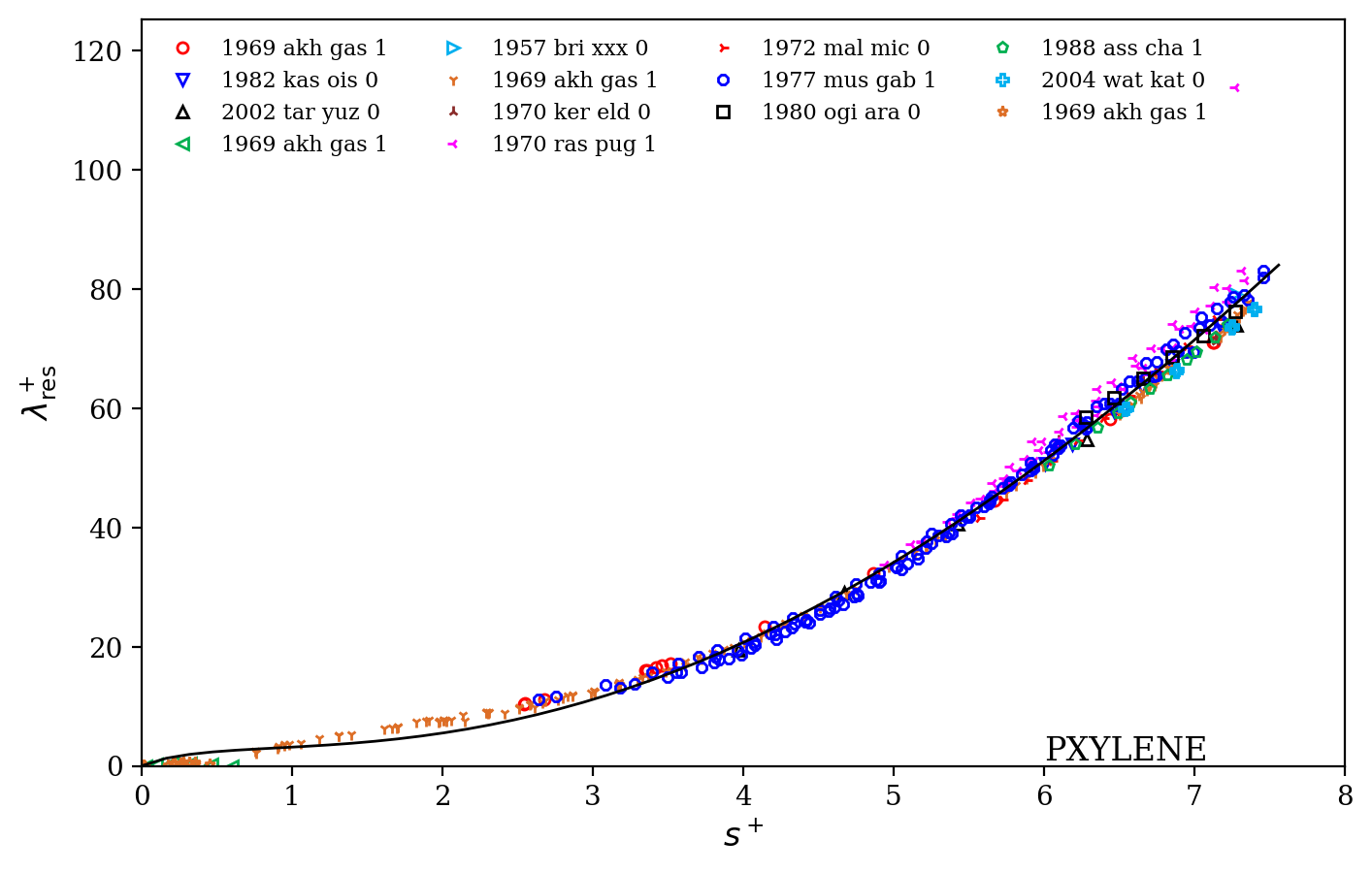

Supplement: Supplementary file 2 — ao4c10815_si_002.zip [file ao4c10815_si_002.zip › Supporting Information/Fig. TC1 - s_plus vs lambda_plus - all data - YFR EoS/PXYLENE.png]

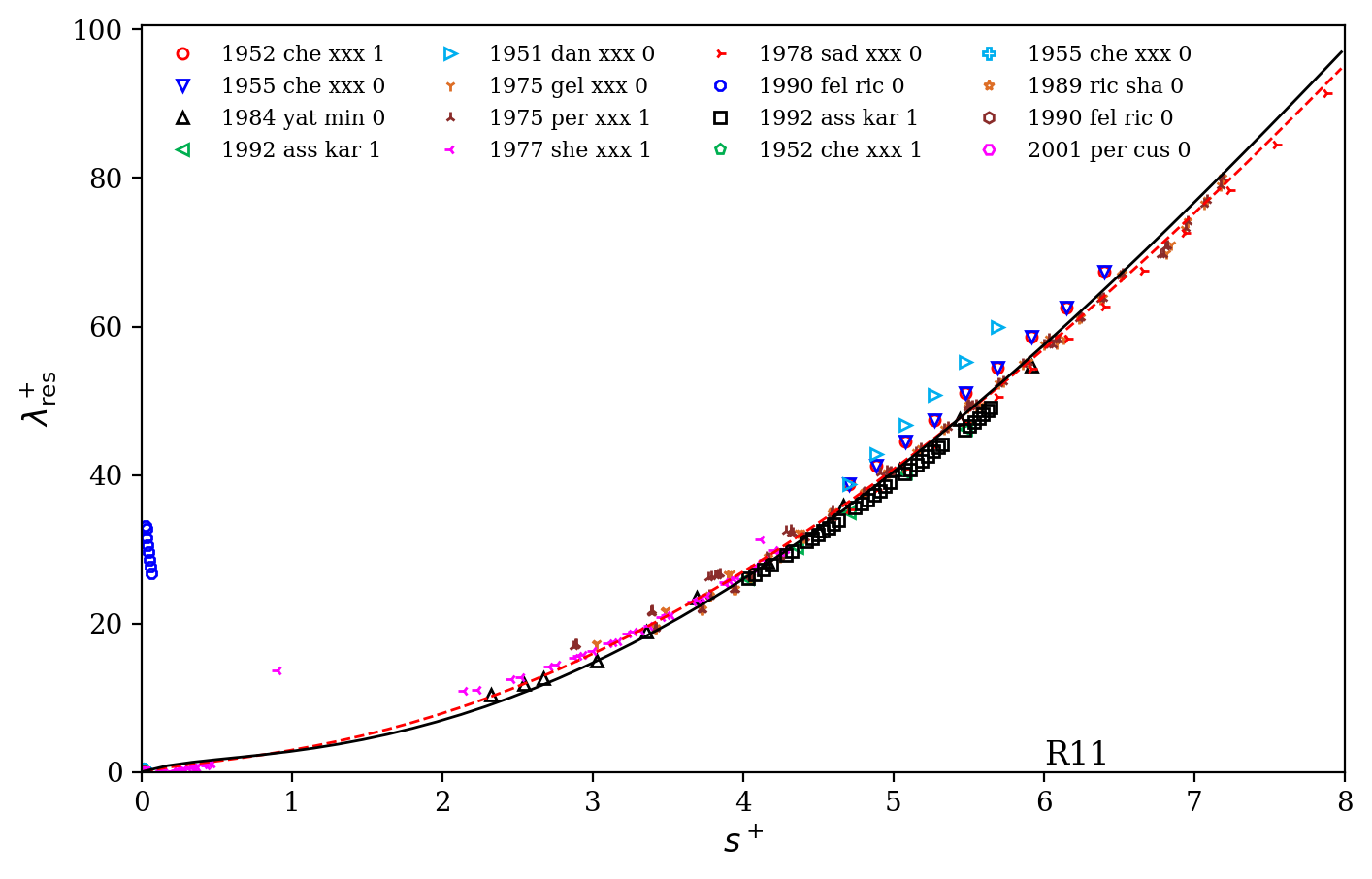

Supplement: Supplementary file 2 — ao4c10815_si_002.zip [file ao4c10815_si_002.zip › Supporting Information/Fig. TC1 - s_plus vs lambda_plus - all data - YFR EoS/R11.png]

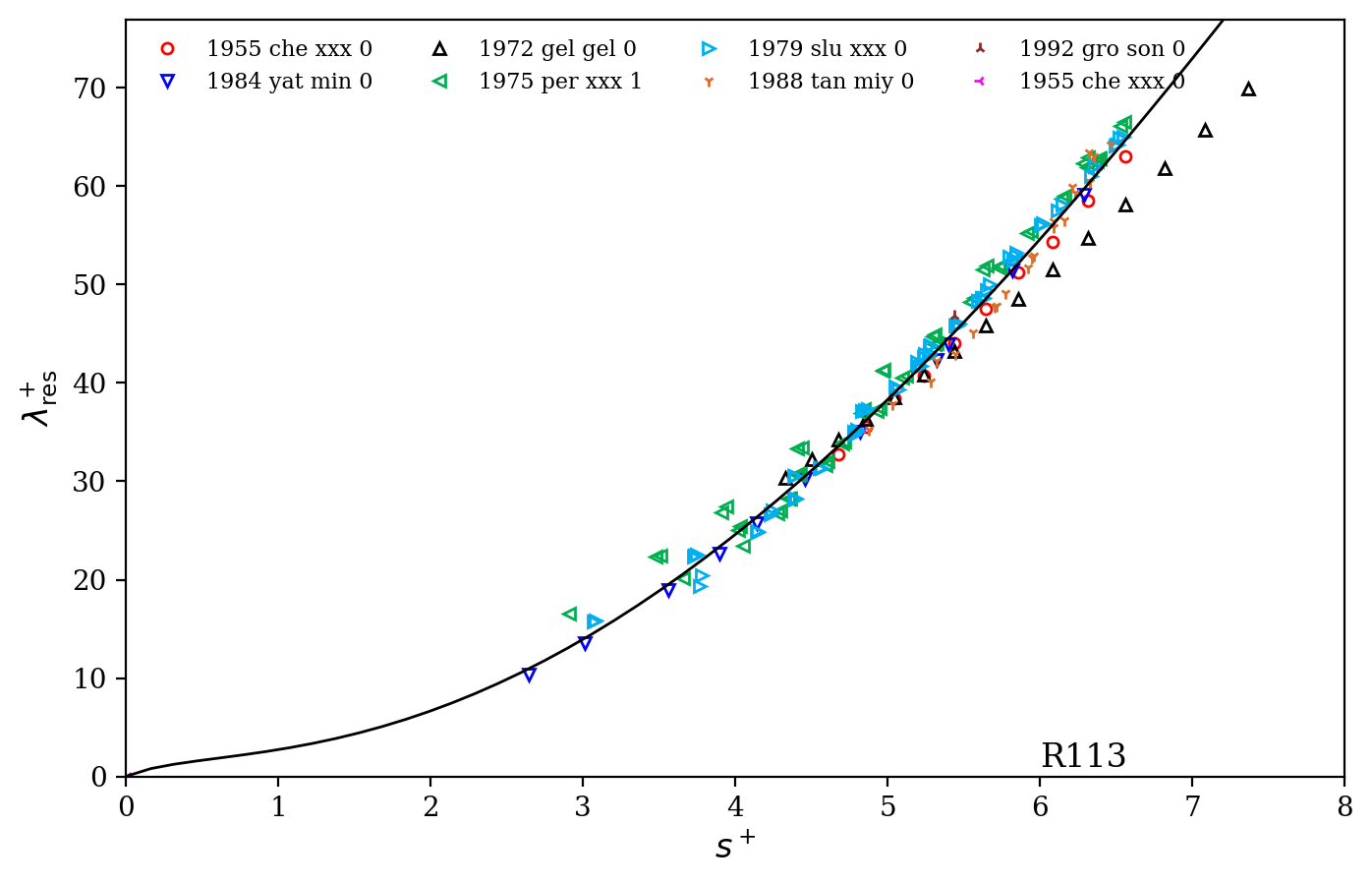

Supplement: Supplementary file 2 — ao4c10815_si_002.zip [file ao4c10815_si_002.zip › Supporting Information/Fig. TC1 - s_plus vs lambda_plus - all data - YFR EoS/R113.png]

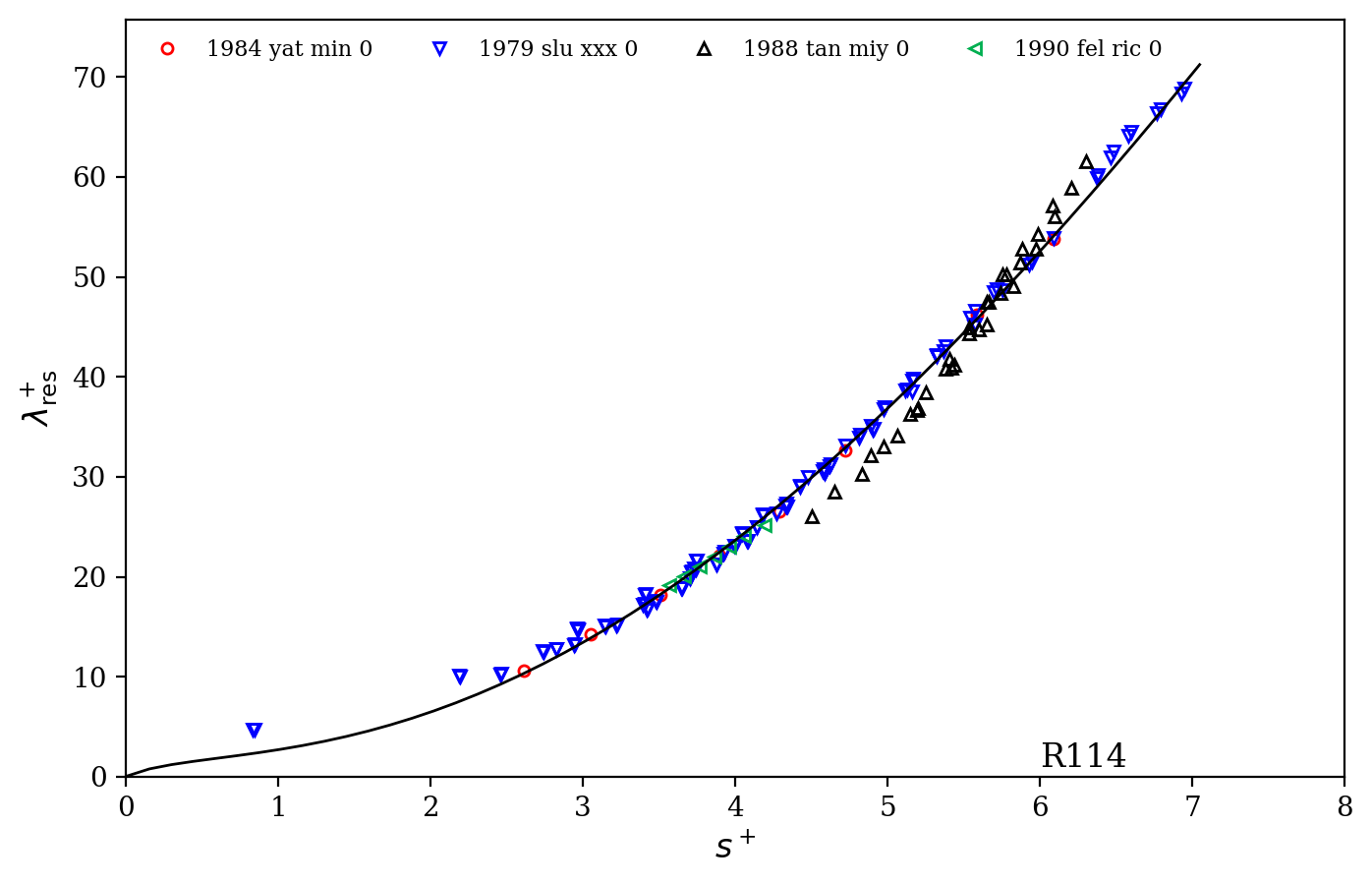

Supplement: Supplementary file 2 — ao4c10815_si_002.zip [file ao4c10815_si_002.zip › Supporting Information/Fig. TC1 - s_plus vs lambda_plus - all data - YFR EoS/R114.png]

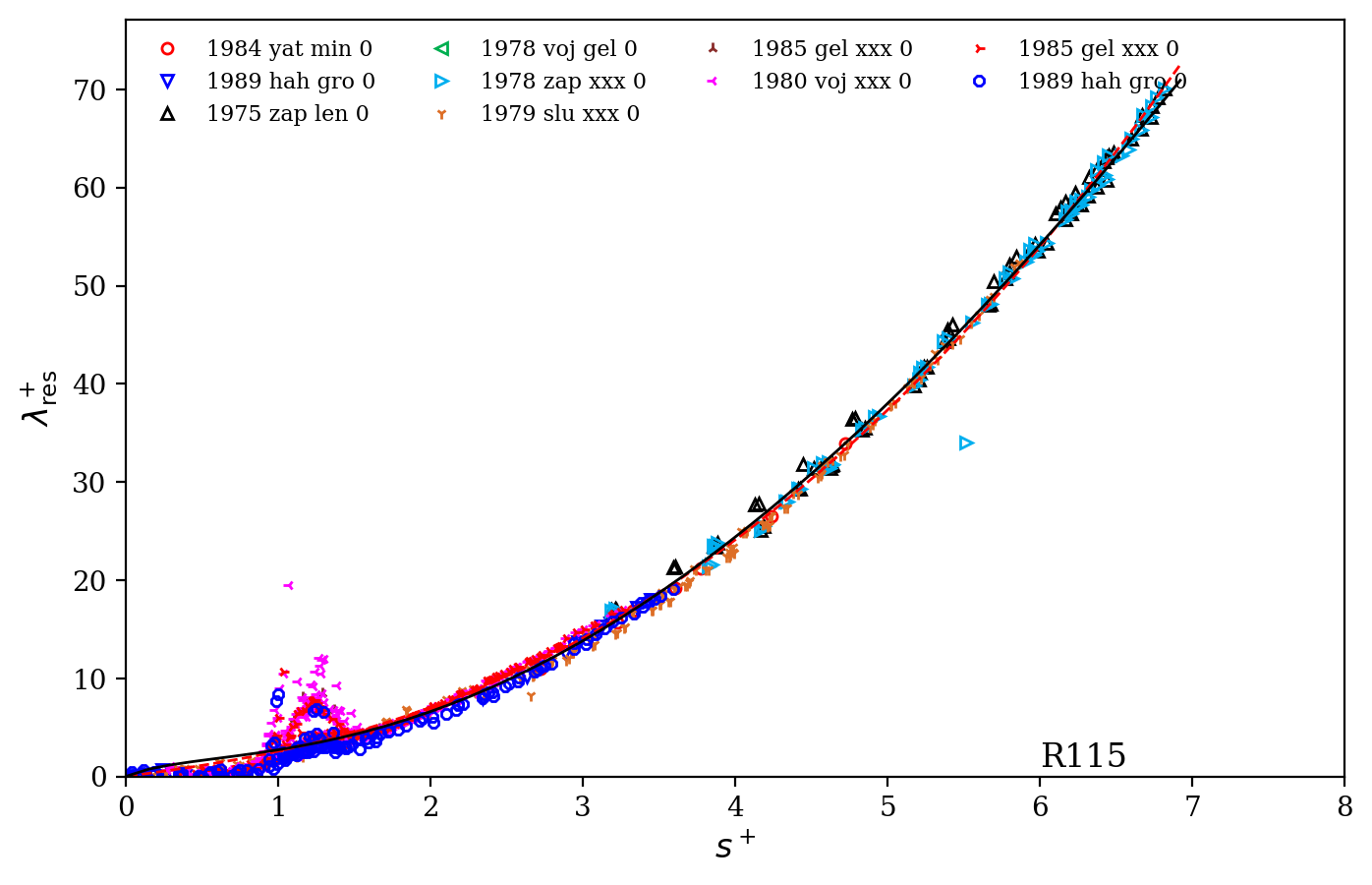

Supplement: Supplementary file 2 — ao4c10815_si_002.zip [file ao4c10815_si_002.zip › Supporting Information/Fig. TC1 - s_plus vs lambda_plus - all data - YFR EoS/R115.png]

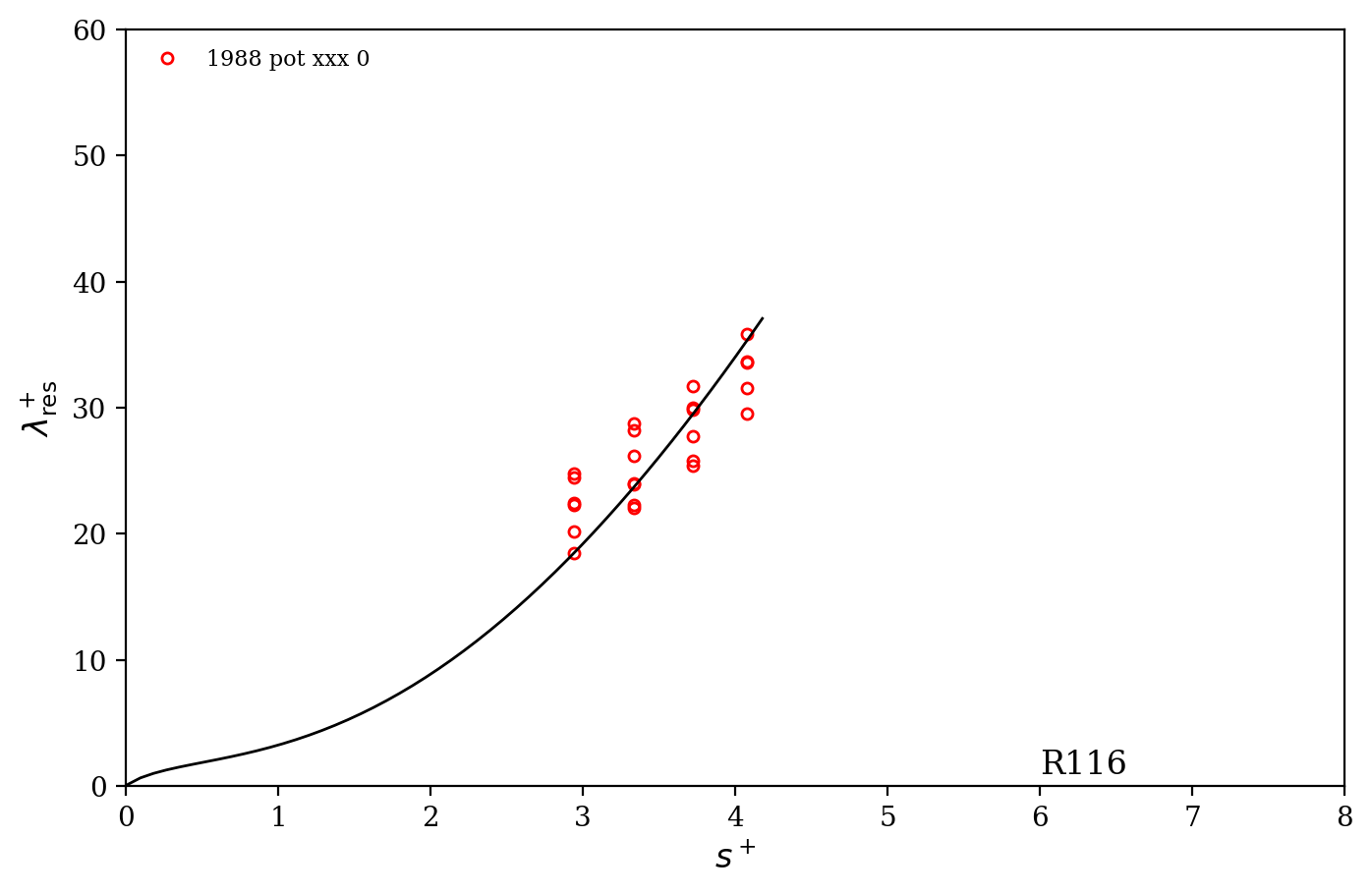

Supplement: Supplementary file 2 — ao4c10815_si_002.zip [file ao4c10815_si_002.zip › Supporting Information/Fig. TC1 - s_plus vs lambda_plus - all data - YFR EoS/R116.png]

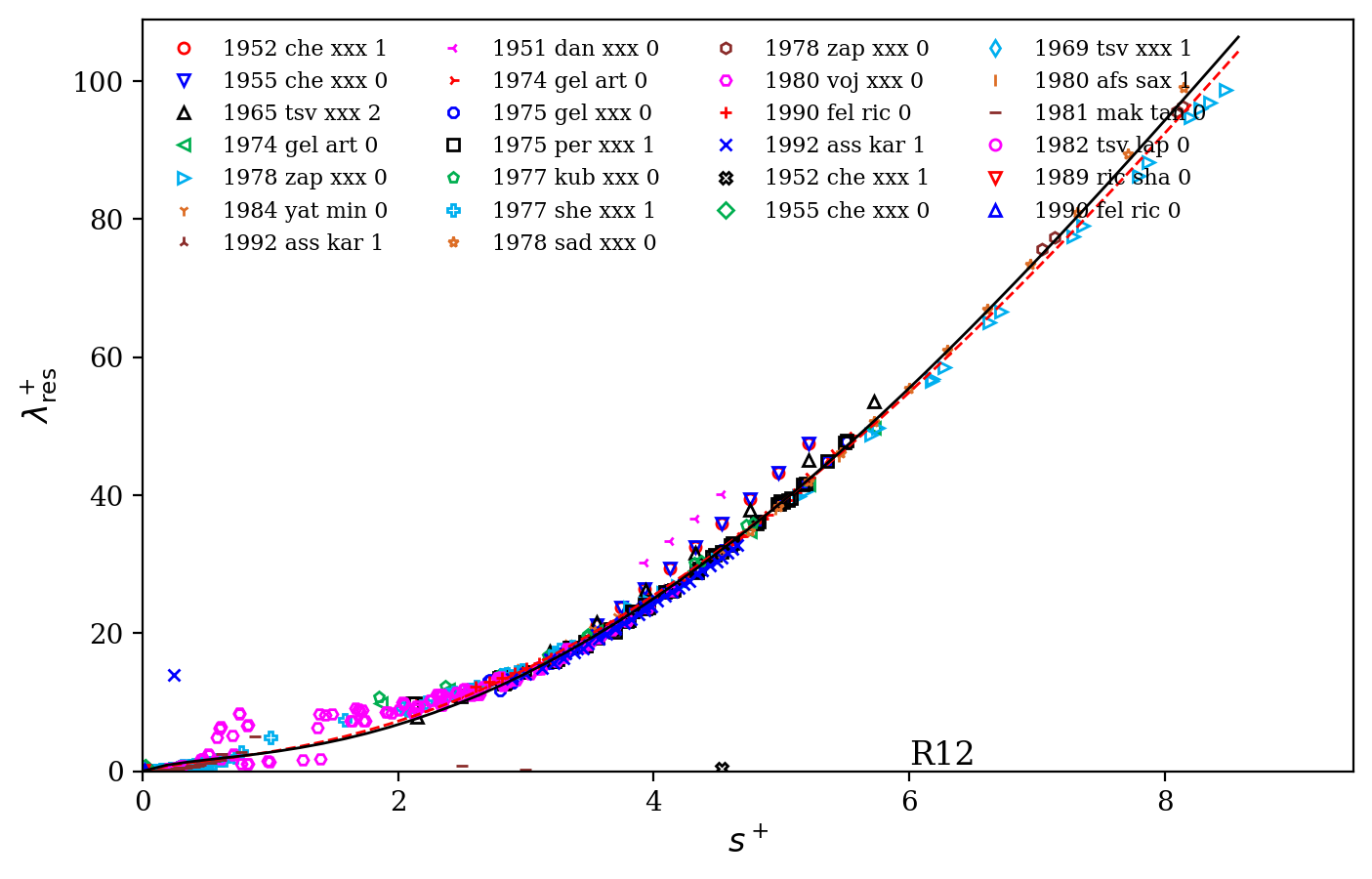

Supplement: Supplementary file 2 — ao4c10815_si_002.zip [file ao4c10815_si_002.zip › Supporting Information/Fig. TC1 - s_plus vs lambda_plus - all data - YFR EoS/R12.png]

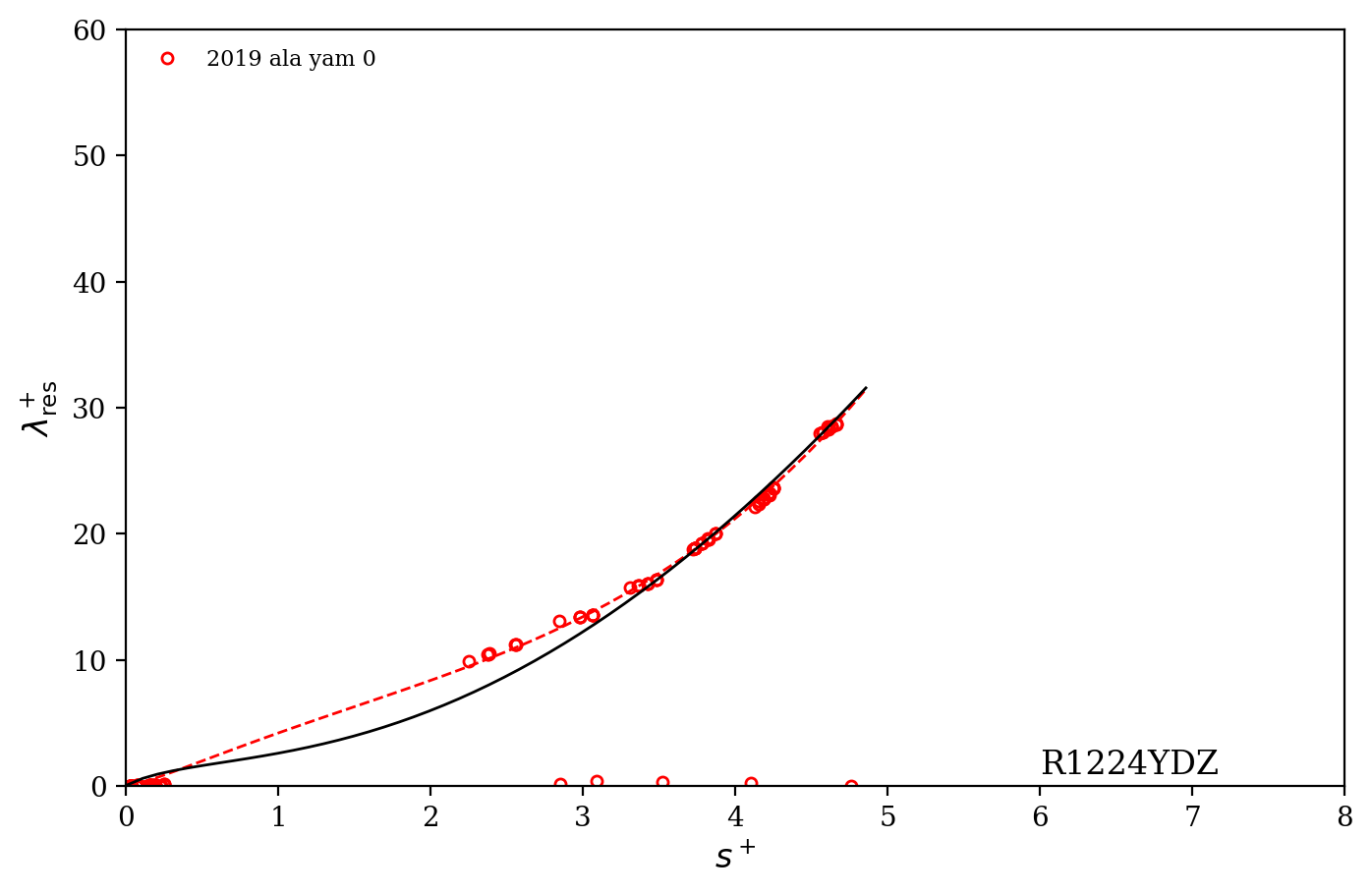

Supplement: Supplementary file 2 — ao4c10815_si_002.zip [file ao4c10815_si_002.zip › Supporting Information/Fig. TC1 - s_plus vs lambda_plus - all data - YFR EoS/R1224YDZ.png]

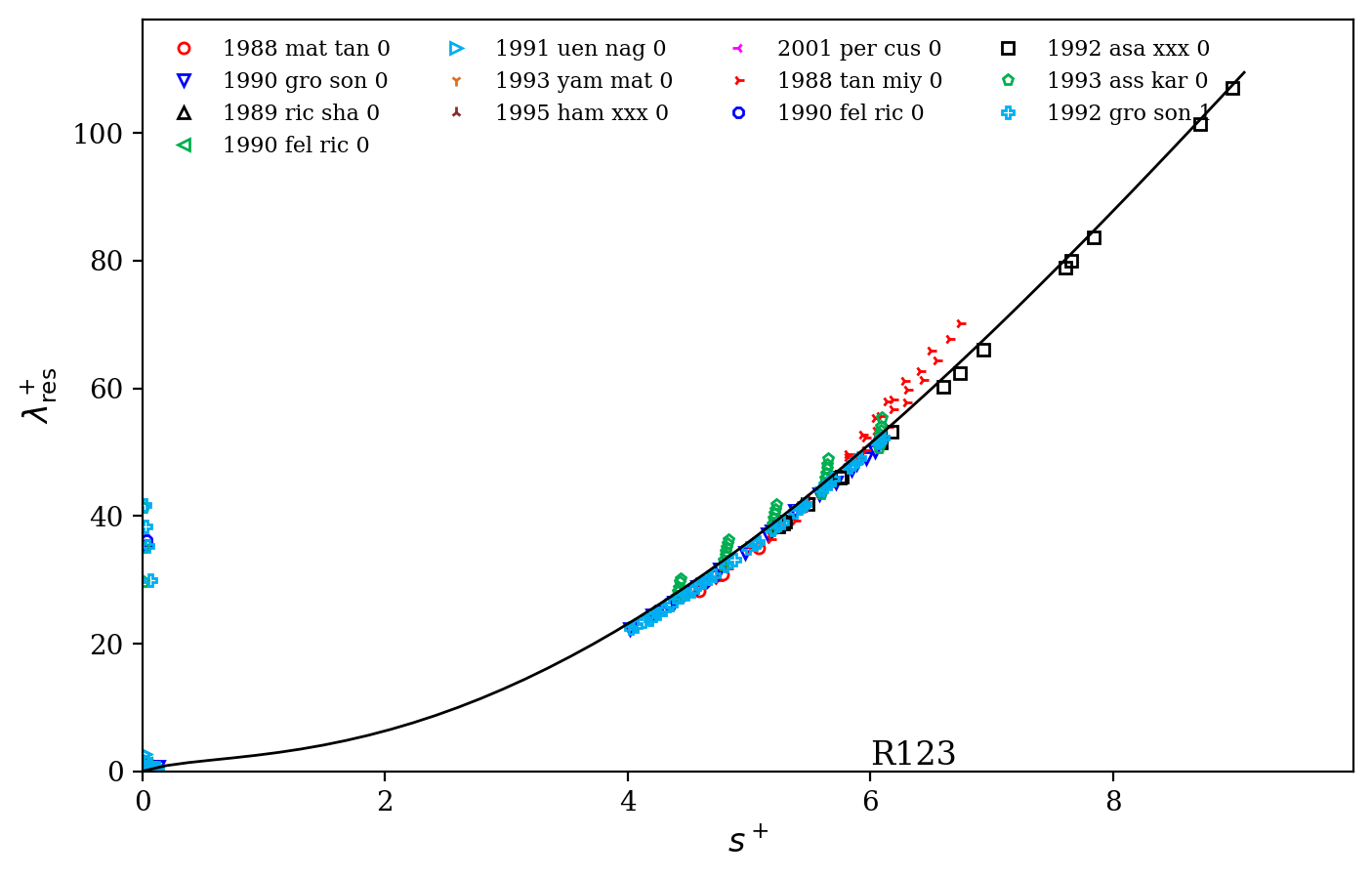

Supplement: Supplementary file 2 — ao4c10815_si_002.zip [file ao4c10815_si_002.zip › Supporting Information/Fig. TC1 - s_plus vs lambda_plus - all data - YFR EoS/R123.png]

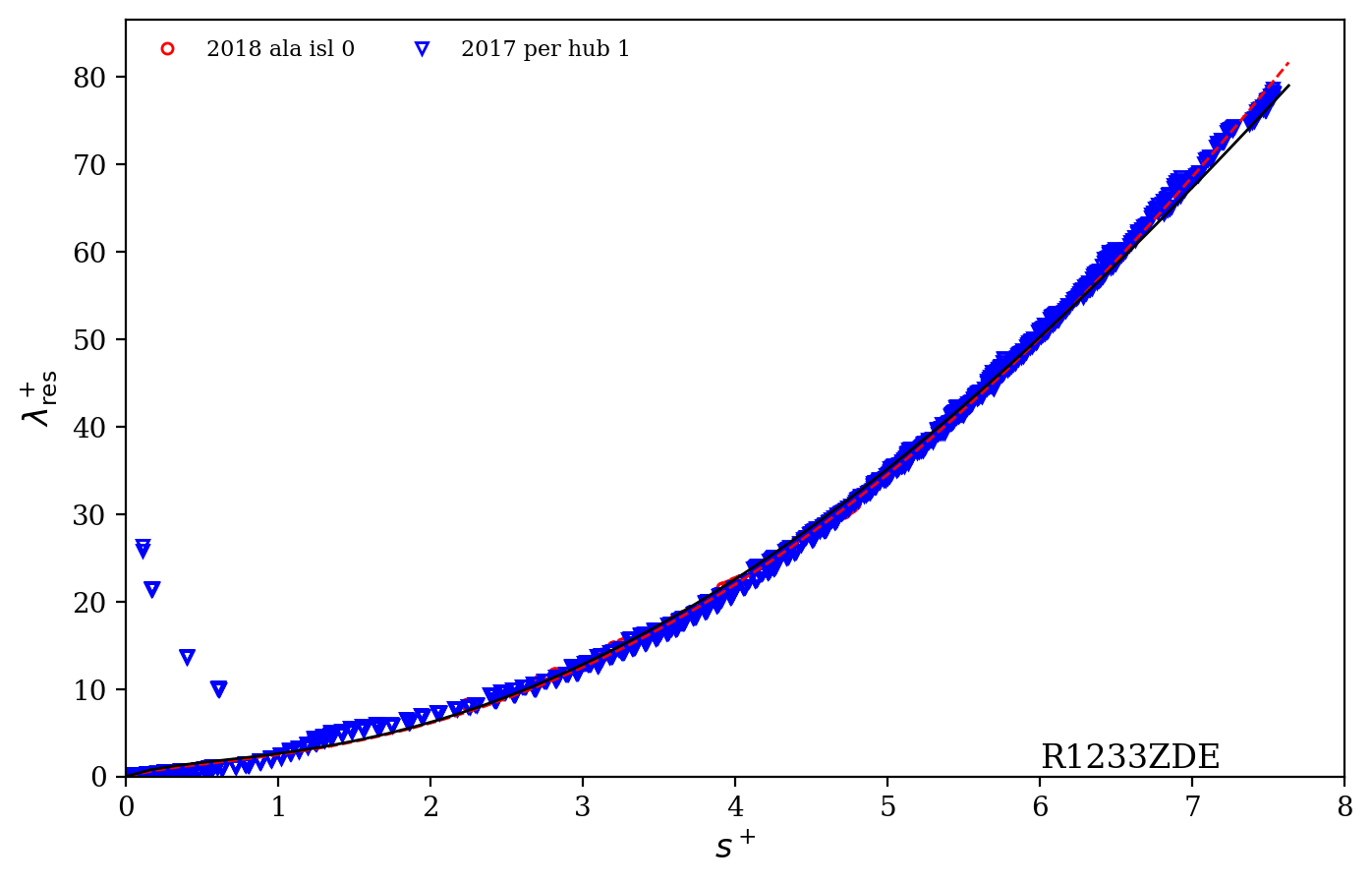

Supplement: Supplementary file 2 — ao4c10815_si_002.zip [file ao4c10815_si_002.zip › Supporting Information/Fig. TC1 - s_plus vs lambda_plus - all data - YFR EoS/R1233ZDE.png]

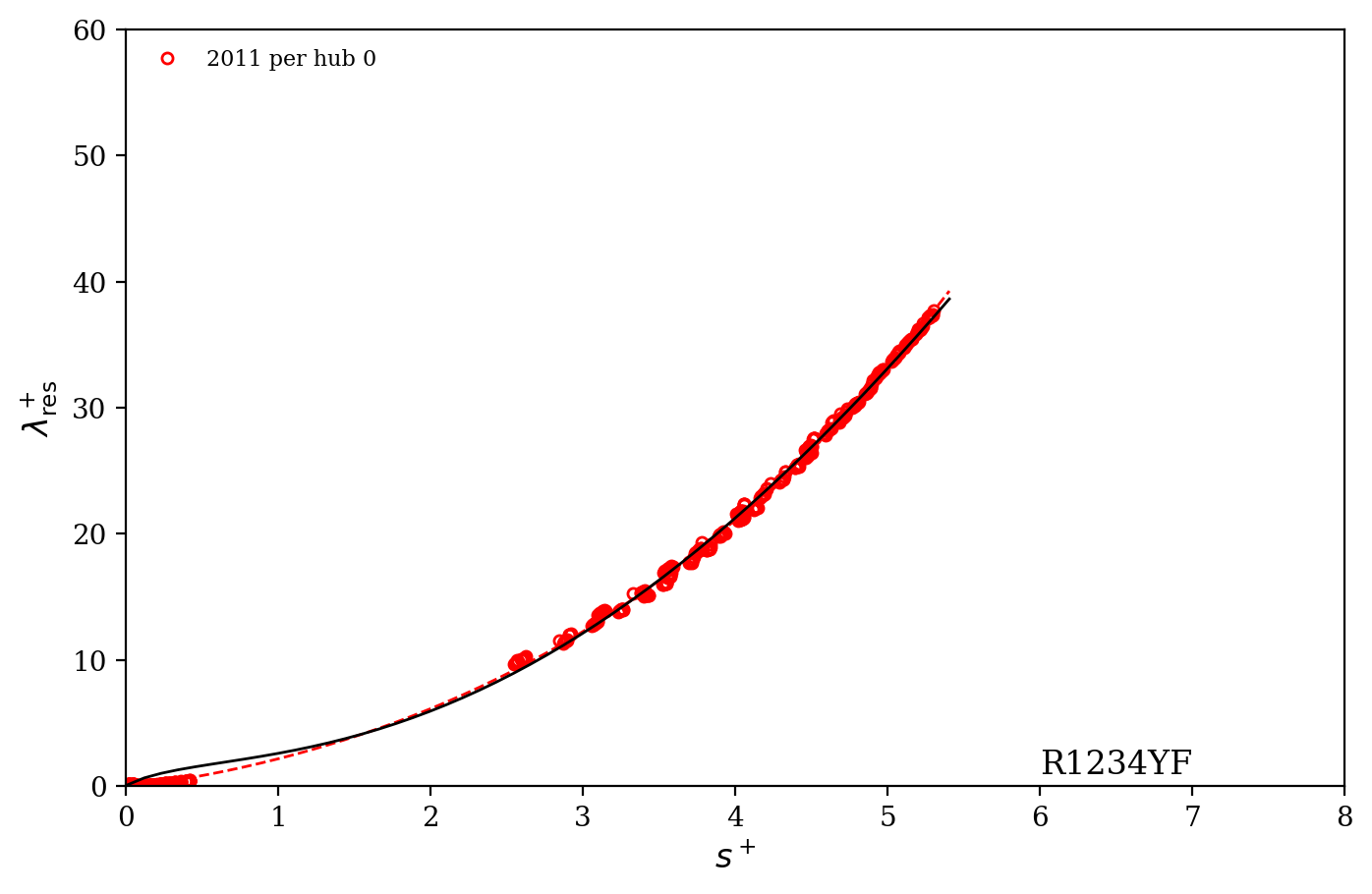

Supplement: Supplementary file 2 — ao4c10815_si_002.zip [file ao4c10815_si_002.zip › Supporting Information/Fig. TC1 - s_plus vs lambda_plus - all data - YFR EoS/R1234YF.png]

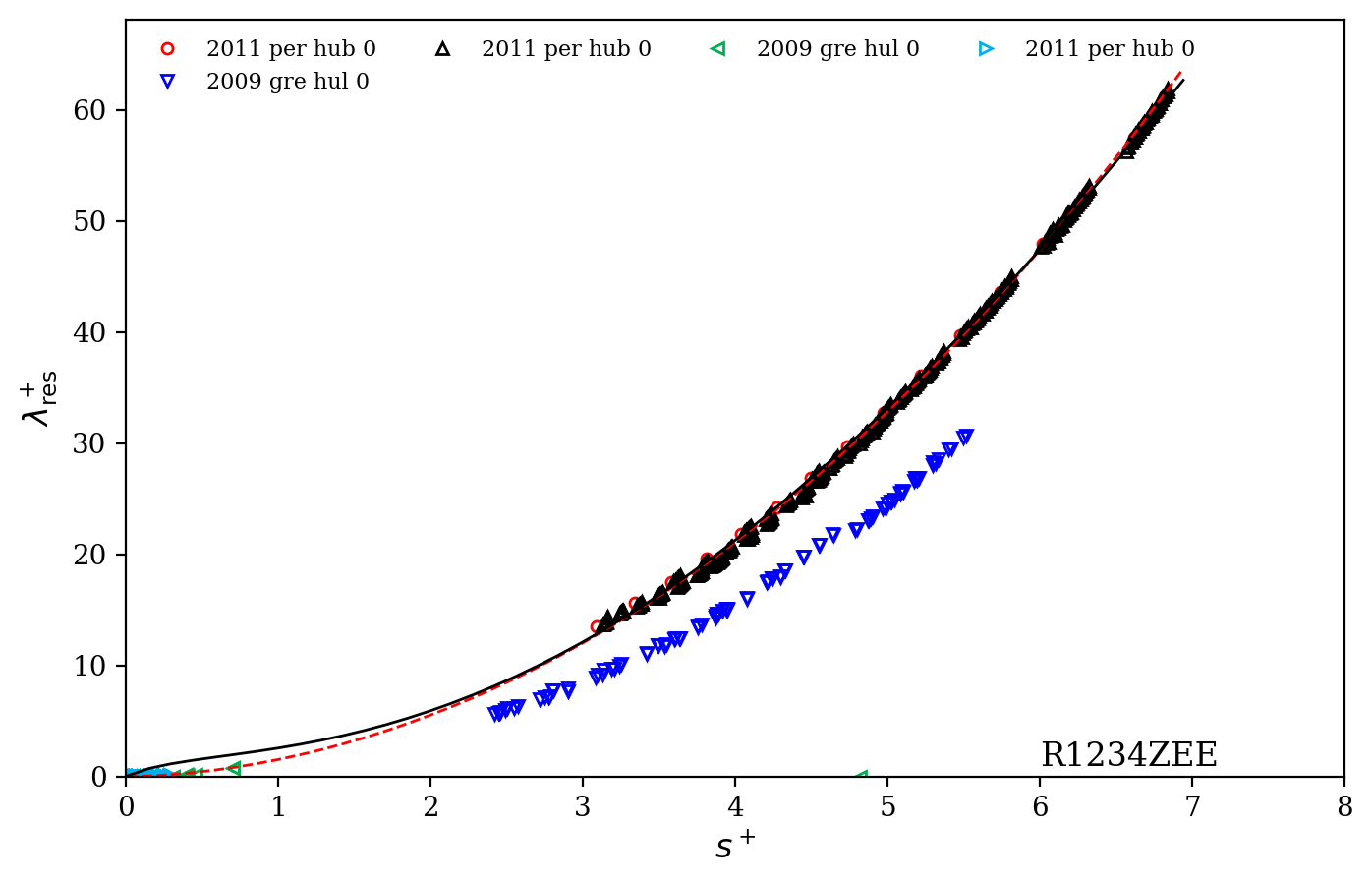

Supplement: Supplementary file 2 — ao4c10815_si_002.zip [file ao4c10815_si_002.zip › Supporting Information/Fig. TC1 - s_plus vs lambda_plus - all data - YFR EoS/R1234ZEE.png]

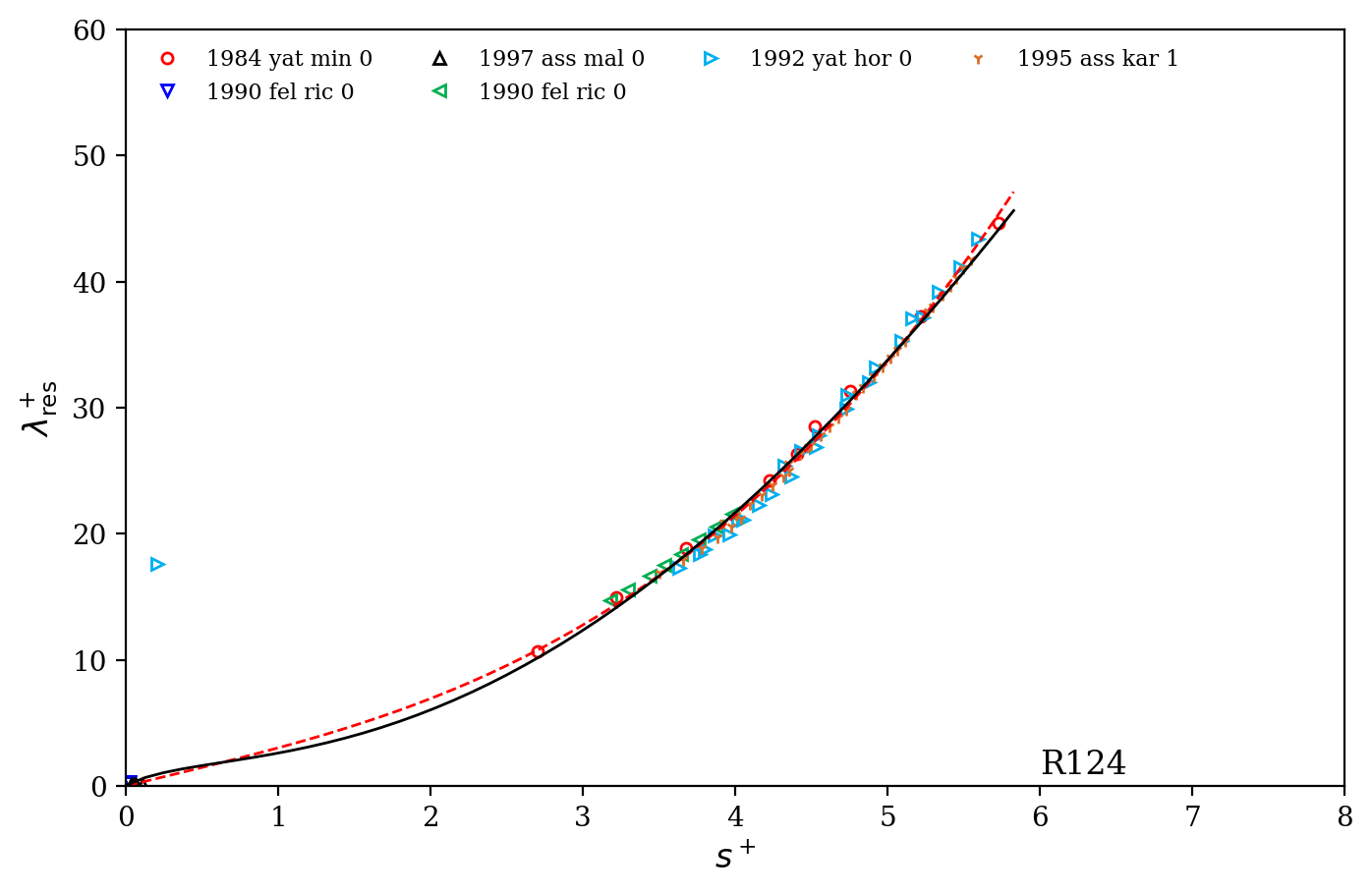

Supplement: Supplementary file 2 — ao4c10815_si_002.zip [file ao4c10815_si_002.zip › Supporting Information/Fig. TC1 - s_plus vs lambda_plus - all data - YFR EoS/R124.png]

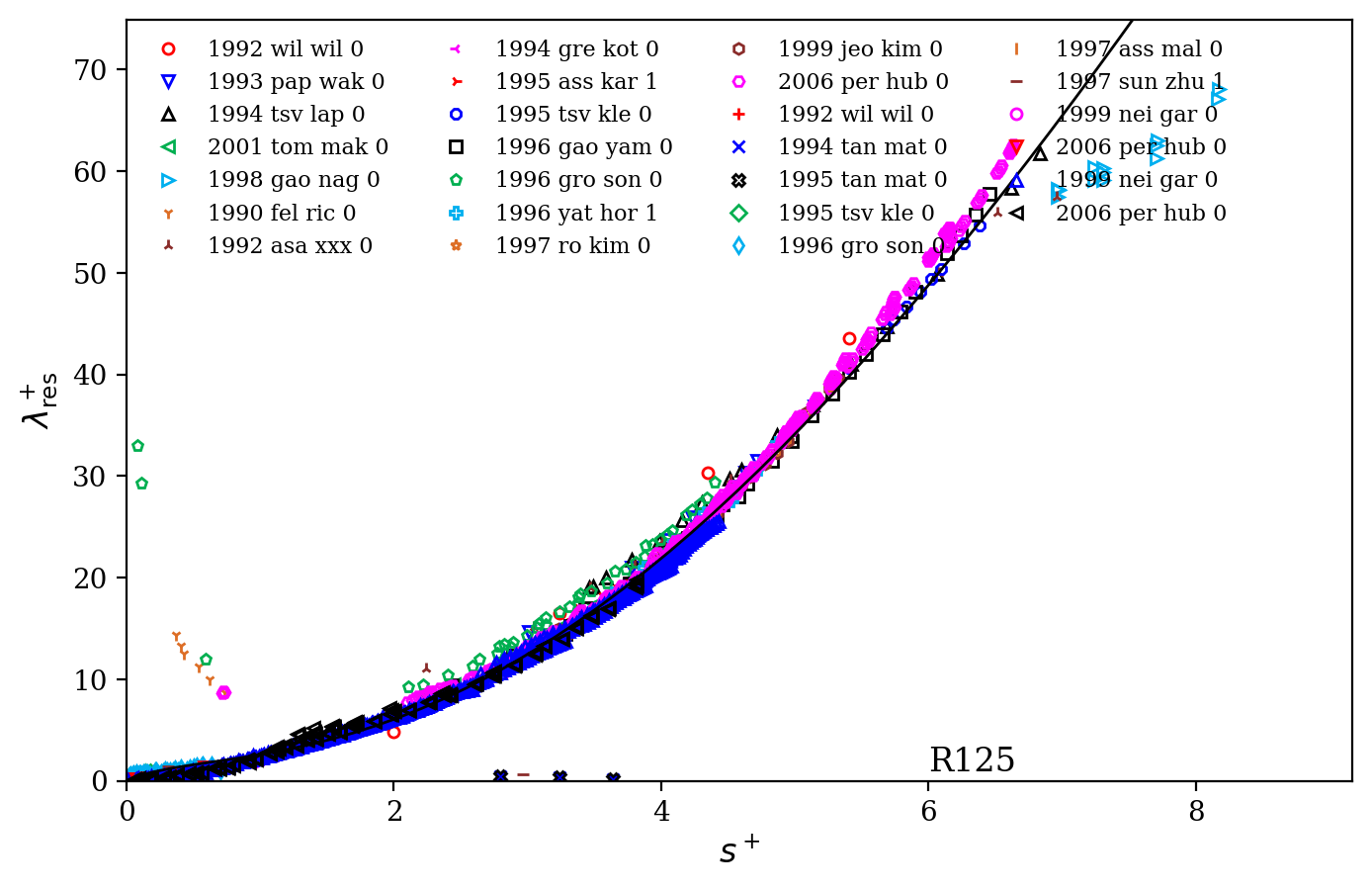

Supplement: Supplementary file 2 — ao4c10815_si_002.zip [file ao4c10815_si_002.zip › Supporting Information/Fig. TC1 - s_plus vs lambda_plus - all data - YFR EoS/R125.png]

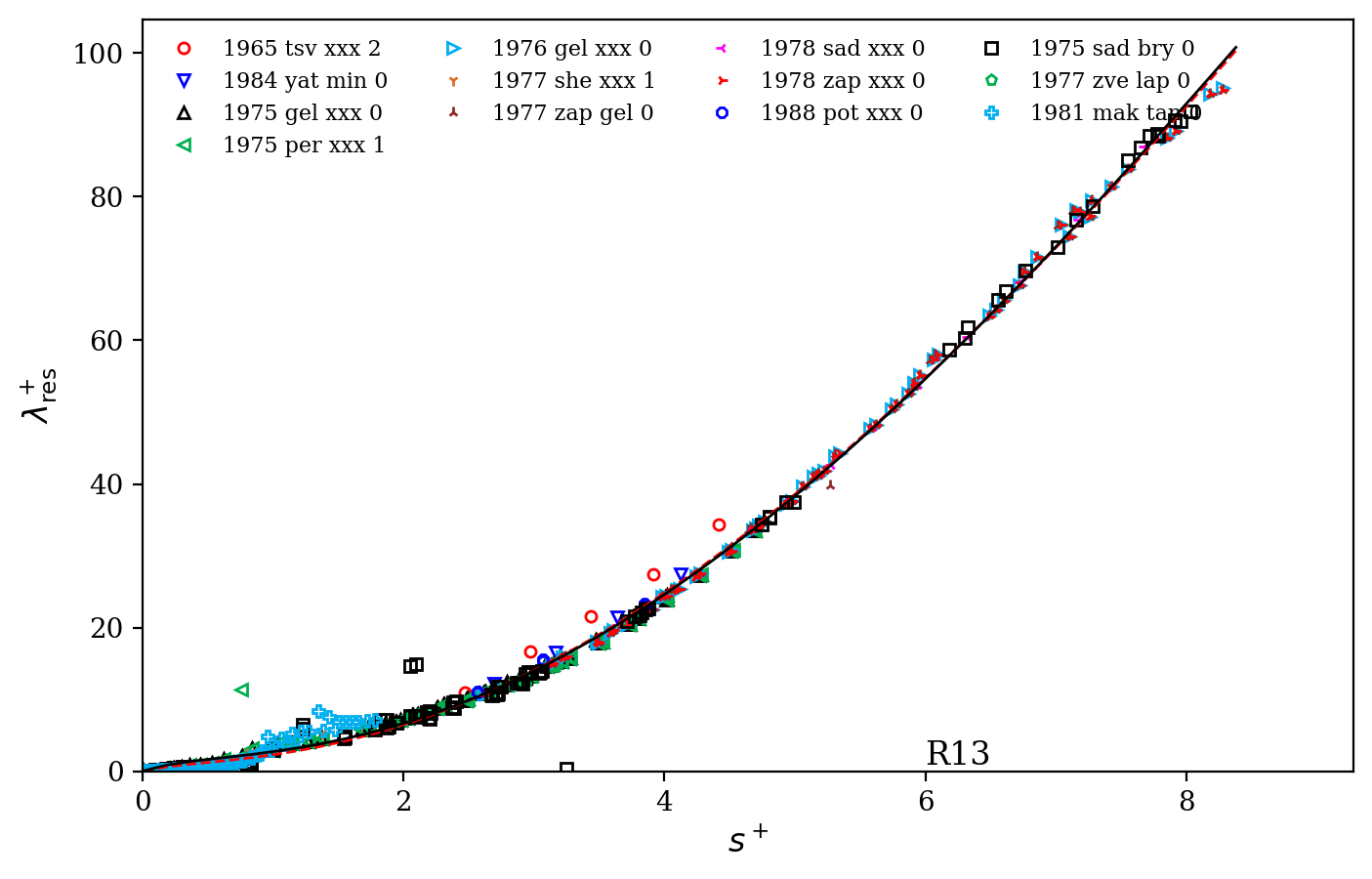

Supplement: Supplementary file 2 — ao4c10815_si_002.zip [file ao4c10815_si_002.zip › Supporting Information/Fig. TC1 - s_plus vs lambda_plus - all data - YFR EoS/R13.png]

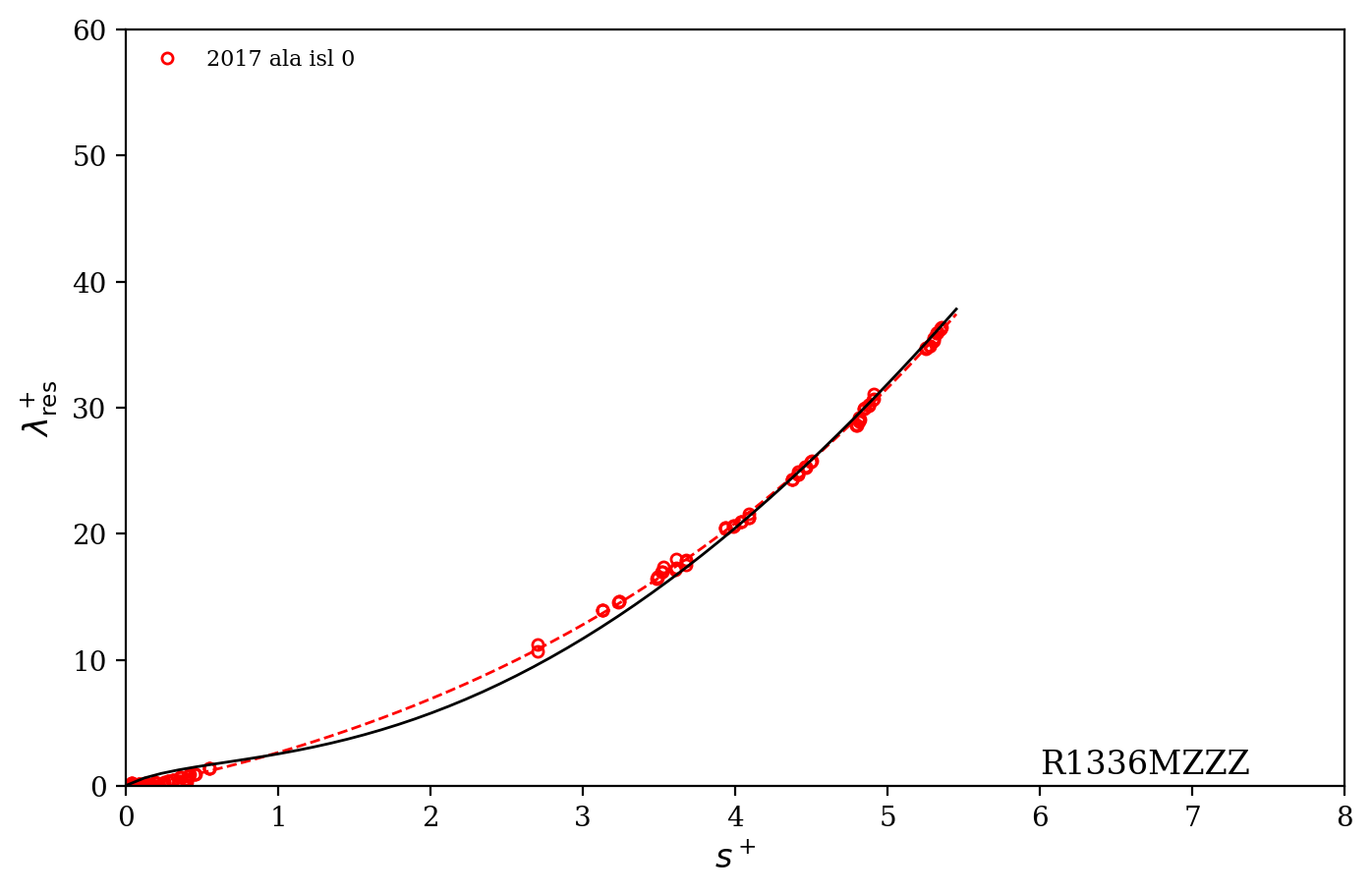

Supplement: Supplementary file 2 — ao4c10815_si_002.zip [file ao4c10815_si_002.zip › Supporting Information/Fig. TC1 - s_plus vs lambda_plus - all data - YFR EoS/R1336MZZZ.png]

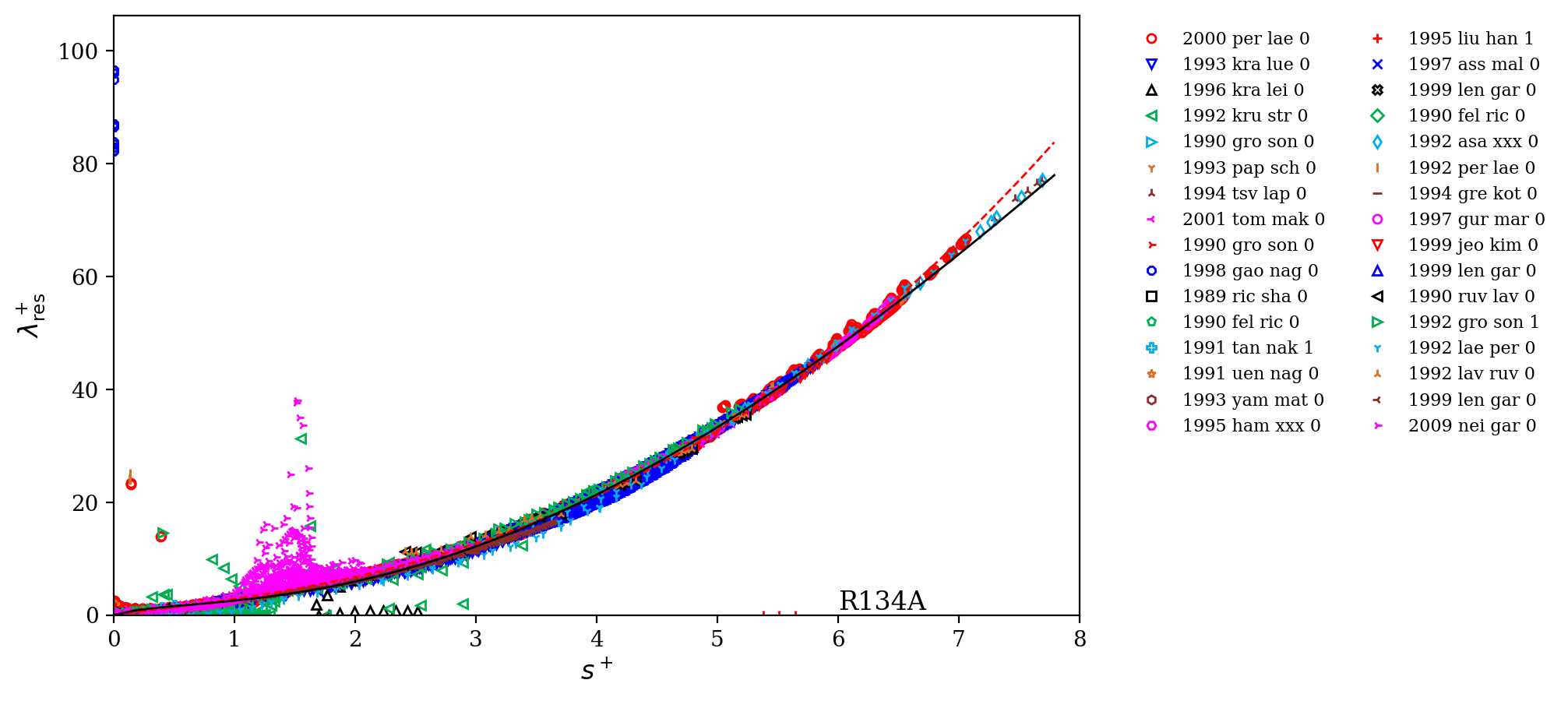

Supplement: Supplementary file 2 — ao4c10815_si_002.zip [file ao4c10815_si_002.zip › Supporting Information/Fig. TC1 - s_plus vs lambda_plus - all data - YFR EoS/R134A.png]

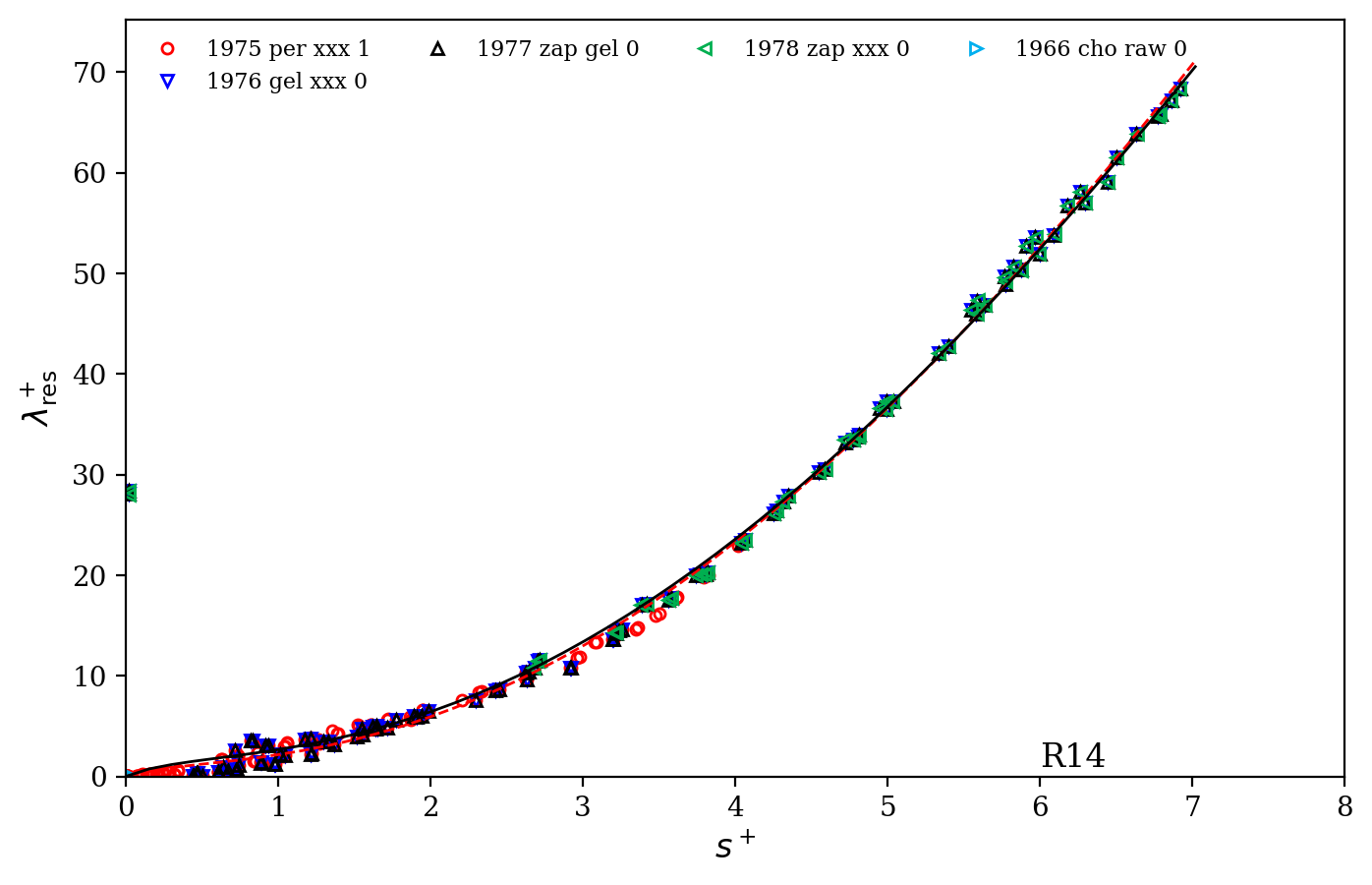

Supplement: Supplementary file 2 — ao4c10815_si_002.zip [file ao4c10815_si_002.zip › Supporting Information/Fig. TC1 - s_plus vs lambda_plus - all data - YFR EoS/R14.png]

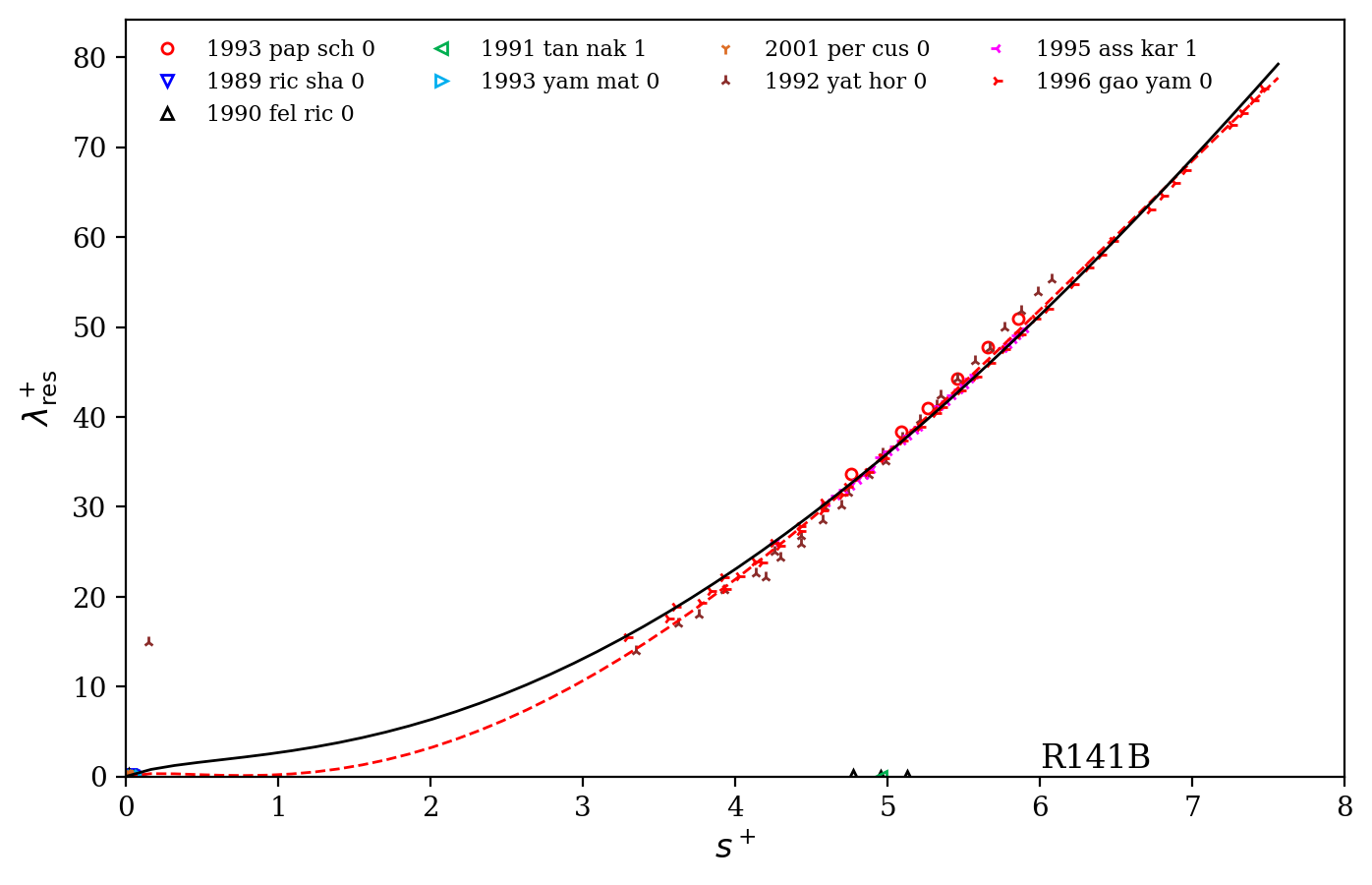

Supplement: Supplementary file 2 — ao4c10815_si_002.zip [file ao4c10815_si_002.zip › Supporting Information/Fig. TC1 - s_plus vs lambda_plus - all data - YFR EoS/R141B.png]

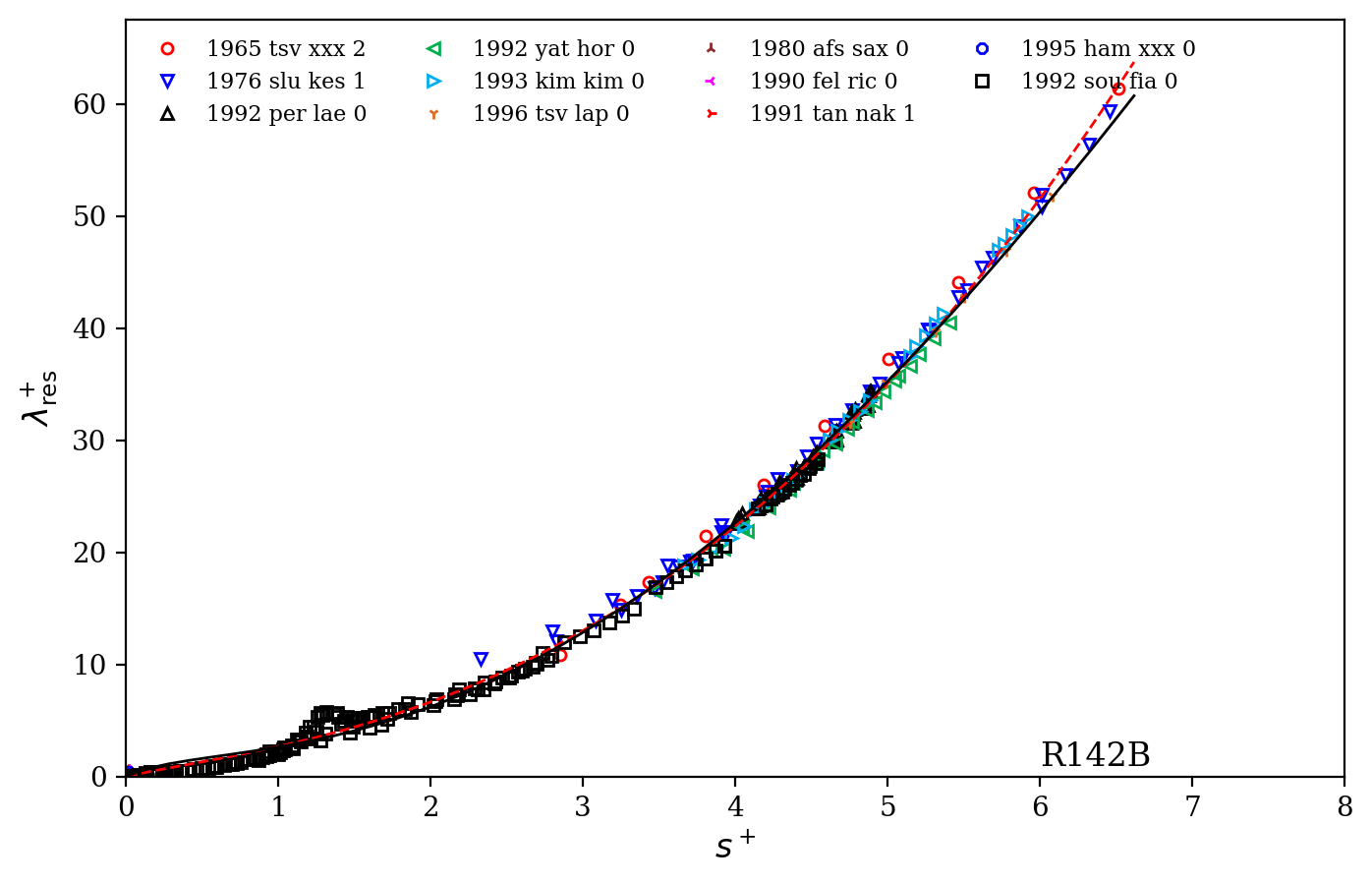

Supplement: Supplementary file 2 — ao4c10815_si_002.zip [file ao4c10815_si_002.zip › Supporting Information/Fig. TC1 - s_plus vs lambda_plus - all data - YFR EoS/R142B.png]

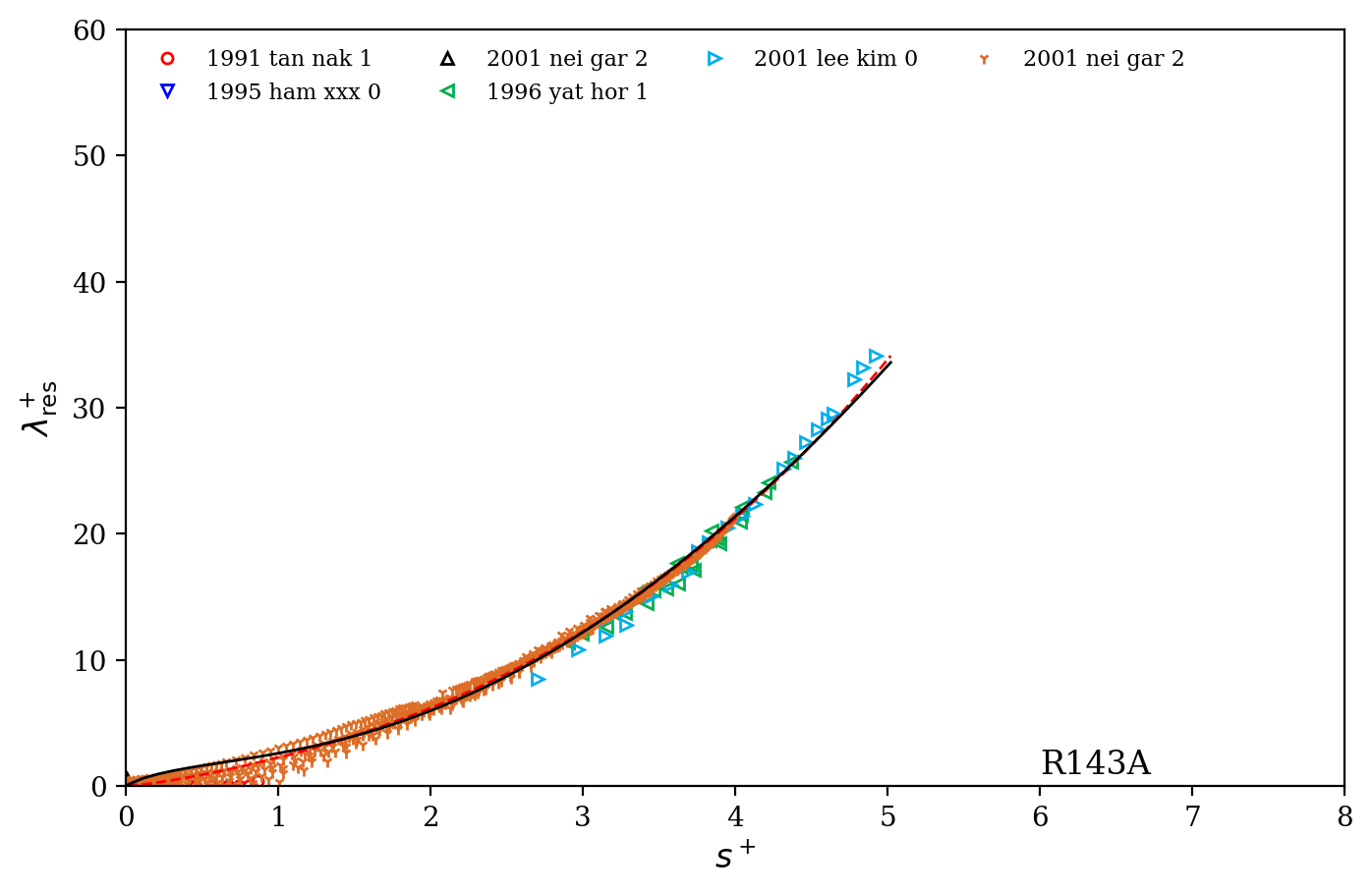

Supplement: Supplementary file 2 — ao4c10815_si_002.zip [file ao4c10815_si_002.zip › Supporting Information/Fig. TC1 - s_plus vs lambda_plus - all data - YFR EoS/R143A.png]

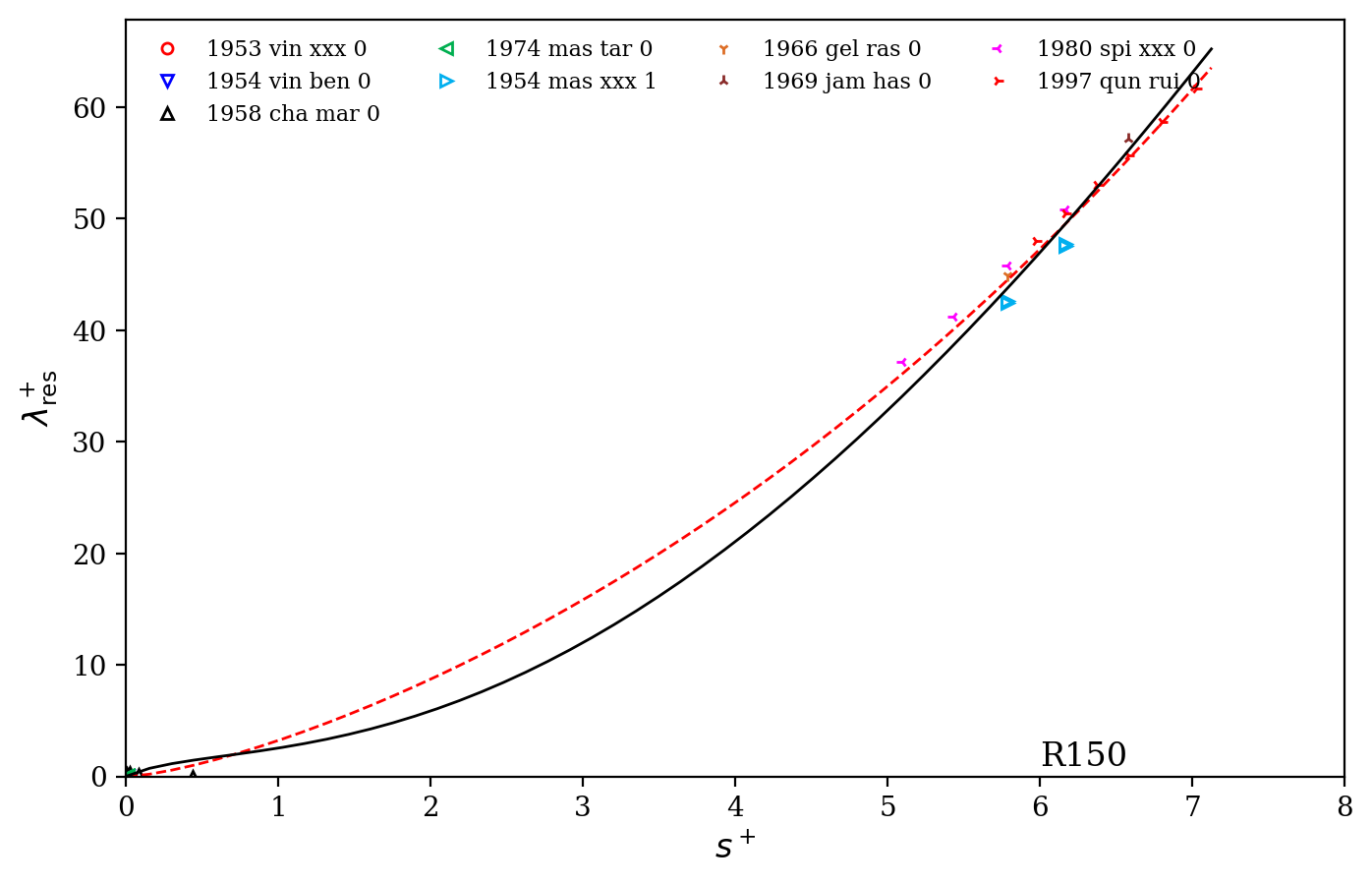

Supplement: Supplementary file 2 — ao4c10815_si_002.zip [file ao4c10815_si_002.zip › Supporting Information/Fig. TC1 - s_plus vs lambda_plus - all data - YFR EoS/R150.png]
